# Supplementary material for: Giant, swimming mouths: oral dimensions of extant sharks do not accurately predict body size in Dunkleosteus terrelli (Placodermi: Arthrodira)
Source: PeerJ. 2023 Apr 10;11:e15131. doi: 10.7717/peerj.15131 (PMC10100833; doi:10.7717/peerj.15131)
Supplement: Supplemental Information 4 — Original R code can be downloaded from this file as an .rmd document by selecting the option “Download Rmd” at the top of the document. [file peerj-11-15131-s004.html]

Supplementary Information for


Code 

- Show All Code
- Hide All Code
- Download Rmd

# Supplementary Information for

# “Giant, Swimming Mouths: Oral Dimensions of Extant Sharks Do Not Accurately Predict Body Size in *Dunkleosteus terrelli* (Placodermi: Arthrodira)”

#### *Russell Engelman*

#### *1/29/2022*

**Important Note:** If the R code (.rmd file) does not work when trying to rerun the analysis, one issue may be that the datasets (the .xlsx and/or .csv files) and additional images (i.e., the .png files) must be in the same working environment (usually the same folder) as the .rmd file. If possible, make sure the supplementary figures are in the same folder as the R Code. In the event that this is not possible, deactivate the chunks that contain references to supplementary figures or delete these lines from the R Code.

# 1 Loading in data

```
data_mouths<-readxl::read_excel("Arthrodire Mouths Supplementary File 1 (Data).xlsx") %>%
  filter(is.na(Specimen)|Specimen!="FMNH PF 1090") %>% #This specimen removed because its proportions are unusual and the specimen may not be complete
  rename(genus=Genus,
         species=Species,
         clade=Clade,
         order=Order,
         family=Family,
         shape=Shape,
         specimen=Specimen,
         ontogeny="Ontogenetic Status",
         extinct=Extinct,
         reference=Reference,
         total_length="Total Length (cm)",
         precaudal_length="Precaudal Length (cm)",
         head_length="Head Length (cm)",
         prebranchial_length="Prebranchial Length (cm)",
         mouth_length="Mouth Length (cm)",
         mouth_width="Mouth Width (cm)",
         inner_mouth_width="Inner Mouth Width (cm)",
         UJP="UJP (cm)")%>%
  mutate(shape=factor(shape,ordered=T,levels=c("macruriform","anguilliform","elongate",
                                               "fusiform","compressiform","flattened")),
         taxon = paste0(genus," ",species))
```

## 1.1 Separating out fossil taxa

```
(fossil_taxa_mouths<-data_mouths%>%
  filter(extinct==T)%>%
  mutate(specimen2=paste0(genus,"_",specimen))%>%
  column_to_rownames("specimen2"))
```

## 1.2 Loading shark data from Lowry et al. (2009) used by Ferron et al. 2017

```
lowry_sharks<-read_excel("Arthrodire Mouths Supplementary File 2 (Lowry et al. Shark Data).xlsx")%>%
  rename(UJP=`UJP(cm)`,total_length=`TBL(cm)`,fork_length=`Fork Length (cm)`,precaudal_length=`Precaudal Length (cm)`,taxon=Species)%>%
  mutate(species2=taxon)%>%
  separate(species2,c("genus","species"))%>%
  mutate(family=case_when(genus %in% c("Carcharodon","Isurus") ~ "Lamnidae",
                          genus %in% c("Sphyrna") ~ "Sphyrnidae",
                          genus %in% c("Carcharhinus","Galeocerdo","Negaprion") ~ "Carcharhinidae"
                          ),
         percent_body=precaudal_length/total_length)
```

## 1.3 Loading in jaw data for FSBC sharks

```
fsbc_sharks<-read_excel("Arthrodire Mouths Supplementary File 3 (FSBC Shark Jaws).xlsx")

#Adding rows for FSBC sharks to main dataset
data_mouths<-data_mouths%>%
  add_row(fsbc_sharks%>%
            select(taxon,specimen,mouth_width,UJP,total_length,precaudal_length)%>%
            separate(.,taxon,into=c("genus","species"),sep=" ",remove=F)%>%
            mutate(clade="Chondrichthyes",
                   reference="Present Study",
                   order=case_when(genus %in% c("Hexanchus","Heptranchias")~"Hexanchiformes",
                                   genus %in% c("Galeocerdo","Carcharhinus",
                                                "Hemipristis")~"Carcharhiniformes",
                                   genus %in% c("Odontaspis")~"Lamniformes"),
                   family=case_when(genus %in% c("Hexanchus","Heptranchias")~"Hexanchiidae",
                                   genus %in% c("Galeocerdo","Carcharhinus")~"Carcharhinidae",
                                   genus %in% c("Hemipristis")~"Hemigaleidae",
                                   genus %in% c("Odontaspis")~"Odontaspidae")))
```

# 2 Data

## 2.1 Measurements

| Measurement | Variable Name | Definition |
| --- | --- | --- |
| Total length | total\_length | Length from anterior tip of rostrum to posterior tip of caudal fin |
| Precaudal length | precaudal\_length | Length from anterior tip of rostrum to posterior tip of caudal peduncle |
| Head length | head\_length | Length from anterior tip of rostrum to posterior margin of branchial cavity   **For sharks:** from snout to opening of terminal gill arch   **For osteichthyans:** from snout to posterior end of operculum   **For arthrodires:** from snout to cranio-thoracic joint |
| Prebranchial length | prebranchial\_length | distance from tip of snout to anterior end of branchial skeleton   **For sharks:** to opening for first gill slit   **For osteichthyans:** to anterior end of opercular series   **For arthrodires:** not distinguishable |
| Upper jaw perimeter | UJP | Perimeter of upper jaw measured from bilateral angle of jaws |
| Mouth width | mouth\_width | Bilateral width of mouth across external angles of the jaw |
| Inner mouth width | inner\_mouth\_width | Bilateral width of mouth across inner angles of the jaw |
| Mouth length | mouth\_length | Length of mouth along anteroposterior axis   **For sharks:** anteroposterior length from the mandibular symphysis to the angle of the jaws   **For osteichthyans:** anteroposterior length of upper jaw   **For arthrodires:** anteroposterior length from tip of supragnathals to point where supraoral sensory line meets margin of jaw |

**Note:** Most of these differences between definitions in taxa are due to the presence of an operculum (in osteichthyans and chimaeroids) or the incorporation of the cranium and gill covers as single unit (i.e., the cheek plates of arthrodires). Thus, despite the differing definitions these measurements are anatomically homologous.

## 2.2 Taxonomic distribution of observations

```
data_mouths%>%
  drop_na(total_length)%>%
  group_by(clade)%>%
  summarise(N=n(),Taxa=n_distinct(taxon))%>%
  add_row(clade="All Species",
          N=data_mouths%>%
            drop_na(total_length)%>%
            summarise(N=n())%>%pull(),
          Taxa=data_mouths%>%
            drop_na(total_length)%>%
            summarise(Taxa=n_distinct(taxon))%>%pull())%>%
  kable(col.names=c("Clade","# Occurences","# Taxa"),
        align=c("l","c","c"),
        caption="Taxonomic distribution of observations made in this study. Placoderms for which only head dimensions are available (e.g., <i>Dunkleosteus</i>) are excluded from this table.")%>%
  row_spec(5, bold = T)%>%
  kable_styling()
```

Table 2.1: Taxonomic distribution of observations made in this study. Placoderms for which only head dimensions are available (e.g., *Dunkleosteus*) are excluded from this table.

| Clade | # Occurences | # Taxa |
| --- | --- | --- |
| Actinopterygii | 2356 | 792 |
| Chondrichthyes | 782 | 180 |
| Placodermi | 17 | 10 |
| Sarcopterygii | 16 | 3 |
| All Species | 3171 | 985 |

# 3 Additional notes on data collection

## 3.1 Method of collecting measurements in Arthrodira

In collecting data for arthrodires, it was often necessary to make use of reconstruction or composite specimens. This is because most complete arthrodires are preserved on flattened slabs, and thus it is not possible to measure total length and mouth dimensions in the same specimens. Even when arthrodires are extracted from the rock to create 3D reconstructions (e.g., the Gogo Formation arthrodires or *Plourdosteus* in Vézina 1988), this typically results in the post-thoracic axial skeleton being left behind because it is too fragile to mount. This means relative mouth size can only be measured in a very small number of arthrodires. Additional information on how measurements were collected in arthrodires are detailed below.

The early middle Devonian (Givetian) *Coccosteus cuspidatus* is the best known arthrodire in terms of its post-thoracic anatomy, having been described in detail by Miles & Westoll (1968). For this study, morphometric data for *Coccosteus cuspidatus* (UJP, mouth width, and head length) was taken from the reconstructions in Miles & Westoll (1968). This reconstructions are derived from several individuals, but when the dimensions of this reconstruction are compared to specimens of *Coccosteus* they appear to accurately reflect the proportions of this species. The reconstruction of *C. cuspidatus* in Miles & Westoll (1968: fig. 48) has a total length of 39.4 cm (scale is not provided for fig. 48, but total length can be estimated from head length, which is provided in fig. 23b).

Mouth width, but not UJP, could be measured in three additional arthrodires known from complete remains: *Holonema westolii* (see Miles & White 1971; Trinajstic 1999), *Plourdosteus canadensis* (see Vézina 1988; Vézina 1990), and *Incisoscutum ritchei* (see Dennis & Miles 1981; Trinajstic et al. 2013). Mouth width in two other species, *Watsonosteus fletti* and *Millerosteus minor*, could not be measured directly but could be approximated based on the preserved extent of the specimens (measuring from the approximate distance between the ends of the supraoral lateral line). For *H. westolii*, the skull of this taxon is figured in a splayed out, slightly crushed fashion in Miles & White (1971: fig. 4), but this depiction was considered close enough to the actual skull dimensions to highlight the traits of interest.

Measurements of *Plourdosteus canadensis* were taken from MNHN 2-177, which is (was) a complete skeleton of *P. canadensis* preserving the entire animal from the rostral plate to the tip of the vertebral column (only missing the soft tissue outline of the caudal fin). Originally preserved as a flattened plate, Vézina (1988) extracted the skull and trunk armor to produce a three-dimensional mount (Vézina 1988: plates 1-2), leaving the post-thoracic skeleton in this matrix (this material currently catalogued as MNHM 02-177c). Head and thoracic armor measurements were taken from the figures in Vézina (1988), whereas the post-thoracic armor length was measured based on photos of MNHM 02-177c provided by J. Kerr. Unfortunately, there are no pictures of this specimen prior to preparation (J. Kerr, pers. comm.), so it is difficult to determine exactly how long this specimen originally was. Assuming the remaining postthoracic material represents the body posterior to the end of the ventral shield, as implied by hollows left by the extraction of plates (posteroventrolaterals?) and a large element at the preserved anterior end of the skeleton that appears to represent a clasper or iliac process of the pelvic girdle MNHM 02-177c would have been about 37.5 cm long originally. The same author (Vézina 1990) later provides a full-body lateral reconstruction of *P. canadensis*. However, this reconstruction shows features that make its proportions suspect, including a post-thoracic region that appears to be directly copied from the figure of *C. cuspidatus* in Miles & Westoll (1968), albeit with a modified pelvic girdle, rather than reflecting the proportions of *Plourdosteus* specimens. Therefore, the preserved dimensions of MNHM 02-177 seem more reliable for estimating the proportions of this species. It was not possible to contact D. Vézina for clarification on this specimen as this researcher is now deceased (J. Kerr, pers. comm.).

Finally, for *Incisoscutum ritchei*, the reconstruction presented in Trinajstic et al. (2013: fig. 1c) was used as a starting point, and then the material was cross-referenced and scaled with *Incisoscutum* fossil material (NHMUK P50923 and WAM 0.3.3.28). The proportions of this reconstruction were similar enough to actual fossils of *I. ritchei* that this reconstruction was considered a reliable representation of the proportions of *Incisoscutum*. Mouth width for *Incisoscutum* was calculating by scaling the mouth width of NKMUK PV P 50929 (Dennis & Miles 1981: fig. 9) to the size of the reconstruction in Trinajstic et al. (2013) using skull length. This ensured mouth width was measured directly from fossil specimens of *I. ritchei*. The skull of NHMUK PV P 50929 is similar in size to the reconstruction in Trinajstic et al. (2013) (5.75 versus 5.74 cm), suggesting bias from allometric scaling, if it exists, should be minor.

Notably, the reconstruction of *Incisoscutum* in Trinajstic et al. (2013: fig. 1c) lacks the caudal fin. This is because none of the eubrachythoracid arthrodires from the Gogo Formation preserves an associated caudal fin (Trinajstic et al. 2022; Trinajstic pers. comm.). Thus, despite the Gogo Formation producing a significant number of extremely well-preserved arthrodire specimens, none of this material can be used to determine the body proportions or overall length of arthrodires. Because complete arthrodires are so rare and the precaudal anatomy of *Incisoscutum* is completely known, total length was estimated for *I. ritchei* assuming the the caudal fin represented ~32% of total length (i.e., total length/precaudal length = 1.46). This is the proportion between caudal fin length and total length in the reconstruction of *Coccosteus cuspidatus* in Miles and Westoll (1968). Additionally, this ratio is similar to the total length/precaudal length ratio in many extant sharks (see below), suggesting it represents a reasonable approximation for *I. ritchei*.

For *Millerosteus minor*, initially no complete specimens of *Millerosteus minor* could be observed for this study. Instead, composite values for *Millerosteus* were calculated using the armor proportions of the specimen described by Desmond (1974) (C.369 of that study, currently cataloged as LDUCZ-V998), which preserves most of the armor but very little of the axial skeleton (Desmond 1974: pl. 1) and the postthoracic dimensions of an undescribed specimen of M. minor collected by M. J. Newman. The Newman specimen is missing the middle caudal vertebrae but preserves the remainder of the post-thoracic skeleton, including the dorsal fin, pelvic girdle, anal plate, and distal caudal vertebrae to the caudal fin tip. The specimen measured 6.325 cm from the snout to the end of the ventral armor and has an approximate total length of 17 cm. The postthoracic dimensions of this specimen were scaled to that of LDUCZ-V998 using the length of the specimen to the end of the ventral armor. The skull of Newman’s specimen is poorly preserved, the outline of the head can be distinguished but the rest of the cranial anatomy cannot. Thus, it cannot be used on its own. Mouth width in *Millerosteus* was approximated by measuring between the ventral ends of the supraoral sensory canal. Late in the study it was possible to access specimens of *Millerosteus* in the Field Museum of Natural History, but mouth width could not be measured in these specimens.

## 3.2 Length-length equations for sharks

In evaluating the dataset of Lowry et al. (2009) it was also of interest to see whether caudal fin proportions between the various groups examined might signficantly bias the allometric relationships seen between UJP and total length in sharks. Therefore, total length was converted to precaudal length for each shark using the length-length equations in Branstetter & Stiles (1987), Cliff (1995), Francis (2006), Piercy et al. (2007), Whitney & Crow (2007), and Pollack et al. (2019). For *Isurus paucus* no length-length equation was available, so one was created using the data from Garrick (1967) and Gilmore (1983).

## 3.3 Note on the ventral armor of *Dunkleosteus*

For the reconstruction of the armor of *Dunkleosteus* in the manuscript, the specimen is depicted as is with no alterations. The figure was drawn directly from images of the specimen, and so represents the actual proportions of the material.

The only modification made was that the *Dunkleosteus* specimens were reconstructed with a curved ventral shield, rather than the flattened one seen in the mounted specimens. This is because three-dimensionally preserved fossils of arthrodires show the ventral armor of marine arthrodires was naturally curved (e.g., Gardiner & Miles 1994; Miles & Dennis 1979), and thus these animals had a torpedo-like cross-section. However, this discovery was made long after the *Dunkleosteus* specimens at the CMNH were mounted (circa 1929; J. Tait, pers. comm.). However, after it was discovered arthrodires naturally had curved ventral armor, retrodeformation of the ventral shield was deemed too destructive to the original material because it involved manually breaking and resetting the specimens (D. Chapman, pers. comm.). Thus, the CMNH *Dunkleosteus* have retained their flattened ventral armor despite this feature otherwise being outdated. Despite being mounted with flat ventral armor, depicting the specimens as such would not accurately depict the anatomy of this taxon and would result in the body appearing unrealistically boxy. Thus, the ventral curvature of the armor was approximated based on the curvature of the specimens.

# 4 Shark UJP plot

```
(lowry2009<-ggplot(lowry_sharks,aes(y=UJP,x=total_length))+
  geom_point(aes(fill="All Species",shape="All Species"))+
  geom_point(aes(fill=family,shape=family))+
  geom_smooth(method="lm",formula=y~x,aes(color="All Species"))+
  geom_smooth(method="lm",formula=y~x,aes(color=family,fill=family),alpha=0.25)+
  geom_smooth(method="lm",formula=y~x,color="black",alpha=0.25)+
  scale_color_manual(values=c("black",hue_pal()(3)))+
  scale_fill_manual(values=c("NA",hue_pal()(3)),na.value="NA")+
  scale_shape_manual(values=c(NA,21,22,24),na.value=NA)+
  labs(x="Total Length (cm)",y="Upper Jaw Perimeter (cm)",
       color="Family",shape="Family",fill="Family")+
  theme_classic()+
  theme(legend.position=c(0.8,0.25)))
```

Figure 4.1: Plot of upper jaw perimeter against total length (both untransformed) from Lowry et al. (2009), showing the different allometric patterns in Sphyrnidae, Lamnidae, and Carcharhinidae relative to the all species regression model.

```
ggsave("Arthrodire Mouths (Lowry 2009 regression).pdf",lowry2009,width=165,height=125,units="mm",
       device="pdf")
```

Note the positive allometry in upper jaw perimeter versus total length due to the fact that the very largest measured sharks all pertain to *Carcharodon carcharias*, thus causing the slope of the regression line to be higher (i.e., produce slightly shorter lengths) at larger values of `UJP`, despite the three groups not showing such a relationship by themselves.

```
summary(lm(total_length~UJP*family,lowry_sharks))
```

```
## 
## Call:
## lm(formula = total_length ~ UJP * family, data = lowry_sharks)
## 
## Residuals:
##     Min      1Q  Median      3Q     Max 
## -59.509 -13.308  -1.223  11.013  92.409 
## 
## Coefficients:
##                      Estimate Std. Error t value Pr(>|t|)    
## (Intercept)           20.3894     4.6947   4.343 2.08e-05 ***
## UJP                    6.3607     0.1597  39.834  < 2e-16 ***
## familyLamnidae       -22.9041     8.9928  -2.547   0.0115 *  
## familySphyrnidae      53.6133    13.5455   3.958 9.97e-05 ***
## UJP:familyLamnidae    -0.5246     0.2184  -2.402   0.0171 *  
## UJP:familySphyrnidae   0.3711     0.4694   0.791   0.4300    
## ---
## Signif. codes:  0 '***' 0.001 '**' 0.01 '*' 0.05 '.' 0.1 ' ' 1
## 
## Residual standard error: 22.21 on 239 degrees of freedom
## Multiple R-squared:  0.9443, Adjusted R-squared:  0.9431 
## F-statistic: 810.6 on 5 and 239 DF,  p-value: < 2.2e-16
```

Based on this, it can be seen that sphyrnids and carcharhinids have similar slopes (but carcharhinids and lamnids do not), and the intercepts between the equations of the three groups are all significantly different from one another.

## 4.1 Using Precaudal Length

```
ggplot(lowry_sharks,aes(y=precaudal_length,x=total_length))+
  geom_point(aes(fill="All Species",shape="All Species"))+
  geom_point(aes(fill=family,shape=family))+
  geom_smooth(method="lm",formula=y~x,aes(color="All Species"))+
  geom_smooth(method="lm",formula=y~x,aes(color=family,fill=family),alpha=0.25)+
  geom_smooth(method="lm",formula=y~x,color="black",alpha=0.25)+
  scale_color_manual(values=c("black",hue_pal()(3)))+
  scale_fill_manual(values=c("NA",hue_pal()(3)),na.value="NA")+
  scale_shape_manual(values=c(NA,21,22,24),na.value=NA)+
  labs(x="Precaudal Length (cm)",y="Upper Jaw Perimeter (cm)",
       color="Family",shape="Family",fill="Family")+
  theme_classic()+
  theme(legend.position=c(0.8,0.25))
```

Figure 4.2: Plot of upper jaw perimeter against precaudal length (both untransformed) from Lowry et al. (2009), showing the different allometric patterns in Sphyrnidae, Lamnidae, and Carcharhinidae relative to the all species regression model.

```
summary(lm(precaudal_length~UJP*family,lowry_sharks))
```

```
## 
## Call:
## lm(formula = precaudal_length ~ UJP * family, data = lowry_sharks)
## 
## Residuals:
##     Min      1Q  Median      3Q     Max 
## -48.705  -9.608  -0.221   7.057  66.904 
## 
## Coefficients:
##                      Estimate Std. Error t value Pr(>|t|)    
## (Intercept)            2.9875     3.5275   0.847   0.3979    
## UJP                    5.1667     0.1200  43.063  < 2e-16 ***
## familyLamnidae       -15.3722     6.7571  -2.275   0.0238 *  
## familySphyrnidae      48.1398    10.1779   4.730 3.84e-06 ***
## UJP:familyLamnidae    -0.1808     0.1641  -1.102   0.2716    
## UJP:familySphyrnidae  -0.4061     0.3527  -1.151   0.2507    
## ---
## Signif. codes:  0 '***' 0.001 '**' 0.01 '*' 0.05 '.' 0.1 ' ' 1
## 
## Residual standard error: 16.69 on 239 degrees of freedom
## Multiple R-squared:  0.9556, Adjusted R-squared:  0.9547 
## F-statistic:  1029 on 5 and 239 DF,  p-value: < 2.2e-16
```

When considering precaudal length, the slopes of the three groups are nonsignificantly different, but the intercepts between the three regression equations are. This suggests the differences in slope in the equation considering total length are due to differing caudal fin proportions between the three groups, but it also suggests that the different UJP-total length relationships cannot be attributed to differencs in caudal fin shape alone (or else the intercepts would not differ between groups).

```
lowry_sharks%>%
  group_by(taxon)%>%
  summarise(mean=round(mean(percent_body)*100,1),sd=round(sd(percent_body)*100,3),family=unique(family))
```

```
lowry_sharks%>%
  group_by(taxon)%>%
  summarise(mean=mean(percent_body),sd=sd(percent_body),family=unique(family))%>%
  group_by(family)%>%
  summarise(mean=round(mean(mean)*100,1))
```

# 5 Comparison of estimates using upper jaw perimeter and mouth width

## 5.1 Specimen-level analysis

**Note:** *Alopias* spp. and Chimaeriformes were excluded for this model.

```
fit.mouthwidth<-lm(log(total_length)~log(mouth_width),
                   data_mouths%>%
                     filter(clade=="Chondrichthyes",
                            order != "Chimaeriformes",
                            genus!="Alopias"))
summary(fit.mouthwidth)
```

```
## 
## Call:
## lm(formula = log(total_length) ~ log(mouth_width), data = data_mouths %>% 
##     filter(clade == "Chondrichthyes", order != "Chimaeriformes", 
##         genus != "Alopias"))
## 
## Residuals:
##      Min       1Q   Median       3Q      Max 
## -0.88416 -0.13332  0.00318  0.13206  0.62083 
## 
## Coefficients:
##                  Estimate Std. Error t value Pr(>|t|)    
## (Intercept)       2.89884    0.02241  129.38   <2e-16 ***
## log(mouth_width)  0.81568    0.01032   79.06   <2e-16 ***
## ---
## Signif. codes:  0 '***' 0.001 '**' 0.01 '*' 0.05 '.' 0.1 ' ' 1
## 
## Residual standard error: 0.2114 on 623 degrees of freedom
##   (117 observations deleted due to missingness)
## Multiple R-squared:  0.9094, Adjusted R-squared:  0.9092 
## F-statistic:  6251 on 1 and 623 DF,  p-value: < 2.2e-16
```

```
regression.stats(fit.mouthwidth)
```

### 5.1.1 Diagnostic plots

```
par(mfrow=c(2,2))
plot(fit.mouthwidth)
```

Based on these diagnostic plots the residuals are normally distributed, homoskedastic, have no outliers with significant leverage, and suggest a linear model, thus should be safe to use to predict new data.

### 5.1.2 Allometric relationship

For this analysis `total_length` was set as the independent variable, to better express changes in proportion in `mouth_width` across chondrichthyans of different sizes.

```
data_mouths%>%
  filter(clade=="Chondrichthyes",!order %in% c("Chimaeriformes"),
         genus!="Alopias")%>%
  drop_na(mouth_width,total_length)%$%
  lm(log(mouth_width)~log(total_length),.)%>%
  summary()
```

```
## 
## Call:
## lm(formula = log(mouth_width) ~ log(total_length), data = .)
## 
## Residuals:
##      Min       1Q   Median       3Q      Max 
## -0.68930 -0.16680 -0.01405  0.15583  0.99567 
## 
## Coefficients:
##                   Estimate Std. Error t value Pr(>|t|)    
## (Intercept)       -3.04952    0.06477  -47.08   <2e-16 ***
## log(total_length)  1.11486    0.01410   79.06   <2e-16 ***
## ---
## Signif. codes:  0 '***' 0.001 '**' 0.01 '*' 0.05 '.' 0.1 ' ' 1
## 
## Residual standard error: 0.2471 on 623 degrees of freedom
## Multiple R-squared:  0.9094, Adjusted R-squared:  0.9092 
## F-statistic:  6251 on 1 and 623 DF,  p-value: < 2.2e-16
```

## 5.2 Examining the allometric relationship of mouth width using species averages

### 5.2.1 Regression equation

**Note:** *Alopias* spp. and Chimaeriformes were excluded for this model. Only adult and subadult (that is, large but not sexually mature) specimens were considered for this analysis.

```
fit.mouthwidth_averages<-data_mouths%>%
  filter(is.na(ontogeny)|ontogeny %in% c("adult","subadult"),
         clade=="Chondrichthyes",!order %in% c("Chimaeriformes"),
         genus!="Alopias")%>%
  drop_na(mouth_width,total_length)%>%
  group_by(taxon)%>%
  summarise(mouth_width=mean(mouth_width),total_length=mean(total_length),order=unique(order))%$%
  lm(log(total_length)~log(mouth_width),.)

summary(fit.mouthwidth_averages)
```

```
## 
## Call:
## lm(formula = log(total_length) ~ log(mouth_width), data = .)
## 
## Residuals:
##      Min       1Q   Median       3Q      Max 
## -0.68961 -0.13979  0.01145  0.15040  0.47539 
## 
## Coefficients:
##                  Estimate Std. Error t value Pr(>|t|)    
## (Intercept)       2.93694    0.05053   58.12   <2e-16 ***
## log(mouth_width)  0.80555    0.02277   35.37   <2e-16 ***
## ---
## Signif. codes:  0 '***' 0.001 '**' 0.01 '*' 0.05 '.' 0.1 ' ' 1
## 
## Residual standard error: 0.2191 on 154 degrees of freedom
## Multiple R-squared:  0.8904, Adjusted R-squared:  0.8897 
## F-statistic:  1251 on 1 and 154 DF,  p-value: < 2.2e-16
```

```
regression.stats(fit.mouthwidth_averages)
```

### 5.2.2 Allometric relationship

For this analysis `total_length` was set as the independent variable, to better express changes in proportion in `mouth_width` across chondrichthyans of different sizes.

```
data_mouths%>%
  filter(is.na(ontogeny)|ontogeny %in% c("adult","subadult"),
         clade=="Chondrichthyes",!order %in% c("Chimaeriformes"),
         genus!="Alopias")%>%
  drop_na(mouth_width,total_length)%>%
  group_by(taxon)%>%
  summarise(mouth_width=mean(mouth_width),total_length=mean(total_length),order=unique(order))%$%
  lm(log(mouth_width)~log(total_length),.)%>%
  summary()
```

```
## 
## Call:
## lm(formula = log(mouth_width) ~ log(total_length), data = .)
## 
## Residuals:
##      Min       1Q   Median       3Q      Max 
## -0.58011 -0.17469 -0.01593  0.16287  0.73161 
## 
## Coefficients:
##                   Estimate Std. Error t value Pr(>|t|)    
## (Intercept)       -3.01826    0.14561  -20.73   <2e-16 ***
## log(total_length)  1.10534    0.03125   35.37   <2e-16 ***
## ---
## Signif. codes:  0 '***' 0.001 '**' 0.01 '*' 0.05 '.' 0.1 ' ' 1
## 
## Residual standard error: 0.2567 on 154 degrees of freedom
## Multiple R-squared:  0.8904, Adjusted R-squared:  0.8897 
## F-statistic:  1251 on 1 and 154 DF,  p-value: < 2.2e-16
```

## 5.3 Comparison of estimates using UJP and mouth width in arthrodires

```
fit.UJP<-lm(total_length~UJP,lowry_sharks)
summary(fit.UJP)
```

```
## 
## Call:
## lm(formula = total_length ~ UJP, data = lowry_sharks)
## 
## Residuals:
##     Min      1Q  Median      3Q     Max 
## -64.184 -24.385  -7.079  18.818 159.091 
## 
## Coefficients:
##             Estimate Std. Error t value Pr(>|t|)    
## (Intercept)  48.6599     5.0565   9.623   <2e-16 ***
## UJP           5.3305     0.1396  38.185   <2e-16 ***
## ---
## Signif. codes:  0 '***' 0.001 '**' 0.01 '*' 0.05 '.' 0.1 ' ' 1
## 
## Residual standard error: 35.28 on 243 degrees of freedom
## Multiple R-squared:  0.8572, Adjusted R-squared:  0.8566 
## F-statistic:  1458 on 1 and 243 DF,  p-value: < 2.2e-16
```

```
# Predictions of total length using UJP and mouth width
mouth_predictions<-fossil_taxa_mouths%>%
  drop_na(UJP,mouth_width)%>%
  augment(x=fit.UJP,newdata=.,interval="prediction") %>% 
  rename_at(vars(starts_with('.')), funs(paste0('fit.UJP', .)))%>%
  augment(x=fit.mouthwidth,newdata=.,interval="prediction") %>% 
  rename_at(vars(starts_with('.')), funs(paste0('fit.mouth_width', .)))%>%
  rename(mouth_width_original=mouth_width,
         mouth_width=inner_mouth_width)%>%
  augment(x=fit.mouthwidth,newdata=.,
          interval="prediction") %>%
  rename_at(vars(starts_with('.')), funs(paste0('fit.inner_mouth_width', .)))%>%
  rename(mouth_width=mouth_width_original,
         inner_mouth_width=mouth_width)%>%
  mutate(across(fit.mouth_width.fitted:fit.inner_mouth_width.upper,
                ~exp(.)*regression.stats(fit.mouthwidth)$CF),
         fit.UJP.range=paste0("(",round(fit.UJP.lower,1),"–",round(fit.UJP.upper,1),")"),
         fit.mouth_width.range=paste0("(",round(fit.mouth_width.lower,1),"–",
                                      round(fit.mouth_width.upper,1),")"),
         fit.inner_mouth_width.range=paste0("(",round(fit.inner_mouth_width.lower,1),"–",
                                      round(fit.inner_mouth_width.upper,1),")"))
# Kable of results
mouth_predictions%>%
  arrange(UJP)%>%
  mutate(diff_pred1=100*abs(fit.UJP.fitted-fit.mouth_width.fitted)/fit.mouth_width.fitted,
         diff_pred2=100*abs(fit.UJP.fitted-fit.inner_mouth_width.fitted)/fit.inner_mouth_width.fitted)%>%
  select(taxon,specimen,UJP,fit.UJP.fitted,fit.UJP.range,
         mouth_width,fit.mouth_width.fitted,fit.mouth_width.range,diff_pred1,
         inner_mouth_width,fit.inner_mouth_width.fitted,fit.inner_mouth_width.range,diff_pred2) %>%
  kable(digits=2,
        align=c("l",rep("c",11)),
        col.names=c("Taxon","Specimen",
                    "UJP","Est.","95% C.I.",
                    "Mouth Width","Est.","95% C.I.","% Dif.",
                    "Inner Mouth Width","Est.","95% C.I.","% Dif."),
        caption="Comparison of total length estimates using upper jaw perimeter and mouth width. Length estimates using both are relatively similar, suggesting mouth width is an appropriate approximation of upper jaw perimeter. All measurements in cm.")%>%
  column_spec(1, italic = T)%>%
  column_spec(c(4,7,11), bold = T)%>%
  add_header_above(c(" "=2,"Upper Jaw Perimeter"=3,"Mouth Width"=4,"Inner Mouth Width"=4))%>%
  kable_styling()%>%
  scroll_box(width = "100%")
```

Table 5.1: Comparison of total length estimates using upper jaw perimeter and mouth width. Length estimates using both are relatively similar, suggesting mouth width is an appropriate approximation of upper jaw perimeter. All measurements in cm.

|  | | Upper Jaw Perimeter | | | Mouth Width | | | | Inner Mouth Width | | | |
| --- | --- | --- | --- | --- | --- | --- | --- | --- | --- | --- | --- | --- |
| Taxon | Specimen | UJP | Est. | 95% C.I. | Mouth Width | Est. | 95% C.I. | % Dif. | Inner Mouth Width | Est. | 95% C.I. | % Dif. |
| Coccosteus cuspidatus | Recon. (M & W 1968) | 9.78 | 100.79 | (30.9–170.7) | 6.87 | 89.10 | (58.8–135) | 13.13 | 6.10 | 80.84 | (53.4–122.5) | 24.68 |
| Dunkleosteus terrelli | CMNH 7424 | 51.00 | 320.51 | (250.7–390.3) | 29.99 | 296.39 | (195.5–449.5) | 8.14 | 21.28 | 224.04 | (147.8–339.6) | 43.06 |
| Dunkleosteus terrelli | CMNH 6090 | 91.00 | 533.73 | (462.3–605.2) | 51.40 | 459.96 | (303.1–698.1) | 16.04 | 40.40 | 377.93 | (249.1–573.4) | 41.22 |
| Dunkleosteus terrelli | CMNH 7054 | 99.00 | 576.38 | (504.4–648.4) | 52.00 | 464.34 | (305.9–704.8) | 24.13 | 40.65 | 379.84 | (250.4–576.3) | 51.74 |
| Dunkleosteus terrelli | CMNH 5768 | 120.00 | 688.32 | (614.6–762) | 71.63 | 602.96 | (397–915.8) | 14.16 | 53.80 | 477.41 | (314.5–724.6) | 44.18 |

## 5.4 Testing accuracy of UJP and mouth width in estimating total length in sharks

```
fsbc_sharks%>%
  mutate(fit_ujp=predict(fit.UJP,.,interval="prediction")[,1],
         lwr_ujp=predict(fit.UJP,.,interval="prediction")[,2],
         upr_ujp=predict(fit.UJP,.,interval="prediction")[,3],
         PE_ujp=((fit_ujp-total_length)/fit_ujp)*100,
         fit_mouth=exp(predict(fit.mouthwidth,.,interval="prediction")[,1]),
         lwr_mouth=exp(predict(fit.mouthwidth,.,interval="prediction")[,2]),
         upr_mouth=exp(predict(fit.mouthwidth,.,interval="prediction")[,3]),
         PE_mouth=((fit_mouth-total_length)/fit_mouth)*100)%>%
  column_to_rownames("specimen")%>%
  select(taxon,total_length,fit_ujp,lwr_ujp,upr_ujp,PE_ujp,fit_mouth,lwr_mouth,upr_mouth,PE_mouth)%>%
  mutate(est_diff=100*abs(fit_ujp-fit_mouth)/fit_mouth)%>%
  kable(digits=1,
        align=c("l","c","c","c","c","c","c","c","c","c","c"),
        caption="Testing the accuracy of upper jaw perimeter and mouth width in estimating the total length of sharks of known length. Specimens of <i>Rhizoprionodon terranovae</i>, <i>Carcharhinus acronotus</i>, and the uncataloged specimen of <i>Galeocerdo cuvieri</i> were measured off of external measurements of fluid-preserved specimens, whereas measurements of <i>Carcharhinus leucas</i> and all other specimens of <i>Galeocerdo</i> were made from dried jaws. Total length in fluid-preserved specimens was measured as natural total length, whereas whether reported total length in the dried jaws represented natural or stretched total length could not be confirmed.",
        col.names = c("Taxon","Total Length","Est.","Lower","Upper","PE","Est.","Lower","Upper","PE","Percent Difference"))%>%
  column_spec(2, italic = T)%>%
  column_spec(c(3,4,8), bold = T)%>%
  add_header_above(c(" "=3,"Upper Jaw Perimeter"=4,"Mouth Width"=4," "=1))%>%
  kable_styling()
```

Table 5.2: Testing the accuracy of upper jaw perimeter and mouth width in estimating the total length of sharks of known length. Specimens of *Rhizoprionodon terranovae*, *Carcharhinus acronotus*, and the uncataloged specimen of *Galeocerdo cuvieri* were measured off of external measurements of fluid-preserved specimens, whereas measurements of *Carcharhinus leucas* and all other specimens of *Galeocerdo* were made from dried jaws. Total length in fluid-preserved specimens was measured as natural total length, whereas whether reported total length in the dried jaws represented natural or stretched total length could not be confirmed.

|  | | | Upper Jaw Perimeter | | | | Mouth Width | | | |  |
| --- | --- | --- | --- | --- | --- | --- | --- | --- | --- | --- | --- |
|  | Taxon | Total Length | Est. | Lower | Upper | PE | Est. | Lower | Upper | PE | Percent Difference |
| FSBC 06415 | Galeocerdo cuvieri | 362.0 | 333.8 | 264.0 | 403.7 | -8.4 | 364.1 | 240.0 | 552.4 | 0.6 | 8.3 |
| FSBC 00844 | Carcharhinus leucas | 198.1 | 251.2 | 181.6 | 320.9 | 21.1 | 223.4 | 147.4 | 338.6 | 11.3 | 12.5 |
| FSBC 00884 | Carcharhinus leucas | 234.9 | 237.9 | 168.2 | 307.5 | 1.2 | 244.2 | 161.1 | 370.2 | 3.8 | 2.6 |
| FSBC 02127 | Galeocerdo cuvieri | 168.9 | 165.9 | 96.2 | 235.6 | -1.8 | 176.0 | 116.1 | 266.7 | 4.0 | 5.7 |
| FSBC 18083 | Carcharhinus leucas | 64.0 | 122.0 | 52.1 | 191.8 | 47.5 | 78.1 | 51.5 | 118.3 | 18.0 | 56.2 |

Based on this, it seems that `UJP` and `mouth_width` capture similar enough morphometric signal that `mouth_width` can be considered an appropriate substitute for testing whether `UJP` in sharks accurately predicts body size in arthrodires (especially given UJP is unavailable for smaller sharks). The two variables disagree in the length estimates of FSBC 18083, but this is likely because this specimen pertains to a very small (possibly neonatal) *Carcharhinus leucas* and is outside the range of values of the dataset of Lowry et al. (2009).

## 5.5 Using *Brachyplatystoma* as a Case Study

```
data_mouths%>%
  filter(genus=="Brachyplatystoma")%>%
  augment(newdata=.,fit.UJP)%>%
  rename(fit1.fitted=.fitted)%>%
  augment(newdata=.,fit.mouthwidth)%>%
  rename(fit2.fitted=.fitted)%>%
  mutate(fit2.fitted=exp(fit2.fitted)*regression.stats(fit.mouthwidth)$CF)%>%
  mutate(error1=100*((fit1.fitted-total_length)/total_length),
         error2=100*((fit2.fitted-total_length)/total_length))%>%
  select(taxon,specimen,total_length,fit1.fitted,error1,fit2.fitted,error2)%>%
  kable(digits=2,align=c("l","c","c","c","c","c","c"),
        col.names = c("Taxon","Specimen","Actual","Estimated","% Difference from Actual Value","Estimated","% Difference from Actual Value"),
        caption="Estimated total length in <i>Brachyplatystoma capapretum</i> using upper jaw perimeter. All measurements in cm.")%>%
  column_spec(1, italic = T)%>%
  add_header_above(c(" "=3,"UJP"=2,"Mouth Width"=2))%>%
  kable_styling()
```

Table 5.3: Estimated total length in *Brachyplatystoma capapretum* using upper jaw perimeter. All measurements in cm.

|  | | | UJP | | Mouth Width | |
| --- | --- | --- | --- | --- | --- | --- |
| Taxon | Specimen | Actual | Estimated | % Difference from Actual Value | Estimated | % Difference from Actual Value |
| Brachyplatystoma capapretum | MZUSP 78481 | 73 | 121.7 | 66.72 | 121.52 | 66.47 |

By using *Brachyplatystoma capapretum*, a large, nektonic fish with a superficially shark-like body shape, as a case study, it can be seen that UJP fails to accurately estimate length in this taxon, and in fact significantly over-estimates its body size (that is, *B. capapretum* has a much larger mouth than expected assuming shark-like proportions). This suggests that `UJP` or `mouth_width` may not exhibit a single allometric relationship with total length across all fishes, particularly non-sharks such as *Brachyplatystoma* (Siluriformes: Pimelodidae) or arthrodires.

# 6 Using mouth width to estimate total length in arthrodires

## 6.1 Mouth width versus total length in chondrichthyans (and arthrodires)

```
(mouth_width_plot<-data_mouths%>%
  drop_na(mouth_width)%>%
  augment(fit.mouthwidth,newdata=.,interval="prediction")%>%
  mutate(across(.fitted:.upper,~exp(.)*regression.stats(fit.mouthwidth)$CF),
         total_length=ifelse(genus=="Dunkleosteus",
                             predict(fit.UJP,.),total_length),
         clade2=case_when(genus=="Dunkleosteus"~"Dunkleosteus",
                          genus %in% c("Amazichthys","Newspecies")~"Est. From Skull",
                          clade=="Placodermi"~"Other Placodermi",
                          genus=="Alopias"~"Alopias",
                          genus=="Cladoselache"~"Cladoselache",
                          genus %in% c("Planonasus","Cephaloscyllium","Isistius")~
                                   "Planonasus + Cephaloscyllium",
                          clade=="Chondrichthyes"~"Other Chondrichthyes"))%>%
  filter(clade %in% c("Placodermi","Chondrichthyes"),
         !is.na(total_length)|genus=="Dunkleosteus")%>%
  ggplot(.,aes(total_length,mouth_width))+
  scale_x_continuous(trans="log10") + scale_y_continuous(trans="log10")+
  geom_star(aes(starshape=clade2,fill=clade2),size=2)+
  scale_starshape_manual(values=c(13,13,13,13,1,1,1),
                         labels=c("Other Chondrichthyes","*Alopias*","*Cladoselache*",
                             "*Plano./Cephalo./Isist.*",
                             "*Dunkleosteus* (Est.)","*Amazichthys*/CMNH Aspin.","Other Placodermi"),
                         breaks=c("Other Chondrichthyes","Alopias",
                                  "Cladoselache",
                                  "Planonasus + Cephaloscyllium",
                                  "Dunkleosteus","Est. From Skull","Other Placodermi"))+
  scale_fill_manual(values=c("gray","green","black","orange","yellow","magenta","black"),
                    breaks=c("Other Chondrichthyes","Alopias","Cladoselache",
                             "Planonasus + Cephaloscyllium",
                             "Dunkleosteus","Est. From Skull","Other Placodermi"),
                    na.value=NA,
                    labels=c("Other Chondrichthyes","*Alopias*","*Cladoselache*",
                             "*Plano./Cephalo./Isist.*",
                             "*Dunkleosteus* (Est.)","*Amazichthys*/CMNH Aspin.","Other Placodermi"))+
  geom_segment(data=.%>%filter(genus %in% c("Coccosteus","Plourdosteus","Incisoscutum")),
               aes(xend=total_length*0.8,yend=mouth_width*1.3))+
  geom_segment(data=.%>%filter(specimen=="CMNH 5768"),
               aes(xend=total_length,yend=mouth_width*0.4))+
  geom_star(data=.%>%filter(clade=="Placodermi"),
            aes(starshape=clade2,fill=clade2),size=3.5,show.legend=F)+
  geom_line(aes(x=.fitted), color = "#3366FF",size=1.25,show.legend=F)+
  geom_line(aes(x=.lower), color = "#3366FF", linetype = "dashed",show.legend=F)+
  geom_line(aes(x=.upper), color = "#3366FF", linetype = "dashed",show.legend=F)+
  geom_star(data=.%>%filter(clade=="Placodermi",clade2=="Est. From Skull"),size=3.5,
            color="white",aes(starshape=clade2,fill=clade2),starstroke=1,show.legend=F)+
   geom_star(data=.%>%filter(clade=="Placodermi",clade2=="Est. From Skull"),size=3,
            color="black",aes(starshape=clade2,fill=clade2),starstroke=0.5,show.legend=F)+
  geom_star(data=.%>%filter(clade=="Placodermi",clade2=="Other Placodermi"),size=3.5,
            starshape=1,color="white",fill="black",starstroke=0.5,show.legend=F)+
  geom_text(data=.%>%filter(genus %in% c("Coccosteus","Plourdosteus","Incisoscutum")),size=3.5,
           aes(x=0.9*total_length*ifelse(genus=="Coccosteus",.85,1),
               y=1.365*mouth_width*ifelse(genus=="Coccosteus",.95,1),
               label=genus),hjust=1,vjust=0,fontface=3)+
  geom_text(data=.%>%filter(specimen=="CMNH 5768"),size=3.5,
            aes(y=0.35*mouth_width,label=genus),hjust=0.5,vjust=0,fontface=3)+
  labs(x="Total Length (cm)",y="Mouth Width (cm)",starshape="Group",fill="Group")+
  theme_classic()+
  theme(legend.position=c(0.8,0.25),
        legend.text=element_markdown()))
```

Figure 6.1: Plot of log10 mouth width versus total length in fishes, focusing on the difference between sharks (filled rectangles) and arthrodires (stars). Black stars represents arthrodires with known total lengths and mouth widths, whereas the yellow stars represents *Dunkleosteus terrelli* with estimated lengths from Ferrón et al. (2017) compared with directly measured mouth widths of the same specimens. Magenta stars are specimens of *Amazichthys trinajsticae* and an undescribed aspinothoracid at the CMNH in which the mouth width cannot be measured, but can be approximated based on skull width. Orange squares represents the genera *Planonasus*, *Cephaloscyllium* and *Isistius*, which have unusually wide mouths among sharks. Green squares represents the thresher shark *Alopias*, which has a narrower mouth relative to total length because of its elongate caudal fin.

```
ggsave("Arthrodire Mouths Figure 3 (Mouth Width).pdf",mouth_width_plot,
       "pdf",width=180,height=135,units="mm")
```

Based on this, it is clear that in all the arthrodires for which body length is directly measurable that arthrodires have much wider mouths than sharks at similar body sizes, in most cases outside the 95% prediction interval for sharks. The only sharks that plot close to arthrodires are *Cephaloscyllium*, *Planonasus*, and a few deep-sea squaloids (e.g., *Isistius*) that have notably large mouths relative to body size among sharks. This means that mouth dimensions are not expected to accurately predict body size in arthrodires. The estimated lengths from Ferrón are within the 95% prediction interval for sharks (and inded predict a smaller-than-average mouth for *Dunkleosteus* compared to sharks), but because these lengths are estimated based on a regression model derived from sharks

The two stars which are closer to the regression line formed by sharks are *Amazichthys trinjasticae* and an undescribed arthrodire (possibly an aspinothoracid) in the collections of the Cleveland Museum of Natural History. However, neither of these two specimens preserves the mouth, in both cases the mouth width is extrapolated from skull roof width assuming a relationship between the two similar to other arthodires. This is not the ideal way of determining mouth size in these taxa, but it’s worth noting even doing this results in mouths that are on the edge of the upper bound of the 95% prediction interval in sharks.

## 6.2 Testing using inner mouth width in arthrodires

```
data_mouths%>%
  drop_na(mouth_width)%>%
  mutate(mouth_width=ifelse(clade=="Placodermi",inner_mouth_width,mouth_width))%>%
  augment(fit.mouthwidth,newdata=.,interval="prediction")%>%
  mutate(across(.fitted:.upper,~exp(.)*regression.stats(fit.mouthwidth)$CF),
         total_length=ifelse(genus=="Dunkleosteus",
                             predict(fit.UJP,.),total_length),
         clade2=case_when(genus=="Dunkleosteus"~"Dunkleosteus",
                          genus %in% c("Amazichthys","Newspecies")~"Est. From Skull",
                          clade=="Placodermi"~"Other Placodermi",
                          genus=="Alopias"~"Alopias",
                          genus=="Cladoselache"~"Cladoselache",
                          genus %in% c("Planonasus","Cephaloscyllium","Isistius")~
                            "Planonasus + Cephaloscyllium",
                          clade=="Chondrichthyes"~"Other Chondrichthyes"))%>%
  filter(clade %in% c("Placodermi","Chondrichthyes"),
         !is.na(total_length)|genus=="Dunkleosteus")%>%
  ggplot(.,aes(total_length,mouth_width))+
  scale_x_continuous(trans="log10") + scale_y_continuous(trans="log10")+
  geom_star(aes(starshape=clade2,fill=clade2),size=2)+
  scale_starshape_manual(values=c(13,13,13,13,1,1,1),
                         labels=c("Other Chondrichthyes","*Alopias*","*Cladoselache*",
                                  "*Plano./Cephalo./Isist.*",
                                  "*Dunkleosteus* (Est.)","*Amazichthys*/CMNH Aspin.","Other Placodermi"),
                         breaks=c("Other Chondrichthyes","Alopias",
                                  "Cladoselache",
                                  "Planonasus + Cephaloscyllium",
                                  "Dunkleosteus","Est. From Skull","Other Placodermi"))+
  scale_fill_manual(values=c("gray","green","black","orange","yellow","darkcyan","black"),
                    breaks=c("Other Chondrichthyes","Alopias","Cladoselache",
                             "Planonasus + Cephaloscyllium",
                             "Dunkleosteus","Est. From Skull","Other Placodermi"),
                    na.value=NA,
                    labels=c("Other Chondrichthyes","*Alopias*","*Cladoselache*",
                             "*Plano./Cephalo./Isist.*",
                             "*Dunkleosteus* (Est.)","*Amazichthys*/CMNH Aspin.","Other Placodermi"))+
  geom_segment(data=.%>%filter(genus %in% c("Coccosteus","Plourdosteus","Incisoscutum")),
               aes(xend=total_length*0.8,yend=mouth_width*1.3))+
  geom_segment(data=.%>%filter(specimen=="CMNH 5768"),
               aes(xend=total_length,yend=mouth_width*0.4))+
  geom_star(data=.%>%filter(clade=="Placodermi"),
            aes(starshape=clade2,fill=clade2),size=3.5,show.legend=F)+
  geom_line(aes(x=.fitted), color = "#3366FF",size=1.25,show.legend=F)+
  geom_line(aes(x=.lower), color = "#3366FF", linetype = "dashed",show.legend=F)+
  geom_line(aes(x=.upper), color = "#3366FF", linetype = "dashed",show.legend=F)+
  geom_star(data=.%>%filter(clade=="Placodermi",clade2=="Est. From Skull"),size=3.5,
            color="white",aes(starshape=clade2,fill=clade2),starstroke=0.5,show.legend=F)+
  geom_star(data=.%>%filter(clade=="Placodermi",clade2=="Other Placodermi"),size=3.5,
            starshape=1,color="white",fill="black",starstroke=0.5,show.legend=F)+
  geom_text(data=.%>%filter(genus %in% c("Coccosteus","Plourdosteus","Incisoscutum")),size=3.5,
            aes(x=0.9*total_length*ifelse(genus=="Coccosteus",.85,1),
               y=1.365*mouth_width*ifelse(genus=="Coccosteus",.95,1),
               label=genus),hjust=1,vjust=0,fontface=3)+
  geom_text(data=.%>%filter(specimen=="CMNH 5768"),size=3.5,
            aes(y=0.35*mouth_width,label=genus),hjust=0.5,vjust=0,fontface=3)+
  labs(x="Total Length (cm)",y="Mouth Width (cm)",starshape="Group",fill="Group")+
  theme_classic()+
  theme(legend.position=c(0.8,0.25),
        legend.text=element_markdown())
```

Figure 6.2: Plot of log10 mouth width versus total length in fishes, using `inner_mouth width` in arthrodires for which this value can be measured.

Inner mouth width could only be measured for *Coccosteus* among arthrodires known from complete remains. Nevertheless, the pattern is similar as for when maximum mouth width is considered: *Coccosteus cuspidatus* is found to have a much wider mouth than sharks of similar size, whereas *Dunkleosteus terrelli* is found to have a much narrower (=smaller) mouth than expected for an animal of its estimated length under the length estimates of Ferrón et al. (2017).

## 6.3 Mouth width versus total length in osteichthyans (and arthrodires)

```
data_mouths%>%
  drop_na(mouth_width)%>%
  augment(fit.mouthwidth,newdata=.,interval="prediction")%>%
  mutate(across(.fitted:.upper,~exp(.)*regression.stats(fit.mouthwidth)$CF),
         total_length=ifelse(genus=="Dunkleosteus",
                             predict(fit.UJP,.),total_length),
         clade2=case_when(order=="Siluriformes"~"Siluriformes",
                          family=="Serranidae"~"Serranidae",
                          clade=="Placodermi"~"Placodermi",
                          clade %in% c("Actinopterygii","Sarcopterygii")~"Other Osteichthyes"))%>%
  filter(!is.na(total_length)|genus=="Dunkleosteus")%>%
  arrange(clade2)%>%
  ggplot(.,aes(total_length,mouth_width))+
  scale_x_continuous(trans="log10") + scale_y_continuous(trans="log10")+
  geom_star(data=.%>%filter(clade=="Chondrichthyes"),starshape=13,
            fill="light gray",guide=F,show.legend=F,color="dark gray",size=2)+
  geom_star(data=.%>%filter(genus=="Dunkleosteus"),starshape=1,
            fill="light gray",guide=F,show.legend=F,color="dark gray",size=2)+
  scale_starshape_manual(values=c(15,15,15,1),
                         breaks=c("Siluriformes","Serranidae","Other Osteichthyes","Placodermi","Chondrichthyes"),
                         na.value=NA)+
  geom_line(aes(x=.fitted), color = "gray",size=1.25,show.legend=F)+
  geom_line(aes(x=.lower), color = "gray", linetype = "dashed",show.legend=F)+
  geom_line(aes(x=.upper), color = "gray", linetype = "dashed",show.legend=F)+
  geom_star(data=.%>%filter(clade!="Chondrichthyes",genus!="Dunkleosteus"),
            aes(starshape=clade2,fill=clade2),size=2)+
  geom_smooth(data=.%>%filter(genus!="Dunkleosteus",clade!="Chondrichthyes"),
              aes(color=clade2),formula=y~x,method="lm",se=F)+
  scale_color_manual(values=c(hue_pal()(3),NA),
                    breaks=c("Siluriformes","Serranidae","Other Osteichthyes","Placodermi"),
                    na.value=NA)+
  scale_fill_manual(values=c(hue_pal()(3),"black"),
                    breaks=c("Siluriformes","Serranidae","Other Osteichthyes","Placodermi"),
                    na.value=NA)+
  geom_segment(data=.%>%filter(genus %in% c("Coccosteus","Plourdosteus","Incisoscutum")),
               aes(xend=total_length*0.8,yend=mouth_width*1.3))+
  geom_star(data=.%>%filter(clade=="Placodermi",genus!="Dunkleosteus"),size=3.5,
            starshape=1,color="white",fill="black",starstroke=0.5)+
  geom_text(data=.%>%filter(genus %in% c("Coccosteus","Plourdosteus","Incisoscutum")),
            aes(x=0.9*total_length*ifelse(genus=="Coccosteus",.85,1),
                y=1.365*mouth_width*ifelse(genus=="Coccosteus",.95,1),
                label=genus),hjust=1,vjust=0,fontface=3)+
  labs(x="Total Length (cm)",y="Mouth Width (cm)",starshape="Group",fill="Group",color="Group")+
  theme_classic()+
  theme(legend.position=c(0.8,0.2))
```

Figure 6.3: Plot of log10 mouth width versus total length focusing on Actinopterygii, showing how Siluriformes have relatively wider mouths to body size similar to *Coccosteus*

By focusing on the relationship between mouth width and total length in all fishes (i.e., adding osteichthyans into consideration), it is clear that the relationship between mouth width and total length is not as consistent as is seen in sharks. In fact, arthrodires show mouth-body proportions that are very similar to extant predatory catfishes (Siluriformes).

## 6.4 Error in using mouth proportions in sharks to estimate length in complete arthrodires

```
fossil_taxa_mouths%>%
  filter(clade=="Placodermi")%>%
  drop_na(total_length,mouth_width)%>%
  augment(fit.mouthwidth,newdata=.)%>%
  mutate(.fitted=exp(.fitted)*regression.stats(fit.mouthwidth)$CF)%>%
  mutate(error=100*(total_length-.fitted)/.fitted)%>%
  mutate(taxon=ifelse(genus=="Newspecies","CMNH aspinothoracid",taxon))%>%
  add_row(taxon="All Species",error=mean(.$error))%>%
  select(taxon,specimen,total_length,.fitted,error)%>%
  kable(digits=c(1,1,1,1,1),col.names=c("Taxon","Specimen","Total Length","Est. Length","PE"),
        caption="Percent error when estimating total length in complete specimens of arthrodires using mouth width.",
        align=c("l","c","c","c","c"))%>%
  row_spec(9, bold = T)%>%
  kable_styling()
```

Table 6.1: Percent error when estimating total length in complete specimens of arthrodires using mouth width.

| Taxon | Specimen | Total Length | Est. Length | PE |
| --- | --- | --- | --- | --- |
| Amazichthys trinajsticae | AA.MEM.DS.8 | 89.7 | 131.3 | -31.7 |
| Coccosteus cuspidatus | Recon. (M & W 1968) | 39.4 | 89.1 | -55.8 |
| Holonema westolli | Recon. (Miles 1971) | 60.6 | 134.5 | -54.9 |
| Incisoscutum ritchei | Recon. (Trinjastic 2013) | 30.3 | 52.6 | -42.4 |
| Millerosteus minor | Composite Millerosteus | 14.9 | 31.1 | -51.9 |
| CMNH aspinothoracid | CMNH 50233 | 63.0 | 105.6 | -40.3 |
| Plourdosteus canadensis | MNHM 2-177 | 37.5 | 103.6 | -63.8 |
| Watsonosteus fletti | NMS G.1995.4.2 | 56.6 | 144.8 | -60.9 |
| All Species | NA | NA | NA | -50.2 |

## 6.5 Estimating mouth size in arthrodires like *Coccosteus* assuming similar proportions to sharks

```
fit.total_length_to_mouth_width<-
  lm(log(mouth_width)~log(total_length),data_mouths%>%
       filter(clade=="Chondrichthyes",
              !order %in% c("Chimaeriformes","Rajiformes"),
              genus!="Alopias"))
data_mouths%>%
  drop_na(mouth_width,total_length)%>%
  filter(clade=="Placodermi",
         !genus %in% c("Amazichthys","Newspecies"))%>%
  augment(newdata=.,fit.total_length_to_mouth_width)%>%
  mutate(.fitted=exp(.fitted)*regression.stats(fit.total_length_to_mouth_width)$CF,
         diff=100*(.fitted-mouth_width)/.fitted)%>%
  select(taxon,specimen,mouth_width,.fitted,diff)%>%
  arrange(mouth_width)%>%
  kable(digits=2,align=c("l","c","c","c","c"),
        col.names = c("Taxon","Specimen","Actual","Estimated","% Diff."),
        caption="Estimated mouth width in arthrodire taxa of known total length using a model based on extant sharks (excluding <i>Alopias</i>). Note that <i>Holonema</i> and <i>Watsonosteus</i> are based off of dorsoventrally crushed specimens but <i>Incisoscutum</i>, <i>Coccosteus</i>, and <i>Plourdosteus</i> are based off of three-dimensional specimens or reconstructions. All measurements in cm.")%>%
  add_header_above(c(" "=2,"Mouth Width"=3))%>%
  column_spec(1, italic = T)%>%
  kable_styling()
```

Table 6.2: Estimated mouth width in arthrodire taxa of known total length using a model based on extant sharks (excluding *Alopias*). Note that *Holonema* and *Watsonosteus* are based off of dorsoventrally crushed specimens but *Incisoscutum*, *Coccosteus*, and *Plourdosteus* are based off of three-dimensional specimens or reconstructions. All measurements in cm.

|  | | Mouth Width | | |
| --- | --- | --- | --- | --- |
| Taxon | Specimen | Actual | Estimated | % Diff. |
| Millerosteus minor | Composite Millerosteus | 1.89 | 1.00 | -88.25 |
| Incisoscutum ritchei | Recon. (Trinjastic 2013) | 3.60 | 2.20 | -63.27 |
| Coccosteus cuspidatus | Recon. (M & W 1968) | 6.87 | 2.96 | -132.29 |
| Plourdosteus canadensis | MNHM 2-177 | 8.27 | 2.80 | -195.34 |
| Holonema westolli | Recon. (Miles 1971) | 11.38 | 4.78 | -138.02 |
| Watsonosteus fletti | NMS G.1995.4.2 | 12.46 | 4.43 | -181.17 |

If reversing the consideration and trying to estimate mouth width in arthrodires based on known total length, arthrodires are predicted to have mouths less than half their actual width. This is particularly pronounced in *Coccosteus*, *Incisoscutum*, and *Plourdosteus*, where the mouths can be measured from 3D specimens or reconstructions and are not subject to dorsoventral crushing.

```
data_mouths%>%
  select(-mouth_width)%>%
  rename(mouth_width=inner_mouth_width)%>%
  drop_na(mouth_width,total_length)%>%
  augment(newdata=.,fit.total_length_to_mouth_width)%>%
  mutate(.fitted=exp(.fitted)*regression.stats(fit.total_length_to_mouth_width)$CF,
         diff=100*(.fitted-mouth_width)/.fitted)%>%
  select(taxon,specimen,mouth_width,.fitted,diff)%>%
  arrange(mouth_width)%>%
  kable(digits=2,align=c("l","c","c","c","c"),
        col.names = c("Taxon","Specimen","Actual","Estimated","% Diff."),
        caption="Estimated mouth width in arthrodire taxa of known total length using a model based on extant sharks and inner mouth width. All measurements in cm.")%>%
  add_header_above(c(" "=2,"Mouth Width"=3))%>%
  column_spec(1, italic = T)%>%
  kable_styling()
```

Table 6.3: Estimated mouth width in arthrodire taxa of known total length using a model based on extant sharks and inner mouth width. All measurements in cm.

|  | | Mouth Width | | |
| --- | --- | --- | --- | --- |
| Taxon | Specimen | Actual | Estimated | % Diff. |
| Coccosteus cuspidatus | Recon. (M & W 1968) | 6.10 | 2.96 | -106.19 |
| Plourdosteus canadensis | MNHM 2-177 | 7.82 | 2.80 | -179.27 |

# 7 Estimating the length of arthrodires using approximated UJP

```
fit.approx_UJP_old<-lm(log(total_length)~log(UJP),
                   data_mouths %>% drop_na(mouth_length) %>% filter(clade=="Chondrichthyes") %>%
                                          mutate(UJP=ramanujan.approx(mouth_length*2,mouth_width)/2))
fit.approx_UJP<-lm(log(total_length)~log(UJP),
                   data_mouths %>% drop_na(mouth_length) %>% filter(clade=="Chondrichthyes") %>%
                     filter(!genus %in% c("Cetorhinus","Rhincodon","Megachasma","Alopias"),
                            order %in% c("Lamniformes","Carcharhiniformes","Hexanchiiformes")) %>%
                     mutate(UJP=ramanujan.approx(mouth_length*2,mouth_width)/2))
```

**Summary of model including all taxa**

```
summary(fit.approx_UJP_old)
```

```
## 
## Call:
## lm(formula = log(total_length) ~ log(UJP), data = data_mouths %>% 
##     drop_na(mouth_length) %>% filter(clade == "Chondrichthyes") %>% 
##     mutate(UJP = ramanujan.approx(mouth_length * 2, mouth_width)/2))
## 
## Residuals:
##      Min       1Q   Median       3Q      Max 
## -0.80346 -0.16582 -0.04366  0.13669  0.83162 
## 
## Coefficients:
##             Estimate Std. Error t value Pr(>|t|)    
## (Intercept)  2.60612    0.03802   68.55   <2e-16 ***
## log(UJP)     0.78160    0.01490   52.47   <2e-16 ***
## ---
## Signif. codes:  0 '***' 0.001 '**' 0.01 '*' 0.05 '.' 0.1 ' ' 1
## 
## Residual standard error: 0.2626 on 446 degrees of freedom
##   (30 observations deleted due to missingness)
## Multiple R-squared:  0.8606, Adjusted R-squared:  0.8603 
## F-statistic:  2753 on 1 and 446 DF,  p-value: < 2.2e-16
```

**Summary of model including only Carcharhiniformes, Lamniformes, and Hexanchiiformes**

```
summary(fit.approx_UJP)
```

```
## 
## Call:
## lm(formula = log(total_length) ~ log(UJP), data = data_mouths %>% 
##     drop_na(mouth_length) %>% filter(clade == "Chondrichthyes") %>% 
##     filter(!genus %in% c("Cetorhinus", "Rhincodon", "Megachasma", 
##         "Alopias"), order %in% c("Lamniformes", "Carcharhiniformes", 
##         "Hexanchiiformes")) %>% mutate(UJP = ramanujan.approx(mouth_length * 
##     2, mouth_width)/2))
## 
## Residuals:
##      Min       1Q   Median       3Q      Max 
## -0.49370 -0.11888 -0.02922  0.09438  0.66242 
## 
## Coefficients:
##             Estimate Std. Error t value Pr(>|t|)    
## (Intercept)  2.44705    0.03519   69.54   <2e-16 ***
## log(UJP)     0.83849    0.01428   58.71   <2e-16 ***
## ---
## Signif. codes:  0 '***' 0.001 '**' 0.01 '*' 0.05 '.' 0.1 ' ' 1
## 
## Residual standard error: 0.1975 on 314 degrees of freedom
##   (22 observations deleted due to missingness)
## Multiple R-squared:  0.9165, Adjusted R-squared:  0.9162 
## F-statistic:  3447 on 1 and 314 DF,  p-value: < 2.2e-16
```

**Regression statistics**

```
rbind("All Taxa"=regression.stats(fit.approx_UJP),
      "Carcharhiniformes, Lamniformes, Hexanchiiformnes"=regression.stats(fit.approx_UJP))
```

```
par(mfrow=c(1,2))
plot(lm(total_length~UJP,
                   data_mouths %>% 
                     drop_na(mouth_length) %>% filter(clade=="Chondrichthyes") %>%
                     mutate(UJP=ramanujan.approx(mouth_length*2,mouth_width)/2)),
     3)
plot(fit.approx_UJP_old,3)
```

Figure 7.1: Scale versus location plot using estimated UJP and an untransformed model (**A**) and a log-transformed model (**B**).

```
data_mouths%>%
  drop_na(mouth_length,mouth_width) %>%
  filter(clade=="Chondrichthyes") %>%
  mutate(UJP=ramanujan.approx(mouth_length*2,mouth_width)/2,
         clade2=case_when(genus == "Alopias" ~ "Alopias",
           order == "Squaliformes" ~ "Squaliformes",
           order == "Orectolobiformes" ~ "Orectolobiformes",
           order == "Chimaeriformes" ~ "Chimaeriformes",
           order == "Rhinopristiformes" ~ "Rhinopristiformes",
           genus %in% c("Megachasma","Cetorhinus","Rhincodon") ~ "Filter-Feeding Sharks",
           TRUE ~ "Other Taxa"))%>%
  mutate(clade2=factor(clade2,ordered=T,levels=c("Alopias",
                                                 "Filter-Feeding Sharks",
                                                 "Chimaeriformes",
                                                 "Orectolobiformes",
                                                 "Rhinopristiformes",
                                                 "Squaliformes",
                                                 "Other Taxa")))%>%
  augment(fit.approx_UJP_old,newdata=.)%>%
  ggplot(aes(.fitted,.resid)) +
  geom_hline(yintercept=0,linetype="dotted",color="light grey") +
  geom_point(aes(fill=clade2,shape=clade2)) +
  geom_smooth(method="loess",formula=y~x,color="red",se=F,size=0.5) +
  scale_shape_manual(values=c(22,22,24,24,24,24,21))+
  scale_fill_manual(values=c(hue_pal()(6),"grey"))+
  theme_classic()+
  labs(x="Fitted Values",fill="Clade",shape="Clade",y="Residuals")+
  theme(legend.position="bottom")
```

Figure 7.2: Residuals versus fitted plot using estimated UJP and a dataset of all sharks

```
plot(fit.approx_UJP,1)
```

Figure 7.3: Residuals versus fitted plot of model only considering Carcharhiniformes, Lamniformes, and Hexanchiformes

## 7.1 Testing how closely estimated UJP matches actual UJP in FSBC Sharks

```
#A separate equation was called for using estimated jaw perimeter rather than actual, as otherwise R freaks out if you try to change an existing equation to use a new variable.
lowry_approx<-lm(total_length ~ perimeter_est,lowry_sharks %>% mutate(perimeter_est=UJP))

fsbc_sharks%>%
  mutate(perimeter_est=ramanujan.approx(mouth_length*2,mouth_width)/2)%>%
  mutate(fit_ujp=predict(fit.UJP,.,interval="prediction")[,1],
         lwr_ujp=predict(fit.UJP,.,interval="prediction")[,2],
         upr_ujp=predict(fit.UJP,.,interval="prediction")[,3],
         PI_ujp=paste0("(",round(lwr_ujp,1),"–",round(upr_ujp,1),")"),
         PE_ujp=((fit_ujp-total_length)/fit_ujp)*100,
         fit_mouth=exp(predict(fit.mouthwidth,.,interval="prediction")[,1]),
         lwr_mouth=exp(predict(fit.mouthwidth,.,interval="prediction")[,2]),
         upr_mouth=exp(predict(fit.mouthwidth,.,interval="prediction")[,3]),
         PI_mouth=paste0("(",round(lwr_mouth,1),"–",round(upr_mouth,1),")"),
         PE_mouth=((fit_mouth-total_length)/fit_mouth)*100,
         fit_approx=predict(lm(total_length ~ perimeter_est,lowry_sharks %>% mutate(perimeter_est=UJP)),
                            .,interval="prediction")[,1],
         lwr_approx=predict(lm(total_length ~ perimeter_est,lowry_sharks %>% mutate(perimeter_est=UJP)),
                            .,interval="prediction")[,2],
         upr_approx=predict(lm(total_length ~ perimeter_est,lowry_sharks %>% mutate(perimeter_est=UJP)),
                            .,interval="prediction")[,3],
         PI_approx=paste0("(",round(lwr_approx,1),"–",round(upr_approx,1),")"),
         PE_approx=((fit_approx-total_length)/fit_approx)*100)%>%
  column_to_rownames("specimen")%>%
  mutate(est_diff_mouth=100*abs(fit_ujp-fit_mouth)/fit_mouth,
         est_diff_approx=100*abs(fit_ujp-fit_approx)/fit_approx)%>%
  select(taxon,total_length,
         UJP,fit_ujp,PI_ujp,PE_ujp,
         mouth_width,fit_mouth,PI_mouth,PE_mouth,est_diff_mouth,
         perimeter_est,fit_approx,PI_approx,PE_approx,est_diff_approx)%>%
  kable(digits=1,
        align=c("l","c","c","c","c","c","c","c","c","c","c","c","c","c","c"),
        caption="Testing how closely Ramanujan's approximation of elliptical perimeter. approximates UJP in dried jaws of sharks. PE is error relative to measured total length and percent difference is difference relative to UJP-derived estimate. All lengths in cm.",
        col.names = c("Taxon","Total Length",
                      "Value","Est.","95% P.I.","PE",
                      "Value","Est.","95% P.I.","PE","Percent Difference",
                      "Value","Est.","95% P.I.","PE","Percent Difference"))%>%
  column_spec(2, italic = T)%>%
  column_spec(c(4,13), bold = T)%>%
  add_header_above(c(" "=3,"Upper Jaw Perimeter"=4,"Mouth Width"=5,"Approximated UJP"=5))%>%
  kable_styling() %>% 
  scroll_box(width = "100%")
```

Table 7.1: Testing how closely Ramanujan’s approximation of elliptical perimeter. approximates UJP in dried jaws of sharks. PE is error relative to measured total length and percent difference is difference relative to UJP-derived estimate. All lengths in cm.

|  | | | Upper Jaw Perimeter | | | | Mouth Width | | | | | Approximated UJP | | | | |
| --- | --- | --- | --- | --- | --- | --- | --- | --- | --- | --- | --- | --- | --- | --- | --- | --- |
|  | Taxon | Total Length | Value | Est. | 95% P.I. | PE | Value | Est. | 95% P.I. | PE | Percent Difference | Value | Est. | 95% P.I. | PE | Percent Difference |
| FSBC 06415 | Galeocerdo cuvieri | 362.0 | 53.5 | 333.8 | (264–403.7) | -8.4 | 39.5 | 364.1 | (240–552.4) | 0.6 | 8.3 | 60.1 | 368.9 | (298.8–438.9) | 1.9 | 9.5 |
| FSBC 00844 | Carcharhinus leucas | 198.1 | 38.0 | 251.2 | (181.6–320.9) | 21.1 | 21.7 | 223.4 | (147.4–338.6) | 11.3 | 12.5 | 36.6 | 244.0 | (174.4–313.7) | 18.8 | 3.0 |
| FSBC 00884 | Carcharhinus leucas | 234.9 | 35.5 | 237.9 | (168.2–307.5) | 1.2 | 24.2 | 244.2 | (161.1–370.2) | 3.8 | 2.6 | 37.5 | 248.8 | (179.1–318.4) | 5.6 | 4.4 |
| FSBC 02127 | Galeocerdo cuvieri | 168.9 | 22.0 | 165.9 | (96.2–235.6) | -1.8 | 16.2 | 176.0 | (116.1–266.7) | 4.0 | 5.7 | 23.7 | 175.0 | (105.3–244.7) | 3.5 | 5.2 |
| FSBC 18083 | Carcharhinus leucas | 64.0 | 13.8 | 122.0 | (52.1–191.8) | 47.5 | 6.0 | 78.1 | (51.5–118.3) | 18.0 | 56.2 | 14.0 | 123.1 | (53.3–193) | 48.0 | 1.0 |

## 7.2 Comparing estimated UJP to actual UJP in arthrodires

```
data_mouths %>%
  filter(clade=="Placodermi") %>%
  drop_na(UJP) %>%
  mutate(UJP2=ramanujan.approx(mouth_length,mouth_width)/2,
         UJP3=c(NA,116,55,84,100),
         ratio=UJP2/UJP) %>%
  select(taxon,specimen, UJP, UJP3, UJP2, ratio) %>%
  kable(digits=2,align=c("l","c","c","c","c","c"),
        col.names=c("Taxon","Specimen","Actual UJP (Ferrón)","Actual UJP (Measured by Author)","Estimated UJP (Ramanujan Approximation)","Ratio"),
        caption="Comparison of estimated UJP to directly measured UJP in arthrodires. Ratio is the ratio between the approximated UJP and the measurements reported in Ferrón et al. (2017)") %>%
  column_spec(1,italic = T) %>%
  kable_styling()
```

Table 7.2: Comparison of estimated UJP to directly measured UJP in arthrodires. Ratio is the ratio between the approximated UJP and the measurements reported in Ferrón et al. (2017)

| Taxon | Specimen | Actual UJP (Ferrón) | Actual UJP (Measured by Author) | Estimated UJP (Ramanujan Approximation) | Ratio |
| --- | --- | --- | --- | --- | --- |
| Coccosteus cuspidatus | Recon. (M & W 1968) | 9.78 | NA | 7.17 | 0.73 |
| Dunkleosteus terrelli | CMNH 5768 | 120.00 | 116 | 88.85 | 0.74 |
| Dunkleosteus terrelli | CMNH 7424 | 51.00 | 55 | 38.30 | 0.75 |
| Dunkleosteus terrelli | CMNH 6090 | 91.00 | 84 | 64.89 | 0.71 |
| Dunkleosteus terrelli | CMNH 7054 | 99.00 | 100 | 67.67 | 0.68 |

Ramanujan’s approximation does not accurately estimate UJP in arthrodires, in contrast to sharks. This is likely because the skulls and mouths of arthrodires are blockier and more rectangular compared to the jaw cartilages of sharks, and thus their UJP cannot be approximated as an idealized ellipse. The ratio of actual UJP to estimated UJP is similar in both *Dunkleosteus* and *Coccosteus*, suggesting that UJP has been measured/estimated correctly and the difference is due to actual differences in the skull shape of arthrodires.

Measurements of UJP on specimens taken by the present author on specimens of *Dunkleosteus* are similar to the values reported in Ferrón et al. (2017), suggesting the smaller UJPs predicted by Ramanujan’s approximation cannot be attributed to measurement error. Differences between Ferrón et al. (2017)’s measurements and the author’s are probably due to measurement error from the difficulties in consistently placing a tape measurer along the UJP of mounted specimens, especially as UJP is not a perimeter along a well-defined edge but a detour must be made to encompass the upper supragnathals. One of the staff members at the Cleveland Museum of Natural History (A. McGee), was involved in the collection of UJP for both studies, and discussions with A. McGee suggest the two measurements were fairly close.

## 7.3 Length estimates in arthrodires using estimated UJP

```
data_mouths %>% filter(clade=="Placodermi") %>% drop_na(UJP) %>%
  arrange(mouth_width) %>%
  augment(fit.approx_UJP_old,newdata=.,interval="predict") %>%
  mutate(across(.fitted:.upper,~exp(.)*regression.stats(fit.approx_UJP_old)$CF)) %>%
  rename_at(vars(starts_with('.')), funs(paste0("fit1",.)))%>%
  augment(fit.approx_UJP,newdata=.,interval="predict") %>%
  rename_at(vars(starts_with('.')), funs(paste0("fit2",.)))%>%
  mutate(across(fit2.fitted:fit2.upper,~exp(.)*regression.stats(fit.approx_UJP)$CF),
         fit_ferron=predict(fit.UJP,.),
         fit1.range=paste0("(",round(fit1.lower,1),"–",round(fit1.upper,1),")"),
         fit2.range=paste0("(",round(fit2.lower,1),"–",round(fit2.upper,1),")"))%>%
  mutate(PE1=100*(fit1.fitted-fit_ferron)/fit1.fitted,
         PE2=100*(fit2.fitted-fit_ferron)/fit2.fitted) %>%
  select(taxon,specimen,
         fit1.fitted,fit1.range,PE1,
         fit2.fitted,fit2.range,PE2) %>%
  kable(caption="Estimated length of arthrodires using UJP and estimated UJP from a broader sample of sharks",
        digits=1,
        align=c("l","c","c","c","c","c","c","c"),
        col.names = c("Taxon","Specimen",
                      "Est.","95% P.I.","PE",
                      "Est.","95% P.I.","PE"))%>%
  add_header_above(c(" "=2,"Using All Sharks"=3,"Predatory Carcharhiniformes, Lamniformes, and Hexanchiiformes Only"=3))%>%
  column_spec(1, italic = T)%>%
  kable_styling()
```

Table 7.3: Estimated length of arthrodires using UJP and estimated UJP from a broader sample of sharks

|  | | Using All Sharks | | | Predatory Carcharhiniformes, Lamniformes, and Hexanchiiformes Only | | |
| --- | --- | --- | --- | --- | --- | --- | --- |
| Taxon | Specimen | Est. | 95% P.I. | PE | Est. | 95% P.I. | PE |
| Coccosteus cuspidatus | Recon. (M & W 1968) | 83.1 | (49.6–139.3) | -21.3 | 79.8 | (54–117.7) | -26.4 |
| Dunkleosteus terrelli | CMNH 7424 | 302.1 | (179.9–507.4) | -6.1 | 318.5 | (215.3–471.3) | -0.6 |
| Dunkleosteus terrelli | CMNH 6090 | 475.0 | (282.3–799.1) | -12.4 | 517.6 | (349.1–767.5) | -3.1 |
| Dunkleosteus terrelli | CMNH 7054 | 507.3 | (301.5–853.8) | -13.6 | 555.5 | (374.5–824) | -3.8 |
| Dunkleosteus terrelli | CMNH 5768 | 589.6 | (350.1–993) | -16.7 | 652.7 | (439.6–969.1) | -5.5 |

```
data_mouths%>%
  drop_na(mouth_length,mouth_width) %>%
  filter(genus=="Cladoselache") %>%
  mutate(UJP=ramanujan.approx(mouth_length*2,mouth_width)/2)%>%
  augment(fit.approx_UJP_old,newdata=.,interval="predict") %>%
  mutate(across(.fitted:.upper,~exp(.)*regression.stats(fit.approx_UJP_old)$CF)) %>%
  rename_at(vars(starts_with('.')), funs(paste0("fit1",.)))%>%
  augment(fit.approx_UJP,newdata=.,interval="predict") %>%
  rename_at(vars(starts_with('.')), funs(paste0("fit2",.)))%>%
  mutate(across(fit2.fitted:fit2.upper,~exp(.)*regression.stats(fit.approx_UJP)$CF),
         fit_ferron=predict(fit.UJP,.),
         fit1.range=paste0("(",round(fit1.lower,1),"–",round(fit1.upper,1),")"),
         fit2.range=paste0("(",round(fit2.lower,1),"–",round(fit2.upper,1),")"))%>%
  mutate(PE1=100*(fit1.fitted-total_length)/fit1.fitted,
         PE2=100*(fit2.fitted-total_length)/fit2.fitted) %>%
  select(taxon,specimen,total_length,
         fit1.fitted,fit1.range,PE1,
         fit2.fitted,fit2.range,PE2) %>%
  kable(caption="Estimated length of *Cladoselache* using estimated UJP. PE represents error relative to measured total length",
        digits=c(1,1,1,1,1,2,1,1,2),
        align=c("l","c","c","c","c","c","c","c","c"),
        col.names = c("Taxon","Specimen","Total Length",
                      "Est.","95% P.I.","PE",
                      "Est.","95% P.I.","PE"))%>%
  add_header_above(c(" "=3,"Using All Sharks"=3,"Predatory Carcharhiniformes, Lamniformes, and Hexanchiiformes Only"=3))%>%
  column_spec(1, italic = T)%>%
  kable_styling()
```

Table 7.4: Estimated length of *Cladoselache* using estimated UJP. PE represents error relative to measured total length

|  | | | Using All Sharks | | | Predatory Carcharhiniformes, Lamniformes, and Hexanchiiformes Only | | |
| --- | --- | --- | --- | --- | --- | --- | --- | --- |
| Taxon | Specimen | Total Length | Est. | 95% P.I. | PE | Est. | 95% P.I. | PE |
| Cladoselache sp. | AMNH 240 | 60.5 | 92.6 | (55.2–155.1) | 34.62 | 89.5 | (60.7–132.1) | 32.41 |
| Cladoselache sp. | CMNH 5934 | 86.0 | 129.1 | (77–216.5) | 33.39 | 128.0 | (86.7–188.9) | 32.79 |
| Cladoselache sp. | CMNH 5047 | 154.0 | 183.2 | (109.2–307.3) | 15.93 | 186.2 | (126.1–275.1) | 17.31 |

## 7.4 Scatterplot of UJP versus total length

```
ggplot(data_mouths %>% 
         mutate(UJP=ramanujan.approx(mouth_length*2,mouth_width)/2,
                clade=ifelse(clade %in% c("Actinopterygii","Sarcopterygii"),"Osteichthyes",clade)) %>%
         drop_na(UJP,total_length) %>%
         group_by(clade),
       aes(total_length,UJP)) +
  geom_star(data=.%>%filter(clade=="Osteichthyes"),aes(starshape=clade,fill=clade,color=clade)) +
  geom_star(data=.%>%filter(clade=="Chondrichthyes"),aes(starshape=clade,fill=clade,color=clade)) +
  geom_segment(data=.%>%filter(genus=="Coccosteus"),
               aes(y=UJP,yend=UJP+4,x=total_length,xend=total_length-24),alpha=0.75)+
  geom_star(data=.%>%filter(clade=="Placodermi"),aes(starshape=clade,fill=clade,color=clade)) +
  geom_star(data=.%>%filter(clade=="Placodermi"),aes(starshape=clade,fill=clade,color=clade),size=2.5,show.legend=F) +
  scale_color_manual(values=c("black","dark grey","black"))+
  scale_fill_manual(values=c(hue_pal()(1),"light grey","white"))+
  scale_starshape_manual(values=c(13,15,1))+
  geom_text(data=. %>% filter(genus=="Coccosteus"),hjust=1,fontface=3,
            aes(label=genus,x=total_length-25,y=UJP+5))+
  scale_x_continuous(trans="log10") +
  scale_y_continuous(trans="log10") +
  labs(x="Total Length (cm)",y="Estimated Upper Jaw Perimeter (cm)",
       starshape="Clade",fill="Clade",color="Clade")+
  theme(legend.position = c(0.8,0.2))
```

Figure 7.4: Scatter plot of estimates UJP versus total length in fishes

## 7.5 Relative size of UJP in *Coccosteus*

```
data_mouths %>% 
  mutate(UJP=ifelse(is.na(UJP),
                    ramanujan.approx(mouth_length*2,mouth_width)/2,
                    UJP)) %>%
  drop_na(UJP) %>%
  group_by(taxon) %>%
  summarise(UJP=mean(UJP),clade=unique(clade),total_length=mean(total_length)) %>%
  mutate(`UJP/total_length`=UJP/total_length) %>%
  arrange(`UJP/total_length`) %>%
  filter(`UJP/total_length` < 0.275 & `UJP/total_length` > 0.225) %>%
  select(taxon,clade,`UJP/total_length`) %>%
  kable(col.names = c("Taxon","Clade","UJP/Total Length"),digits=3,
        caption="Proportional UJP in *Coccosteus* compared with taxa of similar estimated UJP")%>%
  column_spec(1,italic=T) %>%
  kable_styling()
```

Table 7.5: Proportional UJP in *Coccosteus* compared with taxa of similar estimated UJP

| Taxon | Clade | UJP/Total Length |
| --- | --- | --- |
| Epinephelus itajara | Actinopterygii | 0.226 |
| Lepomis cyanellus | Actinopterygii | 0.226 |
| Neogobius melanostomus | Actinopterygii | 0.231 |
| Amia calva | Actinopterygii | 0.232 |
| Lepisosteus oculatus | Actinopterygii | 0.233 |
| Etmopterus spinax | Chondrichthyes | 0.233 |
| Lutjanus cyanopterus | Actinopterygii | 0.233 |
| Heteropriacanthus cruentatus | Actinopterygii | 0.236 |
| Acanthocybium solandri | Actinopterygii | 0.236 |
| Thunnus atlanticus | Actinopterygii | 0.236 |
| Polyprion americanus | Actinopterygii | 0.238 |
| Sander vitreum | Actinopterygii | 0.238 |
| Sander canadense | Actinopterygii | 0.239 |
| Pomoxis annularis | Actinopterygii | 0.239 |
| Salvelinus namaycush | Actinopterygii | 0.241 |
| Esox masquinongy | Actinopterygii | 0.242 |
| Oncorhynchus mykiss | Actinopterygii | 0.244 |
| Oncorhynchus kisutch | Actinopterygii | 0.246 |
| Coccosteus cuspidatus | Placodermi | 0.248 |
| Beryx decadactylus | Actinopterygii | 0.249 |
| Micropterus dolomieu | Actinopterygii | 0.250 |
| Calamus bajonado | Actinopterygii | 0.250 |
| Megachasma pelagios | Chondrichthyes | 0.257 |
| Scombrops oculatus | Actinopterygii | 0.258 |
| Micropterus salmoides | Actinopterygii | 0.259 |
| Salvelinus fontinalis | Actinopterygii | 0.262 |
| Latimeria chalumnae | Sarcopterygii | 0.262 |
| Mycteroperca venenosa | Actinopterygii | 0.263 |
| Epinephelus morio | Actinopterygii | 0.263 |
| Oncorhynchus clarkii | Actinopterygii | 0.266 |
| Brotula barbata | Actinopterygii | 0.267 |
| Tylosurus crocodilus | Actinopterygii | 0.267 |
| Epinephelus drummondhayi | Actinopterygii | 0.268 |
| Hexanchus griseus | Chondrichthyes | 0.270 |
| Mycteroperca interstitialis | Actinopterygii | 0.271 |

# 8 Diagnostic plots for UJP and mouth width

## 8.1 UJP

```
par(mfrow=c(2,2))
plot(fit.UJP)
```

Figure 8.1: Diagnostic plots for model regressing total length against UJP

Based on this UJP shows some slight heteroskedasticity, and the residuals seem to be slightly non-normally distributed.

## 8.2 Mouth Width

```
par(mfrow=c(2,2))
plot(fit.mouthwidth)
```

Figure 8.2: Diagnostic plots for model regressing total length against mouth width

# 9 Plotting against head length

```
data_mouths<-data_mouths%>%
  mutate(total_length=ifelse(genus!="Dunkleosteus",
                             total_length,
                             predict(fit.UJP,.)))
```

```
ggplot(data_mouths %>%
         drop_na(head_length,total_length,shape)%>%
         filter(clade %in% c("Actinopterygii","Sarcopterygii",
                             "Placodermi","Chondrichthyes"))%>%
         mutate(percent_head_length=head_length/total_length),
       aes(total_length,head_length/total_length))+
  geom_star(aes(starshape=clade,fill=shape))+
  scale_starshape_manual(values=c(15,13,1,28))+
  geom_star(data=. %>% filter(clade=="Placodermi",genus!="Dunkleosteus"),
            starshape=1,size=3.5,color="white",fill="black",starstroke=0.5)+
  geom_hline(yintercept=0.17,linetype="dashed")+
  geom_star(data=. %>% filter(genus=="Dunkleosteus"),
            starshape=1,size=3.5,fill="yellow")+
  annotate(geom="text",x=5,y=0.16,label="Eel-Like Taxa")+
  annotate(geom="text",x=480,y=0.085,hjust=1,label="Dunkleosteus",fontface=3)+
  annotate(geom="text",x=975,y=0.06,hjust=1,label="Regalecus",fontface=3)+
  scale_fill_manual(values=c(hue_pal()(5),"gray"))+
  scale_x_continuous(trans="log10")+
  scale_y_continuous(labels=scales::percent_format(accuracy=1))+
  labs(x="Total Length (cm)",y="Percent Head Length",fill="Body Shape",starshape="Clade")+
  theme_classic()+
  guides(fill=guide_legend(nrow=2,override.aes = list(starshape=15)),
         starshape=guide_legend(nrow=2))+
  theme(legend.position="bottom",legend.box = "horizontal")
```

Figure 9.1: Plot of log10 head length against log10 total length in fishes, showing how the length estimates of Ferron et al. (2017) result in unusually small head proportions for *Dunkleosteus* (yellow stars) compared to other fishes. Fishes in this graph are color-coded by body shape categories. Dashed line represents the point at which taxa below this point typically exhibit extremely elongate trunk proportions (e.g., anguilliform taxa, *Cheirocentrus* spp., *Coryphaena* spp.), or else have extremely elongate caudal fins that contribute greatly to total length (e.g., *Alopias*, Chimaeriformes).

Plotting head length as a percent of total length results in *Dunkleosteus* (using the estimates of Ferrón et al. 2017) plotting far outside the morphospace formed by almost all living fishes. Notably, other arthrodires do not show head-body proportions similar to the estimated values for *Dunkleosteus* by Ferrón et al. (2017), instead they plot well within the range of variation of extant fusiform fishes. *Holonema* and *Amazichthys* plot among extant taxa with elongate trunks (e.g., *Coryphaena*, *Cheirocentrus*, some gempylids, etc.), but still do not show proportions as extreme as Ferrón et al. (2017) implies for *Dunkleosteus*.

## 9.1 Highlighting taxa with elongate trunks

```
dunkleosteus_percent_head<-ggplot(
  data_mouths %>%
    drop_na(head_length,total_length)%>%
    mutate(percent_head_length=head_length/total_length,
           elongate=ifelse(shape %in% c("anguilliform","macruriform")|
                             genus %in% c("Cheirocentrus","Coryphaena","Tetrapturus",
                                          "Polypterus","Alepisaurus","Gonorynchus",
                                          "Rhaphiodon",
                                          "Thyrsitoides","Gempylus","Thyrsites")|
                             family %in% "Belonidae",
                           "Elongate Postcranium","Not Elongate"))%>%
    arrange(elongate),
  aes(total_length,head_length/total_length))+
  geom_segment(data=.%>%filter(specimen=="CMNH 6090"),
               aes(xend=total_length*0.8,yend=0.9*head_length/total_length),color="black")+
  geom_segment(data=.%>%filter(genus=="Regalecus",total_length==367),
               aes(xend=1250,yend=percent_head_length-0.001))+
  geom_star(aes(starshape=clade,fill=elongate))+
  geom_star(data=.%>%filter(elongate=="Elongate Postcranium"),
            aes(starshape=clade,fill=elongate))+
  scale_starshape_manual(values=c(15,13,1,28,11))+
  geom_segment(aes(x=70.65,y=0.1554,xend=1250,yend=0.16),size=0.4)+
  geom_segment(aes(x=71.4,y=0.2803,xend=1250,yend=0.325),size=0.4)+
  geom_segment(aes(x=96.8,y=0.2278,xend=1250,yend=0.24),size=0.4)+
  geom_star(data=.%>%
              filter(genus=="Coryphaena"&total_length==70.65|
                     taxon=="Epinephelus_malabaricus"&total_length==71.4|
                     genus=="Ruvettus"&total_length==96.8),
            aes(starshape=clade,fill=elongate))+
  geom_star(data=.%>%filter(clade=="Placodermi"),starshape=1,size=4,
            color="white",fill="black")+
  geom_hline(yintercept=0.17,linetype="dashed")+
  geom_star(data=. %>% filter(genus=="Dunkleosteus"),
            starshape=1,size=4,fill="yellow",color="black")+
  annotate(geom="text",x=2,y=0.16,size=3,label="Eel-Like Taxa")+
  annotate(geom="text",x=480,y=0.08,hjust=1,label="Dunkleosteus",
           size=3,fontface=3)+
  annotate(geom="text",x=4350,y=0.035,hjust=1,label="Regalecus",
           size=3,fontface=3)+
  annotate(geom="text",x=4350,y=0.14,hjust=1,label="Coryphaena",
           size=3,fontface=3)+
  annotate(geom="text",x=4350,y=0.22,hjust=1,label="Ruvettus",
           size=3,fontface=3)+
  annotate(geom="text",x=4350,y=0.305,hjust=1,label="Epinephelus",
           size=3,fontface=3)+
  coord_cartesian(xlim=c(1,2900),ylim=c(0.04,0.39))+
  scale_x_continuous(trans="log10")+
  scale_y_continuous(labels=scales::percent_format(accuracy=1))+
  labs(x="Total Length (cm)",y="Percent Head Length",fill="Body Shape",starshape="Clade")+
  theme_cowplot()+
  guides(fill=guide_legend(nrow=2,override.aes = list(starshape=15)),
         starshape=guide_legend(ncol=2,override.aes = list(fill="light gray")))+
  theme(legend.position="bottom",
        legend.text=element_text(size=9),
        legend.title=element_text(size=10))

(dunkleosteus_percent_head_regalecus<-ggdraw(dunkleosteus_percent_head) +
  draw_image("Arthrodire Mouths Supplementary Figure 4 (Epinephelus).png",
             x=0.415,y=0.335,scale=0.15)+
  draw_image("Arthrodire Mouths Supplementary Figure 2 (Coryphaena).png",
             x=0.415,y=-.01,scale=0.15)+
  draw_image("Arthrodire Mouths Supplementary Figure 3 (Ruvettus).png",
             x=0.415,y=.155,scale=0.15)+
  draw_image("Arthrodire Mouths Supplementary Figure 1 (Regalecus).png",
             x=0.415,y=-.195,scale=0.15))
```

Figure 9.2: Plot of percent head length against total length in fishes. This graph shows how the estimates of Ferron et al. (2017) result in unusually small head proportions for *Dunkleosteus* compared to other fishes. Taxa below the dashed line typically exhibit highly elongate trunks (e.g., anguilliform taxa, *Cheirocentrus* spp., *Coryphaena* spp.), or else have extremely elongate caudal fins that contribute greatly to total length (e.g., *Alopias*, Chimaeriformes). *Regalecus* silhouette modified from image by John Norris Wood, *Coryphaena* silhouette from image by Richard Winterbottom, and *Ruvettus* and *Epinephelus* silhouettes modified from images in Randall (1997).

```
ggsave("Arthrodire Mouths Figure 5 (Percent Head Dunkleosteus).pdf",
       dunkleosteus_percent_head_regalecus,device="pdf",
       units="mm",width=165,height=130)
```

In fact, the estimates from Ferrón et al. (2017) result in *Dunkleosteus* plotting in a region of morphospace only occupied by highly elongate fish. Indeed, the results of Ferrón et al. (2017) suggest head-body proportions that are more extreme than the majority of most elongate-bodied fishes, even moreso than groups such as Anguilliformes. The only extant fishes which show proportions comparable to these inferred proportions are *Electrophorus* and *Regalecus*, making it highly unlikely that *Dunkleosteus* was as long as predicted by Ferrón et al. (2017) or many previous studies on *Dunkleosteus* (as even most smaller estimates in previous studies for *D. terrelli* would still result in this taxon plotting among Anguilliformes and similar taxa).

## 9.2 Using precaudal length

Precaudal length for *Dunkleosteus terrelli* was calculated by taking the length of the upper lobe of the caudal fin from Ferrón et al. (2017), multiplying by the cosine to calculate the anteroposterior length of the fin in natural position, and then subtracting that from the total length.

```
ggplot(data_mouths %>%
         drop_na(shape)%>%
         filter(total_length!=precaudal_length|genus=="Dunkleosteus")%>%
         mutate(percent_head_length=head_length/precaudal_length),
       aes(precaudal_length,head_length/precaudal_length))+
  geom_star(aes(starshape=clade,fill=shape))+
  scale_starshape_manual(values=c(15,13,1,28))+
  geom_star(data=.%>%filter(clade=="Placodermi",genus!="Dunkleosteus"),starshape=1,size=5.5,color="white",fill="white")+
  geom_star(data=.%>%filter(clade=="Placodermi",genus!="Dunkleosteus"),starshape=1,size=3.5,fill="black")+
  geom_hline(yintercept=0.21,linetype="dashed")+
  geom_star(data=.%>% filter(genus=="Dunkleosteus")%>%
              mutate(precaudal_length=ifelse(specimen=="CMNH 5768",
                                             total_length-cos(28*(pi/180))*170,
                                             precaudal_length),
                     precaudal_length=ifelse(specimen=="CMNH 7424",
                                             total_length-cos(22*(pi/180))*80,
                                             precaudal_length),
                     precaudal_length=ifelse(specimen=="CMNH 7054",
                                             total_length-cos(27*(pi/180))*142,
                                             precaudal_length),
                     precaudal_length=ifelse(specimen=="CMNH 6090",
                                             total_length-cos(26*(pi/180))*132,
                                             precaudal_length)),
            starshape=1,size=3.5,fill="yellow",color="black")+
  annotate(geom="text",x=680,y=0.155,hjust=1,label="Dunkleosteus",fontface=3)+
  scale_fill_manual(values=c(hue_pal()(5),"gray"))+
  scale_x_continuous(trans="log10")+
  scale_y_continuous(labels=scales::percent_format(accuracy=1))+
  labs(x="Precaudal Length (cm)",y="Percent Head Length",fill="Body Shape",starshape="Clade")+
  theme_classic()+
  guides(fill=guide_legend(nrow=2,override.aes = list(starshape=15)),
         starshape=guide_legend(nrow=2))+
  theme(legend.position="bottom",legend.box = "horizontal")
```

Figure 9.3: Graph of percent head length against precaudal length in fishes. This shows head-body proportions better in taxa with extremely elongate caudal fins like *Alopias* and *Stegostoma*, but does not allow for comparisons with anguilliform taxa that lack a distinct caudal region.

As can be seen here, this still results in *Dunkleosteus terrelli* plotting as a large outlier to almost all other fishes, particularly fusiform species.

## 9.3 Treating head length in arthrodires as equivalent to prebranchial length

```
ggplot(data_mouths%>%
         drop_na(shape)%>%
         mutate(prebranchial_length=ifelse(clade=="Placodermi",
                                           head_length,prebranchial_length))%>%
         filter(!is.na(prebranchial_length))%>%
         filter(clade!="Placodermi"|!is.na(total_length))%>%
         mutate(percent_prebranchial_length=prebranchial_length/total_length),
       aes(y=percent_prebranchial_length,x=total_length))+
  geom_star(aes(fill=shape,starshape=clade))+
  scale_starshape_manual(values=c(15,13,1,28))+
  scale_fill_manual(values=c(hue_pal()(5),"gray"))+
  geom_star(data=.%>%filter(clade=="Placodermi",genus!="Dunkleosteus")%>%
              mutate(percent_prebranchial_length=head_length/total_length),
            starshape=1,size=5.5,color="white",fill="white")+
  geom_star(data=.%>%filter(clade=="Placodermi",genus!="Dunkleosteus")%>%
              mutate(percent_prebranchial_length=head_length/total_length),
            starshape=1,size=3.5,fill="black")+
  geom_star(data=mouth_predictions%>%
              filter(genus=="Dunkleosteus")%>%
              mutate(total_length=fit.UJP.fitted,
                     percent_prebranchial_length=head_length/total_length),
            starshape=1,
            size=3,fill="yellow")+
  scale_x_continuous(trans='log10')+
  scale_y_continuous(labels=scales::percent_format(accuracy=1))+
  annotate(geom="text",x=680,y=0.085,hjust=1,label="Dunkleosteus",fontface=3)+
  labs(x="Total Length (cm)",y="Percent Prebranchial Length",fill="Body Shape",
       starshape="Clade")+
  theme_classic()+
  guides(fill=guide_legend(nrow=2,override.aes = list(starshape=15)),
         starshape=guide_legend(nrow=2))+
  theme(legend.position="bottom",legend.box = "horizontal")
```

Figure 9.4: Plot of percent prebranchial length against total length in fishes, treating head length in arthrodires as equivalent to prebranchial length

Even if treating head length in arthrodires as equivalent to pre-branchial length in other fishes, this still results in *Dunkleosteus* having a below-average prebranchial length for its size (plotting among elongate, anguilliform, and macruriform taxa), and does not resemble the patterns seen in other arthrodires, which have “prebranchial lengths” that plot much closer to fusiform fishes.

## 9.4 Highlighting groupers

```
ggplot(
  data_mouths %>%
    drop_na(head_length,total_length)%>%
    mutate(percent_head_length=head_length/total_length),
  aes(total_length,head_length/total_length))+
  geom_star(aes(starshape=clade),fill="light gray",color="dark grey")+
  geom_star(data=.%>%filter(family %in% c("Serranidae")),
            aes(starshape=clade,fill=family))+
  scale_starshape_manual(values=c(15,13,1,28,11),guide="none")+
  geom_hline(yintercept=0.17,linetype="dashed")+
  annotate(geom="text",x=2,y=0.16,label="Eel-Like Taxa")+
  scale_x_continuous(trans="log10")+
  scale_y_continuous(labels=scales::percent_format(accuracy=1))+
  labs(x="Total Length (cm)",y="Percent Head Length",fill="Clade")+
  guides(fill=guide_legend(nrow=2,override.aes = list(starshape=15)))+
  theme_classic()+
  theme(legend.position=c(0.15,0.1))
```

Figure 9.5: Plot of percent head length against log-transformed total length, showing how groupers (Serranidae) have much larger heads than other fishes. The one point below the threshhold for elongate bodies is *Pseudanthias fasciatus*, which has a very long caudal fin.

## 9.5 Using lengths of Engelman (2023)

This paper was originally intended to come out before Engelman (2023), in order to lay the groundwork for that study. However, due to the vagaries of publication, Engelman (2023) actually ended up coming out in print first. This study is still written treating the work of Engelman (2023) as if it never existed, but because that study is already out it is possible to make a useful cross-study comparison showing how the length estimates in Engelman (2023) result in *Dunkleosteus* position within the head-trunk proportions of fishes as a postscript.

```
wrapper <- function(x, ...) paste(strwrap(x, ...), collapse = "\n")
(engelman_head_length<-ggplot(
    data_mouths %>%
      drop_na(head_length,total_length)%>%
      mutate(percent_head_length=head_length/total_length,
             elongate=ifelse(shape %in% c("anguilliform","macruriform")|
                               genus %in% c("Cheirocentrus","Coryphaena","Tetrapturus",
                                            "Polypterus","Alepisaurus","Gonorynchus",
                                            "Rhaphiodon",
                                            "Thyrsitoides","Gempylus","Thyrsites")|
                               family %in% "Belonidae",
                             "Elongate Postcranium","Not Elongate"))%>%
      arrange(elongate),
    aes(total_length,head_length/total_length))+
    geom_star(aes(starshape=clade,fill=elongate),data=.%>%filter(genus!="Dunkleosteus"))+
    geom_star(data=.%>%filter(elongate=="Elongate Postcranium",genus!="Dunkleosteus"),
              aes(starshape=clade,fill=elongate))+
    scale_starshape_manual(values=c(15,13,1,28,11))+
    geom_star(data=.%>%filter(clade=="Placodermi",genus!="Dunkleosteus"),
              starshape=1,size=4,
              color="white",fill="black")+
    geom_hline(yintercept=0.17,linetype="longdash")+
      geom_segment(data=.%>%filter(specimen=="CMNH 5768") %>% mutate(total_length=340.7),
               aes(xend=1100,yend=head_length/total_length),color="black")+
    geom_segment(data=.%>%filter(specimen=="CMNH 5768"),
                 aes(xend=1100,yend=head_length/total_length),color="black")+
  geom_star(data=. %>% filter(genus=="Dunkleosteus") %>%
              mutate(total_length=case_when(specimen=="CMNH 5768"~340.7,
                                            specimen=="CMNH 7424"~188.9,
                                            specimen=="CMNH 6090"~283.3,
                                            specimen=="CMNH 7054"~295.5)) %>%
              mutate(percent_head_length=head_length/total_length),
            starshape=1,size=5,fill="white",color="white")+
  geom_segment(
    data=data_mouths %>% filter(genus=="Dunkleosteus") %>% drop_na(UJP) %>%
      mutate(new_length=case_when(specimen=="CMNH 5768"~340.7,
                                  specimen=="CMNH 7424"~188.9,
                                  specimen=="CMNH 6090"~283.3,
                                  specimen=="CMNH 7054"~295.5)) %>%
      mutate(percent_head_length1=head_length/total_length,
             percent_head_length2=head_length/new_length),
    aes(y=percent_head_length1,x=total_length,
        yend=percent_head_length2,xend=new_length),
    linetype="dashed")+
    geom_star(data=. %>% filter(genus=="Dunkleosteus"),
            starshape=1,size=4,fill="white",color="black")+
    geom_star(data=. %>% filter(genus=="Dunkleosteus"),
              starshape=1,size=4,alpha=0.5,fill="yellow",color="grey")+
    geom_star(data=. %>% filter(genus=="Dunkleosteus") %>%
                mutate(total_length=case_when(specimen=="CMNH 5768"~340.7,
                                              specimen=="CMNH 7424"~188.9,
                                              specimen=="CMNH 6090"~283.3,
                                              specimen=="CMNH 7054"~295.5)) %>%
                mutate(percent_head_length=head_length/total_length),
            starshape=1,size=4,fill="yellow",color="black")+
    annotate(geom="text",x=2,y=0.16,size=3,label="Eel-Like Taxa")+
    annotate(geom="text",x=1150,y=0.181,hjust=0,label="Engelman (2023)",
           size=3)+
    annotate(geom="text",x=1150,y=0.09,hjust=0,
             label=wrapper("Ferrón et al. (2017)",width = 14),size=3)+
    coord_cartesian(xlim=c(1,2900),ylim=c(0.04,0.39))+
    theme_classic()+
    scale_x_continuous(trans="log10")+
    scale_y_continuous(labels=scales::percent_format(accuracy=1))+
    labs(x="Total Length (cm)",y="Percent Head Length",fill="Body Shape",starshape="Clade")+
    guides(fill=guide_legend(nrow=2,override.aes = list(starshape=15)),
           starshape=guide_legend(ncol=2,override.aes = list(fill="light gray")))+
    theme(legend.position="bottom",
          legend.text=element_text(size=9),
          legend.title=element_text(size=10)))
```

Figure 9.6: Head-trunk proportions of *Dunkleosteus* in Engelman (2023) compared to those using estimates from Ferrón et al. (2017)

```
ggsave("Arthrodire Mouths Engelman Head Lengths.png",engelman_head_length,
       width=178,height=134,units="mm",device="png")
```

As can be seen, the new length estimates result in head-trunk proportions for *Dunkleosteus* within the range of typical fusiform osteichthyans and chondrichthyans as well as agreeing more closely with those of complete arthrodires, albeit lower than expected based on coccosteomorphs. *Amazichthys* still plots below the threshhold for taxa with elongate trunks, but then again specimens of *Amazichthys* show a body plan with an elongate trunk. The fact that *Dunkleosteus* is close to but perfectly aligns with neither coccosteomorphs nor *Amazichthys* is to be expected given it is in a separate clade from these groups (Dunkleosteoidea) and in several respects its cranial morphology is decidedly generalized (e.g., it lacks the overhanging neurocranium of coccosteomorphs).

However, the new length estimates just *barely* put Dunkleosteus terrelli within the range of head-body proportions expected for fishes without greatly elongated trunks. In other words, despite the extremely short, chunky body depicted in Engelman (2023), that body shape is almost the *longest* *Dunkleosteus* can be without violating a typical fish body plan.

Again, some fishes like *Coryphaena* have very elongate trunks and a somewhat fusiform shape, but *Dunkleosteus*’ preserved anatomy does not show any features indicative of a *Coryphaena*-like body plan. *Dunkleosteus* lacks the elongate ventral armor of *Amazichthys*, and almost every body measurement associated with this species produces lengths that agree with the shorter body plan (Engelman, 2023). Thus, a much longer body shape would be completely speculative, and actually have to disagree with many proportions strongly conserved across fishes.

# 10 Mouth length

```
(mouth_length_plot<-data_mouths%>%
  drop_na(clade)%>%
  mutate(percent_mouth_length=mouth_length/head_length)%>%
  mutate(clade=ifelse(genus=="Dunkleosteus","Dunkleosteus",clade),
         clade=ifelse(clade=="Placodermi"&genus!="Dunkleosteus","Other Placodermi",clade))%>%
  arrange(rev(clade))%>%
  ggplot(aes(head_length,percent_mouth_length))+
  geom_star(aes(starshape=clade,fill=clade))+
  scale_starshape_manual(values=c(15,13,1,1,28),
                         labels=c("Actinopterygii","Chondrichthyes","*Dunkleosteus*",
                    "Other Placodermi","Sarcopterygii"),
                    breaks=c("Actinopterygii","Chondrichthyes","Dunkleosteus",
                             "Other Placodermi","Sarcopterygii"))+
  scale_fill_manual(values=c(hue_pal()(3)[1],hue_pal()(3)[3],
                             "yellow","black",hue_pal()(3)[2]),
                    labels=c("Actinopterygii","Chondrichthyes","*Dunkleosteus*",
                    "Other Placodermi","Sarcopterygii"),
                    breaks=c("Actinopterygii","Chondrichthyes","Dunkleosteus",
                             "Other Placodermi","Sarcopterygii"))+
  geom_star(data=.%>%filter(clade=="Other Placodermi",genus!="Dunkleosteus"),size=4,
            starshape=1,color="white",fill="black",starstroke=0.5)+
  geom_star(data=.%>%filter(genus=="Dunkleosteus"),starshape=1,size=3.5,
            color="black",fill="yellow")+
  scale_x_continuous(trans="log10")+
  scale_y_continuous(labels=scales::percent_format(accuracy=1))+
  labs(starshape="Clade",fill="Clade",x="Head Length (cm)",
       y="Mouth Length (as percent of Head Length)")+
  theme_classic()+
  theme(legend.position=c(0.15,0.85),
        legend.text=element_markdown()))
```

Figure 10.1: Plot of percent mouth length against head length in fishes, showing how *Dunkleosteus terrelli* (yellow stars), especially adult individuals of *D. terrelli*, have much larger mouths than other arthrodires

```
ggsave("Arthrodire Mouths Figure 9 (Mouth Length).pdf",mouth_length_plot,
       width=165,height=125,units="mm",device="pdf")
```

Based on this, it is apparent that *Dunkleosteus* has a much larger mouth (relative to head size) than most other arthrodires, even compared to the closely related dunkleosteoid *Eastmanosteus calliaspis*. The only arthrodires which has mouth proportions similar to *Dunkleosteus* are the Frasnian *Hadrosteus rapax* and the coeval Cleveland Shale arthrodires *Heintzichthys gouldii* and *Bungartius perissus*. Even these taxa only have mouths similar in size to juvenile *D. terrelli*, the adults (which have proportionally larger mouths) have mouths much larger than any other arthrodire.

This result highlights two important points:
1. *Dunkleosteus* has a proportionally large mouth even among arthrodires (which show much larger mouths relative to length than sharks), which means that estimates based on mouth dimensions are even more likely to be overestimates.
2. *Dunkleosteus* has an extremely large mouth relative to other arthrodires and this is highly likely to have affected its paleobiology and paleoecology (i.e., taking larger prey than other arthrodires).

# 11 References

Branstetter S, and Stiles R. 1987. Age and growth estimates of the bull shark, *Carcharhinus leucas*, from the northern Gulf of Mexico. *Environmental Biology of Fishes* 20:169–181.

Cliff G. 1995. Sharks caught in the protective gill nets off KwaZulu-Natal, South Africa. 8. The Great hammerhead shark *Sphyrna mokarran* (Rüppell). *South African Journal of Marine Science* 15:105-114. DOI 10.2989/025776195784156331.

Dennis K, and Miles RS. 1981. A pachyosteomorph arthrodire from Gogo, Western Australia. *Zoological Journal of the Linnean Society* 73:213-258. DOI 10.1111/j.1096-3642.1981.tb01594.x.

Desmond AJ. 1974. On the coccosteid arthrodire *Millerosteus minor*. *Zoological Journal of the Linnean Society* 54:277-298. DOI 10.1111/j.1096-3642.1974.tb00804.x.

Engelman RK. 2023. A Devonian Fish Tale: A New Method of Body Length Estimation Suggests Much Smaller Sizes for *Dunkleosteus terrelli* (Placodermi: Arthrodira). *Diversity* 15:318. DOI 10.3390/d15030318.

Ferrón HG, Martinez-Perez C, and Botella H. 2017. Ecomorphological inferences in early vertebrates: reconstructing *Dunkleosteus terrelli* (Arthrodira, Placodermi) caudal fin from palaeoecological data. *PeerJ* 5:e4081. DOI 10.7717/peerj.4081.

Francis MP. 2006. Morphometric minefields—towards a measurement standard for chondrichthyan fishes. *Environmental Biology of Fishes* 77:407-421. DOI 10.1007/s10641-006-9109-1.

Gardiner BG, and Miles RS. 1994. Eubrachythoracid arthrodires from Gogo, Western Australia. *Zoological Journal of the Linnean Society* 112:443-477. DOI 10.1111/j.1096-3642.1994.tb00331.x.

Garrick JAF. 1967. Revision of sharks of genus *Isurus* with description of a new speces (Galeoidea, Lamnidae). *Proceedings of the United Stated National Museum* 118:663–690.

Gilmore RG. 1983. Observations on the Embryos of the Longfin Mako, *Isurus paucus*, and the Bigeye Thresher, *Alopias superciliosus*. *Copeia* 1983:375-382. DOI 10.2307/1444380.

Hyde JE, and Huwig K. 2004. Images from the Jesse Earl Hyde Collection, Case Western Reserve University (CWRU) Department of Geological Sciences. *Available at https://caslabs.case.edu/hyde-collection/* (accessed September 1 2022).

Lowry D, de Castro ALF, Mara K, Whitenack LB, Delius B, Burgess GH, and Motta P. 2009. Determining shark size from forensic analysis of bite damage. *Marine Biology* 156:2483-2492. DOI 10.1007/s00227-009-1273-3.

Miles RS, and Dennis K. 1979. A primitive eubrachythoracid arthrodire from Gogo, Western Australia. *Zoological Journal of the Linnean Society* 66:31-62. DOI 10.1111/j.1096-3642.1979.tb01900.x.

Miles RS, and Westoll TS. 1968. The Placoderm Fish *Coccosteus cuspidatus* Miller ex Agassiz from the Middle Old Red Sandstone of Scotland. Part I. Descriptive Morphology. *Transactions of the Royal Society of Edinburgh* 67:373-476. DOI 10.1017/S0080456800024078.

Miles RS, and White EI. 1971. The Holonematidae (placoderm fishes), a review based on new specimens of *Holonema* from the Upper Devonian of Western Australia. *Philosophical Transactions of the Royal Society of London B, Biological Sciences* 263:101-234. DOI 10.1098/rstb.1971.0111.

Piercy AN, Carlson JK, Sulikowski JA, and Burgess GH. 2007. Age and growth of the scalloped hammerhead shark, *Sphyrna lewini*, in the north-west Atlantic Ocean and Gulf of Mexico. *Marine and Freshwater Research* 58:34-40.

Pollack AG, Driggers WB, III, Hanisko DS, and Ingram GW, Jr. 2019. Distribution and Length Data for Blacktip Sharks Captured on the NOAA/NMFS/SEFSC/MSLABS Bottom Longline Survey in the Western North Atlantic Ocean. North Charleston: SEDAR. p 16.

Randall JE. 1997. Randall’s tank photos. Collection of 10,000 large-format photos (slides) of dead fishes. *Available at www.fishbase.org (see species pages for more details)* (accessed June 29 2022).

Trinajstic K. 1999. New anatomical information on *Holonema* (Placodermi) based on material from the Frasnian Gogo Formation and the Givetian-Frasnian Gneudna Formation, Western Australia. *Geodiversitas* 21:69-84.

Trinajstic K, Briggs DEG, and Long JA. 2022. The Gogo Formation Lagerstätte: a view of Australia’s first great barrier reef. *Journal of the Geological Society* 179:jgs2021-2105. DOI 10.1144/jgs2021-105.

Trinajstic K, Sanchez S, Dupret V, Tafforeau P, Long J, Young G, Senden T, Boisvert C, Power N, and Ahlberg PE. 2013. Fossil musculature of the most primitive jawed vertebrates. *Science* 341:160-164. DOI 10.1126/science.1237275.

Vézina D. 1988. *Plourdosteus canadensis* (Woodward 1892), un Arthrodire du Frasnien inférieur du Canada: contribution à l’étude morphologique et phylogénétique des Plourdosteidae (Vertebrata, Placodermi) du Dévonien moyen et supérieur. PhD. l’Université de Paris VII.

Vézina D. 1990. Les Plourdosteidae fam. nov. (Placodermi, Arthrodira) et leurs relations phylétiques au sein des Brachythoraci. *Canadian Journal of Earth Sciences* 27:677-683. DOI 10.1139/e90-065.

Whitney NM, and Crow GL. 2007. Reproductive biology of the tiger shark (*Galeocerdo cuvier*) in Hawaii. Marine Biology 151:63-70. DOI 10.1007/s00227-006-0476-0.

# 12 Session Information

```
xfun::session_info()
```

```
## R version 4.2.1 (2022-06-23 ucrt)
## Platform: x86_64-w64-mingw32/x64 (64-bit)
## Running under: Windows 10 x64 (build 22621)
## 
## Locale:
##   LC_COLLATE=English_United States.utf8 
##   LC_CTYPE=English_United States.utf8   
##   LC_MONETARY=English_United States.utf8
##   LC_NUMERIC=C                          
##   LC_TIME=English_United States.utf8    
## 
## Package version:
##   askpass_1.1           assertthat_0.2.1      backports_1.4.1      
##   base64enc_0.1.3       bit_4.0.4             bit64_4.0.5          
##   blob_1.2.3            bookdown_0.30         broom_1.0.1          
##   bslib_0.4.1           cachem_1.0.6          callr_3.7.3          
##   cellranger_1.1.0      cli_3.4.1             clipr_0.8.0          
##   colorspace_2.0-3      commonmark_1.8.1      compiler_4.2.1       
##   cowplot_1.1.1         cpp11_0.4.3           crayon_1.5.2         
##   curl_4.3.3            data.table_1.14.4     DBI_1.1.3            
##   dbplyr_2.2.1          digest_0.6.30         dplyr_1.0.10         
##   dtplyr_1.2.2          ellipsis_0.3.2        evaluate_0.18        
##   fansi_1.0.3           farver_2.1.1          fastmap_1.1.0        
##   forcats_0.5.2         fs_1.5.2              gargle_1.2.1         
##   generics_0.1.3        ggplot2_3.4.0         ggstar_1.0.4         
##   ggtext_0.1.2          glue_1.6.2            googledrive_2.0.0    
##   googlesheets4_1.0.1   graphics_4.2.1        grDevices_4.2.1      
##   grid_4.2.1            gridExtra_2.3         gridtext_0.1.5       
##   gtable_0.3.1          haven_2.5.1           highr_0.9            
##   hms_1.1.2             htmltools_0.5.3       httr_1.4.4           
##   ids_1.0.1             isoband_0.2.7         jpeg_0.1.9           
##   jquerylib_0.1.4       jsonlite_1.8.3        kableExtra_1.3.4     
##   knitr_1.40            labeling_0.4.2        lattice_0.20-45      
##   lifecycle_1.0.3       lubridate_1.9.0       magick_2.7.3         
##   magrittr_2.0.3        markdown_1.3          MASS_7.3.58.1        
##   Matrix_1.5-1          memoise_2.0.1         methods_4.2.1        
##   mgcv_1.8-40           mime_0.12             modelr_0.1.10        
##   munsell_0.5.0         nlme_3.1-160          openssl_2.0.4        
##   pillar_1.8.1          pkgconfig_2.0.3       png_0.1.7            
##   prettyunits_1.1.1     processx_3.8.0        progress_1.2.2       
##   ps_1.7.2              purrr_0.3.5           R6_2.5.1             
##   ragg_1.2.4            rappdirs_0.3.3        RColorBrewer_1.1.3   
##   Rcpp_1.0.9            readr_2.1.3           readxl_1.4.1         
##   rematch_1.0.1         rematch2_2.1.2        reprex_2.0.2         
##   rlang_1.0.6           rmarkdown_2.18        rmdformats_1.0.4.9000
##   rstudioapi_0.14       rvest_1.0.3           sass_0.4.2           
##   scales_1.2.1          selectr_0.4.2         splines_4.2.1        
##   stats_4.2.1           stringi_1.7.8         stringr_1.4.1        
##   svglite_2.1.0         sys_3.4.1             systemfonts_1.0.4    
##   textshaping_0.3.6     tibble_3.1.8          tidyr_1.2.1          
##   tidyselect_1.2.0      tidyverse_1.3.2       timechange_0.1.1     
##   tinytex_0.42          tools_4.2.1           tzdb_0.3.0           
##   utf8_1.2.2            utils_4.2.1           uuid_1.1.0           
##   vctrs_0.5.0           viridisLite_0.4.1     vroom_1.6.0          
##   webshot_0.5.4         withr_2.5.0           xfun_0.34            
##   xml2_1.3.3            yaml_2.3.6
```

LS0tDQp0aXRsZTogIlN1cHBsZW1lbnRhcnkgSW5mb3JtYXRpb24gZm9yIg0Kc3VidGl0bGU6ICciR2lhbnQsIFN3aW1taW5nIE1vdXRoczogT3JhbCBEaW1lbnNpb25zIG9mIEV4dGFudCBTaGFya3MgRG8gTm90IEFjY3VyYXRlbHkgUHJlZGljdCBCb2R5IFNpemUgaW4gPGk+RHVua2xlb3N0ZXVzIHRlcnJlbGxpPC9pPiAoUGxhY29kZXJtaTogQXJ0aHJvZGlyYSkiJw0KYXV0aG9yOiAiUnVzc2VsbCBFbmdlbG1hbiINCmRhdGU6ICIxLzI5LzIwMjIiDQpvdXRwdXQ6DQogIHJtZGZvcm1hdHM6Omh0bWxfY2xlYW46DQogICAgaGlnaGxpZ2h0OiBoYWRkb2NrDQogICAgdG9jOiBUUlVFDQogICAgdG9jX2RlcHRoOiAzDQogICAgdG9jX2Zsb2F0OiBUUlVFDQogICAgbnVtYmVyX3NlY3Rpb25zOiBUUlVFDQogICAgdGh1bWJuYWlsczogRkFMU0UNCiAgICBjb2RlX2ZvbGRpbmc6IHNob3cNCiAgICBkZl9wcmludDogcGFnZWQNCiAgICBEVDogZGF0YXRhYmxlDQogICAgdXNlX2Jvb2tkb3duOiBUUlVFDQogICAgY29kZV9kb3dubG9hZDogdHJ1ZQ0KLS0tDQo8c3R5bGU+DQpib2R5IC5tYWluLWNvbnRhaW5lciB7DQogICAgICAgIG1heC13aWR0aDogMTAwMHB4Ow0KICAgIH0NCiN0b2Mgew0KICB3aWR0aDogMTUlOw0KfQ0KPC9zdHlsZT4NCg0KKipJbXBvcnRhbnQgTm90ZToqKiBJZiB0aGUgUiBjb2RlICgucm1kIGZpbGUpIGRvZXMgbm90IHdvcmsgd2hlbiB0cnlpbmcgdG8gcmVydW4gdGhlIGFuYWx5c2lzLCBvbmUgaXNzdWUgbWF5IGJlIHRoYXQgdGhlIGRhdGFzZXRzICh0aGUgLnhsc3ggYW5kL29yIC5jc3YgZmlsZXMpIGFuZCBhZGRpdGlvbmFsIGltYWdlcyAoaS5lLiwgdGhlIC5wbmcgZmlsZXMpIG11c3QgYmUgaW4gdGhlIHNhbWUgd29ya2luZyBlbnZpcm9ubWVudCAodXN1YWxseSB0aGUgc2FtZSBmb2xkZXIpIGFzIHRoZSAucm1kIGZpbGUuIElmIHBvc3NpYmxlLCBtYWtlIHN1cmUgdGhlIHN1cHBsZW1lbnRhcnkgZmlndXJlcyBhcmUgaW4gdGhlIHNhbWUgZm9sZGVyIGFzIHRoZSBSIENvZGUuIEluIHRoZSBldmVudCB0aGF0IHRoaXMgaXMgbm90IHBvc3NpYmxlLCBkZWFjdGl2YXRlIHRoZSBjaHVua3MgdGhhdCBjb250YWluIHJlZmVyZW5jZXMgdG8gc3VwcGxlbWVudGFyeSBmaWd1cmVzIG9yIGRlbGV0ZSB0aGVzZSBsaW5lcyBmcm9tIHRoZSBSIENvZGUuDQoNCmBgYHtyIHNldHVwLCBpbmNsdWRlPUZBTFNFfQ0Ka25pdHI6Om9wdHNfY2h1bmskc2V0KGVjaG8gPSBUUlVFLGZpZy53aWR0aD03LGZpZy5hc3A9MC43NSx3YXJuaW5nPUYsDQogICAgICAgICAgICAgICAgICAgICAgY2xhc3Muc291cmNlID0gJ2ZvbGQtaGlkZScpDQpsaWJyYXJ5KHJlYWR4bCk7bGlicmFyeShnZ3N0YXIpO2xpYnJhcnkoc2NhbGVzKTtsaWJyYXJ5KGthYmxlRXh0cmEpO2xpYnJhcnkobWFncml0dHIpOw0KbGlicmFyeShicm9vbSk7bGlicmFyeShnZ3RleHQpO2xpYnJhcnkobWFnaWNrKTtsaWJyYXJ5KGNvd3Bsb3QpDQpsaWJyYXJ5KHRpZHl2ZXJzZSkNCiMgRm9ybXVsYXMgZm9yIHJlZ3Jlc3Npb24gc3RhdGlzdGljcw0KcmVncmVzc2lvbi5zdGF0czwtZnVuY3Rpb24oZml0KXsNCiAgZm9ybXVsYTwtZml0JGNhbGw7DQogIGxvZ190cmFuczwtY2FzZV93aGVuKHN1YnN0cihjb2xuYW1lcyhmaXQkbW9kZWxbMV0pLDEsNSk9PSJsb2cxMCJ+ImxvZzEwIiwNCiAgICAgICAgICAgICAgICAgICAgICAgc3Vic3RyKGNvbG5hbWVzKGZpdCRtb2RlbFsxXSksMSw0KT09ImxvZygifiJsbiIsDQogICAgICAgICAgICAgICAgICAgICAgIFRSVUV+Im5vbmUiKTsNCiAgeTwtc3dpdGNoKGxvZ190cmFucywibG9nMTAiPTEwXmZpdCRtb2RlbFssMV0sImxuIj1leHAoZml0JG1vZGVsWywxXSksIm5vbmUiPWZpdCRtb2RlbFssMV0pOw0KICBmaXR0ZWQ8LXN3aXRjaChsb2dfdHJhbnMsImxvZzEwIj0xMF5maXR0ZWQoZml0KSwibG4iPWV4cChmaXR0ZWQoZml0KSksIm5vbmUiPWZpdHRlZChmaXQpKTsNCiAgYWJzZXJyb3I8LWFicyhmaXR0ZWQteSkvZml0dGVkOw0KICBRTUxFPC1pZmVsc2UobG9nX3RyYW5zPT0ibG9nMTAiLA0KICAgICAgICAgICAgICAgMTBeKChzaWdtYShmaXQpXjIpLzIpLA0KICAgICAgICAgICAgICAgZXhwKChzaWdtYShmaXQpXjIpLzIpKTsNCiAgc21lYXI8LWlmZWxzZShsb2dfdHJhbnM9PSJsb2cxMCIsDQogICAgICAgICAgICAgICAgc3VtKDEwXmZpdC50ZXN0JHJlc2lkdWFscykvbnJvdyhmaXQkbW9kZWwpLA0KICAgICAgICAgICAgICAgIHN1bShleHAoZml0JHJlc2lkdWFscykpL25yb3coZml0JG1vZGVsKSk7DQogIFJFPC1tZWFuKHkpL21lYW4oZml0dGVkKTsNCiAgQ0Y8LShSRStzbWVhcitRTUxFKS8zOw0KICBhZGpQRTwtbWVhbihhYnMoKGZpdHRlZCpDRikteSkvKGZpdHRlZCpDRikpOw0KICBTRUU8LWlmZWxzZShsb2dfdHJhbnM9PSJsb2cxMCIsDQogICAgICAgICAgICAgIDEwXigyK3NpZ21hKGZpdCkpLTEwMCwNCiAgICAgICAgICAgICAgZXhwKHNpZ21hKGZpdCkrNC42MDUyKS0xMDApOw0KICBzdW1tYXJ5PC1zdW1tYXJ5KGZpdCk7DQogIHN0YXRpc3RpY3M8LWRhdGEuZnJhbWUoIk4iPW5yb3coZml0JG1vZGVsKSwNCiAgICAgICAgICAgICAgICAgICAgICAgICAiZGYiPWZpdCRkZi5yZXNpZHVhbCwNCiAgICAgICAgICAgICAgICAgICAgICAgICAicjIiPXJvdW5kKHN1bW1hcnkoZml0KSRyLnNxdWFyZWQsNCksDQogICAgICAgICAgICAgICAgICAgICAgICAgImFkanIyIj1yb3VuZChzdW1tYXJ5KGZpdCkkYWRqLnIuc3F1YXJlZCw0KSwNCiAgICAgICAgICAgICAgICAgICAgICAgICAiQUlDIj1yb3VuZChBSUMoZml0KSwwKSwiQklDIj1yb3VuZChCSUMoZml0KSwwKSwNCiAgICAgICAgICAgICAgICAgICAgICAgICAibG9nTGlrIj1yb3VuZChsb2dMaWsoZml0KSwwKSwNCiAgICAgICAgICAgICAgICAgICAgICAgICAiUEUiPXJvdW5kKG1lYW4oYWJzZXJyb3IpKjEwMCwyKSxRTUxFPXJvdW5kKFFNTEUsMyksDQogICAgICAgICAgICAgICAgICAgICAgICAgc21lYXI9cm91bmQoc21lYXIsMyksUkU9cm91bmQoUkUsMyksQ0Y9cm91bmQoQ0YsMyksDQogICAgICAgICAgICAgICAgICAgICAgICAgImFkalBFIj1yb3VuZChtZWFuKGFkalBFKSoxMDAsMiksDQogICAgICAgICAgICAgICAgICAgICAgICAgIlNFRSI9cm91bmQoU0VFLDIpLA0KICAgICAgICAgICAgICAgICAgICAgICAgIHJvdy5uYW1lcz1kZXBhcnNlKHN1YnN0aXR1dGUoZml0KSkpOw0KICByZXR1cm4oc3RhdGlzdGljcykNCn0NCnJhbWFudWphbi5hcHByb3g8LWZ1bmN0aW9uKHgseSkgew0KICBtYWpvcmF4aXM9eC8yOw0KICBtaW5vcmF4aXM9eS8yOw0KICBwaSooKG1ham9yYXhpcyttaW5vcmF4aXMpLQ0KICAgICAgICAoKDMqKG1ham9yYXhpcy1taW5vcmF4aXMpXjIpLw0KICAgICAgICAgICAoMTAqKHgrbWlub3JheGlzKStzcXJ0KHheMisxNCptYWpvcmF4aXMqbWlub3JheGlzK21pbm9yYXhpc14yKSkpKQ0KfQ0KYGBgDQoNCiMgTG9hZGluZyBpbiBkYXRhDQoNCmBgYHtyfQ0KZGF0YV9tb3V0aHM8LXJlYWR4bDo6cmVhZF9leGNlbCgiQXJ0aHJvZGlyZSBNb3V0aHMgU3VwcGxlbWVudGFyeSBGaWxlIDEgKERhdGEpLnhsc3giKSAlPiUNCiAgZmlsdGVyKGlzLm5hKFNwZWNpbWVuKXxTcGVjaW1lbiE9IkZNTkggUEYgMTA5MCIpICU+JSAjVGhpcyBzcGVjaW1lbiByZW1vdmVkIGJlY2F1c2UgaXRzIHByb3BvcnRpb25zIGFyZSB1bnVzdWFsIGFuZCB0aGUgc3BlY2ltZW4gbWF5IG5vdCBiZSBjb21wbGV0ZQ0KICByZW5hbWUoZ2VudXM9R2VudXMsDQogICAgICAgICBzcGVjaWVzPVNwZWNpZXMsDQogICAgICAgICBjbGFkZT1DbGFkZSwNCiAgICAgICAgIG9yZGVyPU9yZGVyLA0KICAgICAgICAgZmFtaWx5PUZhbWlseSwNCiAgICAgICAgIHNoYXBlPVNoYXBlLA0KICAgICAgICAgc3BlY2ltZW49U3BlY2ltZW4sDQogICAgICAgICBvbnRvZ2VueT0iT250b2dlbmV0aWMgU3RhdHVzIiwNCiAgICAgICAgIGV4dGluY3Q9RXh0aW5jdCwNCiAgICAgICAgIHJlZmVyZW5jZT1SZWZlcmVuY2UsDQogICAgICAgICB0b3RhbF9sZW5ndGg9IlRvdGFsIExlbmd0aCAoY20pIiwNCiAgICAgICAgIHByZWNhdWRhbF9sZW5ndGg9IlByZWNhdWRhbCBMZW5ndGggKGNtKSIsDQogICAgICAgICBoZWFkX2xlbmd0aD0iSGVhZCBMZW5ndGggKGNtKSIsDQogICAgICAgICBwcmVicmFuY2hpYWxfbGVuZ3RoPSJQcmVicmFuY2hpYWwgTGVuZ3RoIChjbSkiLA0KICAgICAgICAgbW91dGhfbGVuZ3RoPSJNb3V0aCBMZW5ndGggKGNtKSIsDQogICAgICAgICBtb3V0aF93aWR0aD0iTW91dGggV2lkdGggKGNtKSIsDQogICAgICAgICBpbm5lcl9tb3V0aF93aWR0aD0iSW5uZXIgTW91dGggV2lkdGggKGNtKSIsDQogICAgICAgICBVSlA9IlVKUCAoY20pIiklPiUNCiAgbXV0YXRlKHNoYXBlPWZhY3RvcihzaGFwZSxvcmRlcmVkPVQsbGV2ZWxzPWMoIm1hY3J1cmlmb3JtIiwiYW5ndWlsbGlmb3JtIiwiZWxvbmdhdGUiLA0KICAgICAgICAgICAgICAgICAgICAgICAgICAgICAgICAgICAgICAgICAgICAgICAiZnVzaWZvcm0iLCJjb21wcmVzc2lmb3JtIiwiZmxhdHRlbmVkIikpLA0KICAgICAgICAgdGF4b24gPSBwYXN0ZTAoZ2VudXMsIiAiLHNwZWNpZXMpKQ0KYGBgDQoNCiMjIFNlcGFyYXRpbmcgb3V0IGZvc3NpbCB0YXhhDQoNCmBgYHtyfQ0KKGZvc3NpbF90YXhhX21vdXRoczwtZGF0YV9tb3V0aHMlPiUNCiAgZmlsdGVyKGV4dGluY3Q9PVQpJT4lDQogIG11dGF0ZShzcGVjaW1lbjI9cGFzdGUwKGdlbnVzLCJfIixzcGVjaW1lbikpJT4lDQogIGNvbHVtbl90b19yb3duYW1lcygic3BlY2ltZW4yIikpDQpgYGANCg0KIyMgTG9hZGluZyBzaGFyayBkYXRhIGZyb20gTG93cnkgZXQgYWwuICgyMDA5KSB1c2VkIGJ5IEZlcnJvbiBldCBhbC4gMjAxNw0KDQpgYGB7cn0NCmxvd3J5X3NoYXJrczwtcmVhZF9leGNlbCgiQXJ0aHJvZGlyZSBNb3V0aHMgU3VwcGxlbWVudGFyeSBGaWxlIDIgKExvd3J5IGV0IGFsLiBTaGFyayBEYXRhKS54bHN4IiklPiUNCiAgcmVuYW1lKFVKUD1gVUpQKGNtKWAsdG90YWxfbGVuZ3RoPWBUQkwoY20pYCxmb3JrX2xlbmd0aD1gRm9yayBMZW5ndGggKGNtKWAscHJlY2F1ZGFsX2xlbmd0aD1gUHJlY2F1ZGFsIExlbmd0aCAoY20pYCx0YXhvbj1TcGVjaWVzKSU+JQ0KICBtdXRhdGUoc3BlY2llczI9dGF4b24pJT4lDQogIHNlcGFyYXRlKHNwZWNpZXMyLGMoImdlbnVzIiwic3BlY2llcyIpKSU+JQ0KICBtdXRhdGUoZmFtaWx5PWNhc2Vfd2hlbihnZW51cyAlaW4lIGMoIkNhcmNoYXJvZG9uIiwiSXN1cnVzIikgfiAiTGFtbmlkYWUiLA0KICAgICAgICAgICAgICAgICAgICAgICAgICBnZW51cyAlaW4lIGMoIlNwaHlybmEiKSB+ICJTcGh5cm5pZGFlIiwNCiAgICAgICAgICAgICAgICAgICAgICAgICAgZ2VudXMgJWluJSBjKCJDYXJjaGFyaGludXMiLCJHYWxlb2NlcmRvIiwiTmVnYXByaW9uIikgfiAiQ2FyY2hhcmhpbmlkYWUiDQogICAgICAgICAgICAgICAgICAgICAgICAgICksDQogICAgICAgICBwZXJjZW50X2JvZHk9cHJlY2F1ZGFsX2xlbmd0aC90b3RhbF9sZW5ndGgpDQpgYGANCg0KIyMgTG9hZGluZyBpbiBqYXcgZGF0YSBmb3IgRlNCQyBzaGFya3MNCg0KYGBge3J9DQpmc2JjX3NoYXJrczwtcmVhZF9leGNlbCgiQXJ0aHJvZGlyZSBNb3V0aHMgU3VwcGxlbWVudGFyeSBGaWxlIDMgKEZTQkMgU2hhcmsgSmF3cykueGxzeCIpDQoNCiNBZGRpbmcgcm93cyBmb3IgRlNCQyBzaGFya3MgdG8gbWFpbiBkYXRhc2V0DQpkYXRhX21vdXRoczwtZGF0YV9tb3V0aHMlPiUNCiAgYWRkX3Jvdyhmc2JjX3NoYXJrcyU+JQ0KICAgICAgICAgICAgc2VsZWN0KHRheG9uLHNwZWNpbWVuLG1vdXRoX3dpZHRoLFVKUCx0b3RhbF9sZW5ndGgscHJlY2F1ZGFsX2xlbmd0aCklPiUNCiAgICAgICAgICAgIHNlcGFyYXRlKC4sdGF4b24saW50bz1jKCJnZW51cyIsInNwZWNpZXMiKSxzZXA9IiAiLHJlbW92ZT1GKSU+JQ0KICAgICAgICAgICAgbXV0YXRlKGNsYWRlPSJDaG9uZHJpY2h0aHllcyIsDQogICAgICAgICAgICAgICAgICAgcmVmZXJlbmNlPSJQcmVzZW50IFN0dWR5IiwNCiAgICAgICAgICAgICAgICAgICBvcmRlcj1jYXNlX3doZW4oZ2VudXMgJWluJSBjKCJIZXhhbmNodXMiLCJIZXB0cmFuY2hpYXMiKX4iSGV4YW5jaGlmb3JtZXMiLA0KICAgICAgICAgICAgICAgICAgICAgICAgICAgICAgICAgICBnZW51cyAlaW4lIGMoIkdhbGVvY2VyZG8iLCJDYXJjaGFyaGludXMiLA0KICAgICAgICAgICAgICAgICAgICAgICAgICAgICAgICAgICAgICAgICAgICAgICAgIkhlbWlwcmlzdGlzIil+IkNhcmNoYXJoaW5pZm9ybWVzIiwNCiAgICAgICAgICAgICAgICAgICAgICAgICAgICAgICAgICAgZ2VudXMgJWluJSBjKCJPZG9udGFzcGlzIil+IkxhbW5pZm9ybWVzIiksDQogICAgICAgICAgICAgICAgICAgZmFtaWx5PWNhc2Vfd2hlbihnZW51cyAlaW4lIGMoIkhleGFuY2h1cyIsIkhlcHRyYW5jaGlhcyIpfiJIZXhhbmNoaWlkYWUiLA0KICAgICAgICAgICAgICAgICAgICAgICAgICAgICAgICAgICBnZW51cyAlaW4lIGMoIkdhbGVvY2VyZG8iLCJDYXJjaGFyaGludXMiKX4iQ2FyY2hhcmhpbmlkYWUiLA0KICAgICAgICAgICAgICAgICAgICAgICAgICAgICAgICAgICBnZW51cyAlaW4lIGMoIkhlbWlwcmlzdGlzIil+IkhlbWlnYWxlaWRhZSIsDQogICAgICAgICAgICAgICAgICAgICAgICAgICAgICAgICAgIGdlbnVzICVpbiUgYygiT2RvbnRhc3BpcyIpfiJPZG9udGFzcGlkYWUiKSkpDQpgYGANCg0KIyBEYXRhDQoNCiMjIE1lYXN1cmVtZW50cw0KDQpNZWFzdXJlbWVudCB8IFZhcmlhYmxlIE5hbWUgfCBEZWZpbml0aW9uDQotLS0tLS0tLS0tLSB8IC0tLS0tLS0tLS0tLS0gfCAtLS0tLS0tLS0tLS0tLS0tLS0tLS0tLS0tLS0tLS0tLS0tLS0tLS0tDQpUb3RhbCBsZW5ndGggfCB0b3RhbF9sZW5ndGggfCBMZW5ndGggZnJvbSBhbnRlcmlvciB0aXAgb2Ygcm9zdHJ1bSB0byBwb3N0ZXJpb3IgdGlwIG9mIGNhdWRhbCBmaW4NClByZWNhdWRhbCBsZW5ndGggfCBwcmVjYXVkYWxfbGVuZ3RoIHwgTGVuZ3RoIGZyb20gYW50ZXJpb3IgdGlwIG9mIHJvc3RydW0gdG8gcG9zdGVyaW9yIHRpcCBvZiBjYXVkYWwgcGVkdW5jbGUNCkhlYWQgbGVuZ3RoIHwgaGVhZF9sZW5ndGggfCBMZW5ndGggZnJvbSBhbnRlcmlvciB0aXAgb2Ygcm9zdHJ1bSB0byBwb3N0ZXJpb3IgbWFyZ2luIG9mIGJyYW5jaGlhbCBjYXZpdHkgPGJyPiAqKkZvciBzaGFya3M6KiogZnJvbSBzbm91dCB0byBvcGVuaW5nIG9mIHRlcm1pbmFsIGdpbGwgYXJjaCA8YnI+ICoqRm9yIG9zdGVpY2h0aHlhbnM6KiogZnJvbSBzbm91dCB0byBwb3N0ZXJpb3IgZW5kIG9mIG9wZXJjdWx1bSA8YnI+ICoqRm9yIGFydGhyb2RpcmVzOioqIGZyb20gc25vdXQgdG8gY3JhbmlvLXRob3JhY2ljIGpvaW50DQpQcmVicmFuY2hpYWwgbGVuZ3RoIHwgcHJlYnJhbmNoaWFsX2xlbmd0aCB8IGRpc3RhbmNlIGZyb20gdGlwIG9mIHNub3V0IHRvIGFudGVyaW9yIGVuZCBvZiBicmFuY2hpYWwgc2tlbGV0b24gPGJyPiAqKkZvciBzaGFya3M6KiogdG8gb3BlbmluZyBmb3IgZmlyc3QgZ2lsbCBzbGl0IDxicj4gKipGb3Igb3N0ZWljaHRoeWFuczoqKiB0byBhbnRlcmlvciBlbmQgb2Ygb3BlcmN1bGFyIHNlcmllcyA8YnI+ICoqRm9yIGFydGhyb2RpcmVzOioqIG5vdCBkaXN0aW5ndWlzaGFibGUNClVwcGVyIGphdyBwZXJpbWV0ZXIgfCBVSlAgfCBQZXJpbWV0ZXIgb2YgdXBwZXIgamF3IG1lYXN1cmVkIGZyb20gYmlsYXRlcmFsIGFuZ2xlIG9mIGphd3MNCk1vdXRoIHdpZHRoIHwgbW91dGhfd2lkdGggfCBCaWxhdGVyYWwgd2lkdGggb2YgbW91dGggYWNyb3NzIGV4dGVybmFsIGFuZ2xlcyBvZiB0aGUgamF3DQpJbm5lciBtb3V0aCB3aWR0aCB8IGlubmVyX21vdXRoX3dpZHRoIHwgIEJpbGF0ZXJhbCB3aWR0aCBvZiBtb3V0aCBhY3Jvc3MgaW5uZXIgYW5nbGVzIG9mIHRoZSBqYXcNCk1vdXRoIGxlbmd0aCB8IG1vdXRoX2xlbmd0aCB8IExlbmd0aCBvZiBtb3V0aCBhbG9uZyBhbnRlcm9wb3N0ZXJpb3IgYXhpcyA8YnI+ICoqRm9yIHNoYXJrczoqKiBhbnRlcm9wb3N0ZXJpb3IgbGVuZ3RoIGZyb20gdGhlIG1hbmRpYnVsYXIgc3ltcGh5c2lzIHRvIHRoZSBhbmdsZSBvZiB0aGUgamF3cyA8YnI+ICoqRm9yIG9zdGVpY2h0aHlhbnM6KiogYW50ZXJvcG9zdGVyaW9yIGxlbmd0aCBvZiB1cHBlciBqYXcgPGJyPiAgKipGb3IgYXJ0aHJvZGlyZXM6KiogYW50ZXJvcG9zdGVyaW9yIGxlbmd0aCBmcm9tIHRpcCBvZiBzdXByYWduYXRoYWxzIHRvIHBvaW50IHdoZXJlIHN1cHJhb3JhbCBzZW5zb3J5IGxpbmUgbWVldHMgbWFyZ2luIG9mIGphdw0KDQoqKk5vdGU6KiogTW9zdCBvZiB0aGVzZSBkaWZmZXJlbmNlcyBiZXR3ZWVuIGRlZmluaXRpb25zIGluIHRheGEgYXJlIGR1ZSB0byB0aGUgcHJlc2VuY2Ugb2YgYW4gb3BlcmN1bHVtIChpbiBvc3RlaWNodGh5YW5zIGFuZCBjaGltYWVyb2lkcykgb3IgdGhlIGluY29ycG9yYXRpb24gb2YgdGhlIGNyYW5pdW0gYW5kIGdpbGwgY292ZXJzIGFzIHNpbmdsZSB1bml0IChpLmUuLCB0aGUgY2hlZWsgcGxhdGVzIG9mIGFydGhyb2RpcmVzKS4gVGh1cywgZGVzcGl0ZSB0aGUgZGlmZmVyaW5nIGRlZmluaXRpb25zIHRoZXNlIG1lYXN1cmVtZW50cyBhcmUgYW5hdG9taWNhbGx5IGhvbW9sb2dvdXMuDQoNCiMjIFRheG9ub21pYyBkaXN0cmlidXRpb24gb2Ygb2JzZXJ2YXRpb25zDQoNCmBgYHtyLGNsYXNzLnNvdXJjZSA9ICdmb2xkLWhpZGUnfQ0KZGF0YV9tb3V0aHMlPiUNCiAgZHJvcF9uYSh0b3RhbF9sZW5ndGgpJT4lDQogIGdyb3VwX2J5KGNsYWRlKSU+JQ0KICBzdW1tYXJpc2UoTj1uKCksVGF4YT1uX2Rpc3RpbmN0KHRheG9uKSklPiUNCiAgYWRkX3JvdyhjbGFkZT0iQWxsIFNwZWNpZXMiLA0KICAgICAgICAgIE49ZGF0YV9tb3V0aHMlPiUNCiAgICAgICAgICAgIGRyb3BfbmEodG90YWxfbGVuZ3RoKSU+JQ0KICAgICAgICAgICAgc3VtbWFyaXNlKE49bigpKSU+JXB1bGwoKSwNCiAgICAgICAgICBUYXhhPWRhdGFfbW91dGhzJT4lDQogICAgICAgICAgICBkcm9wX25hKHRvdGFsX2xlbmd0aCklPiUNCiAgICAgICAgICAgIHN1bW1hcmlzZShUYXhhPW5fZGlzdGluY3QodGF4b24pKSU+JXB1bGwoKSklPiUNCiAga2FibGUoY29sLm5hbWVzPWMoIkNsYWRlIiwiIyBPY2N1cmVuY2VzIiwiIyBUYXhhIiksDQogICAgICAgIGFsaWduPWMoImwiLCJjIiwiYyIpLA0KICAgICAgICBjYXB0aW9uPSJUYXhvbm9taWMgZGlzdHJpYnV0aW9uIG9mIG9ic2VydmF0aW9ucyBtYWRlIGluIHRoaXMgc3R1ZHkuIFBsYWNvZGVybXMgZm9yIHdoaWNoIG9ubHkgaGVhZCBkaW1lbnNpb25zIGFyZSBhdmFpbGFibGUgKGUuZy4sIDxpPkR1bmtsZW9zdGV1czwvaT4pIGFyZSBleGNsdWRlZCBmcm9tIHRoaXMgdGFibGUuIiklPiUNCiAgcm93X3NwZWMoNSwgYm9sZCA9IFQpJT4lDQogIGthYmxlX3N0eWxpbmcoKQ0KYGBgDQoNCiMgQWRkaXRpb25hbCBub3RlcyBvbiBkYXRhIGNvbGxlY3Rpb24NCg0KIyMgTWV0aG9kIG9mIGNvbGxlY3RpbmcgbWVhc3VyZW1lbnRzIGluIEFydGhyb2RpcmENCg0KSW4gY29sbGVjdGluZyBkYXRhIGZvciBhcnRocm9kaXJlcywgaXQgd2FzIG9mdGVuIG5lY2Vzc2FyeSB0byBtYWtlIHVzZSBvZiByZWNvbnN0cnVjdGlvbiBvciBjb21wb3NpdGUgc3BlY2ltZW5zLiBUaGlzIGlzIGJlY2F1c2UgbW9zdCBjb21wbGV0ZSBhcnRocm9kaXJlcyBhcmUgcHJlc2VydmVkIG9uIGZsYXR0ZW5lZCBzbGFicywgYW5kIHRodXMgaXQgaXMgbm90IHBvc3NpYmxlIHRvIG1lYXN1cmUgdG90YWwgbGVuZ3RoIGFuZCBtb3V0aCBkaW1lbnNpb25zIGluIHRoZSBzYW1lIHNwZWNpbWVucy4gRXZlbiB3aGVuIGFydGhyb2RpcmVzIGFyZSBleHRyYWN0ZWQgZnJvbSB0aGUgcm9jayB0byBjcmVhdGUgM0QgcmVjb25zdHJ1Y3Rpb25zIChlLmcuLCB0aGUgR29nbyBGb3JtYXRpb24gYXJ0aHJvZGlyZXMgb3IgKlBsb3VyZG9zdGV1cyogaW4gVsOpemluYSAxOTg4KSwgdGhpcyB0eXBpY2FsbHkgcmVzdWx0cyBpbiB0aGUgcG9zdC10aG9yYWNpYyBheGlhbCBza2VsZXRvbiBiZWluZyBsZWZ0IGJlaGluZCBiZWNhdXNlIGl0IGlzIHRvbyBmcmFnaWxlIHRvIG1vdW50LiBUaGlzIG1lYW5zIHJlbGF0aXZlIG1vdXRoIHNpemUgY2FuIG9ubHkgYmUgbWVhc3VyZWQgaW4gYSB2ZXJ5IHNtYWxsIG51bWJlciBvZiBhcnRocm9kaXJlcy4gQWRkaXRpb25hbCBpbmZvcm1hdGlvbiBvbiBob3cgbWVhc3VyZW1lbnRzIHdlcmUgY29sbGVjdGVkIGluIGFydGhyb2RpcmVzIGFyZSBkZXRhaWxlZCBiZWxvdy4NCg0KVGhlIGVhcmx5IG1pZGRsZSBEZXZvbmlhbiAoR2l2ZXRpYW4pICpDb2Njb3N0ZXVzIGN1c3BpZGF0dXMqIGlzIHRoZSBiZXN0IGtub3duIGFydGhyb2RpcmUgaW4gdGVybXMgb2YgaXRzIHBvc3QtdGhvcmFjaWMgYW5hdG9teSwgaGF2aW5nIGJlZW4gZGVzY3JpYmVkIGluIGRldGFpbCBieSBNaWxlcyAmIFdlc3RvbGwgKDE5NjgpLiBGb3IgdGhpcyBzdHVkeSwgbW9ycGhvbWV0cmljIGRhdGEgZm9yICpDb2Njb3N0ZXVzIGN1c3BpZGF0dXMqIChVSlAsIG1vdXRoIHdpZHRoLCBhbmQgaGVhZCBsZW5ndGgpIHdhcyB0YWtlbiBmcm9tIHRoZSByZWNvbnN0cnVjdGlvbnMgaW4gTWlsZXMgJiBXZXN0b2xsICgxOTY4KS4gVGhpcyByZWNvbnN0cnVjdGlvbnMgYXJlIGRlcml2ZWQgZnJvbSBzZXZlcmFsIGluZGl2aWR1YWxzLCBidXQgd2hlbiB0aGUgZGltZW5zaW9ucyBvZiB0aGlzIHJlY29uc3RydWN0aW9uIGFyZSBjb21wYXJlZCB0byBzcGVjaW1lbnMgb2YgKkNvY2Nvc3RldXMqIHRoZXkgYXBwZWFyIHRvIGFjY3VyYXRlbHkgcmVmbGVjdCB0aGUgcHJvcG9ydGlvbnMgb2YgdGhpcyBzcGVjaWVzLiBUaGUgcmVjb25zdHJ1Y3Rpb24gb2YgKkMuIGN1c3BpZGF0dXMqIGluIE1pbGVzICYgV2VzdG9sbCAoMTk2ODogZmlnLiA0OCkgaGFzIGEgdG90YWwgbGVuZ3RoIG9mIDM5LjQgY20gKHNjYWxlIGlzIG5vdCBwcm92aWRlZCBmb3IgZmlnLiA0OCwgYnV0IHRvdGFsIGxlbmd0aCBjYW4gYmUgZXN0aW1hdGVkIGZyb20gaGVhZCBsZW5ndGgsIHdoaWNoIGlzIHByb3ZpZGVkIGluIGZpZy4gMjNiKS4NCg0KTW91dGggd2lkdGgsIGJ1dCBub3QgVUpQLCBjb3VsZCBiZSBtZWFzdXJlZCBpbiB0aHJlZSBhZGRpdGlvbmFsIGFydGhyb2RpcmVzIGtub3duIGZyb20gY29tcGxldGUgcmVtYWluczogKkhvbG9uZW1hIHdlc3RvbGlpKiAoc2VlIE1pbGVzICYgV2hpdGUgMTk3MTsgVHJpbmFqc3RpYyAxOTk5KSwgKlBsb3VyZG9zdGV1cyBjYW5hZGVuc2lzKiAoc2VlIFbDqXppbmEgMTk4ODsgVsOpemluYSAxOTkwKSwgYW5kICpJbmNpc29zY3V0dW0gcml0Y2hlaSogKHNlZSBEZW5uaXMgJiBNaWxlcyAxOTgxOyBUcmluYWpzdGljIGV0IGFsLiAyMDEzKS4gTW91dGggd2lkdGggaW4gdHdvIG90aGVyIHNwZWNpZXMsICpXYXRzb25vc3RldXMgZmxldHRpKiBhbmQgKk1pbGxlcm9zdGV1cyBtaW5vciosIGNvdWxkIG5vdCBiZSBtZWFzdXJlZCBkaXJlY3RseSBidXQgY291bGQgYmUgYXBwcm94aW1hdGVkIGJhc2VkIG9uIHRoZSBwcmVzZXJ2ZWQgZXh0ZW50IG9mIHRoZSBzcGVjaW1lbnMgKG1lYXN1cmluZyBmcm9tIHRoZSBhcHByb3hpbWF0ZSBkaXN0YW5jZSBiZXR3ZWVuIHRoZSBlbmRzIG9mIHRoZSBzdXByYW9yYWwgbGF0ZXJhbCBsaW5lKS4gRm9yICpILiB3ZXN0b2xpaSosIHRoZSBza3VsbCBvZiB0aGlzIHRheG9uIGlzIGZpZ3VyZWQgaW4gYSBzcGxheWVkIG91dCwgc2xpZ2h0bHkgY3J1c2hlZCBmYXNoaW9uIGluIE1pbGVzICYgV2hpdGUgKDE5NzE6IGZpZy4gNCksIGJ1dCB0aGlzIGRlcGljdGlvbiB3YXMgY29uc2lkZXJlZCBjbG9zZSBlbm91Z2ggdG8gdGhlIGFjdHVhbCBza3VsbCBkaW1lbnNpb25zICB0byBoaWdobGlnaHQgdGhlIHRyYWl0cyBvZiBpbnRlcmVzdC4NCg0KTWVhc3VyZW1lbnRzIG9mICpQbG91cmRvc3RldXMgY2FuYWRlbnNpcyogd2VyZSB0YWtlbiBmcm9tIE1OSE4gMi0xNzcsIHdoaWNoIGlzICh3YXMpIGEgY29tcGxldGUgc2tlbGV0b24gb2YgKlAuIGNhbmFkZW5zaXMqIHByZXNlcnZpbmcgdGhlIGVudGlyZSBhbmltYWwgZnJvbSB0aGUgcm9zdHJhbCBwbGF0ZSB0byB0aGUgdGlwIG9mIHRoZSB2ZXJ0ZWJyYWwgY29sdW1uIChvbmx5IG1pc3NpbmcgdGhlIHNvZnQgdGlzc3VlIG91dGxpbmUgb2YgdGhlIGNhdWRhbCBmaW4pLiBPcmlnaW5hbGx5IHByZXNlcnZlZCBhcyBhIGZsYXR0ZW5lZCBwbGF0ZSwgVsOpemluYSAoMTk4OCkgZXh0cmFjdGVkIHRoZSBza3VsbCBhbmQgdHJ1bmsgYXJtb3IgdG8gcHJvZHVjZSBhIHRocmVlLWRpbWVuc2lvbmFsIG1vdW50IChWw6l6aW5hIDE5ODg6IHBsYXRlcyAxLTIpLCBsZWF2aW5nIHRoZSBwb3N0LXRob3JhY2ljIHNrZWxldG9uIGluIHRoaXMgbWF0cml4ICh0aGlzIG1hdGVyaWFsIGN1cnJlbnRseSBjYXRhbG9ndWVkIGFzIE1OSE0gMDItMTc3YykuIEhlYWQgYW5kIHRob3JhY2ljIGFybW9yIG1lYXN1cmVtZW50cyB3ZXJlIHRha2VuIGZyb20gdGhlIGZpZ3VyZXMgaW4gVsOpemluYSAoMTk4OCksIHdoZXJlYXMgdGhlIHBvc3QtdGhvcmFjaWMgYXJtb3IgbGVuZ3RoIHdhcyBtZWFzdXJlZCBiYXNlZCBvbiBwaG90b3Mgb2YgTU5ITSAwMi0xNzdjIHByb3ZpZGVkIGJ5IEouIEtlcnIuIFVuZm9ydHVuYXRlbHksIHRoZXJlIGFyZSBubyBwaWN0dXJlcyBvZiB0aGlzIHNwZWNpbWVuIHByaW9yIHRvIHByZXBhcmF0aW9uIChKLiBLZXJyLCBwZXJzLiBjb21tLiksIHNvIGl0IGlzIGRpZmZpY3VsdCB0byBkZXRlcm1pbmUgZXhhY3RseSBob3cgbG9uZyB0aGlzIHNwZWNpbWVuIG9yaWdpbmFsbHkgd2FzLiBBc3N1bWluZyB0aGUgcmVtYWluaW5nIHBvc3R0aG9yYWNpYyBtYXRlcmlhbCByZXByZXNlbnRzIHRoZSBib2R5IHBvc3RlcmlvciB0byB0aGUgZW5kIG9mIHRoZSB2ZW50cmFsIHNoaWVsZCwgYXMgaW1wbGllZCBieSBob2xsb3dzIGxlZnQgYnkgdGhlIGV4dHJhY3Rpb24gb2YgcGxhdGVzIChwb3N0ZXJvdmVudHJvbGF0ZXJhbHM/KSBhbmQgYSBsYXJnZSBlbGVtZW50IGF0IHRoZSBwcmVzZXJ2ZWQgYW50ZXJpb3IgZW5kIG9mIHRoZSBza2VsZXRvbiB0aGF0IGFwcGVhcnMgdG8gcmVwcmVzZW50IGEgY2xhc3BlciBvciBpbGlhYyBwcm9jZXNzIG9mIHRoZSBwZWx2aWMgZ2lyZGxlIE1OSE0gMDItMTc3YyB3b3VsZCBoYXZlIGJlZW4gYWJvdXQgMzcuNSBjbSBsb25nIG9yaWdpbmFsbHkuIFRoZSBzYW1lIGF1dGhvciAoVsOpemluYSAxOTkwKSBsYXRlciBwcm92aWRlcyBhIGZ1bGwtYm9keSBsYXRlcmFsIHJlY29uc3RydWN0aW9uIG9mICpQLiBjYW5hZGVuc2lzKi4gSG93ZXZlciwgdGhpcyByZWNvbnN0cnVjdGlvbiBzaG93cyBmZWF0dXJlcyB0aGF0IG1ha2UgaXRzIHByb3BvcnRpb25zIHN1c3BlY3QsIGluY2x1ZGluZyBhIHBvc3QtdGhvcmFjaWMgcmVnaW9uIHRoYXQgYXBwZWFycyB0byBiZSBkaXJlY3RseSBjb3BpZWQgZnJvbSB0aGUgZmlndXJlIG9mICpDLiBjdXNwaWRhdHVzKiBpbiBNaWxlcyAmIFdlc3RvbGwgKDE5NjgpLCBhbGJlaXQgd2l0aCBhIG1vZGlmaWVkIHBlbHZpYyBnaXJkbGUsIHJhdGhlciB0aGFuIHJlZmxlY3RpbmcgdGhlIHByb3BvcnRpb25zIG9mICpQbG91cmRvc3RldXMqIHNwZWNpbWVucy4gVGhlcmVmb3JlLCB0aGUgcHJlc2VydmVkIGRpbWVuc2lvbnMgb2YgTU5ITSAwMi0xNzcgc2VlbSBtb3JlIHJlbGlhYmxlIGZvciBlc3RpbWF0aW5nIHRoZSBwcm9wb3J0aW9ucyBvZiB0aGlzIHNwZWNpZXMuIEl0IHdhcyBub3QgcG9zc2libGUgdG8gY29udGFjdCBELiBWw6l6aW5hIGZvciBjbGFyaWZpY2F0aW9uIG9uIHRoaXMgc3BlY2ltZW4gYXMgdGhpcyByZXNlYXJjaGVyIGlzIG5vdyBkZWNlYXNlZCAoSi4gS2VyciwgcGVycy4gY29tbS4pLg0KDQpGaW5hbGx5LCBmb3IgKkluY2lzb3NjdXR1bSByaXRjaGVpKiwgdGhlIHJlY29uc3RydWN0aW9uIHByZXNlbnRlZCBpbiBUcmluYWpzdGljIGV0IGFsLiAoMjAxMzogZmlnLiAxYykgd2FzIHVzZWQgYXMgYSBzdGFydGluZyBwb2ludCwgYW5kIHRoZW4gdGhlIG1hdGVyaWFsIHdhcyBjcm9zcy1yZWZlcmVuY2VkIGFuZCBzY2FsZWQgd2l0aCAqSW5jaXNvc2N1dHVtKiBmb3NzaWwgbWF0ZXJpYWwgKE5ITVVLIFA1MDkyMyBhbmQgV0FNIDAuMy4zLjI4KS4gVGhlIHByb3BvcnRpb25zIG9mIHRoaXMgcmVjb25zdHJ1Y3Rpb24gd2VyZSBzaW1pbGFyIGVub3VnaCB0byAgYWN0dWFsIGZvc3NpbHMgb2YgKkkuIHJpdGNoZWkqIHRoYXQgdGhpcyByZWNvbnN0cnVjdGlvbiB3YXMgY29uc2lkZXJlZCBhIHJlbGlhYmxlIHJlcHJlc2VudGF0aW9uIG9mIHRoZSBwcm9wb3J0aW9ucyBvZiAqSW5jaXNvc2N1dHVtKi4gTW91dGggd2lkdGggZm9yICpJbmNpc29zY3V0dW0qIHdhcyBjYWxjdWxhdGluZyBieSBzY2FsaW5nIHRoZSBtb3V0aCB3aWR0aCBvZiBOS01VSyBQViBQIDUwOTI5ICAoRGVubmlzICYgTWlsZXMgMTk4MTogZmlnLiA5KSB0byB0aGUgc2l6ZSBvZiB0aGUgcmVjb25zdHJ1Y3Rpb24gaW4gVHJpbmFqc3RpYyBldCBhbC4gKDIwMTMpIHVzaW5nIHNrdWxsIGxlbmd0aC4gVGhpcyBlbnN1cmVkIG1vdXRoIHdpZHRoIHdhcyBtZWFzdXJlZCBkaXJlY3RseSBmcm9tIGZvc3NpbCBzcGVjaW1lbnMgb2YgKkkuIHJpdGNoZWkqLiBUaGUgc2t1bGwgb2YgTkhNVUsgUFYgUCA1MDkyOSBpcyBzaW1pbGFyIGluIHNpemUgdG8gdGhlIHJlY29uc3RydWN0aW9uIGluIFRyaW5hanN0aWMgZXQgYWwuICgyMDEzKSAoNS43NSB2ZXJzdXMgNS43NCBjbSksIHN1Z2dlc3RpbmcgYmlhcyBmcm9tIGFsbG9tZXRyaWMgc2NhbGluZywgaWYgaXQgZXhpc3RzLCBzaG91bGQgYmUgbWlub3IuDQoNCk5vdGFibHksIHRoZSByZWNvbnN0cnVjdGlvbiBvZiAqSW5jaXNvc2N1dHVtKiBpbiBUcmluYWpzdGljIGV0IGFsLiAoMjAxMzogZmlnLiAxYykgbGFja3MgdGhlIGNhdWRhbCBmaW4uIFRoaXMgaXMgYmVjYXVzZSBub25lIG9mIHRoZSBldWJyYWNoeXRob3JhY2lkIGFydGhyb2RpcmVzIGZyb20gdGhlIEdvZ28gRm9ybWF0aW9uIHByZXNlcnZlcyBhbiBhc3NvY2lhdGVkIGNhdWRhbCBmaW4gKFRyaW5hanN0aWMgZXQgYWwuIDIwMjI7IFRyaW5hanN0aWMgcGVycy4gY29tbS4pLiBUaHVzLCBkZXNwaXRlIHRoZSBHb2dvIEZvcm1hdGlvbiBwcm9kdWNpbmcgYSBzaWduaWZpY2FudCBudW1iZXIgb2YgZXh0cmVtZWx5IHdlbGwtcHJlc2VydmVkIGFydGhyb2RpcmUgc3BlY2ltZW5zLCBub25lIG9mIHRoaXMgbWF0ZXJpYWwgY2FuIGJlIHVzZWQgdG8gZGV0ZXJtaW5lIHRoZSBib2R5IHByb3BvcnRpb25zIG9yIG92ZXJhbGwgbGVuZ3RoIG9mIGFydGhyb2RpcmVzLiBCZWNhdXNlIGNvbXBsZXRlIGFydGhyb2RpcmVzIGFyZSBzbyByYXJlIGFuZCB0aGUgcHJlY2F1ZGFsIGFuYXRvbXkgb2YgKkluY2lzb3NjdXR1bSogaXMgY29tcGxldGVseSBrbm93biwgdG90YWwgbGVuZ3RoIHdhcyBlc3RpbWF0ZWQgZm9yICpJLiByaXRjaGVpKiBhc3N1bWluZyB0aGUgdGhlIGNhdWRhbCBmaW4gcmVwcmVzZW50ZWQgfjMyJSBvZiB0b3RhbCBsZW5ndGggKGkuZS4sIHRvdGFsIGxlbmd0aC9wcmVjYXVkYWwgbGVuZ3RoID0gMS40NikuIFRoaXMgaXMgdGhlIHByb3BvcnRpb24gYmV0d2VlbiBjYXVkYWwgZmluIGxlbmd0aCBhbmQgdG90YWwgbGVuZ3RoIGluIHRoZSByZWNvbnN0cnVjdGlvbiBvZiAqQ29jY29zdGV1cyBjdXNwaWRhdHVzKiBpbiBNaWxlcyBhbmQgV2VzdG9sbCAoMTk2OCkuIEFkZGl0aW9uYWxseSwgdGhpcyByYXRpbyBpcyBzaW1pbGFyIHRvIHRoZSB0b3RhbCBsZW5ndGgvcHJlY2F1ZGFsIGxlbmd0aCByYXRpbyBpbiBtYW55IGV4dGFudCBzaGFya3MgKHNlZSBiZWxvdyksIHN1Z2dlc3RpbmcgaXQgcmVwcmVzZW50cyBhIHJlYXNvbmFibGUgYXBwcm94aW1hdGlvbiBmb3IgKkkuIHJpdGNoZWkqLg0KDQpGb3IgKk1pbGxlcm9zdGV1cyBtaW5vciosIGluaXRpYWxseSBubyBjb21wbGV0ZSBzcGVjaW1lbnMgb2YgKk1pbGxlcm9zdGV1cyBtaW5vciogY291bGQgYmUgb2JzZXJ2ZWQgZm9yIHRoaXMgc3R1ZHkuIEluc3RlYWQsIGNvbXBvc2l0ZSB2YWx1ZXMgZm9yICpNaWxsZXJvc3RldXMqIHdlcmUgY2FsY3VsYXRlZCB1c2luZyB0aGUgYXJtb3IgcHJvcG9ydGlvbnMgb2YgdGhlIHNwZWNpbWVuIGRlc2NyaWJlZCBieSBEZXNtb25kICgxOTc0KSAoQy4zNjkgb2YgdGhhdCBzdHVkeSwgY3VycmVudGx5IGNhdGFsb2dlZCBhcyBMRFVDWi1WOTk4KSwgd2hpY2ggcHJlc2VydmVzIG1vc3Qgb2YgdGhlIGFybW9yIGJ1dCB2ZXJ5IGxpdHRsZSBvZiB0aGUgYXhpYWwgc2tlbGV0b24gKERlc21vbmQgMTk3NDogcGwuIDEpIGFuZCB0aGUgcG9zdHRob3JhY2ljIGRpbWVuc2lvbnMgb2YgYW4gdW5kZXNjcmliZWQgc3BlY2ltZW4gb2YgTS4gbWlub3IgY29sbGVjdGVkIGJ5IE0uIEouIE5ld21hbi4gVGhlIE5ld21hbiBzcGVjaW1lbiBpcyBtaXNzaW5nIHRoZSBtaWRkbGUgY2F1ZGFsIHZlcnRlYnJhZSBidXQgcHJlc2VydmVzIHRoZSByZW1haW5kZXIgb2YgdGhlIHBvc3QtdGhvcmFjaWMgc2tlbGV0b24sIGluY2x1ZGluZyB0aGUgZG9yc2FsIGZpbiwgcGVsdmljIGdpcmRsZSwgYW5hbCBwbGF0ZSwgYW5kIGRpc3RhbCBjYXVkYWwgdmVydGVicmFlIHRvIHRoZSBjYXVkYWwgZmluIHRpcC4gVGhlIHNwZWNpbWVuIG1lYXN1cmVkIDYuMzI1IGNtIGZyb20gdGhlIHNub3V0IHRvIHRoZSBlbmQgb2YgdGhlIHZlbnRyYWwgYXJtb3IgYW5kIGhhcyBhbiBhcHByb3hpbWF0ZSB0b3RhbCBsZW5ndGggb2YgMTcgY20uIFRoZSBwb3N0dGhvcmFjaWMgZGltZW5zaW9ucyBvZiB0aGlzIHNwZWNpbWVuIHdlcmUgc2NhbGVkIHRvIHRoYXQgb2YgTERVQ1otVjk5OCB1c2luZyB0aGUgbGVuZ3RoIG9mIHRoZSBzcGVjaW1lbiB0byB0aGUgZW5kIG9mIHRoZSB2ZW50cmFsIGFybW9yLiBUaGUgc2t1bGwgb2YgTmV3bWFu4oCZcyBzcGVjaW1lbiBpcyBwb29ybHkgcHJlc2VydmVkLCB0aGUgb3V0bGluZSBvZiB0aGUgaGVhZCBjYW4gYmUgZGlzdGluZ3Vpc2hlZCBidXQgdGhlIHJlc3Qgb2YgdGhlIGNyYW5pYWwgYW5hdG9teSBjYW5ub3QuIFRodXMsIGl0IGNhbm5vdCBiZSB1c2VkIG9uIGl0cyBvd24uIE1vdXRoIHdpZHRoIGluICpNaWxsZXJvc3RldXMqIHdhcyBhcHByb3hpbWF0ZWQgYnkgbWVhc3VyaW5nIGJldHdlZW4gdGhlIHZlbnRyYWwgZW5kcyBvZiB0aGUgc3VwcmFvcmFsIHNlbnNvcnkgY2FuYWwuIExhdGUgaW4gdGhlIHN0dWR5IGl0IHdhcyBwb3NzaWJsZSB0byBhY2Nlc3Mgc3BlY2ltZW5zIG9mICpNaWxsZXJvc3RldXMqIGluIHRoZSBGaWVsZCBNdXNldW0gb2YgTmF0dXJhbCBIaXN0b3J5LCBidXQgbW91dGggd2lkdGggY291bGQgbm90IGJlIG1lYXN1cmVkIGluIHRoZXNlIHNwZWNpbWVucy4NCg0KIyMgTGVuZ3RoLWxlbmd0aCBlcXVhdGlvbnMgZm9yIHNoYXJrcw0KDQpJbiBldmFsdWF0aW5nIHRoZSBkYXRhc2V0IG9mIExvd3J5IGV0IGFsLiAoMjAwOSkgaXQgd2FzIGFsc28gb2YgaW50ZXJlc3QgdG8gc2VlIHdoZXRoZXIgY2F1ZGFsIGZpbiBwcm9wb3J0aW9ucyBiZXR3ZWVuIHRoZSB2YXJpb3VzIGdyb3VwcyBleGFtaW5lZCBtaWdodCBzaWduZmljYW50bHkgYmlhcyB0aGUgYWxsb21ldHJpYyByZWxhdGlvbnNoaXBzIHNlZW4gYmV0d2VlbiBVSlAgYW5kIHRvdGFsIGxlbmd0aCBpbiBzaGFya3MuIFRoZXJlZm9yZSwgdG90YWwgbGVuZ3RoIHdhcyBjb252ZXJ0ZWQgdG8gcHJlY2F1ZGFsIGxlbmd0aCBmb3IgZWFjaCBzaGFyayB1c2luZyB0aGUgbGVuZ3RoLWxlbmd0aCBlcXVhdGlvbnMgaW4gQnJhbnN0ZXR0ZXIgJiBTdGlsZXMgKDE5ODcpLCBDbGlmZiAoMTk5NSksIEZyYW5jaXMgKDIwMDYpLCBQaWVyY3kgZXQgYWwuICgyMDA3KSwgV2hpdG5leSAmIENyb3cgKDIwMDcpLCBhbmQgUG9sbGFjayBldCBhbC4gKDIwMTkpLiBGb3IgKklzdXJ1cyBwYXVjdXMqIG5vIGxlbmd0aC1sZW5ndGggZXF1YXRpb24gd2FzIGF2YWlsYWJsZSwgc28gb25lIHdhcyBjcmVhdGVkIHVzaW5nIHRoZSBkYXRhIGZyb20gR2FycmljayAoMTk2NykgYW5kIEdpbG1vcmUgKDE5ODMpLg0KDQojIyBOb3RlIG9uIHRoZSB2ZW50cmFsIGFybW9yIG9mICpEdW5rbGVvc3RldXMqDQoNCkZvciB0aGUgcmVjb25zdHJ1Y3Rpb24gb2YgdGhlIGFybW9yIG9mICpEdW5rbGVvc3RldXMqIGluIHRoZSBtYW51c2NyaXB0LCB0aGUgc3BlY2ltZW4gaXMgZGVwaWN0ZWQgYXMgaXMgd2l0aCBubyBhbHRlcmF0aW9ucy4gVGhlIGZpZ3VyZSB3YXMgZHJhd24gZGlyZWN0bHkgZnJvbSBpbWFnZXMgb2YgdGhlIHNwZWNpbWVuLCBhbmQgc28gcmVwcmVzZW50cyB0aGUgYWN0dWFsIHByb3BvcnRpb25zIG9mIHRoZSBtYXRlcmlhbC4NCg0KVGhlIG9ubHkgbW9kaWZpY2F0aW9uIG1hZGUgd2FzIHRoYXQgdGhlICpEdW5rbGVvc3RldXMqIHNwZWNpbWVucyB3ZXJlIHJlY29uc3RydWN0ZWQgd2l0aCBhIGN1cnZlZCB2ZW50cmFsIHNoaWVsZCwgcmF0aGVyIHRoYW4gdGhlIGZsYXR0ZW5lZCBvbmUgc2VlbiBpbiB0aGUgbW91bnRlZCBzcGVjaW1lbnMuIFRoaXMgaXMgYmVjYXVzZSB0aHJlZS1kaW1lbnNpb25hbGx5IHByZXNlcnZlZCBmb3NzaWxzIG9mIGFydGhyb2RpcmVzIHNob3cgdGhlIHZlbnRyYWwgYXJtb3Igb2YgbWFyaW5lIGFydGhyb2RpcmVzIHdhcyBuYXR1cmFsbHkgY3VydmVkIChlLmcuLCBHYXJkaW5lciAmIE1pbGVzIDE5OTQ7IE1pbGVzICYgRGVubmlzIDE5NzkpLCBhbmQgdGh1cyB0aGVzZSBhbmltYWxzIGhhZCBhIHRvcnBlZG8tbGlrZSBjcm9zcy1zZWN0aW9uLiBIb3dldmVyLCB0aGlzIGRpc2NvdmVyeSB3YXMgbWFkZSBsb25nIGFmdGVyIHRoZSAqRHVua2xlb3N0ZXVzKiBzcGVjaW1lbnMgYXQgdGhlIENNTkggd2VyZSBtb3VudGVkIChjaXJjYSAxOTI5OyBKLiBUYWl0LCBwZXJzLiBjb21tLikuIEhvd2V2ZXIsIGFmdGVyIGl0IHdhcyBkaXNjb3ZlcmVkIGFydGhyb2RpcmVzIG5hdHVyYWxseSBoYWQgY3VydmVkIHZlbnRyYWwgYXJtb3IsIHJldHJvZGVmb3JtYXRpb24gb2YgdGhlIHZlbnRyYWwgc2hpZWxkIHdhcyBkZWVtZWQgdG9vIGRlc3RydWN0aXZlIHRvIHRoZSBvcmlnaW5hbCBtYXRlcmlhbCBiZWNhdXNlIGl0IGludm9sdmVkIG1hbnVhbGx5IGJyZWFraW5nIGFuZCByZXNldHRpbmcgdGhlIHNwZWNpbWVucyAoRC4gQ2hhcG1hbiwgcGVycy4gY29tbS4pLiBUaHVzLCB0aGUgQ01OSCAqRHVua2xlb3N0ZXVzKiBoYXZlIHJldGFpbmVkIHRoZWlyIGZsYXR0ZW5lZCB2ZW50cmFsIGFybW9yIGRlc3BpdGUgdGhpcyBmZWF0dXJlIG90aGVyd2lzZSBiZWluZyBvdXRkYXRlZC4gRGVzcGl0ZSBiZWluZyBtb3VudGVkIHdpdGggZmxhdCB2ZW50cmFsIGFybW9yLCBkZXBpY3RpbmcgdGhlIHNwZWNpbWVucyBhcyBzdWNoIHdvdWxkIG5vdCBhY2N1cmF0ZWx5IGRlcGljdCB0aGUgYW5hdG9teSBvZiB0aGlzIHRheG9uIGFuZCB3b3VsZCByZXN1bHQgaW4gdGhlIGJvZHkgYXBwZWFyaW5nIHVucmVhbGlzdGljYWxseSBib3h5LiBUaHVzLCB0aGUgdmVudHJhbCBjdXJ2YXR1cmUgb2YgdGhlIGFybW9yIHdhcyBhcHByb3hpbWF0ZWQgYmFzZWQgb24gdGhlIGN1cnZhdHVyZSBvZiB0aGUgc3BlY2ltZW5zLg0KDQojIFNoYXJrIFVKUCBwbG90DQoNCihyZWY6VUpQKSBQbG90IG9mIHVwcGVyIGphdyBwZXJpbWV0ZXIgYWdhaW5zdCB0b3RhbCBsZW5ndGggKGJvdGggdW50cmFuc2Zvcm1lZCkgZnJvbSBMb3dyeSBldCBhbC4gKDIwMDkpLCBzaG93aW5nIHRoZSBkaWZmZXJlbnQgYWxsb21ldHJpYyBwYXR0ZXJucyBpbiBTcGh5cm5pZGFlLCBMYW1uaWRhZSwgYW5kIENhcmNoYXJoaW5pZGFlIHJlbGF0aXZlIHRvIHRoZSBhbGwgc3BlY2llcyByZWdyZXNzaW9uIG1vZGVsLg0KDQpgYGB7cixmaWcuY2FwPSIocmVmOlVKUCkifQ0KKGxvd3J5MjAwOTwtZ2dwbG90KGxvd3J5X3NoYXJrcyxhZXMoeT1VSlAseD10b3RhbF9sZW5ndGgpKSsNCiAgZ2VvbV9wb2ludChhZXMoZmlsbD0iQWxsIFNwZWNpZXMiLHNoYXBlPSJBbGwgU3BlY2llcyIpKSsNCiAgZ2VvbV9wb2ludChhZXMoZmlsbD1mYW1pbHksc2hhcGU9ZmFtaWx5KSkrDQogIGdlb21fc21vb3RoKG1ldGhvZD0ibG0iLGZvcm11bGE9eX54LGFlcyhjb2xvcj0iQWxsIFNwZWNpZXMiKSkrDQogIGdlb21fc21vb3RoKG1ldGhvZD0ibG0iLGZvcm11bGE9eX54LGFlcyhjb2xvcj1mYW1pbHksZmlsbD1mYW1pbHkpLGFscGhhPTAuMjUpKw0KICBnZW9tX3Ntb290aChtZXRob2Q9ImxtIixmb3JtdWxhPXl+eCxjb2xvcj0iYmxhY2siLGFscGhhPTAuMjUpKw0KICBzY2FsZV9jb2xvcl9tYW51YWwodmFsdWVzPWMoImJsYWNrIixodWVfcGFsKCkoMykpKSsNCiAgc2NhbGVfZmlsbF9tYW51YWwodmFsdWVzPWMoIk5BIixodWVfcGFsKCkoMykpLG5hLnZhbHVlPSJOQSIpKw0KICBzY2FsZV9zaGFwZV9tYW51YWwodmFsdWVzPWMoTkEsMjEsMjIsMjQpLG5hLnZhbHVlPU5BKSsNCiAgbGFicyh4PSJUb3RhbCBMZW5ndGggKGNtKSIseT0iVXBwZXIgSmF3IFBlcmltZXRlciAoY20pIiwNCiAgICAgICBjb2xvcj0iRmFtaWx5IixzaGFwZT0iRmFtaWx5IixmaWxsPSJGYW1pbHkiKSsNCiAgdGhlbWVfY2xhc3NpYygpKw0KICB0aGVtZShsZWdlbmQucG9zaXRpb249YygwLjgsMC4yNSkpKQ0KZ2dzYXZlKCJBcnRocm9kaXJlIE1vdXRocyAoTG93cnkgMjAwOSByZWdyZXNzaW9uKS5wZGYiLGxvd3J5MjAwOSx3aWR0aD0xNjUsaGVpZ2h0PTEyNSx1bml0cz0ibW0iLA0KICAgICAgIGRldmljZT0icGRmIikNCmBgYA0KDQpOb3RlIHRoZSBwb3NpdGl2ZSBhbGxvbWV0cnkgaW4gdXBwZXIgamF3IHBlcmltZXRlciB2ZXJzdXMgdG90YWwgbGVuZ3RoIGR1ZSB0byB0aGUgZmFjdCB0aGF0IHRoZSB2ZXJ5IGxhcmdlc3QgbWVhc3VyZWQgc2hhcmtzIGFsbCBwZXJ0YWluIHRvICpDYXJjaGFyb2RvbiBjYXJjaGFyaWFzKiwgdGh1cyBjYXVzaW5nIHRoZSBzbG9wZSBvZiB0aGUgcmVncmVzc2lvbiBsaW5lIHRvIGJlIGhpZ2hlciAoaS5lLiwgcHJvZHVjZSBzbGlnaHRseSBzaG9ydGVyIGxlbmd0aHMpIGF0IGxhcmdlciB2YWx1ZXMgb2YgYFVKUGAsIGRlc3BpdGUgdGhlIHRocmVlIGdyb3VwcyBub3Qgc2hvd2luZyBzdWNoIGEgcmVsYXRpb25zaGlwIGJ5IHRoZW1zZWx2ZXMuDQoNCmBgYHtyfQ0Kc3VtbWFyeShsbSh0b3RhbF9sZW5ndGh+VUpQKmZhbWlseSxsb3dyeV9zaGFya3MpKQ0KYGBgDQoNCkJhc2VkIG9uIHRoaXMsIGl0IGNhbiBiZSBzZWVuIHRoYXQgc3BoeXJuaWRzIGFuZCBjYXJjaGFyaGluaWRzIGhhdmUgc2ltaWxhciBzbG9wZXMgKGJ1dCBjYXJjaGFyaGluaWRzIGFuZCBsYW1uaWRzIGRvIG5vdCksIGFuZCB0aGUgaW50ZXJjZXB0cyBiZXR3ZWVuIHRoZSBlcXVhdGlvbnMgb2YgdGhlIHRocmVlIGdyb3VwcyBhcmUgYWxsIHNpZ25pZmljYW50bHkgZGlmZmVyZW50IGZyb20gb25lIGFub3RoZXIuDQoNCiMjIFVzaW5nIFByZWNhdWRhbCBMZW5ndGgNCg0KKHJlZjpVSlBwcmVjYXVkYWwpIFBsb3Qgb2YgdXBwZXIgamF3IHBlcmltZXRlciBhZ2FpbnN0IHByZWNhdWRhbCBsZW5ndGggKGJvdGggdW50cmFuc2Zvcm1lZCkgZnJvbSBMb3dyeSBldCBhbC4gKDIwMDkpLCBzaG93aW5nIHRoZSBkaWZmZXJlbnQgYWxsb21ldHJpYyBwYXR0ZXJucyBpbiBTcGh5cm5pZGFlLCBMYW1uaWRhZSwgYW5kIENhcmNoYXJoaW5pZGFlIHJlbGF0aXZlIHRvIHRoZSBhbGwgc3BlY2llcyByZWdyZXNzaW9uIG1vZGVsLg0KDQpgYGB7cixmaWcuY2FwPSIocmVmOlVKUHByZWNhdWRhbCkiLGZpZy5hc3A9MC42LGZpZy53aWR0aD05fQ0KZ2dwbG90KGxvd3J5X3NoYXJrcyxhZXMoeT1wcmVjYXVkYWxfbGVuZ3RoLHg9dG90YWxfbGVuZ3RoKSkrDQogIGdlb21fcG9pbnQoYWVzKGZpbGw9IkFsbCBTcGVjaWVzIixzaGFwZT0iQWxsIFNwZWNpZXMiKSkrDQogIGdlb21fcG9pbnQoYWVzKGZpbGw9ZmFtaWx5LHNoYXBlPWZhbWlseSkpKw0KICBnZW9tX3Ntb290aChtZXRob2Q9ImxtIixmb3JtdWxhPXl+eCxhZXMoY29sb3I9IkFsbCBTcGVjaWVzIikpKw0KICBnZW9tX3Ntb290aChtZXRob2Q9ImxtIixmb3JtdWxhPXl+eCxhZXMoY29sb3I9ZmFtaWx5LGZpbGw9ZmFtaWx5KSxhbHBoYT0wLjI1KSsNCiAgZ2VvbV9zbW9vdGgobWV0aG9kPSJsbSIsZm9ybXVsYT15fngsY29sb3I9ImJsYWNrIixhbHBoYT0wLjI1KSsNCiAgc2NhbGVfY29sb3JfbWFudWFsKHZhbHVlcz1jKCJibGFjayIsaHVlX3BhbCgpKDMpKSkrDQogIHNjYWxlX2ZpbGxfbWFudWFsKHZhbHVlcz1jKCJOQSIsaHVlX3BhbCgpKDMpKSxuYS52YWx1ZT0iTkEiKSsNCiAgc2NhbGVfc2hhcGVfbWFudWFsKHZhbHVlcz1jKE5BLDIxLDIyLDI0KSxuYS52YWx1ZT1OQSkrDQogIGxhYnMoeD0iUHJlY2F1ZGFsIExlbmd0aCAoY20pIix5PSJVcHBlciBKYXcgUGVyaW1ldGVyIChjbSkiLA0KICAgICAgIGNvbG9yPSJGYW1pbHkiLHNoYXBlPSJGYW1pbHkiLGZpbGw9IkZhbWlseSIpKw0KICB0aGVtZV9jbGFzc2ljKCkrDQogIHRoZW1lKGxlZ2VuZC5wb3NpdGlvbj1jKDAuOCwwLjI1KSkNCmBgYA0KDQpgYGB7cn0NCnN1bW1hcnkobG0ocHJlY2F1ZGFsX2xlbmd0aH5VSlAqZmFtaWx5LGxvd3J5X3NoYXJrcykpDQpgYGANCg0KV2hlbiBjb25zaWRlcmluZyBwcmVjYXVkYWwgbGVuZ3RoLCB0aGUgc2xvcGVzIG9mIHRoZSB0aHJlZSBncm91cHMgYXJlIG5vbnNpZ25pZmljYW50bHkgZGlmZmVyZW50LCBidXQgdGhlIGludGVyY2VwdHMgYmV0d2VlbiB0aGUgdGhyZWUgcmVncmVzc2lvbiBlcXVhdGlvbnMgYXJlLiBUaGlzIHN1Z2dlc3RzIHRoZSBkaWZmZXJlbmNlcyBpbiBzbG9wZSBpbiB0aGUgZXF1YXRpb24gY29uc2lkZXJpbmcgdG90YWwgbGVuZ3RoIGFyZSBkdWUgdG8gZGlmZmVyaW5nIGNhdWRhbCBmaW4gcHJvcG9ydGlvbnMgYmV0d2VlbiB0aGUgdGhyZWUgZ3JvdXBzLCBidXQgaXQgYWxzbyBzdWdnZXN0cyB0aGF0IHRoZSBkaWZmZXJlbnQgVUpQLXRvdGFsIGxlbmd0aCByZWxhdGlvbnNoaXBzIGNhbm5vdCBiZSBhdHRyaWJ1dGVkIHRvIGRpZmZlcmVuY3MgaW4gY2F1ZGFsIGZpbiBzaGFwZSBhbG9uZSAob3IgZWxzZSB0aGUgaW50ZXJjZXB0cyB3b3VsZCBub3QgZGlmZmVyIGJldHdlZW4gZ3JvdXBzKS4NCg0KYGBge3J9DQpsb3dyeV9zaGFya3MlPiUNCiAgZ3JvdXBfYnkodGF4b24pJT4lDQogIHN1bW1hcmlzZShtZWFuPXJvdW5kKG1lYW4ocGVyY2VudF9ib2R5KSoxMDAsMSksc2Q9cm91bmQoc2QocGVyY2VudF9ib2R5KSoxMDAsMyksZmFtaWx5PXVuaXF1ZShmYW1pbHkpKQ0KDQpsb3dyeV9zaGFya3MlPiUNCiAgZ3JvdXBfYnkodGF4b24pJT4lDQogIHN1bW1hcmlzZShtZWFuPW1lYW4ocGVyY2VudF9ib2R5KSxzZD1zZChwZXJjZW50X2JvZHkpLGZhbWlseT11bmlxdWUoZmFtaWx5KSklPiUNCiAgZ3JvdXBfYnkoZmFtaWx5KSU+JQ0KICBzdW1tYXJpc2UobWVhbj1yb3VuZChtZWFuKG1lYW4pKjEwMCwxKSkNCmBgYA0KDQojIENvbXBhcmlzb24gb2YgZXN0aW1hdGVzIHVzaW5nIHVwcGVyIGphdyBwZXJpbWV0ZXIgYW5kIG1vdXRoIHdpZHRoDQoNCiMjIFNwZWNpbWVuLWxldmVsIGFuYWx5c2lzDQoNCioqTm90ZToqKiAqQWxvcGlhcyogc3BwLiBhbmQgQ2hpbWFlcmlmb3JtZXMgd2VyZSBleGNsdWRlZCBmb3IgdGhpcyBtb2RlbC4NCg0KYGBge3J9DQpmaXQubW91dGh3aWR0aDwtbG0obG9nKHRvdGFsX2xlbmd0aCl+bG9nKG1vdXRoX3dpZHRoKSwNCiAgICAgICAgICAgICAgICAgICBkYXRhX21vdXRocyU+JQ0KICAgICAgICAgICAgICAgICAgICAgZmlsdGVyKGNsYWRlPT0iQ2hvbmRyaWNodGh5ZXMiLA0KICAgICAgICAgICAgICAgICAgICAgICAgICAgIG9yZGVyICE9ICJDaGltYWVyaWZvcm1lcyIsDQogICAgICAgICAgICAgICAgICAgICAgICAgICAgZ2VudXMhPSJBbG9waWFzIikpDQpzdW1tYXJ5KGZpdC5tb3V0aHdpZHRoKQ0KcmVncmVzc2lvbi5zdGF0cyhmaXQubW91dGh3aWR0aCkNCmBgYA0KDQojIyMgRGlhZ25vc3RpYyBwbG90cw0KDQpgYGB7cixmaWcud2lkdGg9MTAsZmlnLmhlaWdodD03LjV9DQpwYXIobWZyb3c9YygyLDIpKQ0KcGxvdChmaXQubW91dGh3aWR0aCkNCmBgYA0KDQpCYXNlZCBvbiB0aGVzZSBkaWFnbm9zdGljIHBsb3RzIHRoZSByZXNpZHVhbHMgYXJlIG5vcm1hbGx5IGRpc3RyaWJ1dGVkLCBob21vc2tlZGFzdGljLCBoYXZlIG5vIG91dGxpZXJzIHdpdGggc2lnbmlmaWNhbnQgbGV2ZXJhZ2UsIGFuZCBzdWdnZXN0IGEgbGluZWFyIG1vZGVsLCB0aHVzIHNob3VsZCBiZSBzYWZlIHRvIHVzZSB0byBwcmVkaWN0IG5ldyBkYXRhLg0KDQojIyMgQWxsb21ldHJpYyByZWxhdGlvbnNoaXANCg0KRm9yIHRoaXMgYW5hbHlzaXMgYHRvdGFsX2xlbmd0aGAgd2FzIHNldCBhcyB0aGUgaW5kZXBlbmRlbnQgdmFyaWFibGUsIHRvIGJldHRlciBleHByZXNzIGNoYW5nZXMgaW4gcHJvcG9ydGlvbiBpbiBgbW91dGhfd2lkdGhgIGFjcm9zcyBjaG9uZHJpY2h0aHlhbnMgb2YgZGlmZmVyZW50IHNpemVzLg0KDQpgYGB7cn0NCmRhdGFfbW91dGhzJT4lDQogIGZpbHRlcihjbGFkZT09IkNob25kcmljaHRoeWVzIiwhb3JkZXIgJWluJSBjKCJDaGltYWVyaWZvcm1lcyIpLA0KICAgICAgICAgZ2VudXMhPSJBbG9waWFzIiklPiUNCiAgZHJvcF9uYShtb3V0aF93aWR0aCx0b3RhbF9sZW5ndGgpJSQlDQogIGxtKGxvZyhtb3V0aF93aWR0aCl+bG9nKHRvdGFsX2xlbmd0aCksLiklPiUNCiAgc3VtbWFyeSgpDQpgYGANCg0KIyMgRXhhbWluaW5nIHRoZSBhbGxvbWV0cmljIHJlbGF0aW9uc2hpcCBvZiBtb3V0aCB3aWR0aCB1c2luZyBzcGVjaWVzIGF2ZXJhZ2VzDQoNCiMjIyBSZWdyZXNzaW9uIGVxdWF0aW9uDQoNCioqTm90ZToqKiAqQWxvcGlhcyogc3BwLiBhbmQgQ2hpbWFlcmlmb3JtZXMgd2VyZSBleGNsdWRlZCBmb3IgdGhpcyBtb2RlbC4gT25seSBhZHVsdCBhbmQgc3ViYWR1bHQgKHRoYXQgaXMsIGxhcmdlIGJ1dCBub3Qgc2V4dWFsbHkgbWF0dXJlKSBzcGVjaW1lbnMgd2VyZSBjb25zaWRlcmVkIGZvciB0aGlzIGFuYWx5c2lzLg0KDQpgYGB7cn0NCmZpdC5tb3V0aHdpZHRoX2F2ZXJhZ2VzPC1kYXRhX21vdXRocyU+JQ0KICBmaWx0ZXIoaXMubmEob250b2dlbnkpfG9udG9nZW55ICVpbiUgYygiYWR1bHQiLCJzdWJhZHVsdCIpLA0KICAgICAgICAgY2xhZGU9PSJDaG9uZHJpY2h0aHllcyIsIW9yZGVyICVpbiUgYygiQ2hpbWFlcmlmb3JtZXMiKSwNCiAgICAgICAgIGdlbnVzIT0iQWxvcGlhcyIpJT4lDQogIGRyb3BfbmEobW91dGhfd2lkdGgsdG90YWxfbGVuZ3RoKSU+JQ0KICBncm91cF9ieSh0YXhvbiklPiUNCiAgc3VtbWFyaXNlKG1vdXRoX3dpZHRoPW1lYW4obW91dGhfd2lkdGgpLHRvdGFsX2xlbmd0aD1tZWFuKHRvdGFsX2xlbmd0aCksb3JkZXI9dW5pcXVlKG9yZGVyKSklJCUNCiAgbG0obG9nKHRvdGFsX2xlbmd0aCl+bG9nKG1vdXRoX3dpZHRoKSwuKQ0KDQpzdW1tYXJ5KGZpdC5tb3V0aHdpZHRoX2F2ZXJhZ2VzKQ0KcmVncmVzc2lvbi5zdGF0cyhmaXQubW91dGh3aWR0aF9hdmVyYWdlcykNCmBgYA0KDQojIyMgQWxsb21ldHJpYyByZWxhdGlvbnNoaXANCg0KRm9yIHRoaXMgYW5hbHlzaXMgYHRvdGFsX2xlbmd0aGAgd2FzIHNldCBhcyB0aGUgaW5kZXBlbmRlbnQgdmFyaWFibGUsIHRvIGJldHRlciBleHByZXNzIGNoYW5nZXMgaW4gcHJvcG9ydGlvbiBpbiBgbW91dGhfd2lkdGhgIGFjcm9zcyBjaG9uZHJpY2h0aHlhbnMgb2YgZGlmZmVyZW50IHNpemVzLg0KDQpgYGB7cn0NCmRhdGFfbW91dGhzJT4lDQogIGZpbHRlcihpcy5uYShvbnRvZ2VueSl8b250b2dlbnkgJWluJSBjKCJhZHVsdCIsInN1YmFkdWx0IiksDQogICAgICAgICBjbGFkZT09IkNob25kcmljaHRoeWVzIiwhb3JkZXIgJWluJSBjKCJDaGltYWVyaWZvcm1lcyIpLA0KICAgICAgICAgZ2VudXMhPSJBbG9waWFzIiklPiUNCiAgZHJvcF9uYShtb3V0aF93aWR0aCx0b3RhbF9sZW5ndGgpJT4lDQogIGdyb3VwX2J5KHRheG9uKSU+JQ0KICBzdW1tYXJpc2UobW91dGhfd2lkdGg9bWVhbihtb3V0aF93aWR0aCksdG90YWxfbGVuZ3RoPW1lYW4odG90YWxfbGVuZ3RoKSxvcmRlcj11bmlxdWUob3JkZXIpKSUkJQ0KICBsbShsb2cobW91dGhfd2lkdGgpfmxvZyh0b3RhbF9sZW5ndGgpLC4pJT4lDQogIHN1bW1hcnkoKQ0KYGBgDQoNCiMjIENvbXBhcmlzb24gb2YgZXN0aW1hdGVzIHVzaW5nIFVKUCBhbmQgbW91dGggd2lkdGggaW4gYXJ0aHJvZGlyZXMNCg0KYGBge3J9DQpmaXQuVUpQPC1sbSh0b3RhbF9sZW5ndGh+VUpQLGxvd3J5X3NoYXJrcykNCnN1bW1hcnkoZml0LlVKUCkNCiMgUHJlZGljdGlvbnMgb2YgdG90YWwgbGVuZ3RoIHVzaW5nIFVKUCBhbmQgbW91dGggd2lkdGgNCm1vdXRoX3ByZWRpY3Rpb25zPC1mb3NzaWxfdGF4YV9tb3V0aHMlPiUNCiAgZHJvcF9uYShVSlAsbW91dGhfd2lkdGgpJT4lDQogIGF1Z21lbnQoeD1maXQuVUpQLG5ld2RhdGE9LixpbnRlcnZhbD0icHJlZGljdGlvbiIpICU+JSANCiAgcmVuYW1lX2F0KHZhcnMoc3RhcnRzX3dpdGgoJy4nKSksIGZ1bnMocGFzdGUwKCdmaXQuVUpQJywgLikpKSU+JQ0KICBhdWdtZW50KHg9Zml0Lm1vdXRod2lkdGgsbmV3ZGF0YT0uLGludGVydmFsPSJwcmVkaWN0aW9uIikgJT4lIA0KICByZW5hbWVfYXQodmFycyhzdGFydHNfd2l0aCgnLicpKSwgZnVucyhwYXN0ZTAoJ2ZpdC5tb3V0aF93aWR0aCcsIC4pKSklPiUNCiAgcmVuYW1lKG1vdXRoX3dpZHRoX29yaWdpbmFsPW1vdXRoX3dpZHRoLA0KICAgICAgICAgbW91dGhfd2lkdGg9aW5uZXJfbW91dGhfd2lkdGgpJT4lDQogIGF1Z21lbnQoeD1maXQubW91dGh3aWR0aCxuZXdkYXRhPS4sDQogICAgICAgICAgaW50ZXJ2YWw9InByZWRpY3Rpb24iKSAlPiUNCiAgcmVuYW1lX2F0KHZhcnMoc3RhcnRzX3dpdGgoJy4nKSksIGZ1bnMocGFzdGUwKCdmaXQuaW5uZXJfbW91dGhfd2lkdGgnLCAuKSkpJT4lDQogIHJlbmFtZShtb3V0aF93aWR0aD1tb3V0aF93aWR0aF9vcmlnaW5hbCwNCiAgICAgICAgIGlubmVyX21vdXRoX3dpZHRoPW1vdXRoX3dpZHRoKSU+JQ0KICBtdXRhdGUoYWNyb3NzKGZpdC5tb3V0aF93aWR0aC5maXR0ZWQ6Zml0LmlubmVyX21vdXRoX3dpZHRoLnVwcGVyLA0KICAgICAgICAgICAgICAgIH5leHAoLikqcmVncmVzc2lvbi5zdGF0cyhmaXQubW91dGh3aWR0aCkkQ0YpLA0KICAgICAgICAgZml0LlVKUC5yYW5nZT1wYXN0ZTAoIigiLHJvdW5kKGZpdC5VSlAubG93ZXIsMSksIuKAkyIscm91bmQoZml0LlVKUC51cHBlciwxKSwiKSIpLA0KICAgICAgICAgZml0Lm1vdXRoX3dpZHRoLnJhbmdlPXBhc3RlMCgiKCIscm91bmQoZml0Lm1vdXRoX3dpZHRoLmxvd2VyLDEpLCLigJMiLA0KICAgICAgICAgICAgICAgICAgICAgICAgICAgICAgICAgICAgICByb3VuZChmaXQubW91dGhfd2lkdGgudXBwZXIsMSksIikiKSwNCiAgICAgICAgIGZpdC5pbm5lcl9tb3V0aF93aWR0aC5yYW5nZT1wYXN0ZTAoIigiLHJvdW5kKGZpdC5pbm5lcl9tb3V0aF93aWR0aC5sb3dlciwxKSwi4oCTIiwNCiAgICAgICAgICAgICAgICAgICAgICAgICAgICAgICAgICAgICAgcm91bmQoZml0LmlubmVyX21vdXRoX3dpZHRoLnVwcGVyLDEpLCIpIikpDQojIEthYmxlIG9mIHJlc3VsdHMNCm1vdXRoX3ByZWRpY3Rpb25zJT4lDQogIGFycmFuZ2UoVUpQKSU+JQ0KICBtdXRhdGUoZGlmZl9wcmVkMT0xMDAqYWJzKGZpdC5VSlAuZml0dGVkLWZpdC5tb3V0aF93aWR0aC5maXR0ZWQpL2ZpdC5tb3V0aF93aWR0aC5maXR0ZWQsDQogICAgICAgICBkaWZmX3ByZWQyPTEwMCphYnMoZml0LlVKUC5maXR0ZWQtZml0LmlubmVyX21vdXRoX3dpZHRoLmZpdHRlZCkvZml0LmlubmVyX21vdXRoX3dpZHRoLmZpdHRlZCklPiUNCiAgc2VsZWN0KHRheG9uLHNwZWNpbWVuLFVKUCxmaXQuVUpQLmZpdHRlZCxmaXQuVUpQLnJhbmdlLA0KICAgICAgICAgbW91dGhfd2lkdGgsZml0Lm1vdXRoX3dpZHRoLmZpdHRlZCxmaXQubW91dGhfd2lkdGgucmFuZ2UsZGlmZl9wcmVkMSwNCiAgICAgICAgIGlubmVyX21vdXRoX3dpZHRoLGZpdC5pbm5lcl9tb3V0aF93aWR0aC5maXR0ZWQsZml0LmlubmVyX21vdXRoX3dpZHRoLnJhbmdlLGRpZmZfcHJlZDIpICU+JQ0KICBrYWJsZShkaWdpdHM9MiwNCiAgICAgICAgYWxpZ249YygibCIscmVwKCJjIiwxMSkpLA0KICAgICAgICBjb2wubmFtZXM9YygiVGF4b24iLCJTcGVjaW1lbiIsDQogICAgICAgICAgICAgICAgICAgICJVSlAiLCJFc3QuIiwiOTUlIEMuSS4iLA0KICAgICAgICAgICAgICAgICAgICAiTW91dGggV2lkdGgiLCJFc3QuIiwiOTUlIEMuSS4iLCIlIERpZi4iLA0KICAgICAgICAgICAgICAgICAgICAiSW5uZXIgTW91dGggV2lkdGgiLCJFc3QuIiwiOTUlIEMuSS4iLCIlIERpZi4iKSwNCiAgICAgICAgY2FwdGlvbj0iQ29tcGFyaXNvbiBvZiB0b3RhbCBsZW5ndGggZXN0aW1hdGVzIHVzaW5nIHVwcGVyIGphdyBwZXJpbWV0ZXIgYW5kIG1vdXRoIHdpZHRoLiBMZW5ndGggZXN0aW1hdGVzIHVzaW5nIGJvdGggYXJlIHJlbGF0aXZlbHkgc2ltaWxhciwgc3VnZ2VzdGluZyBtb3V0aCB3aWR0aCBpcyBhbiBhcHByb3ByaWF0ZSBhcHByb3hpbWF0aW9uIG9mIHVwcGVyIGphdyBwZXJpbWV0ZXIuIEFsbCBtZWFzdXJlbWVudHMgaW4gY20uIiklPiUNCiAgY29sdW1uX3NwZWMoMSwgaXRhbGljID0gVCklPiUNCiAgY29sdW1uX3NwZWMoYyg0LDcsMTEpLCBib2xkID0gVCklPiUNCiAgYWRkX2hlYWRlcl9hYm92ZShjKCIgIj0yLCJVcHBlciBKYXcgUGVyaW1ldGVyIj0zLCJNb3V0aCBXaWR0aCI9NCwiSW5uZXIgTW91dGggV2lkdGgiPTQpKSU+JQ0KICBrYWJsZV9zdHlsaW5nKCklPiUNCiAgc2Nyb2xsX2JveCh3aWR0aCA9ICIxMDAlIikNCmBgYA0KDQojIyBUZXN0aW5nIGFjY3VyYWN5IG9mIFVKUCBhbmQgbW91dGggd2lkdGggaW4gZXN0aW1hdGluZyB0b3RhbCBsZW5ndGggaW4gc2hhcmtzDQoNCmBgYHtyfQ0KZnNiY19zaGFya3MlPiUNCiAgbXV0YXRlKGZpdF91anA9cHJlZGljdChmaXQuVUpQLC4saW50ZXJ2YWw9InByZWRpY3Rpb24iKVssMV0sDQogICAgICAgICBsd3JfdWpwPXByZWRpY3QoZml0LlVKUCwuLGludGVydmFsPSJwcmVkaWN0aW9uIilbLDJdLA0KICAgICAgICAgdXByX3VqcD1wcmVkaWN0KGZpdC5VSlAsLixpbnRlcnZhbD0icHJlZGljdGlvbiIpWywzXSwNCiAgICAgICAgIFBFX3VqcD0oKGZpdF91anAtdG90YWxfbGVuZ3RoKS9maXRfdWpwKSoxMDAsDQogICAgICAgICBmaXRfbW91dGg9ZXhwKHByZWRpY3QoZml0Lm1vdXRod2lkdGgsLixpbnRlcnZhbD0icHJlZGljdGlvbiIpWywxXSksDQogICAgICAgICBsd3JfbW91dGg9ZXhwKHByZWRpY3QoZml0Lm1vdXRod2lkdGgsLixpbnRlcnZhbD0icHJlZGljdGlvbiIpWywyXSksDQogICAgICAgICB1cHJfbW91dGg9ZXhwKHByZWRpY3QoZml0Lm1vdXRod2lkdGgsLixpbnRlcnZhbD0icHJlZGljdGlvbiIpWywzXSksDQogICAgICAgICBQRV9tb3V0aD0oKGZpdF9tb3V0aC10b3RhbF9sZW5ndGgpL2ZpdF9tb3V0aCkqMTAwKSU+JQ0KICBjb2x1bW5fdG9fcm93bmFtZXMoInNwZWNpbWVuIiklPiUNCiAgc2VsZWN0KHRheG9uLHRvdGFsX2xlbmd0aCxmaXRfdWpwLGx3cl91anAsdXByX3VqcCxQRV91anAsZml0X21vdXRoLGx3cl9tb3V0aCx1cHJfbW91dGgsUEVfbW91dGgpJT4lDQogIG11dGF0ZShlc3RfZGlmZj0xMDAqYWJzKGZpdF91anAtZml0X21vdXRoKS9maXRfbW91dGgpJT4lDQogIGthYmxlKGRpZ2l0cz0xLA0KICAgICAgICBhbGlnbj1jKCJsIiwiYyIsImMiLCJjIiwiYyIsImMiLCJjIiwiYyIsImMiLCJjIiwiYyIpLA0KICAgICAgICBjYXB0aW9uPSJUZXN0aW5nIHRoZSBhY2N1cmFjeSBvZiB1cHBlciBqYXcgcGVyaW1ldGVyIGFuZCBtb3V0aCB3aWR0aCBpbiBlc3RpbWF0aW5nIHRoZSB0b3RhbCBsZW5ndGggb2Ygc2hhcmtzIG9mIGtub3duIGxlbmd0aC4gU3BlY2ltZW5zIG9mIDxpPlJoaXpvcHJpb25vZG9uIHRlcnJhbm92YWU8L2k+LCA8aT5DYXJjaGFyaGludXMgYWNyb25vdHVzPC9pPiwgYW5kIHRoZSB1bmNhdGFsb2dlZCBzcGVjaW1lbiBvZiA8aT5HYWxlb2NlcmRvIGN1dmllcmk8L2k+IHdlcmUgbWVhc3VyZWQgb2ZmIG9mIGV4dGVybmFsIG1lYXN1cmVtZW50cyBvZiBmbHVpZC1wcmVzZXJ2ZWQgc3BlY2ltZW5zLCB3aGVyZWFzIG1lYXN1cmVtZW50cyBvZiA8aT5DYXJjaGFyaGludXMgbGV1Y2FzPC9pPiBhbmQgYWxsIG90aGVyIHNwZWNpbWVucyBvZiA8aT5HYWxlb2NlcmRvPC9pPiB3ZXJlIG1hZGUgZnJvbSBkcmllZCBqYXdzLiBUb3RhbCBsZW5ndGggaW4gZmx1aWQtcHJlc2VydmVkIHNwZWNpbWVucyB3YXMgbWVhc3VyZWQgYXMgbmF0dXJhbCB0b3RhbCBsZW5ndGgsIHdoZXJlYXMgd2hldGhlciByZXBvcnRlZCB0b3RhbCBsZW5ndGggaW4gdGhlIGRyaWVkIGphd3MgcmVwcmVzZW50ZWQgbmF0dXJhbCBvciBzdHJldGNoZWQgdG90YWwgbGVuZ3RoIGNvdWxkIG5vdCBiZSBjb25maXJtZWQuIiwNCiAgICAgICAgY29sLm5hbWVzID0gYygiVGF4b24iLCJUb3RhbCBMZW5ndGgiLCJFc3QuIiwiTG93ZXIiLCJVcHBlciIsIlBFIiwiRXN0LiIsIkxvd2VyIiwiVXBwZXIiLCJQRSIsIlBlcmNlbnQgRGlmZmVyZW5jZSIpKSU+JQ0KICBjb2x1bW5fc3BlYygyLCBpdGFsaWMgPSBUKSU+JQ0KICBjb2x1bW5fc3BlYyhjKDMsNCw4KSwgYm9sZCA9IFQpJT4lDQogIGFkZF9oZWFkZXJfYWJvdmUoYygiICI9MywiVXBwZXIgSmF3IFBlcmltZXRlciI9NCwiTW91dGggV2lkdGgiPTQsIiAiPTEpKSU+JQ0KICBrYWJsZV9zdHlsaW5nKCkNCmBgYA0KDQpCYXNlZCBvbiB0aGlzLCBpdCBzZWVtcyB0aGF0IGBVSlBgIGFuZCBgbW91dGhfd2lkdGhgIGNhcHR1cmUgc2ltaWxhciBlbm91Z2ggbW9ycGhvbWV0cmljIHNpZ25hbCB0aGF0IGBtb3V0aF93aWR0aGAgY2FuIGJlIGNvbnNpZGVyZWQgYW4gYXBwcm9wcmlhdGUgc3Vic3RpdHV0ZSBmb3IgdGVzdGluZyB3aGV0aGVyIGBVSlBgIGluIHNoYXJrcyBhY2N1cmF0ZWx5IHByZWRpY3RzIGJvZHkgc2l6ZSBpbiBhcnRocm9kaXJlcyAoZXNwZWNpYWxseSBnaXZlbiBVSlAgaXMgdW5hdmFpbGFibGUgZm9yIHNtYWxsZXIgc2hhcmtzKS4gVGhlIHR3byB2YXJpYWJsZXMgZGlzYWdyZWUgaW4gdGhlIGxlbmd0aCBlc3RpbWF0ZXMgb2YgRlNCQyAxODA4MywgYnV0IHRoaXMgaXMgbGlrZWx5IGJlY2F1c2UgdGhpcyBzcGVjaW1lbiBwZXJ0YWlucyB0byBhIHZlcnkgc21hbGwgKHBvc3NpYmx5IG5lb25hdGFsKSAqQ2FyY2hhcmhpbnVzIGxldWNhcyogYW5kIGlzIG91dHNpZGUgdGhlIHJhbmdlIG9mIHZhbHVlcyBvZiB0aGUgZGF0YXNldCBvZiBMb3dyeSBldCBhbC4gKDIwMDkpLg0KDQojIyBVc2luZyAqQnJhY2h5cGxhdHlzdG9tYSogYXMgYSBDYXNlIFN0dWR5DQoNCmBgYHtyfQ0KZGF0YV9tb3V0aHMlPiUNCiAgZmlsdGVyKGdlbnVzPT0iQnJhY2h5cGxhdHlzdG9tYSIpJT4lDQogIGF1Z21lbnQobmV3ZGF0YT0uLGZpdC5VSlApJT4lDQogIHJlbmFtZShmaXQxLmZpdHRlZD0uZml0dGVkKSU+JQ0KICBhdWdtZW50KG5ld2RhdGE9LixmaXQubW91dGh3aWR0aCklPiUNCiAgcmVuYW1lKGZpdDIuZml0dGVkPS5maXR0ZWQpJT4lDQogIG11dGF0ZShmaXQyLmZpdHRlZD1leHAoZml0Mi5maXR0ZWQpKnJlZ3Jlc3Npb24uc3RhdHMoZml0Lm1vdXRod2lkdGgpJENGKSU+JQ0KICBtdXRhdGUoZXJyb3IxPTEwMCooKGZpdDEuZml0dGVkLXRvdGFsX2xlbmd0aCkvdG90YWxfbGVuZ3RoKSwNCiAgICAgICAgIGVycm9yMj0xMDAqKChmaXQyLmZpdHRlZC10b3RhbF9sZW5ndGgpL3RvdGFsX2xlbmd0aCkpJT4lDQogIHNlbGVjdCh0YXhvbixzcGVjaW1lbix0b3RhbF9sZW5ndGgsZml0MS5maXR0ZWQsZXJyb3IxLGZpdDIuZml0dGVkLGVycm9yMiklPiUNCiAga2FibGUoZGlnaXRzPTIsYWxpZ249YygibCIsImMiLCJjIiwiYyIsImMiLCJjIiwiYyIpLA0KICAgICAgICBjb2wubmFtZXMgPSBjKCJUYXhvbiIsIlNwZWNpbWVuIiwiQWN0dWFsIiwiRXN0aW1hdGVkIiwiJSBEaWZmZXJlbmNlIGZyb20gQWN0dWFsIFZhbHVlIiwiRXN0aW1hdGVkIiwiJSBEaWZmZXJlbmNlIGZyb20gQWN0dWFsIFZhbHVlIiksDQogICAgICAgIGNhcHRpb249IkVzdGltYXRlZCB0b3RhbCBsZW5ndGggaW4gPGk+QnJhY2h5cGxhdHlzdG9tYSBjYXBhcHJldHVtPC9pPiB1c2luZyB1cHBlciBqYXcgcGVyaW1ldGVyLiBBbGwgbWVhc3VyZW1lbnRzIGluIGNtLiIpJT4lDQogIGNvbHVtbl9zcGVjKDEsIGl0YWxpYyA9IFQpJT4lDQogIGFkZF9oZWFkZXJfYWJvdmUoYygiICI9MywiVUpQIj0yLCJNb3V0aCBXaWR0aCI9MikpJT4lDQogIGthYmxlX3N0eWxpbmcoKQ0KYGBgDQoNCkJ5IHVzaW5nICpCcmFjaHlwbGF0eXN0b21hIGNhcGFwcmV0dW0qLCBhIGxhcmdlLCBuZWt0b25pYyBmaXNoIHdpdGggYSBzdXBlcmZpY2lhbGx5IHNoYXJrLWxpa2UgYm9keSBzaGFwZSwgYXMgYSBjYXNlIHN0dWR5LCBpdCBjYW4gYmUgc2VlbiB0aGF0IFVKUCBmYWlscyB0byBhY2N1cmF0ZWx5IGVzdGltYXRlIGxlbmd0aCBpbiB0aGlzIHRheG9uLCBhbmQgaW4gZmFjdCBzaWduaWZpY2FudGx5IG92ZXItZXN0aW1hdGVzIGl0cyBib2R5IHNpemUgKHRoYXQgaXMsICpCLiBjYXBhcHJldHVtKiBoYXMgYSBtdWNoIGxhcmdlciBtb3V0aCB0aGFuIGV4cGVjdGVkIGFzc3VtaW5nIHNoYXJrLWxpa2UgcHJvcG9ydGlvbnMpLiBUaGlzIHN1Z2dlc3RzIHRoYXQgYFVKUGAgb3IgYG1vdXRoX3dpZHRoYCBtYXkgbm90IGV4aGliaXQgYSBzaW5nbGUgYWxsb21ldHJpYyByZWxhdGlvbnNoaXAgd2l0aCB0b3RhbCBsZW5ndGggYWNyb3NzIGFsbCBmaXNoZXMsIHBhcnRpY3VsYXJseSBub24tc2hhcmtzIHN1Y2ggYXMgKkJyYWNoeXBsYXR5c3RvbWEqIChTaWx1cmlmb3JtZXM6IFBpbWVsb2RpZGFlKSBvciBhcnRocm9kaXJlcy4NCg0KIyBVc2luZyBtb3V0aCB3aWR0aCB0byBlc3RpbWF0ZSB0b3RhbCBsZW5ndGggaW4gYXJ0aHJvZGlyZXMNCg0KIyMgTW91dGggd2lkdGggdmVyc3VzIHRvdGFsIGxlbmd0aCBpbiBjaG9uZHJpY2h0aHlhbnMgKGFuZCBhcnRocm9kaXJlcykNCg0KKHJlZjptb3V0aHdpZHRoKSBQbG90IG9mIGxvZzEwIG1vdXRoIHdpZHRoIHZlcnN1cyB0b3RhbCBsZW5ndGggaW4gZmlzaGVzLCBmb2N1c2luZyBvbiB0aGUgZGlmZmVyZW5jZSBiZXR3ZWVuIHNoYXJrcyAoZmlsbGVkIHJlY3RhbmdsZXMpIGFuZCBhcnRocm9kaXJlcyAoc3RhcnMpLiBCbGFjayBzdGFycyByZXByZXNlbnRzIGFydGhyb2RpcmVzIHdpdGgga25vd24gdG90YWwgbGVuZ3RocyBhbmQgbW91dGggd2lkdGhzLCB3aGVyZWFzIHRoZSB5ZWxsb3cgc3RhcnMgcmVwcmVzZW50cyAqRHVua2xlb3N0ZXVzIHRlcnJlbGxpKiB3aXRoIGVzdGltYXRlZCBsZW5ndGhzIGZyb20gRmVycsOzbiBldCBhbC4gKDIwMTcpIGNvbXBhcmVkIHdpdGggZGlyZWN0bHkgbWVhc3VyZWQgbW91dGggd2lkdGhzIG9mIHRoZSBzYW1lIHNwZWNpbWVucy4gTWFnZW50YSBzdGFycyBhcmUgc3BlY2ltZW5zIG9mICpBbWF6aWNodGh5cyB0cmluYWpzdGljYWUqIGFuZCBhbiB1bmRlc2NyaWJlZCBhc3Bpbm90aG9yYWNpZCBhdCB0aGUgQ01OSCBpbiB3aGljaCB0aGUgbW91dGggd2lkdGggY2Fubm90IGJlIG1lYXN1cmVkLCBidXQgY2FuIGJlIGFwcHJveGltYXRlZCBiYXNlZCBvbiBza3VsbCB3aWR0aC4gT3JhbmdlIHNxdWFyZXMgcmVwcmVzZW50cyB0aGUgZ2VuZXJhICpQbGFub25hc3VzKiwgKkNlcGhhbG9zY3lsbGl1bSogYW5kICpJc2lzdGl1cyosIHdoaWNoIGhhdmUgdW51c3VhbGx5IHdpZGUgbW91dGhzIGFtb25nIHNoYXJrcy4gR3JlZW4gc3F1YXJlcyByZXByZXNlbnRzIHRoZSB0aHJlc2hlciBzaGFyayAqQWxvcGlhcyosIHdoaWNoIGhhcyBhIG5hcnJvd2VyIG1vdXRoIHJlbGF0aXZlIHRvIHRvdGFsIGxlbmd0aCBiZWNhdXNlIG9mIGl0cyBlbG9uZ2F0ZSBjYXVkYWwgZmluLg0KDQpgYGB7cixmaWcuY2FwPSIocmVmOm1vdXRod2lkdGgpIn0NCihtb3V0aF93aWR0aF9wbG90PC1kYXRhX21vdXRocyU+JQ0KICBkcm9wX25hKG1vdXRoX3dpZHRoKSU+JQ0KICBhdWdtZW50KGZpdC5tb3V0aHdpZHRoLG5ld2RhdGE9LixpbnRlcnZhbD0icHJlZGljdGlvbiIpJT4lDQogIG11dGF0ZShhY3Jvc3MoLmZpdHRlZDoudXBwZXIsfmV4cCguKSpyZWdyZXNzaW9uLnN0YXRzKGZpdC5tb3V0aHdpZHRoKSRDRiksDQogICAgICAgICB0b3RhbF9sZW5ndGg9aWZlbHNlKGdlbnVzPT0iRHVua2xlb3N0ZXVzIiwNCiAgICAgICAgICAgICAgICAgICAgICAgICAgICAgcHJlZGljdChmaXQuVUpQLC4pLHRvdGFsX2xlbmd0aCksDQogICAgICAgICBjbGFkZTI9Y2FzZV93aGVuKGdlbnVzPT0iRHVua2xlb3N0ZXVzIn4iRHVua2xlb3N0ZXVzIiwNCiAgICAgICAgICAgICAgICAgICAgICAgICAgZ2VudXMgJWluJSBjKCJBbWF6aWNodGh5cyIsIk5ld3NwZWNpZXMiKX4iRXN0LiBGcm9tIFNrdWxsIiwNCiAgICAgICAgICAgICAgICAgICAgICAgICAgY2xhZGU9PSJQbGFjb2Rlcm1pIn4iT3RoZXIgUGxhY29kZXJtaSIsDQogICAgICAgICAgICAgICAgICAgICAgICAgIGdlbnVzPT0iQWxvcGlhcyJ+IkFsb3BpYXMiLA0KICAgICAgICAgICAgICAgICAgICAgICAgICBnZW51cz09IkNsYWRvc2VsYWNoZSJ+IkNsYWRvc2VsYWNoZSIsDQogICAgICAgICAgICAgICAgICAgICAgICAgIGdlbnVzICVpbiUgYygiUGxhbm9uYXN1cyIsIkNlcGhhbG9zY3lsbGl1bSIsIklzaXN0aXVzIil+DQogICAgICAgICAgICAgICAgICAgICAgICAgICAgICAgICAgICJQbGFub25hc3VzICsgQ2VwaGFsb3NjeWxsaXVtIiwNCiAgICAgICAgICAgICAgICAgICAgICAgICAgY2xhZGU9PSJDaG9uZHJpY2h0aHllcyJ+Ik90aGVyIENob25kcmljaHRoeWVzIikpJT4lDQogIGZpbHRlcihjbGFkZSAlaW4lIGMoIlBsYWNvZGVybWkiLCJDaG9uZHJpY2h0aHllcyIpLA0KICAgICAgICAgIWlzLm5hKHRvdGFsX2xlbmd0aCl8Z2VudXM9PSJEdW5rbGVvc3RldXMiKSU+JQ0KICBnZ3Bsb3QoLixhZXModG90YWxfbGVuZ3RoLG1vdXRoX3dpZHRoKSkrDQogIHNjYWxlX3hfY29udGludW91cyh0cmFucz0ibG9nMTAiKSArIHNjYWxlX3lfY29udGludW91cyh0cmFucz0ibG9nMTAiKSsNCiAgZ2VvbV9zdGFyKGFlcyhzdGFyc2hhcGU9Y2xhZGUyLGZpbGw9Y2xhZGUyKSxzaXplPTIpKw0KICBzY2FsZV9zdGFyc2hhcGVfbWFudWFsKHZhbHVlcz1jKDEzLDEzLDEzLDEzLDEsMSwxKSwNCiAgICAgICAgICAgICAgICAgICAgICAgICBsYWJlbHM9YygiT3RoZXIgQ2hvbmRyaWNodGh5ZXMiLCIqQWxvcGlhcyoiLCIqQ2xhZG9zZWxhY2hlKiIsDQogICAgICAgICAgICAgICAgICAgICAgICAgICAgICIqUGxhbm8uL0NlcGhhbG8uL0lzaXN0LioiLA0KICAgICAgICAgICAgICAgICAgICAgICAgICAgICAiKkR1bmtsZW9zdGV1cyogKEVzdC4pIiwiKkFtYXppY2h0aHlzKi9DTU5IIEFzcGluLiIsIk90aGVyIFBsYWNvZGVybWkiKSwNCiAgICAgICAgICAgICAgICAgICAgICAgICBicmVha3M9YygiT3RoZXIgQ2hvbmRyaWNodGh5ZXMiLCJBbG9waWFzIiwNCiAgICAgICAgICAgICAgICAgICAgICAgICAgICAgICAgICAiQ2xhZG9zZWxhY2hlIiwNCiAgICAgICAgICAgICAgICAgICAgICAgICAgICAgICAgICAiUGxhbm9uYXN1cyArIENlcGhhbG9zY3lsbGl1bSIsDQogICAgICAgICAgICAgICAgICAgICAgICAgICAgICAgICAgIkR1bmtsZW9zdGV1cyIsIkVzdC4gRnJvbSBTa3VsbCIsIk90aGVyIFBsYWNvZGVybWkiKSkrDQogIHNjYWxlX2ZpbGxfbWFudWFsKHZhbHVlcz1jKCJncmF5IiwiZ3JlZW4iLCJibGFjayIsIm9yYW5nZSIsInllbGxvdyIsIm1hZ2VudGEiLCJibGFjayIpLA0KICAgICAgICAgICAgICAgICAgICBicmVha3M9YygiT3RoZXIgQ2hvbmRyaWNodGh5ZXMiLCJBbG9waWFzIiwiQ2xhZG9zZWxhY2hlIiwNCiAgICAgICAgICAgICAgICAgICAgICAgICAgICAgIlBsYW5vbmFzdXMgKyBDZXBoYWxvc2N5bGxpdW0iLA0KICAgICAgICAgICAgICAgICAgICAgICAgICAgICAiRHVua2xlb3N0ZXVzIiwiRXN0LiBGcm9tIFNrdWxsIiwiT3RoZXIgUGxhY29kZXJtaSIpLA0KICAgICAgICAgICAgICAgICAgICBuYS52YWx1ZT1OQSwNCiAgICAgICAgICAgICAgICAgICAgbGFiZWxzPWMoIk90aGVyIENob25kcmljaHRoeWVzIiwiKkFsb3BpYXMqIiwiKkNsYWRvc2VsYWNoZSoiLA0KICAgICAgICAgICAgICAgICAgICAgICAgICAgICAiKlBsYW5vLi9DZXBoYWxvLi9Jc2lzdC4qIiwNCiAgICAgICAgICAgICAgICAgICAgICAgICAgICAgIipEdW5rbGVvc3RldXMqIChFc3QuKSIsIipBbWF6aWNodGh5cyovQ01OSCBBc3Bpbi4iLCJPdGhlciBQbGFjb2Rlcm1pIikpKw0KICBnZW9tX3NlZ21lbnQoZGF0YT0uJT4lZmlsdGVyKGdlbnVzICVpbiUgYygiQ29jY29zdGV1cyIsIlBsb3VyZG9zdGV1cyIsIkluY2lzb3NjdXR1bSIpKSwNCiAgICAgICAgICAgICAgIGFlcyh4ZW5kPXRvdGFsX2xlbmd0aCowLjgseWVuZD1tb3V0aF93aWR0aCoxLjMpKSsNCiAgZ2VvbV9zZWdtZW50KGRhdGE9LiU+JWZpbHRlcihzcGVjaW1lbj09IkNNTkggNTc2OCIpLA0KICAgICAgICAgICAgICAgYWVzKHhlbmQ9dG90YWxfbGVuZ3RoLHllbmQ9bW91dGhfd2lkdGgqMC40KSkrDQogIGdlb21fc3RhcihkYXRhPS4lPiVmaWx0ZXIoY2xhZGU9PSJQbGFjb2Rlcm1pIiksDQogICAgICAgICAgICBhZXMoc3RhcnNoYXBlPWNsYWRlMixmaWxsPWNsYWRlMiksc2l6ZT0zLjUsc2hvdy5sZWdlbmQ9RikrDQogIGdlb21fbGluZShhZXMoeD0uZml0dGVkKSwgY29sb3IgPSAiIzMzNjZGRiIsc2l6ZT0xLjI1LHNob3cubGVnZW5kPUYpKw0KICBnZW9tX2xpbmUoYWVzKHg9Lmxvd2VyKSwgY29sb3IgPSAiIzMzNjZGRiIsIGxpbmV0eXBlID0gImRhc2hlZCIsc2hvdy5sZWdlbmQ9RikrDQogIGdlb21fbGluZShhZXMoeD0udXBwZXIpLCBjb2xvciA9ICIjMzM2NkZGIiwgbGluZXR5cGUgPSAiZGFzaGVkIixzaG93LmxlZ2VuZD1GKSsNCiAgZ2VvbV9zdGFyKGRhdGE9LiU+JWZpbHRlcihjbGFkZT09IlBsYWNvZGVybWkiLGNsYWRlMj09IkVzdC4gRnJvbSBTa3VsbCIpLHNpemU9My41LA0KICAgICAgICAgICAgY29sb3I9IndoaXRlIixhZXMoc3RhcnNoYXBlPWNsYWRlMixmaWxsPWNsYWRlMiksc3RhcnN0cm9rZT0xLHNob3cubGVnZW5kPUYpKw0KICAgZ2VvbV9zdGFyKGRhdGE9LiU+JWZpbHRlcihjbGFkZT09IlBsYWNvZGVybWkiLGNsYWRlMj09IkVzdC4gRnJvbSBTa3VsbCIpLHNpemU9MywNCiAgICAgICAgICAgIGNvbG9yPSJibGFjayIsYWVzKHN0YXJzaGFwZT1jbGFkZTIsZmlsbD1jbGFkZTIpLHN0YXJzdHJva2U9MC41LHNob3cubGVnZW5kPUYpKw0KICBnZW9tX3N0YXIoZGF0YT0uJT4lZmlsdGVyKGNsYWRlPT0iUGxhY29kZXJtaSIsY2xhZGUyPT0iT3RoZXIgUGxhY29kZXJtaSIpLHNpemU9My41LA0KICAgICAgICAgICAgc3RhcnNoYXBlPTEsY29sb3I9IndoaXRlIixmaWxsPSJibGFjayIsc3RhcnN0cm9rZT0wLjUsc2hvdy5sZWdlbmQ9RikrDQogIGdlb21fdGV4dChkYXRhPS4lPiVmaWx0ZXIoZ2VudXMgJWluJSBjKCJDb2Njb3N0ZXVzIiwiUGxvdXJkb3N0ZXVzIiwiSW5jaXNvc2N1dHVtIikpLHNpemU9My41LA0KICAgICAgICAgICBhZXMoeD0wLjkqdG90YWxfbGVuZ3RoKmlmZWxzZShnZW51cz09IkNvY2Nvc3RldXMiLC44NSwxKSwNCiAgICAgICAgICAgICAgIHk9MS4zNjUqbW91dGhfd2lkdGgqaWZlbHNlKGdlbnVzPT0iQ29jY29zdGV1cyIsLjk1LDEpLA0KICAgICAgICAgICAgICAgbGFiZWw9Z2VudXMpLGhqdXN0PTEsdmp1c3Q9MCxmb250ZmFjZT0zKSsNCiAgZ2VvbV90ZXh0KGRhdGE9LiU+JWZpbHRlcihzcGVjaW1lbj09IkNNTkggNTc2OCIpLHNpemU9My41LA0KICAgICAgICAgICAgYWVzKHk9MC4zNSptb3V0aF93aWR0aCxsYWJlbD1nZW51cyksaGp1c3Q9MC41LHZqdXN0PTAsZm9udGZhY2U9MykrDQogIGxhYnMoeD0iVG90YWwgTGVuZ3RoIChjbSkiLHk9Ik1vdXRoIFdpZHRoIChjbSkiLHN0YXJzaGFwZT0iR3JvdXAiLGZpbGw9Ikdyb3VwIikrDQogIHRoZW1lX2NsYXNzaWMoKSsNCiAgdGhlbWUobGVnZW5kLnBvc2l0aW9uPWMoMC44LDAuMjUpLA0KICAgICAgICBsZWdlbmQudGV4dD1lbGVtZW50X21hcmtkb3duKCkpKQ0KZ2dzYXZlKCJBcnRocm9kaXJlIE1vdXRocyBGaWd1cmUgMyAoTW91dGggV2lkdGgpLnBkZiIsbW91dGhfd2lkdGhfcGxvdCwNCiAgICAgICAicGRmIix3aWR0aD0xODAsaGVpZ2h0PTEzNSx1bml0cz0ibW0iKQ0KYGBgDQoNCkJhc2VkIG9uIHRoaXMsIGl0IGlzIGNsZWFyIHRoYXQgaW4gYWxsIHRoZSBhcnRocm9kaXJlcyBmb3Igd2hpY2ggYm9keSBsZW5ndGggaXMgZGlyZWN0bHkgbWVhc3VyYWJsZSB0aGF0IGFydGhyb2RpcmVzIGhhdmUgbXVjaCB3aWRlciBtb3V0aHMgdGhhbiBzaGFya3MgYXQgc2ltaWxhciBib2R5IHNpemVzLCBpbiBtb3N0IGNhc2VzIG91dHNpZGUgdGhlIDk1JSBwcmVkaWN0aW9uIGludGVydmFsIGZvciBzaGFya3MuIFRoZSBvbmx5IHNoYXJrcyB0aGF0IHBsb3QgY2xvc2UgdG8gYXJ0aHJvZGlyZXMgYXJlICpDZXBoYWxvc2N5bGxpdW0qLCAqUGxhbm9uYXN1cyosIGFuZCBhIGZldyBkZWVwLXNlYSBzcXVhbG9pZHMgKGUuZy4sICpJc2lzdGl1cyopIHRoYXQgaGF2ZSBub3RhYmx5IGxhcmdlIG1vdXRocyByZWxhdGl2ZSB0byBib2R5IHNpemUgYW1vbmcgc2hhcmtzLiBUaGlzIG1lYW5zIHRoYXQgbW91dGggZGltZW5zaW9ucyBhcmUgbm90IGV4cGVjdGVkIHRvIGFjY3VyYXRlbHkgcHJlZGljdCBib2R5IHNpemUgaW4gYXJ0aHJvZGlyZXMuIFRoZSBlc3RpbWF0ZWQgbGVuZ3RocyBmcm9tIEZlcnLDs24gYXJlIHdpdGhpbiB0aGUgOTUlIHByZWRpY3Rpb24gaW50ZXJ2YWwgZm9yIHNoYXJrcyAoYW5kIGluZGVkIHByZWRpY3QgYSBzbWFsbGVyLXRoYW4tYXZlcmFnZSBtb3V0aCBmb3IgKkR1bmtsZW9zdGV1cyogY29tcGFyZWQgdG8gc2hhcmtzKSwgYnV0IGJlY2F1c2UgdGhlc2UgbGVuZ3RocyBhcmUgZXN0aW1hdGVkIGJhc2VkIG9uIGEgcmVncmVzc2lvbiBtb2RlbCBkZXJpdmVkIGZyb20gc2hhcmtzDQoNClRoZSB0d28gc3RhcnMgd2hpY2ggYXJlIGNsb3NlciB0byB0aGUgcmVncmVzc2lvbiBsaW5lIGZvcm1lZCBieSBzaGFya3MgYXJlICpBbWF6aWNodGh5cyB0cmluamFzdGljYWUqIGFuZCBhbiB1bmRlc2NyaWJlZCBhcnRocm9kaXJlIChwb3NzaWJseSBhbiBhc3Bpbm90aG9yYWNpZCkgaW4gdGhlIGNvbGxlY3Rpb25zIG9mIHRoZSBDbGV2ZWxhbmQgTXVzZXVtIG9mIE5hdHVyYWwgSGlzdG9yeS4gSG93ZXZlciwgbmVpdGhlciBvZiB0aGVzZSB0d28gc3BlY2ltZW5zIHByZXNlcnZlcyB0aGUgbW91dGgsIGluIGJvdGggY2FzZXMgdGhlIG1vdXRoIHdpZHRoIGlzIGV4dHJhcG9sYXRlZCBmcm9tIHNrdWxsIHJvb2Ygd2lkdGggYXNzdW1pbmcgYSByZWxhdGlvbnNoaXAgYmV0d2VlbiB0aGUgdHdvIHNpbWlsYXIgdG8gb3RoZXIgYXJ0aG9kaXJlcy4gVGhpcyBpcyBub3QgdGhlIGlkZWFsIHdheSBvZiBkZXRlcm1pbmluZyBtb3V0aCBzaXplIGluIHRoZXNlIHRheGEsIGJ1dCBpdCdzIHdvcnRoIG5vdGluZyBldmVuIGRvaW5nIHRoaXMgcmVzdWx0cyBpbiBtb3V0aHMgdGhhdCBhcmUgb24gdGhlIGVkZ2Ugb2YgdGhlIHVwcGVyIGJvdW5kIG9mIHRoZSA5NSUgcHJlZGljdGlvbiBpbnRlcnZhbCBpbiBzaGFya3MuDQoNCiMjIFRlc3RpbmcgdXNpbmcgaW5uZXIgbW91dGggd2lkdGggaW4gYXJ0aHJvZGlyZXMNCg0KKHJlZjppbm5lcm1vdXRocGxvdCkgUGxvdCBvZiBsb2cxMCBtb3V0aCB3aWR0aCB2ZXJzdXMgdG90YWwgbGVuZ3RoIGluIGZpc2hlcywgdXNpbmcgYGlubmVyX21vdXRoIHdpZHRoYCBpbiBhcnRocm9kaXJlcyBmb3Igd2hpY2ggdGhpcyB2YWx1ZSBjYW4gYmUgbWVhc3VyZWQuIA0KDQpgYGB7cixmaWcuY2FwPSIocmVmOmlubmVybW91dGhwbG90KSJ9DQpkYXRhX21vdXRocyU+JQ0KICBkcm9wX25hKG1vdXRoX3dpZHRoKSU+JQ0KICBtdXRhdGUobW91dGhfd2lkdGg9aWZlbHNlKGNsYWRlPT0iUGxhY29kZXJtaSIsaW5uZXJfbW91dGhfd2lkdGgsbW91dGhfd2lkdGgpKSU+JQ0KICBhdWdtZW50KGZpdC5tb3V0aHdpZHRoLG5ld2RhdGE9LixpbnRlcnZhbD0icHJlZGljdGlvbiIpJT4lDQogIG11dGF0ZShhY3Jvc3MoLmZpdHRlZDoudXBwZXIsfmV4cCguKSpyZWdyZXNzaW9uLnN0YXRzKGZpdC5tb3V0aHdpZHRoKSRDRiksDQogICAgICAgICB0b3RhbF9sZW5ndGg9aWZlbHNlKGdlbnVzPT0iRHVua2xlb3N0ZXVzIiwNCiAgICAgICAgICAgICAgICAgICAgICAgICAgICAgcHJlZGljdChmaXQuVUpQLC4pLHRvdGFsX2xlbmd0aCksDQogICAgICAgICBjbGFkZTI9Y2FzZV93aGVuKGdlbnVzPT0iRHVua2xlb3N0ZXVzIn4iRHVua2xlb3N0ZXVzIiwNCiAgICAgICAgICAgICAgICAgICAgICAgICAgZ2VudXMgJWluJSBjKCJBbWF6aWNodGh5cyIsIk5ld3NwZWNpZXMiKX4iRXN0LiBGcm9tIFNrdWxsIiwNCiAgICAgICAgICAgICAgICAgICAgICAgICAgY2xhZGU9PSJQbGFjb2Rlcm1pIn4iT3RoZXIgUGxhY29kZXJtaSIsDQogICAgICAgICAgICAgICAgICAgICAgICAgIGdlbnVzPT0iQWxvcGlhcyJ+IkFsb3BpYXMiLA0KICAgICAgICAgICAgICAgICAgICAgICAgICBnZW51cz09IkNsYWRvc2VsYWNoZSJ+IkNsYWRvc2VsYWNoZSIsDQogICAgICAgICAgICAgICAgICAgICAgICAgIGdlbnVzICVpbiUgYygiUGxhbm9uYXN1cyIsIkNlcGhhbG9zY3lsbGl1bSIsIklzaXN0aXVzIil+DQogICAgICAgICAgICAgICAgICAgICAgICAgICAgIlBsYW5vbmFzdXMgKyBDZXBoYWxvc2N5bGxpdW0iLA0KICAgICAgICAgICAgICAgICAgICAgICAgICBjbGFkZT09IkNob25kcmljaHRoeWVzIn4iT3RoZXIgQ2hvbmRyaWNodGh5ZXMiKSklPiUNCiAgZmlsdGVyKGNsYWRlICVpbiUgYygiUGxhY29kZXJtaSIsIkNob25kcmljaHRoeWVzIiksDQogICAgICAgICAhaXMubmEodG90YWxfbGVuZ3RoKXxnZW51cz09IkR1bmtsZW9zdGV1cyIpJT4lDQogIGdncGxvdCguLGFlcyh0b3RhbF9sZW5ndGgsbW91dGhfd2lkdGgpKSsNCiAgc2NhbGVfeF9jb250aW51b3VzKHRyYW5zPSJsb2cxMCIpICsgc2NhbGVfeV9jb250aW51b3VzKHRyYW5zPSJsb2cxMCIpKw0KICBnZW9tX3N0YXIoYWVzKHN0YXJzaGFwZT1jbGFkZTIsZmlsbD1jbGFkZTIpLHNpemU9MikrDQogIHNjYWxlX3N0YXJzaGFwZV9tYW51YWwodmFsdWVzPWMoMTMsMTMsMTMsMTMsMSwxLDEpLA0KICAgICAgICAgICAgICAgICAgICAgICAgIGxhYmVscz1jKCJPdGhlciBDaG9uZHJpY2h0aHllcyIsIipBbG9waWFzKiIsIipDbGFkb3NlbGFjaGUqIiwNCiAgICAgICAgICAgICAgICAgICAgICAgICAgICAgICAgICAiKlBsYW5vLi9DZXBoYWxvLi9Jc2lzdC4qIiwNCiAgICAgICAgICAgICAgICAgICAgICAgICAgICAgICAgICAiKkR1bmtsZW9zdGV1cyogKEVzdC4pIiwiKkFtYXppY2h0aHlzKi9DTU5IIEFzcGluLiIsIk90aGVyIFBsYWNvZGVybWkiKSwNCiAgICAgICAgICAgICAgICAgICAgICAgICBicmVha3M9YygiT3RoZXIgQ2hvbmRyaWNodGh5ZXMiLCJBbG9waWFzIiwNCiAgICAgICAgICAgICAgICAgICAgICAgICAgICAgICAgICAiQ2xhZG9zZWxhY2hlIiwNCiAgICAgICAgICAgICAgICAgICAgICAgICAgICAgICAgICAiUGxhbm9uYXN1cyArIENlcGhhbG9zY3lsbGl1bSIsDQogICAgICAgICAgICAgICAgICAgICAgICAgICAgICAgICAgIkR1bmtsZW9zdGV1cyIsIkVzdC4gRnJvbSBTa3VsbCIsIk90aGVyIFBsYWNvZGVybWkiKSkrDQogIHNjYWxlX2ZpbGxfbWFudWFsKHZhbHVlcz1jKCJncmF5IiwiZ3JlZW4iLCJibGFjayIsIm9yYW5nZSIsInllbGxvdyIsImRhcmtjeWFuIiwiYmxhY2siKSwNCiAgICAgICAgICAgICAgICAgICAgYnJlYWtzPWMoIk90aGVyIENob25kcmljaHRoeWVzIiwiQWxvcGlhcyIsIkNsYWRvc2VsYWNoZSIsDQogICAgICAgICAgICAgICAgICAgICAgICAgICAgICJQbGFub25hc3VzICsgQ2VwaGFsb3NjeWxsaXVtIiwNCiAgICAgICAgICAgICAgICAgICAgICAgICAgICAgIkR1bmtsZW9zdGV1cyIsIkVzdC4gRnJvbSBTa3VsbCIsIk90aGVyIFBsYWNvZGVybWkiKSwNCiAgICAgICAgICAgICAgICAgICAgbmEudmFsdWU9TkEsDQogICAgICAgICAgICAgICAgICAgIGxhYmVscz1jKCJPdGhlciBDaG9uZHJpY2h0aHllcyIsIipBbG9waWFzKiIsIipDbGFkb3NlbGFjaGUqIiwNCiAgICAgICAgICAgICAgICAgICAgICAgICAgICAgIipQbGFuby4vQ2VwaGFsby4vSXNpc3QuKiIsDQogICAgICAgICAgICAgICAgICAgICAgICAgICAgICIqRHVua2xlb3N0ZXVzKiAoRXN0LikiLCIqQW1hemljaHRoeXMqL0NNTkggQXNwaW4uIiwiT3RoZXIgUGxhY29kZXJtaSIpKSsNCiAgZ2VvbV9zZWdtZW50KGRhdGE9LiU+JWZpbHRlcihnZW51cyAlaW4lIGMoIkNvY2Nvc3RldXMiLCJQbG91cmRvc3RldXMiLCJJbmNpc29zY3V0dW0iKSksDQogICAgICAgICAgICAgICBhZXMoeGVuZD10b3RhbF9sZW5ndGgqMC44LHllbmQ9bW91dGhfd2lkdGgqMS4zKSkrDQogIGdlb21fc2VnbWVudChkYXRhPS4lPiVmaWx0ZXIoc3BlY2ltZW49PSJDTU5IIDU3NjgiKSwNCiAgICAgICAgICAgICAgIGFlcyh4ZW5kPXRvdGFsX2xlbmd0aCx5ZW5kPW1vdXRoX3dpZHRoKjAuNCkpKw0KICBnZW9tX3N0YXIoZGF0YT0uJT4lZmlsdGVyKGNsYWRlPT0iUGxhY29kZXJtaSIpLA0KICAgICAgICAgICAgYWVzKHN0YXJzaGFwZT1jbGFkZTIsZmlsbD1jbGFkZTIpLHNpemU9My41LHNob3cubGVnZW5kPUYpKw0KICBnZW9tX2xpbmUoYWVzKHg9LmZpdHRlZCksIGNvbG9yID0gIiMzMzY2RkYiLHNpemU9MS4yNSxzaG93LmxlZ2VuZD1GKSsNCiAgZ2VvbV9saW5lKGFlcyh4PS5sb3dlciksIGNvbG9yID0gIiMzMzY2RkYiLCBsaW5ldHlwZSA9ICJkYXNoZWQiLHNob3cubGVnZW5kPUYpKw0KICBnZW9tX2xpbmUoYWVzKHg9LnVwcGVyKSwgY29sb3IgPSAiIzMzNjZGRiIsIGxpbmV0eXBlID0gImRhc2hlZCIsc2hvdy5sZWdlbmQ9RikrDQogIGdlb21fc3RhcihkYXRhPS4lPiVmaWx0ZXIoY2xhZGU9PSJQbGFjb2Rlcm1pIixjbGFkZTI9PSJFc3QuIEZyb20gU2t1bGwiKSxzaXplPTMuNSwNCiAgICAgICAgICAgIGNvbG9yPSJ3aGl0ZSIsYWVzKHN0YXJzaGFwZT1jbGFkZTIsZmlsbD1jbGFkZTIpLHN0YXJzdHJva2U9MC41LHNob3cubGVnZW5kPUYpKw0KICBnZW9tX3N0YXIoZGF0YT0uJT4lZmlsdGVyKGNsYWRlPT0iUGxhY29kZXJtaSIsY2xhZGUyPT0iT3RoZXIgUGxhY29kZXJtaSIpLHNpemU9My41LA0KICAgICAgICAgICAgc3RhcnNoYXBlPTEsY29sb3I9IndoaXRlIixmaWxsPSJibGFjayIsc3RhcnN0cm9rZT0wLjUsc2hvdy5sZWdlbmQ9RikrDQogIGdlb21fdGV4dChkYXRhPS4lPiVmaWx0ZXIoZ2VudXMgJWluJSBjKCJDb2Njb3N0ZXVzIiwiUGxvdXJkb3N0ZXVzIiwiSW5jaXNvc2N1dHVtIikpLHNpemU9My41LA0KICAgICAgICAgICAgYWVzKHg9MC45KnRvdGFsX2xlbmd0aCppZmVsc2UoZ2VudXM9PSJDb2Njb3N0ZXVzIiwuODUsMSksDQogICAgICAgICAgICAgICB5PTEuMzY1Km1vdXRoX3dpZHRoKmlmZWxzZShnZW51cz09IkNvY2Nvc3RldXMiLC45NSwxKSwNCiAgICAgICAgICAgICAgIGxhYmVsPWdlbnVzKSxoanVzdD0xLHZqdXN0PTAsZm9udGZhY2U9MykrDQogIGdlb21fdGV4dChkYXRhPS4lPiVmaWx0ZXIoc3BlY2ltZW49PSJDTU5IIDU3NjgiKSxzaXplPTMuNSwNCiAgICAgICAgICAgIGFlcyh5PTAuMzUqbW91dGhfd2lkdGgsbGFiZWw9Z2VudXMpLGhqdXN0PTAuNSx2anVzdD0wLGZvbnRmYWNlPTMpKw0KICBsYWJzKHg9IlRvdGFsIExlbmd0aCAoY20pIix5PSJNb3V0aCBXaWR0aCAoY20pIixzdGFyc2hhcGU9Ikdyb3VwIixmaWxsPSJHcm91cCIpKw0KICB0aGVtZV9jbGFzc2ljKCkrDQogIHRoZW1lKGxlZ2VuZC5wb3NpdGlvbj1jKDAuOCwwLjI1KSwNCiAgICAgICAgbGVnZW5kLnRleHQ9ZWxlbWVudF9tYXJrZG93bigpKQ0KYGBgDQoNCklubmVyIG1vdXRoIHdpZHRoIGNvdWxkIG9ubHkgYmUgbWVhc3VyZWQgZm9yICpDb2Njb3N0ZXVzKiBhbW9uZyBhcnRocm9kaXJlcyBrbm93biBmcm9tIGNvbXBsZXRlIHJlbWFpbnMuIE5ldmVydGhlbGVzcywgdGhlIHBhdHRlcm4gaXMgc2ltaWxhciBhcyBmb3Igd2hlbiBtYXhpbXVtIG1vdXRoIHdpZHRoIGlzIGNvbnNpZGVyZWQ6ICpDb2Njb3N0ZXVzIGN1c3BpZGF0dXMqIGlzIGZvdW5kIHRvIGhhdmUgYSBtdWNoIHdpZGVyIG1vdXRoIHRoYW4gc2hhcmtzIG9mIHNpbWlsYXIgc2l6ZSwgd2hlcmVhcyAqRHVua2xlb3N0ZXVzIHRlcnJlbGxpKiBpcyBmb3VuZCB0byBoYXZlIGEgbXVjaCBuYXJyb3dlciAoPXNtYWxsZXIpIG1vdXRoIHRoYW4gZXhwZWN0ZWQgZm9yIGFuIGFuaW1hbCBvZiBpdHMgZXN0aW1hdGVkIGxlbmd0aCB1bmRlciB0aGUgbGVuZ3RoIGVzdGltYXRlcyBvZiBGZXJyw7NuIGV0IGFsLiAoMjAxNykuDQoNCiMjIE1vdXRoIHdpZHRoIHZlcnN1cyB0b3RhbCBsZW5ndGggaW4gb3N0ZWljaHRoeWFucyAoYW5kIGFydGhyb2RpcmVzKQ0KDQoocmVmOm1vdXRod2lkdGhBY3Rpbm9wdGVyeWdpaSkgUGxvdCBvZiBsb2cxMCBtb3V0aCB3aWR0aCB2ZXJzdXMgdG90YWwgbGVuZ3RoIGZvY3VzaW5nIG9uIEFjdGlub3B0ZXJ5Z2lpLCBzaG93aW5nIGhvdyBTaWx1cmlmb3JtZXMgaGF2ZSByZWxhdGl2ZWx5IHdpZGVyIG1vdXRocyB0byBib2R5IHNpemUgc2ltaWxhciB0byAqQ29jY29zdGV1cyoNCg0KYGBge3IsZmlnLmNhcD0iKHJlZjptb3V0aHdpZHRoQWN0aW5vcHRlcnlnaWkpIn0NCmRhdGFfbW91dGhzJT4lDQogIGRyb3BfbmEobW91dGhfd2lkdGgpJT4lDQogIGF1Z21lbnQoZml0Lm1vdXRod2lkdGgsbmV3ZGF0YT0uLGludGVydmFsPSJwcmVkaWN0aW9uIiklPiUNCiAgbXV0YXRlKGFjcm9zcyguZml0dGVkOi51cHBlcix+ZXhwKC4pKnJlZ3Jlc3Npb24uc3RhdHMoZml0Lm1vdXRod2lkdGgpJENGKSwNCiAgICAgICAgIHRvdGFsX2xlbmd0aD1pZmVsc2UoZ2VudXM9PSJEdW5rbGVvc3RldXMiLA0KICAgICAgICAgICAgICAgICAgICAgICAgICAgICBwcmVkaWN0KGZpdC5VSlAsLiksdG90YWxfbGVuZ3RoKSwNCiAgICAgICAgIGNsYWRlMj1jYXNlX3doZW4ob3JkZXI9PSJTaWx1cmlmb3JtZXMifiJTaWx1cmlmb3JtZXMiLA0KICAgICAgICAgICAgICAgICAgICAgICAgICBmYW1pbHk9PSJTZXJyYW5pZGFlIn4iU2VycmFuaWRhZSIsDQogICAgICAgICAgICAgICAgICAgICAgICAgIGNsYWRlPT0iUGxhY29kZXJtaSJ+IlBsYWNvZGVybWkiLA0KICAgICAgICAgICAgICAgICAgICAgICAgICBjbGFkZSAlaW4lIGMoIkFjdGlub3B0ZXJ5Z2lpIiwiU2FyY29wdGVyeWdpaSIpfiJPdGhlciBPc3RlaWNodGh5ZXMiKSklPiUNCiAgZmlsdGVyKCFpcy5uYSh0b3RhbF9sZW5ndGgpfGdlbnVzPT0iRHVua2xlb3N0ZXVzIiklPiUNCiAgYXJyYW5nZShjbGFkZTIpJT4lDQogIGdncGxvdCguLGFlcyh0b3RhbF9sZW5ndGgsbW91dGhfd2lkdGgpKSsNCiAgc2NhbGVfeF9jb250aW51b3VzKHRyYW5zPSJsb2cxMCIpICsgc2NhbGVfeV9jb250aW51b3VzKHRyYW5zPSJsb2cxMCIpKw0KICBnZW9tX3N0YXIoZGF0YT0uJT4lZmlsdGVyKGNsYWRlPT0iQ2hvbmRyaWNodGh5ZXMiKSxzdGFyc2hhcGU9MTMsDQogICAgICAgICAgICBmaWxsPSJsaWdodCBncmF5IixndWlkZT1GLHNob3cubGVnZW5kPUYsY29sb3I9ImRhcmsgZ3JheSIsc2l6ZT0yKSsNCiAgZ2VvbV9zdGFyKGRhdGE9LiU+JWZpbHRlcihnZW51cz09IkR1bmtsZW9zdGV1cyIpLHN0YXJzaGFwZT0xLA0KICAgICAgICAgICAgZmlsbD0ibGlnaHQgZ3JheSIsZ3VpZGU9RixzaG93LmxlZ2VuZD1GLGNvbG9yPSJkYXJrIGdyYXkiLHNpemU9MikrDQogIHNjYWxlX3N0YXJzaGFwZV9tYW51YWwodmFsdWVzPWMoMTUsMTUsMTUsMSksDQogICAgICAgICAgICAgICAgICAgICAgICAgYnJlYWtzPWMoIlNpbHVyaWZvcm1lcyIsIlNlcnJhbmlkYWUiLCJPdGhlciBPc3RlaWNodGh5ZXMiLCJQbGFjb2Rlcm1pIiwiQ2hvbmRyaWNodGh5ZXMiKSwNCiAgICAgICAgICAgICAgICAgICAgICAgICBuYS52YWx1ZT1OQSkrDQogIGdlb21fbGluZShhZXMoeD0uZml0dGVkKSwgY29sb3IgPSAiZ3JheSIsc2l6ZT0xLjI1LHNob3cubGVnZW5kPUYpKw0KICBnZW9tX2xpbmUoYWVzKHg9Lmxvd2VyKSwgY29sb3IgPSAiZ3JheSIsIGxpbmV0eXBlID0gImRhc2hlZCIsc2hvdy5sZWdlbmQ9RikrDQogIGdlb21fbGluZShhZXMoeD0udXBwZXIpLCBjb2xvciA9ICJncmF5IiwgbGluZXR5cGUgPSAiZGFzaGVkIixzaG93LmxlZ2VuZD1GKSsNCiAgZ2VvbV9zdGFyKGRhdGE9LiU+JWZpbHRlcihjbGFkZSE9IkNob25kcmljaHRoeWVzIixnZW51cyE9IkR1bmtsZW9zdGV1cyIpLA0KICAgICAgICAgICAgYWVzKHN0YXJzaGFwZT1jbGFkZTIsZmlsbD1jbGFkZTIpLHNpemU9MikrDQogIGdlb21fc21vb3RoKGRhdGE9LiU+JWZpbHRlcihnZW51cyE9IkR1bmtsZW9zdGV1cyIsY2xhZGUhPSJDaG9uZHJpY2h0aHllcyIpLA0KICAgICAgICAgICAgICBhZXMoY29sb3I9Y2xhZGUyKSxmb3JtdWxhPXl+eCxtZXRob2Q9ImxtIixzZT1GKSsNCiAgc2NhbGVfY29sb3JfbWFudWFsKHZhbHVlcz1jKGh1ZV9wYWwoKSgzKSxOQSksDQogICAgICAgICAgICAgICAgICAgIGJyZWFrcz1jKCJTaWx1cmlmb3JtZXMiLCJTZXJyYW5pZGFlIiwiT3RoZXIgT3N0ZWljaHRoeWVzIiwiUGxhY29kZXJtaSIpLA0KICAgICAgICAgICAgICAgICAgICBuYS52YWx1ZT1OQSkrDQogIHNjYWxlX2ZpbGxfbWFudWFsKHZhbHVlcz1jKGh1ZV9wYWwoKSgzKSwiYmxhY2siKSwNCiAgICAgICAgICAgICAgICAgICAgYnJlYWtzPWMoIlNpbHVyaWZvcm1lcyIsIlNlcnJhbmlkYWUiLCJPdGhlciBPc3RlaWNodGh5ZXMiLCJQbGFjb2Rlcm1pIiksDQogICAgICAgICAgICAgICAgICAgIG5hLnZhbHVlPU5BKSsNCiAgZ2VvbV9zZWdtZW50KGRhdGE9LiU+JWZpbHRlcihnZW51cyAlaW4lIGMoIkNvY2Nvc3RldXMiLCJQbG91cmRvc3RldXMiLCJJbmNpc29zY3V0dW0iKSksDQogICAgICAgICAgICAgICBhZXMoeGVuZD10b3RhbF9sZW5ndGgqMC44LHllbmQ9bW91dGhfd2lkdGgqMS4zKSkrDQogIGdlb21fc3RhcihkYXRhPS4lPiVmaWx0ZXIoY2xhZGU9PSJQbGFjb2Rlcm1pIixnZW51cyE9IkR1bmtsZW9zdGV1cyIpLHNpemU9My41LA0KICAgICAgICAgICAgc3RhcnNoYXBlPTEsY29sb3I9IndoaXRlIixmaWxsPSJibGFjayIsc3RhcnN0cm9rZT0wLjUpKw0KICBnZW9tX3RleHQoZGF0YT0uJT4lZmlsdGVyKGdlbnVzICVpbiUgYygiQ29jY29zdGV1cyIsIlBsb3VyZG9zdGV1cyIsIkluY2lzb3NjdXR1bSIpKSwNCiAgICAgICAgICAgIGFlcyh4PTAuOSp0b3RhbF9sZW5ndGgqaWZlbHNlKGdlbnVzPT0iQ29jY29zdGV1cyIsLjg1LDEpLA0KICAgICAgICAgICAgICAgIHk9MS4zNjUqbW91dGhfd2lkdGgqaWZlbHNlKGdlbnVzPT0iQ29jY29zdGV1cyIsLjk1LDEpLA0KICAgICAgICAgICAgICAgIGxhYmVsPWdlbnVzKSxoanVzdD0xLHZqdXN0PTAsZm9udGZhY2U9MykrDQogIGxhYnMoeD0iVG90YWwgTGVuZ3RoIChjbSkiLHk9Ik1vdXRoIFdpZHRoIChjbSkiLHN0YXJzaGFwZT0iR3JvdXAiLGZpbGw9Ikdyb3VwIixjb2xvcj0iR3JvdXAiKSsNCiAgdGhlbWVfY2xhc3NpYygpKw0KICB0aGVtZShsZWdlbmQucG9zaXRpb249YygwLjgsMC4yKSkNCmBgYA0KDQpCeSBmb2N1c2luZyBvbiB0aGUgcmVsYXRpb25zaGlwIGJldHdlZW4gbW91dGggd2lkdGggYW5kIHRvdGFsIGxlbmd0aCBpbiBhbGwgZmlzaGVzIChpLmUuLCBhZGRpbmcgb3N0ZWljaHRoeWFucyBpbnRvIGNvbnNpZGVyYXRpb24pLCBpdCBpcyBjbGVhciB0aGF0IHRoZSByZWxhdGlvbnNoaXAgYmV0d2VlbiBtb3V0aCB3aWR0aCBhbmQgdG90YWwgbGVuZ3RoIGlzIG5vdCBhcyBjb25zaXN0ZW50IGFzIGlzIHNlZW4gaW4gc2hhcmtzLiBJbiBmYWN0LCBhcnRocm9kaXJlcyBzaG93IG1vdXRoLWJvZHkgcHJvcG9ydGlvbnMgdGhhdCBhcmUgdmVyeSBzaW1pbGFyIHRvIGV4dGFudCBwcmVkYXRvcnkgY2F0ZmlzaGVzIChTaWx1cmlmb3JtZXMpLg0KDQojIyBFcnJvciBpbiB1c2luZyBtb3V0aCBwcm9wb3J0aW9ucyBpbiBzaGFya3MgdG8gZXN0aW1hdGUgbGVuZ3RoIGluIGNvbXBsZXRlIGFydGhyb2RpcmVzDQoNCmBgYHtyfQ0KZm9zc2lsX3RheGFfbW91dGhzJT4lDQogIGZpbHRlcihjbGFkZT09IlBsYWNvZGVybWkiKSU+JQ0KICBkcm9wX25hKHRvdGFsX2xlbmd0aCxtb3V0aF93aWR0aCklPiUNCiAgYXVnbWVudChmaXQubW91dGh3aWR0aCxuZXdkYXRhPS4pJT4lDQogIG11dGF0ZSguZml0dGVkPWV4cCguZml0dGVkKSpyZWdyZXNzaW9uLnN0YXRzKGZpdC5tb3V0aHdpZHRoKSRDRiklPiUNCiAgbXV0YXRlKGVycm9yPTEwMCoodG90YWxfbGVuZ3RoLS5maXR0ZWQpLy5maXR0ZWQpJT4lDQogIG11dGF0ZSh0YXhvbj1pZmVsc2UoZ2VudXM9PSJOZXdzcGVjaWVzIiwiQ01OSCBhc3Bpbm90aG9yYWNpZCIsdGF4b24pKSU+JQ0KICBhZGRfcm93KHRheG9uPSJBbGwgU3BlY2llcyIsZXJyb3I9bWVhbiguJGVycm9yKSklPiUNCiAgc2VsZWN0KHRheG9uLHNwZWNpbWVuLHRvdGFsX2xlbmd0aCwuZml0dGVkLGVycm9yKSU+JQ0KICBrYWJsZShkaWdpdHM9YygxLDEsMSwxLDEpLGNvbC5uYW1lcz1jKCJUYXhvbiIsIlNwZWNpbWVuIiwiVG90YWwgTGVuZ3RoIiwiRXN0LiBMZW5ndGgiLCJQRSIpLA0KICAgICAgICBjYXB0aW9uPSJQZXJjZW50IGVycm9yIHdoZW4gZXN0aW1hdGluZyB0b3RhbCBsZW5ndGggaW4gY29tcGxldGUgc3BlY2ltZW5zIG9mIGFydGhyb2RpcmVzIHVzaW5nIG1vdXRoIHdpZHRoLiIsDQogICAgICAgIGFsaWduPWMoImwiLCJjIiwiYyIsImMiLCJjIikpJT4lDQogIHJvd19zcGVjKDksIGJvbGQgPSBUKSU+JQ0KICBrYWJsZV9zdHlsaW5nKCkNCmBgYA0KDQojIyBFc3RpbWF0aW5nIG1vdXRoIHNpemUgaW4gYXJ0aHJvZGlyZXMgbGlrZSAqQ29jY29zdGV1cyogYXNzdW1pbmcgc2ltaWxhciBwcm9wb3J0aW9ucyB0byBzaGFya3MNCg0KYGBge3J9DQpmaXQudG90YWxfbGVuZ3RoX3RvX21vdXRoX3dpZHRoPC0NCiAgbG0obG9nKG1vdXRoX3dpZHRoKX5sb2codG90YWxfbGVuZ3RoKSxkYXRhX21vdXRocyU+JQ0KICAgICAgIGZpbHRlcihjbGFkZT09IkNob25kcmljaHRoeWVzIiwNCiAgICAgICAgICAgICAgIW9yZGVyICVpbiUgYygiQ2hpbWFlcmlmb3JtZXMiLCJSYWppZm9ybWVzIiksDQogICAgICAgICAgICAgIGdlbnVzIT0iQWxvcGlhcyIpKQ0KZGF0YV9tb3V0aHMlPiUNCiAgZHJvcF9uYShtb3V0aF93aWR0aCx0b3RhbF9sZW5ndGgpJT4lDQogIGZpbHRlcihjbGFkZT09IlBsYWNvZGVybWkiLA0KICAgICAgICAgIWdlbnVzICVpbiUgYygiQW1hemljaHRoeXMiLCJOZXdzcGVjaWVzIikpJT4lDQogIGF1Z21lbnQobmV3ZGF0YT0uLGZpdC50b3RhbF9sZW5ndGhfdG9fbW91dGhfd2lkdGgpJT4lDQogIG11dGF0ZSguZml0dGVkPWV4cCguZml0dGVkKSpyZWdyZXNzaW9uLnN0YXRzKGZpdC50b3RhbF9sZW5ndGhfdG9fbW91dGhfd2lkdGgpJENGLA0KICAgICAgICAgZGlmZj0xMDAqKC5maXR0ZWQtbW91dGhfd2lkdGgpLy5maXR0ZWQpJT4lDQogIHNlbGVjdCh0YXhvbixzcGVjaW1lbixtb3V0aF93aWR0aCwuZml0dGVkLGRpZmYpJT4lDQogIGFycmFuZ2UobW91dGhfd2lkdGgpJT4lDQogIGthYmxlKGRpZ2l0cz0yLGFsaWduPWMoImwiLCJjIiwiYyIsImMiLCJjIiksDQogICAgICAgIGNvbC5uYW1lcyA9IGMoIlRheG9uIiwiU3BlY2ltZW4iLCJBY3R1YWwiLCJFc3RpbWF0ZWQiLCIlIERpZmYuIiksDQogICAgICAgIGNhcHRpb249IkVzdGltYXRlZCBtb3V0aCB3aWR0aCBpbiBhcnRocm9kaXJlIHRheGEgb2Yga25vd24gdG90YWwgbGVuZ3RoIHVzaW5nIGEgbW9kZWwgYmFzZWQgb24gZXh0YW50IHNoYXJrcyAoZXhjbHVkaW5nIDxpPkFsb3BpYXM8L2k+KS4gTm90ZSB0aGF0IDxpPkhvbG9uZW1hPC9pPiBhbmQgPGk+V2F0c29ub3N0ZXVzPC9pPiBhcmUgYmFzZWQgb2ZmIG9mIGRvcnNvdmVudHJhbGx5IGNydXNoZWQgc3BlY2ltZW5zIGJ1dCA8aT5JbmNpc29zY3V0dW08L2k+LCA8aT5Db2Njb3N0ZXVzPC9pPiwgYW5kIDxpPlBsb3VyZG9zdGV1czwvaT4gYXJlIGJhc2VkIG9mZiBvZiB0aHJlZS1kaW1lbnNpb25hbCBzcGVjaW1lbnMgb3IgcmVjb25zdHJ1Y3Rpb25zLiBBbGwgbWVhc3VyZW1lbnRzIGluIGNtLiIpJT4lDQogIGFkZF9oZWFkZXJfYWJvdmUoYygiICI9MiwiTW91dGggV2lkdGgiPTMpKSU+JQ0KICBjb2x1bW5fc3BlYygxLCBpdGFsaWMgPSBUKSU+JQ0KICBrYWJsZV9zdHlsaW5nKCkNCmBgYA0KDQpJZiByZXZlcnNpbmcgdGhlIGNvbnNpZGVyYXRpb24gYW5kIHRyeWluZyB0byBlc3RpbWF0ZSBtb3V0aCB3aWR0aCBpbiBhcnRocm9kaXJlcyBiYXNlZCBvbiBrbm93biB0b3RhbCBsZW5ndGgsIGFydGhyb2RpcmVzIGFyZSBwcmVkaWN0ZWQgdG8gaGF2ZSBtb3V0aHMgbGVzcyB0aGFuIGhhbGYgdGhlaXIgYWN0dWFsIHdpZHRoLiBUaGlzIGlzIHBhcnRpY3VsYXJseSBwcm9ub3VuY2VkIGluICpDb2Njb3N0ZXVzKiwgKkluY2lzb3NjdXR1bSosIGFuZCAqUGxvdXJkb3N0ZXVzKiwgd2hlcmUgdGhlIG1vdXRocyBjYW4gYmUgbWVhc3VyZWQgZnJvbSAzRCBzcGVjaW1lbnMgb3IgcmVjb25zdHJ1Y3Rpb25zIGFuZCBhcmUgbm90IHN1YmplY3QgdG8gZG9yc292ZW50cmFsIGNydXNoaW5nLg0KDQpgYGB7cn0NCmRhdGFfbW91dGhzJT4lDQogIHNlbGVjdCgtbW91dGhfd2lkdGgpJT4lDQogIHJlbmFtZShtb3V0aF93aWR0aD1pbm5lcl9tb3V0aF93aWR0aCklPiUNCiAgZHJvcF9uYShtb3V0aF93aWR0aCx0b3RhbF9sZW5ndGgpJT4lDQogIGF1Z21lbnQobmV3ZGF0YT0uLGZpdC50b3RhbF9sZW5ndGhfdG9fbW91dGhfd2lkdGgpJT4lDQogIG11dGF0ZSguZml0dGVkPWV4cCguZml0dGVkKSpyZWdyZXNzaW9uLnN0YXRzKGZpdC50b3RhbF9sZW5ndGhfdG9fbW91dGhfd2lkdGgpJENGLA0KICAgICAgICAgZGlmZj0xMDAqKC5maXR0ZWQtbW91dGhfd2lkdGgpLy5maXR0ZWQpJT4lDQogIHNlbGVjdCh0YXhvbixzcGVjaW1lbixtb3V0aF93aWR0aCwuZml0dGVkLGRpZmYpJT4lDQogIGFycmFuZ2UobW91dGhfd2lkdGgpJT4lDQogIGthYmxlKGRpZ2l0cz0yLGFsaWduPWMoImwiLCJjIiwiYyIsImMiLCJjIiksDQogICAgICAgIGNvbC5uYW1lcyA9IGMoIlRheG9uIiwiU3BlY2ltZW4iLCJBY3R1YWwiLCJFc3RpbWF0ZWQiLCIlIERpZmYuIiksDQogICAgICAgIGNhcHRpb249IkVzdGltYXRlZCBtb3V0aCB3aWR0aCBpbiBhcnRocm9kaXJlIHRheGEgb2Yga25vd24gdG90YWwgbGVuZ3RoIHVzaW5nIGEgbW9kZWwgYmFzZWQgb24gZXh0YW50IHNoYXJrcyBhbmQgaW5uZXIgbW91dGggd2lkdGguIEFsbCBtZWFzdXJlbWVudHMgaW4gY20uIiklPiUNCiAgYWRkX2hlYWRlcl9hYm92ZShjKCIgIj0yLCJNb3V0aCBXaWR0aCI9MykpJT4lDQogIGNvbHVtbl9zcGVjKDEsIGl0YWxpYyA9IFQpJT4lDQogIGthYmxlX3N0eWxpbmcoKQ0KYGBgDQoNCiMgRXN0aW1hdGluZyB0aGUgbGVuZ3RoIG9mIGFydGhyb2RpcmVzIHVzaW5nIGFwcHJveGltYXRlZCBVSlANCg0KYGBge3J9DQpmaXQuYXBwcm94X1VKUF9vbGQ8LWxtKGxvZyh0b3RhbF9sZW5ndGgpfmxvZyhVSlApLA0KICAgICAgICAgICAgICAgICAgIGRhdGFfbW91dGhzICU+JSBkcm9wX25hKG1vdXRoX2xlbmd0aCkgJT4lIGZpbHRlcihjbGFkZT09IkNob25kcmljaHRoeWVzIikgJT4lDQogICAgICAgICAgICAgICAgICAgICAgICAgICAgICAgICAgICAgICAgICBtdXRhdGUoVUpQPXJhbWFudWphbi5hcHByb3gobW91dGhfbGVuZ3RoKjIsbW91dGhfd2lkdGgpLzIpKQ0KZml0LmFwcHJveF9VSlA8LWxtKGxvZyh0b3RhbF9sZW5ndGgpfmxvZyhVSlApLA0KICAgICAgICAgICAgICAgICAgIGRhdGFfbW91dGhzICU+JSBkcm9wX25hKG1vdXRoX2xlbmd0aCkgJT4lIGZpbHRlcihjbGFkZT09IkNob25kcmljaHRoeWVzIikgJT4lDQogICAgICAgICAgICAgICAgICAgICBmaWx0ZXIoIWdlbnVzICVpbiUgYygiQ2V0b3JoaW51cyIsIlJoaW5jb2RvbiIsIk1lZ2FjaGFzbWEiLCJBbG9waWFzIiksDQogICAgICAgICAgICAgICAgICAgICAgICAgICAgb3JkZXIgJWluJSBjKCJMYW1uaWZvcm1lcyIsIkNhcmNoYXJoaW5pZm9ybWVzIiwiSGV4YW5jaGlpZm9ybWVzIikpICU+JQ0KICAgICAgICAgICAgICAgICAgICAgbXV0YXRlKFVKUD1yYW1hbnVqYW4uYXBwcm94KG1vdXRoX2xlbmd0aCoyLG1vdXRoX3dpZHRoKS8yKSkNCmBgYA0KDQoqKlN1bW1hcnkgb2YgbW9kZWwgaW5jbHVkaW5nIGFsbCB0YXhhKioNCg0KYGBge3J9DQpzdW1tYXJ5KGZpdC5hcHByb3hfVUpQX29sZCkNCmBgYA0KDQoqKlN1bW1hcnkgb2YgbW9kZWwgaW5jbHVkaW5nIG9ubHkgQ2FyY2hhcmhpbmlmb3JtZXMsIExhbW5pZm9ybWVzLCBhbmQgSGV4YW5jaGlpZm9ybWVzKioNCg0KYGBge3J9DQpzdW1tYXJ5KGZpdC5hcHByb3hfVUpQKQ0KYGBgDQoNCioqUmVncmVzc2lvbiBzdGF0aXN0aWNzKioNCg0KYGBge3J9DQpyYmluZCgiQWxsIFRheGEiPXJlZ3Jlc3Npb24uc3RhdHMoZml0LmFwcHJveF9VSlApLA0KICAgICAgIkNhcmNoYXJoaW5pZm9ybWVzLCBMYW1uaWZvcm1lcywgSGV4YW5jaGlpZm9ybW5lcyI9cmVncmVzc2lvbi5zdGF0cyhmaXQuYXBwcm94X1VKUCkpDQpgYGANCg0KKHJlZjpzY2FsZWxvY2F0aW9uZXN0aW1hdGVkdWpwKSBTY2FsZSB2ZXJzdXMgbG9jYXRpb24gcGxvdCB1c2luZyBlc3RpbWF0ZWQgVUpQIGFuZCBhbiB1bnRyYW5zZm9ybWVkIG1vZGVsICgqKkEqKikgYW5kIGEgbG9nLXRyYW5zZm9ybWVkIG1vZGVsICgqKkIqKikuDQoNCmBgYHtyLGZpZy53aWR0aD0xMCxmaWcuYXNwPTAuMzc1LGZpZy5jYXA9IihyZWY6c2NhbGVsb2NhdGlvbmVzdGltYXRlZHVqcCkifQ0KcGFyKG1mcm93PWMoMSwyKSkNCnBsb3QobG0odG90YWxfbGVuZ3RoflVKUCwNCiAgICAgICAgICAgICAgICAgICBkYXRhX21vdXRocyAlPiUgDQogICAgICAgICAgICAgICAgICAgICBkcm9wX25hKG1vdXRoX2xlbmd0aCkgJT4lIGZpbHRlcihjbGFkZT09IkNob25kcmljaHRoeWVzIikgJT4lDQogICAgICAgICAgICAgICAgICAgICBtdXRhdGUoVUpQPXJhbWFudWphbi5hcHByb3gobW91dGhfbGVuZ3RoKjIsbW91dGhfd2lkdGgpLzIpKSwNCiAgICAgMykNCnBsb3QoZml0LmFwcHJveF9VSlBfb2xkLDMpDQpgYGANCg0KYGBge3IsZmlnLmNhcD0iUmVzaWR1YWxzIHZlcnN1cyBmaXR0ZWQgcGxvdCB1c2luZyBlc3RpbWF0ZWQgVUpQIGFuZCBhIGRhdGFzZXQgb2YgYWxsIHNoYXJrcyJ9DQpkYXRhX21vdXRocyU+JQ0KICBkcm9wX25hKG1vdXRoX2xlbmd0aCxtb3V0aF93aWR0aCkgJT4lDQogIGZpbHRlcihjbGFkZT09IkNob25kcmljaHRoeWVzIikgJT4lDQogIG11dGF0ZShVSlA9cmFtYW51amFuLmFwcHJveChtb3V0aF9sZW5ndGgqMixtb3V0aF93aWR0aCkvMiwNCiAgICAgICAgIGNsYWRlMj1jYXNlX3doZW4oZ2VudXMgPT0gIkFsb3BpYXMiIH4gIkFsb3BpYXMiLA0KICAgICAgICAgICBvcmRlciA9PSAiU3F1YWxpZm9ybWVzIiB+ICJTcXVhbGlmb3JtZXMiLA0KICAgICAgICAgICBvcmRlciA9PSAiT3JlY3RvbG9iaWZvcm1lcyIgfiAiT3JlY3RvbG9iaWZvcm1lcyIsDQogICAgICAgICAgIG9yZGVyID09ICJDaGltYWVyaWZvcm1lcyIgfiAiQ2hpbWFlcmlmb3JtZXMiLA0KICAgICAgICAgICBvcmRlciA9PSAiUmhpbm9wcmlzdGlmb3JtZXMiIH4gIlJoaW5vcHJpc3RpZm9ybWVzIiwNCiAgICAgICAgICAgZ2VudXMgJWluJSBjKCJNZWdhY2hhc21hIiwiQ2V0b3JoaW51cyIsIlJoaW5jb2RvbiIpIH4gIkZpbHRlci1GZWVkaW5nIFNoYXJrcyIsDQogICAgICAgICAgIFRSVUUgfiAiT3RoZXIgVGF4YSIpKSU+JQ0KICBtdXRhdGUoY2xhZGUyPWZhY3RvcihjbGFkZTIsb3JkZXJlZD1ULGxldmVscz1jKCJBbG9waWFzIiwNCiAgICAgICAgICAgICAgICAgICAgICAgICAgICAgICAgICAgICAgICAgICAgICAgICAiRmlsdGVyLUZlZWRpbmcgU2hhcmtzIiwNCiAgICAgICAgICAgICAgICAgICAgICAgICAgICAgICAgICAgICAgICAgICAgICAgICAiQ2hpbWFlcmlmb3JtZXMiLA0KICAgICAgICAgICAgICAgICAgICAgICAgICAgICAgICAgICAgICAgICAgICAgICAgICJPcmVjdG9sb2JpZm9ybWVzIiwNCiAgICAgICAgICAgICAgICAgICAgICAgICAgICAgICAgICAgICAgICAgICAgICAgICAiUmhpbm9wcmlzdGlmb3JtZXMiLA0KICAgICAgICAgICAgICAgICAgICAgICAgICAgICAgICAgICAgICAgICAgICAgICAgICJTcXVhbGlmb3JtZXMiLA0KICAgICAgICAgICAgICAgICAgICAgICAgICAgICAgICAgICAgICAgICAgICAgICAgICJPdGhlciBUYXhhIikpKSU+JQ0KICBhdWdtZW50KGZpdC5hcHByb3hfVUpQX29sZCxuZXdkYXRhPS4pJT4lDQogIGdncGxvdChhZXMoLmZpdHRlZCwucmVzaWQpKSArDQogIGdlb21faGxpbmUoeWludGVyY2VwdD0wLGxpbmV0eXBlPSJkb3R0ZWQiLGNvbG9yPSJsaWdodCBncmV5IikgKw0KICBnZW9tX3BvaW50KGFlcyhmaWxsPWNsYWRlMixzaGFwZT1jbGFkZTIpKSArDQogIGdlb21fc21vb3RoKG1ldGhvZD0ibG9lc3MiLGZvcm11bGE9eX54LGNvbG9yPSJyZWQiLHNlPUYsc2l6ZT0wLjUpICsNCiAgc2NhbGVfc2hhcGVfbWFudWFsKHZhbHVlcz1jKDIyLDIyLDI0LDI0LDI0LDI0LDIxKSkrDQogIHNjYWxlX2ZpbGxfbWFudWFsKHZhbHVlcz1jKGh1ZV9wYWwoKSg2KSwiZ3JleSIpKSsNCiAgdGhlbWVfY2xhc3NpYygpKw0KICBsYWJzKHg9IkZpdHRlZCBWYWx1ZXMiLGZpbGw9IkNsYWRlIixzaGFwZT0iQ2xhZGUiLHk9IlJlc2lkdWFscyIpKw0KICB0aGVtZShsZWdlbmQucG9zaXRpb249ImJvdHRvbSIpDQpgYGANCg0KYGBge3IsZmlnLmNhcD0iUmVzaWR1YWxzIHZlcnN1cyBmaXR0ZWQgcGxvdCBvZiBtb2RlbCBvbmx5IGNvbnNpZGVyaW5nIENhcmNoYXJoaW5pZm9ybWVzLCBMYW1uaWZvcm1lcywgYW5kIEhleGFuY2hpZm9ybWVzIn0NCnBsb3QoZml0LmFwcHJveF9VSlAsMSkNCmBgYA0KDQojIyBUZXN0aW5nIGhvdyBjbG9zZWx5IGVzdGltYXRlZCBVSlAgbWF0Y2hlcyBhY3R1YWwgVUpQIGluIEZTQkMgU2hhcmtzDQoNCmBgYHtyfQ0KI0Egc2VwYXJhdGUgZXF1YXRpb24gd2FzIGNhbGxlZCBmb3IgdXNpbmcgZXN0aW1hdGVkIGphdyBwZXJpbWV0ZXIgcmF0aGVyIHRoYW4gYWN0dWFsLCBhcyBvdGhlcndpc2UgUiBmcmVha3Mgb3V0IGlmIHlvdSB0cnkgdG8gY2hhbmdlIGFuIGV4aXN0aW5nIGVxdWF0aW9uIHRvIHVzZSBhIG5ldyB2YXJpYWJsZS4NCmxvd3J5X2FwcHJveDwtbG0odG90YWxfbGVuZ3RoIH4gcGVyaW1ldGVyX2VzdCxsb3dyeV9zaGFya3MgJT4lIG11dGF0ZShwZXJpbWV0ZXJfZXN0PVVKUCkpDQoNCmZzYmNfc2hhcmtzJT4lDQogIG11dGF0ZShwZXJpbWV0ZXJfZXN0PXJhbWFudWphbi5hcHByb3gobW91dGhfbGVuZ3RoKjIsbW91dGhfd2lkdGgpLzIpJT4lDQogIG11dGF0ZShmaXRfdWpwPXByZWRpY3QoZml0LlVKUCwuLGludGVydmFsPSJwcmVkaWN0aW9uIilbLDFdLA0KICAgICAgICAgbHdyX3VqcD1wcmVkaWN0KGZpdC5VSlAsLixpbnRlcnZhbD0icHJlZGljdGlvbiIpWywyXSwNCiAgICAgICAgIHVwcl91anA9cHJlZGljdChmaXQuVUpQLC4saW50ZXJ2YWw9InByZWRpY3Rpb24iKVssM10sDQogICAgICAgICBQSV91anA9cGFzdGUwKCIoIixyb3VuZChsd3JfdWpwLDEpLCLigJMiLHJvdW5kKHVwcl91anAsMSksIikiKSwNCiAgICAgICAgIFBFX3VqcD0oKGZpdF91anAtdG90YWxfbGVuZ3RoKS9maXRfdWpwKSoxMDAsDQogICAgICAgICBmaXRfbW91dGg9ZXhwKHByZWRpY3QoZml0Lm1vdXRod2lkdGgsLixpbnRlcnZhbD0icHJlZGljdGlvbiIpWywxXSksDQogICAgICAgICBsd3JfbW91dGg9ZXhwKHByZWRpY3QoZml0Lm1vdXRod2lkdGgsLixpbnRlcnZhbD0icHJlZGljdGlvbiIpWywyXSksDQogICAgICAgICB1cHJfbW91dGg9ZXhwKHByZWRpY3QoZml0Lm1vdXRod2lkdGgsLixpbnRlcnZhbD0icHJlZGljdGlvbiIpWywzXSksDQogICAgICAgICBQSV9tb3V0aD1wYXN0ZTAoIigiLHJvdW5kKGx3cl9tb3V0aCwxKSwi4oCTIixyb3VuZCh1cHJfbW91dGgsMSksIikiKSwNCiAgICAgICAgIFBFX21vdXRoPSgoZml0X21vdXRoLXRvdGFsX2xlbmd0aCkvZml0X21vdXRoKSoxMDAsDQogICAgICAgICBmaXRfYXBwcm94PXByZWRpY3QobG0odG90YWxfbGVuZ3RoIH4gcGVyaW1ldGVyX2VzdCxsb3dyeV9zaGFya3MgJT4lIG11dGF0ZShwZXJpbWV0ZXJfZXN0PVVKUCkpLA0KICAgICAgICAgICAgICAgICAgICAgICAgICAgIC4saW50ZXJ2YWw9InByZWRpY3Rpb24iKVssMV0sDQogICAgICAgICBsd3JfYXBwcm94PXByZWRpY3QobG0odG90YWxfbGVuZ3RoIH4gcGVyaW1ldGVyX2VzdCxsb3dyeV9zaGFya3MgJT4lIG11dGF0ZShwZXJpbWV0ZXJfZXN0PVVKUCkpLA0KICAgICAgICAgICAgICAgICAgICAgICAgICAgIC4saW50ZXJ2YWw9InByZWRpY3Rpb24iKVssMl0sDQogICAgICAgICB1cHJfYXBwcm94PXByZWRpY3QobG0odG90YWxfbGVuZ3RoIH4gcGVyaW1ldGVyX2VzdCxsb3dyeV9zaGFya3MgJT4lIG11dGF0ZShwZXJpbWV0ZXJfZXN0PVVKUCkpLA0KICAgICAgICAgICAgICAgICAgICAgICAgICAgIC4saW50ZXJ2YWw9InByZWRpY3Rpb24iKVssM10sDQogICAgICAgICBQSV9hcHByb3g9cGFzdGUwKCIoIixyb3VuZChsd3JfYXBwcm94LDEpLCLigJMiLHJvdW5kKHVwcl9hcHByb3gsMSksIikiKSwNCiAgICAgICAgIFBFX2FwcHJveD0oKGZpdF9hcHByb3gtdG90YWxfbGVuZ3RoKS9maXRfYXBwcm94KSoxMDApJT4lDQogIGNvbHVtbl90b19yb3duYW1lcygic3BlY2ltZW4iKSU+JQ0KICBtdXRhdGUoZXN0X2RpZmZfbW91dGg9MTAwKmFicyhmaXRfdWpwLWZpdF9tb3V0aCkvZml0X21vdXRoLA0KICAgICAgICAgZXN0X2RpZmZfYXBwcm94PTEwMCphYnMoZml0X3VqcC1maXRfYXBwcm94KS9maXRfYXBwcm94KSU+JQ0KICBzZWxlY3QodGF4b24sdG90YWxfbGVuZ3RoLA0KICAgICAgICAgVUpQLGZpdF91anAsUElfdWpwLFBFX3VqcCwNCiAgICAgICAgIG1vdXRoX3dpZHRoLGZpdF9tb3V0aCxQSV9tb3V0aCxQRV9tb3V0aCxlc3RfZGlmZl9tb3V0aCwNCiAgICAgICAgIHBlcmltZXRlcl9lc3QsZml0X2FwcHJveCxQSV9hcHByb3gsUEVfYXBwcm94LGVzdF9kaWZmX2FwcHJveCklPiUNCiAga2FibGUoZGlnaXRzPTEsDQogICAgICAgIGFsaWduPWMoImwiLCJjIiwiYyIsImMiLCJjIiwiYyIsImMiLCJjIiwiYyIsImMiLCJjIiwiYyIsImMiLCJjIiwiYyIpLA0KICAgICAgICBjYXB0aW9uPSJUZXN0aW5nIGhvdyBjbG9zZWx5IFJhbWFudWphbidzIGFwcHJveGltYXRpb24gb2YgZWxsaXB0aWNhbCBwZXJpbWV0ZXIuIGFwcHJveGltYXRlcyBVSlAgaW4gZHJpZWQgamF3cyBvZiBzaGFya3MuIFBFIGlzIGVycm9yIHJlbGF0aXZlIHRvIG1lYXN1cmVkIHRvdGFsIGxlbmd0aCBhbmQgcGVyY2VudCBkaWZmZXJlbmNlIGlzIGRpZmZlcmVuY2UgcmVsYXRpdmUgdG8gVUpQLWRlcml2ZWQgZXN0aW1hdGUuIEFsbCBsZW5ndGhzIGluIGNtLiIsDQogICAgICAgIGNvbC5uYW1lcyA9IGMoIlRheG9uIiwiVG90YWwgTGVuZ3RoIiwNCiAgICAgICAgICAgICAgICAgICAgICAiVmFsdWUiLCJFc3QuIiwiOTUlIFAuSS4iLCJQRSIsDQogICAgICAgICAgICAgICAgICAgICAgIlZhbHVlIiwiRXN0LiIsIjk1JSBQLkkuIiwiUEUiLCJQZXJjZW50IERpZmZlcmVuY2UiLA0KICAgICAgICAgICAgICAgICAgICAgICJWYWx1ZSIsIkVzdC4iLCI5NSUgUC5JLiIsIlBFIiwiUGVyY2VudCBEaWZmZXJlbmNlIikpJT4lDQogIGNvbHVtbl9zcGVjKDIsIGl0YWxpYyA9IFQpJT4lDQogIGNvbHVtbl9zcGVjKGMoNCwxMyksIGJvbGQgPSBUKSU+JQ0KICBhZGRfaGVhZGVyX2Fib3ZlKGMoIiAiPTMsIlVwcGVyIEphdyBQZXJpbWV0ZXIiPTQsIk1vdXRoIFdpZHRoIj01LCJBcHByb3hpbWF0ZWQgVUpQIj01KSklPiUNCiAga2FibGVfc3R5bGluZygpICU+JSANCiAgc2Nyb2xsX2JveCh3aWR0aCA9ICIxMDAlIikNCmBgYA0KDQojIyBDb21wYXJpbmcgZXN0aW1hdGVkIFVKUCB0byBhY3R1YWwgVUpQIGluIGFydGhyb2RpcmVzDQoNCmBgYHtyfQ0KZGF0YV9tb3V0aHMgJT4lDQogIGZpbHRlcihjbGFkZT09IlBsYWNvZGVybWkiKSAlPiUNCiAgZHJvcF9uYShVSlApICU+JQ0KICBtdXRhdGUoVUpQMj1yYW1hbnVqYW4uYXBwcm94KG1vdXRoX2xlbmd0aCxtb3V0aF93aWR0aCkvMiwNCiAgICAgICAgIFVKUDM9YyhOQSwxMTYsNTUsODQsMTAwKSwNCiAgICAgICAgIHJhdGlvPVVKUDIvVUpQKSAlPiUNCiAgc2VsZWN0KHRheG9uLHNwZWNpbWVuLCBVSlAsIFVKUDMsIFVKUDIsIHJhdGlvKSAlPiUNCiAga2FibGUoZGlnaXRzPTIsYWxpZ249YygibCIsImMiLCJjIiwiYyIsImMiLCJjIiksDQogICAgICAgIGNvbC5uYW1lcz1jKCJUYXhvbiIsIlNwZWNpbWVuIiwiQWN0dWFsIFVKUCAoRmVycsOzbikiLCJBY3R1YWwgVUpQIChNZWFzdXJlZCBieSBBdXRob3IpIiwiRXN0aW1hdGVkIFVKUCAoUmFtYW51amFuIEFwcHJveGltYXRpb24pIiwiUmF0aW8iKSwNCiAgICAgICAgY2FwdGlvbj0iQ29tcGFyaXNvbiBvZiBlc3RpbWF0ZWQgVUpQIHRvIGRpcmVjdGx5IG1lYXN1cmVkIFVKUCBpbiBhcnRocm9kaXJlcy4gUmF0aW8gaXMgdGhlIHJhdGlvIGJldHdlZW4gdGhlIGFwcHJveGltYXRlZCBVSlAgYW5kIHRoZSBtZWFzdXJlbWVudHMgcmVwb3J0ZWQgaW4gRmVycsOzbiBldCBhbC4gKDIwMTcpIikgJT4lDQogIGNvbHVtbl9zcGVjKDEsaXRhbGljID0gVCkgJT4lDQogIGthYmxlX3N0eWxpbmcoKQ0KYGBgDQoNClJhbWFudWphbidzIGFwcHJveGltYXRpb24gZG9lcyBub3QgYWNjdXJhdGVseSBlc3RpbWF0ZSBVSlAgaW4gYXJ0aHJvZGlyZXMsIGluIGNvbnRyYXN0IHRvIHNoYXJrcy4gVGhpcyBpcyBsaWtlbHkgYmVjYXVzZSB0aGUgc2t1bGxzIGFuZCBtb3V0aHMgb2YgYXJ0aHJvZGlyZXMgYXJlIGJsb2NraWVyIGFuZCBtb3JlIHJlY3Rhbmd1bGFyIGNvbXBhcmVkIHRvIHRoZSBqYXcgY2FydGlsYWdlcyBvZiBzaGFya3MsIGFuZCB0aHVzIHRoZWlyIFVKUCBjYW5ub3QgYmUgYXBwcm94aW1hdGVkIGFzIGFuIGlkZWFsaXplZCBlbGxpcHNlLiBUaGUgcmF0aW8gb2YgYWN0dWFsIFVKUCB0byBlc3RpbWF0ZWQgVUpQIGlzIHNpbWlsYXIgaW4gYm90aCAqRHVua2xlb3N0ZXVzKiBhbmQgKkNvY2Nvc3RldXMqLCBzdWdnZXN0aW5nIHRoYXQgVUpQIGhhcyBiZWVuIG1lYXN1cmVkL2VzdGltYXRlZCBjb3JyZWN0bHkgYW5kIHRoZSBkaWZmZXJlbmNlIGlzIGR1ZSB0byBhY3R1YWwgZGlmZmVyZW5jZXMgaW4gdGhlIHNrdWxsIHNoYXBlIG9mIGFydGhyb2RpcmVzLg0KDQpNZWFzdXJlbWVudHMgb2YgVUpQIG9uIHNwZWNpbWVucyB0YWtlbiBieSB0aGUgcHJlc2VudCBhdXRob3Igb24gc3BlY2ltZW5zIG9mICpEdW5rbGVvc3RldXMqIGFyZSBzaW1pbGFyIHRvIHRoZSB2YWx1ZXMgcmVwb3J0ZWQgaW4gRmVycsOzbiBldCBhbC4gKDIwMTcpLCBzdWdnZXN0aW5nIHRoZSBzbWFsbGVyIFVKUHMgcHJlZGljdGVkIGJ5IFJhbWFudWphbidzIGFwcHJveGltYXRpb24gY2Fubm90IGJlIGF0dHJpYnV0ZWQgdG8gbWVhc3VyZW1lbnQgZXJyb3IuIERpZmZlcmVuY2VzIGJldHdlZW4gRmVycsOzbiBldCBhbC4gKDIwMTcpJ3MgbWVhc3VyZW1lbnRzIGFuZCB0aGUgYXV0aG9yJ3MgYXJlIHByb2JhYmx5IGR1ZSB0byBtZWFzdXJlbWVudCBlcnJvciBmcm9tIHRoZSBkaWZmaWN1bHRpZXMgaW4gY29uc2lzdGVudGx5IHBsYWNpbmcgYSB0YXBlIG1lYXN1cmVyIGFsb25nIHRoZSBVSlAgb2YgbW91bnRlZCBzcGVjaW1lbnMsIGVzcGVjaWFsbHkgYXMgVUpQIGlzIG5vdCBhIHBlcmltZXRlciBhbG9uZyBhIHdlbGwtZGVmaW5lZCBlZGdlIGJ1dCBhIGRldG91ciBtdXN0IGJlIG1hZGUgdG8gZW5jb21wYXNzIHRoZSB1cHBlciBzdXByYWduYXRoYWxzLiBPbmUgb2YgdGhlIHN0YWZmIG1lbWJlcnMgYXQgdGhlIENsZXZlbGFuZCBNdXNldW0gb2YgTmF0dXJhbCBIaXN0b3J5IChBLiBNY0dlZSksIHdhcyBpbnZvbHZlZCBpbiB0aGUgY29sbGVjdGlvbiBvZiBVSlAgZm9yIGJvdGggc3R1ZGllcywgYW5kIGRpc2N1c3Npb25zIHdpdGggQS4gTWNHZWUgc3VnZ2VzdCB0aGUgdHdvIG1lYXN1cmVtZW50cyB3ZXJlIGZhaXJseSBjbG9zZS4NCg0KIyMgTGVuZ3RoIGVzdGltYXRlcyBpbiBhcnRocm9kaXJlcyB1c2luZyBlc3RpbWF0ZWQgVUpQDQoNCmBgYHtyfQ0KZGF0YV9tb3V0aHMgJT4lIGZpbHRlcihjbGFkZT09IlBsYWNvZGVybWkiKSAlPiUgZHJvcF9uYShVSlApICU+JQ0KICBhcnJhbmdlKG1vdXRoX3dpZHRoKSAlPiUNCiAgYXVnbWVudChmaXQuYXBwcm94X1VKUF9vbGQsbmV3ZGF0YT0uLGludGVydmFsPSJwcmVkaWN0IikgJT4lDQogIG11dGF0ZShhY3Jvc3MoLmZpdHRlZDoudXBwZXIsfmV4cCguKSpyZWdyZXNzaW9uLnN0YXRzKGZpdC5hcHByb3hfVUpQX29sZCkkQ0YpKSAlPiUNCiAgcmVuYW1lX2F0KHZhcnMoc3RhcnRzX3dpdGgoJy4nKSksIGZ1bnMocGFzdGUwKCJmaXQxIiwuKSkpJT4lDQogIGF1Z21lbnQoZml0LmFwcHJveF9VSlAsbmV3ZGF0YT0uLGludGVydmFsPSJwcmVkaWN0IikgJT4lDQogIHJlbmFtZV9hdCh2YXJzKHN0YXJ0c193aXRoKCcuJykpLCBmdW5zKHBhc3RlMCgiZml0MiIsLikpKSU+JQ0KICBtdXRhdGUoYWNyb3NzKGZpdDIuZml0dGVkOmZpdDIudXBwZXIsfmV4cCguKSpyZWdyZXNzaW9uLnN0YXRzKGZpdC5hcHByb3hfVUpQKSRDRiksDQogICAgICAgICBmaXRfZmVycm9uPXByZWRpY3QoZml0LlVKUCwuKSwNCiAgICAgICAgIGZpdDEucmFuZ2U9cGFzdGUwKCIoIixyb3VuZChmaXQxLmxvd2VyLDEpLCLigJMiLHJvdW5kKGZpdDEudXBwZXIsMSksIikiKSwNCiAgICAgICAgIGZpdDIucmFuZ2U9cGFzdGUwKCIoIixyb3VuZChmaXQyLmxvd2VyLDEpLCLigJMiLHJvdW5kKGZpdDIudXBwZXIsMSksIikiKSklPiUNCiAgbXV0YXRlKFBFMT0xMDAqKGZpdDEuZml0dGVkLWZpdF9mZXJyb24pL2ZpdDEuZml0dGVkLA0KICAgICAgICAgUEUyPTEwMCooZml0Mi5maXR0ZWQtZml0X2ZlcnJvbikvZml0Mi5maXR0ZWQpICU+JQ0KICBzZWxlY3QodGF4b24sc3BlY2ltZW4sDQogICAgICAgICBmaXQxLmZpdHRlZCxmaXQxLnJhbmdlLFBFMSwNCiAgICAgICAgIGZpdDIuZml0dGVkLGZpdDIucmFuZ2UsUEUyKSAlPiUNCiAga2FibGUoY2FwdGlvbj0iRXN0aW1hdGVkIGxlbmd0aCBvZiBhcnRocm9kaXJlcyB1c2luZyBVSlAgYW5kIGVzdGltYXRlZCBVSlAgZnJvbSBhIGJyb2FkZXIgc2FtcGxlIG9mIHNoYXJrcyIsDQogICAgICAgIGRpZ2l0cz0xLA0KICAgICAgICBhbGlnbj1jKCJsIiwiYyIsImMiLCJjIiwiYyIsImMiLCJjIiwiYyIpLA0KICAgICAgICBjb2wubmFtZXMgPSBjKCJUYXhvbiIsIlNwZWNpbWVuIiwNCiAgICAgICAgICAgICAgICAgICAgICAiRXN0LiIsIjk1JSBQLkkuIiwiUEUiLA0KICAgICAgICAgICAgICAgICAgICAgICJFc3QuIiwiOTUlIFAuSS4iLCJQRSIpKSU+JQ0KICBhZGRfaGVhZGVyX2Fib3ZlKGMoIiAiPTIsIlVzaW5nIEFsbCBTaGFya3MiPTMsIlByZWRhdG9yeSBDYXJjaGFyaGluaWZvcm1lcywgTGFtbmlmb3JtZXMsIGFuZCBIZXhhbmNoaWlmb3JtZXMgT25seSI9MykpJT4lDQogIGNvbHVtbl9zcGVjKDEsIGl0YWxpYyA9IFQpJT4lDQogIGthYmxlX3N0eWxpbmcoKQ0KYGBgDQoNCmBgYHtyfQ0KZGF0YV9tb3V0aHMlPiUNCiAgZHJvcF9uYShtb3V0aF9sZW5ndGgsbW91dGhfd2lkdGgpICU+JQ0KICBmaWx0ZXIoZ2VudXM9PSJDbGFkb3NlbGFjaGUiKSAlPiUNCiAgbXV0YXRlKFVKUD1yYW1hbnVqYW4uYXBwcm94KG1vdXRoX2xlbmd0aCoyLG1vdXRoX3dpZHRoKS8yKSU+JQ0KICBhdWdtZW50KGZpdC5hcHByb3hfVUpQX29sZCxuZXdkYXRhPS4saW50ZXJ2YWw9InByZWRpY3QiKSAlPiUNCiAgbXV0YXRlKGFjcm9zcyguZml0dGVkOi51cHBlcix+ZXhwKC4pKnJlZ3Jlc3Npb24uc3RhdHMoZml0LmFwcHJveF9VSlBfb2xkKSRDRikpICU+JQ0KICByZW5hbWVfYXQodmFycyhzdGFydHNfd2l0aCgnLicpKSwgZnVucyhwYXN0ZTAoImZpdDEiLC4pKSklPiUNCiAgYXVnbWVudChmaXQuYXBwcm94X1VKUCxuZXdkYXRhPS4saW50ZXJ2YWw9InByZWRpY3QiKSAlPiUNCiAgcmVuYW1lX2F0KHZhcnMoc3RhcnRzX3dpdGgoJy4nKSksIGZ1bnMocGFzdGUwKCJmaXQyIiwuKSkpJT4lDQogIG11dGF0ZShhY3Jvc3MoZml0Mi5maXR0ZWQ6Zml0Mi51cHBlcix+ZXhwKC4pKnJlZ3Jlc3Npb24uc3RhdHMoZml0LmFwcHJveF9VSlApJENGKSwNCiAgICAgICAgIGZpdF9mZXJyb249cHJlZGljdChmaXQuVUpQLC4pLA0KICAgICAgICAgZml0MS5yYW5nZT1wYXN0ZTAoIigiLHJvdW5kKGZpdDEubG93ZXIsMSksIuKAkyIscm91bmQoZml0MS51cHBlciwxKSwiKSIpLA0KICAgICAgICAgZml0Mi5yYW5nZT1wYXN0ZTAoIigiLHJvdW5kKGZpdDIubG93ZXIsMSksIuKAkyIscm91bmQoZml0Mi51cHBlciwxKSwiKSIpKSU+JQ0KICBtdXRhdGUoUEUxPTEwMCooZml0MS5maXR0ZWQtdG90YWxfbGVuZ3RoKS9maXQxLmZpdHRlZCwNCiAgICAgICAgIFBFMj0xMDAqKGZpdDIuZml0dGVkLXRvdGFsX2xlbmd0aCkvZml0Mi5maXR0ZWQpICU+JQ0KICBzZWxlY3QodGF4b24sc3BlY2ltZW4sdG90YWxfbGVuZ3RoLA0KICAgICAgICAgZml0MS5maXR0ZWQsZml0MS5yYW5nZSxQRTEsDQogICAgICAgICBmaXQyLmZpdHRlZCxmaXQyLnJhbmdlLFBFMikgJT4lDQogIGthYmxlKGNhcHRpb249IkVzdGltYXRlZCBsZW5ndGggb2YgKkNsYWRvc2VsYWNoZSogdXNpbmcgZXN0aW1hdGVkIFVKUC4gUEUgcmVwcmVzZW50cyBlcnJvciByZWxhdGl2ZSB0byBtZWFzdXJlZCB0b3RhbCBsZW5ndGgiLA0KICAgICAgICBkaWdpdHM9YygxLDEsMSwxLDEsMiwxLDEsMiksDQogICAgICAgIGFsaWduPWMoImwiLCJjIiwiYyIsImMiLCJjIiwiYyIsImMiLCJjIiwiYyIpLA0KICAgICAgICBjb2wubmFtZXMgPSBjKCJUYXhvbiIsIlNwZWNpbWVuIiwiVG90YWwgTGVuZ3RoIiwNCiAgICAgICAgICAgICAgICAgICAgICAiRXN0LiIsIjk1JSBQLkkuIiwiUEUiLA0KICAgICAgICAgICAgICAgICAgICAgICJFc3QuIiwiOTUlIFAuSS4iLCJQRSIpKSU+JQ0KICBhZGRfaGVhZGVyX2Fib3ZlKGMoIiAiPTMsIlVzaW5nIEFsbCBTaGFya3MiPTMsIlByZWRhdG9yeSBDYXJjaGFyaGluaWZvcm1lcywgTGFtbmlmb3JtZXMsIGFuZCBIZXhhbmNoaWlmb3JtZXMgT25seSI9MykpJT4lDQogIGNvbHVtbl9zcGVjKDEsIGl0YWxpYyA9IFQpJT4lDQogIGthYmxlX3N0eWxpbmcoKQ0KYGBgDQoNCiMjIFNjYXR0ZXJwbG90IG9mIFVKUCB2ZXJzdXMgdG90YWwgbGVuZ3RoDQoNCmBgYHtyLGZpZy5jYXA9IlNjYXR0ZXIgcGxvdCBvZiBlc3RpbWF0ZXMgVUpQIHZlcnN1cyB0b3RhbCBsZW5ndGggaW4gZmlzaGVzIn0NCmdncGxvdChkYXRhX21vdXRocyAlPiUgDQogICAgICAgICBtdXRhdGUoVUpQPXJhbWFudWphbi5hcHByb3gobW91dGhfbGVuZ3RoKjIsbW91dGhfd2lkdGgpLzIsDQogICAgICAgICAgICAgICAgY2xhZGU9aWZlbHNlKGNsYWRlICVpbiUgYygiQWN0aW5vcHRlcnlnaWkiLCJTYXJjb3B0ZXJ5Z2lpIiksIk9zdGVpY2h0aHllcyIsY2xhZGUpKSAlPiUNCiAgICAgICAgIGRyb3BfbmEoVUpQLHRvdGFsX2xlbmd0aCkgJT4lDQogICAgICAgICBncm91cF9ieShjbGFkZSksDQogICAgICAgYWVzKHRvdGFsX2xlbmd0aCxVSlApKSArDQogIGdlb21fc3RhcihkYXRhPS4lPiVmaWx0ZXIoY2xhZGU9PSJPc3RlaWNodGh5ZXMiKSxhZXMoc3RhcnNoYXBlPWNsYWRlLGZpbGw9Y2xhZGUsY29sb3I9Y2xhZGUpKSArDQogIGdlb21fc3RhcihkYXRhPS4lPiVmaWx0ZXIoY2xhZGU9PSJDaG9uZHJpY2h0aHllcyIpLGFlcyhzdGFyc2hhcGU9Y2xhZGUsZmlsbD1jbGFkZSxjb2xvcj1jbGFkZSkpICsNCiAgZ2VvbV9zZWdtZW50KGRhdGE9LiU+JWZpbHRlcihnZW51cz09IkNvY2Nvc3RldXMiKSwNCiAgICAgICAgICAgICAgIGFlcyh5PVVKUCx5ZW5kPVVKUCs0LHg9dG90YWxfbGVuZ3RoLHhlbmQ9dG90YWxfbGVuZ3RoLTI0KSxhbHBoYT0wLjc1KSsNCiAgZ2VvbV9zdGFyKGRhdGE9LiU+JWZpbHRlcihjbGFkZT09IlBsYWNvZGVybWkiKSxhZXMoc3RhcnNoYXBlPWNsYWRlLGZpbGw9Y2xhZGUsY29sb3I9Y2xhZGUpKSArDQogIGdlb21fc3RhcihkYXRhPS4lPiVmaWx0ZXIoY2xhZGU9PSJQbGFjb2Rlcm1pIiksYWVzKHN0YXJzaGFwZT1jbGFkZSxmaWxsPWNsYWRlLGNvbG9yPWNsYWRlKSxzaXplPTIuNSxzaG93LmxlZ2VuZD1GKSArDQogIHNjYWxlX2NvbG9yX21hbnVhbCh2YWx1ZXM9YygiYmxhY2siLCJkYXJrIGdyZXkiLCJibGFjayIpKSsNCiAgc2NhbGVfZmlsbF9tYW51YWwodmFsdWVzPWMoaHVlX3BhbCgpKDEpLCJsaWdodCBncmV5Iiwid2hpdGUiKSkrDQogIHNjYWxlX3N0YXJzaGFwZV9tYW51YWwodmFsdWVzPWMoMTMsMTUsMSkpKw0KICBnZW9tX3RleHQoZGF0YT0uICU+JSBmaWx0ZXIoZ2VudXM9PSJDb2Njb3N0ZXVzIiksaGp1c3Q9MSxmb250ZmFjZT0zLA0KICAgICAgICAgICAgYWVzKGxhYmVsPWdlbnVzLHg9dG90YWxfbGVuZ3RoLTI1LHk9VUpQKzUpKSsNCiAgc2NhbGVfeF9jb250aW51b3VzKHRyYW5zPSJsb2cxMCIpICsNCiAgc2NhbGVfeV9jb250aW51b3VzKHRyYW5zPSJsb2cxMCIpICsNCiAgbGFicyh4PSJUb3RhbCBMZW5ndGggKGNtKSIseT0iRXN0aW1hdGVkIFVwcGVyIEphdyBQZXJpbWV0ZXIgKGNtKSIsDQogICAgICAgc3RhcnNoYXBlPSJDbGFkZSIsZmlsbD0iQ2xhZGUiLGNvbG9yPSJDbGFkZSIpKw0KICB0aGVtZShsZWdlbmQucG9zaXRpb24gPSBjKDAuOCwwLjIpKQ0KYGBgDQoNCiMjIFJlbGF0aXZlIHNpemUgb2YgVUpQIGluICpDb2Njb3N0ZXVzKg0KDQpgYGB7cn0NCmRhdGFfbW91dGhzICU+JSANCiAgbXV0YXRlKFVKUD1pZmVsc2UoaXMubmEoVUpQKSwNCiAgICAgICAgICAgICAgICAgICAgcmFtYW51amFuLmFwcHJveChtb3V0aF9sZW5ndGgqMixtb3V0aF93aWR0aCkvMiwNCiAgICAgICAgICAgICAgICAgICAgVUpQKSkgJT4lDQogIGRyb3BfbmEoVUpQKSAlPiUNCiAgZ3JvdXBfYnkodGF4b24pICU+JQ0KICBzdW1tYXJpc2UoVUpQPW1lYW4oVUpQKSxjbGFkZT11bmlxdWUoY2xhZGUpLHRvdGFsX2xlbmd0aD1tZWFuKHRvdGFsX2xlbmd0aCkpICU+JQ0KICBtdXRhdGUoYFVKUC90b3RhbF9sZW5ndGhgPVVKUC90b3RhbF9sZW5ndGgpICU+JQ0KICBhcnJhbmdlKGBVSlAvdG90YWxfbGVuZ3RoYCkgJT4lDQogIGZpbHRlcihgVUpQL3RvdGFsX2xlbmd0aGAgPCAwLjI3NSAmIGBVSlAvdG90YWxfbGVuZ3RoYCA+IDAuMjI1KSAlPiUNCiAgc2VsZWN0KHRheG9uLGNsYWRlLGBVSlAvdG90YWxfbGVuZ3RoYCkgJT4lDQogIGthYmxlKGNvbC5uYW1lcyA9IGMoIlRheG9uIiwiQ2xhZGUiLCJVSlAvVG90YWwgTGVuZ3RoIiksZGlnaXRzPTMsDQogICAgICAgIGNhcHRpb249IlByb3BvcnRpb25hbCBVSlAgaW4gKkNvY2Nvc3RldXMqIGNvbXBhcmVkIHdpdGggdGF4YSBvZiBzaW1pbGFyIGVzdGltYXRlZCBVSlAiKSU+JQ0KICBjb2x1bW5fc3BlYygxLGl0YWxpYz1UKSAlPiUNCiAga2FibGVfc3R5bGluZygpDQpgYGANCg0KIyBEaWFnbm9zdGljIHBsb3RzIGZvciBVSlAgYW5kIG1vdXRoIHdpZHRoDQoNCiMjIFVKUA0KDQpgYGB7cixmaWcud2lkdGg9MTAsZmlnLmhlaWdodD03LjUsZmlnLmNhcD0iRGlhZ25vc3RpYyBwbG90cyBmb3IgbW9kZWwgcmVncmVzc2luZyB0b3RhbCBsZW5ndGggYWdhaW5zdCBVSlAifQ0KcGFyKG1mcm93PWMoMiwyKSkNCnBsb3QoZml0LlVKUCkNCmBgYA0KDQpCYXNlZCBvbiB0aGlzIFVKUCBzaG93cyBzb21lIHNsaWdodCBoZXRlcm9za2VkYXN0aWNpdHksIGFuZCB0aGUgcmVzaWR1YWxzIHNlZW0gdG8gYmUgc2xpZ2h0bHkgbm9uLW5vcm1hbGx5IGRpc3RyaWJ1dGVkLg0KDQojIyBNb3V0aCBXaWR0aA0KDQpgYGB7cixmaWcud2lkdGg9MTAsZmlnLmhlaWdodD03LjUsZmlnLmNhcD0iRGlhZ25vc3RpYyBwbG90cyBmb3IgbW9kZWwgcmVncmVzc2luZyB0b3RhbCBsZW5ndGggYWdhaW5zdCBtb3V0aCB3aWR0aCJ9DQpwYXIobWZyb3c9YygyLDIpKQ0KcGxvdChmaXQubW91dGh3aWR0aCkNCmBgYA0KDQoNCiMgUGxvdHRpbmcgYWdhaW5zdCBoZWFkIGxlbmd0aA0KDQpgYGB7cn0NCmRhdGFfbW91dGhzPC1kYXRhX21vdXRocyU+JQ0KICBtdXRhdGUodG90YWxfbGVuZ3RoPWlmZWxzZShnZW51cyE9IkR1bmtsZW9zdGV1cyIsDQogICAgICAgICAgICAgICAgICAgICAgICAgICAgIHRvdGFsX2xlbmd0aCwNCiAgICAgICAgICAgICAgICAgICAgICAgICAgICAgcHJlZGljdChmaXQuVUpQLC4pKSkNCmBgYA0KDQoocmVmOmhlYWRsZW5ndGgpIFBsb3Qgb2YgbG9nMTAgaGVhZCBsZW5ndGggYWdhaW5zdCBsb2cxMCB0b3RhbCBsZW5ndGggaW4gZmlzaGVzLCBzaG93aW5nIGhvdyB0aGUgbGVuZ3RoIGVzdGltYXRlcyBvZiBGZXJyb24gZXQgYWwuICgyMDE3KSByZXN1bHQgaW4gdW51c3VhbGx5IHNtYWxsIGhlYWQgcHJvcG9ydGlvbnMgZm9yICpEdW5rbGVvc3RldXMqICh5ZWxsb3cgc3RhcnMpIGNvbXBhcmVkIHRvIG90aGVyIGZpc2hlcy4gRmlzaGVzIGluIHRoaXMgZ3JhcGggYXJlIGNvbG9yLWNvZGVkIGJ5IGJvZHkgc2hhcGUgY2F0ZWdvcmllcy4gRGFzaGVkIGxpbmUgcmVwcmVzZW50cyB0aGUgcG9pbnQgYXQgd2hpY2ggdGF4YSBiZWxvdyB0aGlzIHBvaW50IHR5cGljYWxseSBleGhpYml0IGV4dHJlbWVseSBlbG9uZ2F0ZSB0cnVuayBwcm9wb3J0aW9ucyAoZS5nLiwgYW5ndWlsbGlmb3JtIHRheGEsICpDaGVpcm9jZW50cnVzKiBzcHAuLCAqQ29yeXBoYWVuYSogc3BwLiksIG9yIGVsc2UgaGF2ZSBleHRyZW1lbHkgZWxvbmdhdGUgY2F1ZGFsIGZpbnMgdGhhdCBjb250cmlidXRlIGdyZWF0bHkgdG8gdG90YWwgbGVuZ3RoIChlLmcuLCAqQWxvcGlhcyosIENoaW1hZXJpZm9ybWVzKS4NCg0KYGBge3IsZmlnLmNhcD0iKHJlZjpoZWFkbGVuZ3RoKSIsZmlnLmFzcD0wLjY1LGZpZy53aWR0aD05fQ0KZ2dwbG90KGRhdGFfbW91dGhzICU+JQ0KICAgICAgICAgZHJvcF9uYShoZWFkX2xlbmd0aCx0b3RhbF9sZW5ndGgsc2hhcGUpJT4lDQogICAgICAgICBmaWx0ZXIoY2xhZGUgJWluJSBjKCJBY3Rpbm9wdGVyeWdpaSIsIlNhcmNvcHRlcnlnaWkiLA0KICAgICAgICAgICAgICAgICAgICAgICAgICAgICAiUGxhY29kZXJtaSIsIkNob25kcmljaHRoeWVzIikpJT4lDQogICAgICAgICBtdXRhdGUocGVyY2VudF9oZWFkX2xlbmd0aD1oZWFkX2xlbmd0aC90b3RhbF9sZW5ndGgpLA0KICAgICAgIGFlcyh0b3RhbF9sZW5ndGgsaGVhZF9sZW5ndGgvdG90YWxfbGVuZ3RoKSkrDQogIGdlb21fc3RhcihhZXMoc3RhcnNoYXBlPWNsYWRlLGZpbGw9c2hhcGUpKSsNCiAgc2NhbGVfc3RhcnNoYXBlX21hbnVhbCh2YWx1ZXM9YygxNSwxMywxLDI4KSkrDQogIGdlb21fc3RhcihkYXRhPS4gJT4lIGZpbHRlcihjbGFkZT09IlBsYWNvZGVybWkiLGdlbnVzIT0iRHVua2xlb3N0ZXVzIiksDQogICAgICAgICAgICBzdGFyc2hhcGU9MSxzaXplPTMuNSxjb2xvcj0id2hpdGUiLGZpbGw9ImJsYWNrIixzdGFyc3Ryb2tlPTAuNSkrDQogIGdlb21faGxpbmUoeWludGVyY2VwdD0wLjE3LGxpbmV0eXBlPSJkYXNoZWQiKSsNCiAgZ2VvbV9zdGFyKGRhdGE9LiAlPiUgZmlsdGVyKGdlbnVzPT0iRHVua2xlb3N0ZXVzIiksDQogICAgICAgICAgICBzdGFyc2hhcGU9MSxzaXplPTMuNSxmaWxsPSJ5ZWxsb3ciKSsNCiAgYW5ub3RhdGUoZ2VvbT0idGV4dCIseD01LHk9MC4xNixsYWJlbD0iRWVsLUxpa2UgVGF4YSIpKw0KICBhbm5vdGF0ZShnZW9tPSJ0ZXh0Iix4PTQ4MCx5PTAuMDg1LGhqdXN0PTEsbGFiZWw9IkR1bmtsZW9zdGV1cyIsZm9udGZhY2U9MykrDQogIGFubm90YXRlKGdlb209InRleHQiLHg9OTc1LHk9MC4wNixoanVzdD0xLGxhYmVsPSJSZWdhbGVjdXMiLGZvbnRmYWNlPTMpKw0KICBzY2FsZV9maWxsX21hbnVhbCh2YWx1ZXM9YyhodWVfcGFsKCkoNSksImdyYXkiKSkrDQogIHNjYWxlX3hfY29udGludW91cyh0cmFucz0ibG9nMTAiKSsNCiAgc2NhbGVfeV9jb250aW51b3VzKGxhYmVscz1zY2FsZXM6OnBlcmNlbnRfZm9ybWF0KGFjY3VyYWN5PTEpKSsNCiAgbGFicyh4PSJUb3RhbCBMZW5ndGggKGNtKSIseT0iUGVyY2VudCBIZWFkIExlbmd0aCIsZmlsbD0iQm9keSBTaGFwZSIsc3RhcnNoYXBlPSJDbGFkZSIpKw0KICB0aGVtZV9jbGFzc2ljKCkrDQogIGd1aWRlcyhmaWxsPWd1aWRlX2xlZ2VuZChucm93PTIsb3ZlcnJpZGUuYWVzID0gbGlzdChzdGFyc2hhcGU9MTUpKSwNCiAgICAgICAgIHN0YXJzaGFwZT1ndWlkZV9sZWdlbmQobnJvdz0yKSkrDQogIHRoZW1lKGxlZ2VuZC5wb3NpdGlvbj0iYm90dG9tIixsZWdlbmQuYm94ID0gImhvcml6b250YWwiKQ0KYGBgDQoNClBsb3R0aW5nIGhlYWQgbGVuZ3RoIGFzIGEgcGVyY2VudCBvZiB0b3RhbCBsZW5ndGggcmVzdWx0cyBpbiAqRHVua2xlb3N0ZXVzKiAodXNpbmcgdGhlIGVzdGltYXRlcyBvZiBGZXJyw7NuIGV0IGFsLiAyMDE3KSBwbG90dGluZyBmYXIgb3V0c2lkZSB0aGUgbW9ycGhvc3BhY2UgZm9ybWVkIGJ5IGFsbW9zdCBhbGwgbGl2aW5nIGZpc2hlcy4gTm90YWJseSwgb3RoZXIgYXJ0aHJvZGlyZXMgZG8gbm90IHNob3cgaGVhZC1ib2R5IHByb3BvcnRpb25zIHNpbWlsYXIgdG8gdGhlIGVzdGltYXRlZCB2YWx1ZXMgZm9yICpEdW5rbGVvc3RldXMqIGJ5IEZlcnLDs24gZXQgYWwuICgyMDE3KSwgaW5zdGVhZCB0aGV5IHBsb3Qgd2VsbCB3aXRoaW4gdGhlIHJhbmdlIG9mIHZhcmlhdGlvbiBvZiBleHRhbnQgZnVzaWZvcm0gZmlzaGVzLiAqSG9sb25lbWEqIGFuZCAqQW1hemljaHRoeXMqIHBsb3QgYW1vbmcgZXh0YW50IHRheGEgd2l0aCBlbG9uZ2F0ZSB0cnVua3MgKGUuZy4sICpDb3J5cGhhZW5hKiwgKkNoZWlyb2NlbnRydXMqLCBzb21lIGdlbXB5bGlkcywgZXRjLiksIGJ1dCBzdGlsbCBkbyBub3Qgc2hvdyBwcm9wb3J0aW9ucyBhcyBleHRyZW1lIGFzIEZlcnLDs24gZXQgYWwuICgyMDE3KSBpbXBsaWVzIGZvciAqRHVua2xlb3N0ZXVzKi4NCg0KIyMgSGlnaGxpZ2h0aW5nIHRheGEgd2l0aCBlbG9uZ2F0ZSB0cnVua3MNCg0KKHJlZjplbG9uZ2F0ZSkgUGxvdCBvZiBwZXJjZW50IGhlYWQgbGVuZ3RoIGFnYWluc3QgdG90YWwgbGVuZ3RoIGluIGZpc2hlcy4gVGhpcyBncmFwaCBzaG93cyBob3cgdGhlIGVzdGltYXRlcyBvZiBGZXJyb24gZXQgYWwuICgyMDE3KSByZXN1bHQgaW4gdW51c3VhbGx5IHNtYWxsIGhlYWQgcHJvcG9ydGlvbnMgZm9yICpEdW5rbGVvc3RldXMqIGNvbXBhcmVkIHRvIG90aGVyIGZpc2hlcy4gVGF4YSBiZWxvdyB0aGUgZGFzaGVkIGxpbmUgdHlwaWNhbGx5IGV4aGliaXQgaGlnaGx5IGVsb25nYXRlIHRydW5rcyAoZS5nLiwgYW5ndWlsbGlmb3JtIHRheGEsICpDaGVpcm9jZW50cnVzKiBzcHAuLCAqQ29yeXBoYWVuYSogc3BwLiksIG9yIGVsc2UgaGF2ZSBleHRyZW1lbHkgZWxvbmdhdGUgY2F1ZGFsIGZpbnMgdGhhdCBjb250cmlidXRlIGdyZWF0bHkgdG8gdG90YWwgbGVuZ3RoIChlLmcuLCAqQWxvcGlhcyosIENoaW1hZXJpZm9ybWVzKS4gKlJlZ2FsZWN1cyogc2lsaG91ZXR0ZSBtb2RpZmllZCBmcm9tIGltYWdlIGJ5IEpvaG4gTm9ycmlzIFdvb2QsICpDb3J5cGhhZW5hKiBzaWxob3VldHRlIGZyb20gaW1hZ2UgYnkgUmljaGFyZCBXaW50ZXJib3R0b20sIGFuZCAqUnV2ZXR0dXMqIGFuZCAqRXBpbmVwaGVsdXMqIHNpbGhvdWV0dGVzIG1vZGlmaWVkIGZyb20gaW1hZ2VzIGluIFJhbmRhbGwgKDE5OTcpLg0KDQpgYGB7cixmaWcuY2FwPSIocmVmOmVsb25nYXRlKSIsZmlnLndpZHRoPTYuNyxmaWcuYXNwPTAuODB9DQpkdW5rbGVvc3RldXNfcGVyY2VudF9oZWFkPC1nZ3Bsb3QoDQogIGRhdGFfbW91dGhzICU+JQ0KICAgIGRyb3BfbmEoaGVhZF9sZW5ndGgsdG90YWxfbGVuZ3RoKSU+JQ0KICAgIG11dGF0ZShwZXJjZW50X2hlYWRfbGVuZ3RoPWhlYWRfbGVuZ3RoL3RvdGFsX2xlbmd0aCwNCiAgICAgICAgICAgZWxvbmdhdGU9aWZlbHNlKHNoYXBlICVpbiUgYygiYW5ndWlsbGlmb3JtIiwibWFjcnVyaWZvcm0iKXwNCiAgICAgICAgICAgICAgICAgICAgICAgICAgICAgZ2VudXMgJWluJSBjKCJDaGVpcm9jZW50cnVzIiwiQ29yeXBoYWVuYSIsIlRldHJhcHR1cnVzIiwNCiAgICAgICAgICAgICAgICAgICAgICAgICAgICAgICAgICAgICAgICAgICJQb2x5cHRlcnVzIiwiQWxlcGlzYXVydXMiLCJHb25vcnluY2h1cyIsDQogICAgICAgICAgICAgICAgICAgICAgICAgICAgICAgICAgICAgICAgICAiUmhhcGhpb2RvbiIsDQogICAgICAgICAgICAgICAgICAgICAgICAgICAgICAgICAgICAgICAgICAiVGh5cnNpdG9pZGVzIiwiR2VtcHlsdXMiLCJUaHlyc2l0ZXMiKXwNCiAgICAgICAgICAgICAgICAgICAgICAgICAgICAgZmFtaWx5ICVpbiUgIkJlbG9uaWRhZSIsDQogICAgICAgICAgICAgICAgICAgICAgICAgICAiRWxvbmdhdGUgUG9zdGNyYW5pdW0iLCJOb3QgRWxvbmdhdGUiKSklPiUNCiAgICBhcnJhbmdlKGVsb25nYXRlKSwNCiAgYWVzKHRvdGFsX2xlbmd0aCxoZWFkX2xlbmd0aC90b3RhbF9sZW5ndGgpKSsNCiAgZ2VvbV9zZWdtZW50KGRhdGE9LiU+JWZpbHRlcihzcGVjaW1lbj09IkNNTkggNjA5MCIpLA0KICAgICAgICAgICAgICAgYWVzKHhlbmQ9dG90YWxfbGVuZ3RoKjAuOCx5ZW5kPTAuOSpoZWFkX2xlbmd0aC90b3RhbF9sZW5ndGgpLGNvbG9yPSJibGFjayIpKw0KICBnZW9tX3NlZ21lbnQoZGF0YT0uJT4lZmlsdGVyKGdlbnVzPT0iUmVnYWxlY3VzIix0b3RhbF9sZW5ndGg9PTM2NyksDQogICAgICAgICAgICAgICBhZXMoeGVuZD0xMjUwLHllbmQ9cGVyY2VudF9oZWFkX2xlbmd0aC0wLjAwMSkpKw0KICBnZW9tX3N0YXIoYWVzKHN0YXJzaGFwZT1jbGFkZSxmaWxsPWVsb25nYXRlKSkrDQogIGdlb21fc3RhcihkYXRhPS4lPiVmaWx0ZXIoZWxvbmdhdGU9PSJFbG9uZ2F0ZSBQb3N0Y3Jhbml1bSIpLA0KICAgICAgICAgICAgYWVzKHN0YXJzaGFwZT1jbGFkZSxmaWxsPWVsb25nYXRlKSkrDQogIHNjYWxlX3N0YXJzaGFwZV9tYW51YWwodmFsdWVzPWMoMTUsMTMsMSwyOCwxMSkpKw0KICBnZW9tX3NlZ21lbnQoYWVzKHg9NzAuNjUseT0wLjE1NTQseGVuZD0xMjUwLHllbmQ9MC4xNiksc2l6ZT0wLjQpKw0KICBnZW9tX3NlZ21lbnQoYWVzKHg9NzEuNCx5PTAuMjgwMyx4ZW5kPTEyNTAseWVuZD0wLjMyNSksc2l6ZT0wLjQpKw0KICBnZW9tX3NlZ21lbnQoYWVzKHg9OTYuOCx5PTAuMjI3OCx4ZW5kPTEyNTAseWVuZD0wLjI0KSxzaXplPTAuNCkrDQogIGdlb21fc3RhcihkYXRhPS4lPiUNCiAgICAgICAgICAgICAgZmlsdGVyKGdlbnVzPT0iQ29yeXBoYWVuYSImdG90YWxfbGVuZ3RoPT03MC42NXwNCiAgICAgICAgICAgICAgICAgICAgIHRheG9uPT0iRXBpbmVwaGVsdXNfbWFsYWJhcmljdXMiJnRvdGFsX2xlbmd0aD09NzEuNHwNCiAgICAgICAgICAgICAgICAgICAgIGdlbnVzPT0iUnV2ZXR0dXMiJnRvdGFsX2xlbmd0aD09OTYuOCksDQogICAgICAgICAgICBhZXMoc3RhcnNoYXBlPWNsYWRlLGZpbGw9ZWxvbmdhdGUpKSsNCiAgZ2VvbV9zdGFyKGRhdGE9LiU+JWZpbHRlcihjbGFkZT09IlBsYWNvZGVybWkiKSxzdGFyc2hhcGU9MSxzaXplPTQsDQogICAgICAgICAgICBjb2xvcj0id2hpdGUiLGZpbGw9ImJsYWNrIikrDQogIGdlb21faGxpbmUoeWludGVyY2VwdD0wLjE3LGxpbmV0eXBlPSJkYXNoZWQiKSsNCiAgZ2VvbV9zdGFyKGRhdGE9LiAlPiUgZmlsdGVyKGdlbnVzPT0iRHVua2xlb3N0ZXVzIiksDQogICAgICAgICAgICBzdGFyc2hhcGU9MSxzaXplPTQsZmlsbD0ieWVsbG93Iixjb2xvcj0iYmxhY2siKSsNCiAgYW5ub3RhdGUoZ2VvbT0idGV4dCIseD0yLHk9MC4xNixzaXplPTMsbGFiZWw9IkVlbC1MaWtlIFRheGEiKSsNCiAgYW5ub3RhdGUoZ2VvbT0idGV4dCIseD00ODAseT0wLjA4LGhqdXN0PTEsbGFiZWw9IkR1bmtsZW9zdGV1cyIsDQogICAgICAgICAgIHNpemU9Myxmb250ZmFjZT0zKSsNCiAgYW5ub3RhdGUoZ2VvbT0idGV4dCIseD00MzUwLHk9MC4wMzUsaGp1c3Q9MSxsYWJlbD0iUmVnYWxlY3VzIiwNCiAgICAgICAgICAgc2l6ZT0zLGZvbnRmYWNlPTMpKw0KICBhbm5vdGF0ZShnZW9tPSJ0ZXh0Iix4PTQzNTAseT0wLjE0LGhqdXN0PTEsbGFiZWw9IkNvcnlwaGFlbmEiLA0KICAgICAgICAgICBzaXplPTMsZm9udGZhY2U9MykrDQogIGFubm90YXRlKGdlb209InRleHQiLHg9NDM1MCx5PTAuMjIsaGp1c3Q9MSxsYWJlbD0iUnV2ZXR0dXMiLA0KICAgICAgICAgICBzaXplPTMsZm9udGZhY2U9MykrDQogIGFubm90YXRlKGdlb209InRleHQiLHg9NDM1MCx5PTAuMzA1LGhqdXN0PTEsbGFiZWw9IkVwaW5lcGhlbHVzIiwNCiAgICAgICAgICAgc2l6ZT0zLGZvbnRmYWNlPTMpKw0KICBjb29yZF9jYXJ0ZXNpYW4oeGxpbT1jKDEsMjkwMCkseWxpbT1jKDAuMDQsMC4zOSkpKw0KICBzY2FsZV94X2NvbnRpbnVvdXModHJhbnM9ImxvZzEwIikrDQogIHNjYWxlX3lfY29udGludW91cyhsYWJlbHM9c2NhbGVzOjpwZXJjZW50X2Zvcm1hdChhY2N1cmFjeT0xKSkrDQogIGxhYnMoeD0iVG90YWwgTGVuZ3RoIChjbSkiLHk9IlBlcmNlbnQgSGVhZCBMZW5ndGgiLGZpbGw9IkJvZHkgU2hhcGUiLHN0YXJzaGFwZT0iQ2xhZGUiKSsNCiAgdGhlbWVfY293cGxvdCgpKw0KICBndWlkZXMoZmlsbD1ndWlkZV9sZWdlbmQobnJvdz0yLG92ZXJyaWRlLmFlcyA9IGxpc3Qoc3RhcnNoYXBlPTE1KSksDQogICAgICAgICBzdGFyc2hhcGU9Z3VpZGVfbGVnZW5kKG5jb2w9MixvdmVycmlkZS5hZXMgPSBsaXN0KGZpbGw9ImxpZ2h0IGdyYXkiKSkpKw0KICB0aGVtZShsZWdlbmQucG9zaXRpb249ImJvdHRvbSIsDQogICAgICAgIGxlZ2VuZC50ZXh0PWVsZW1lbnRfdGV4dChzaXplPTkpLA0KICAgICAgICBsZWdlbmQudGl0bGU9ZWxlbWVudF90ZXh0KHNpemU9MTApKQ0KDQooZHVua2xlb3N0ZXVzX3BlcmNlbnRfaGVhZF9yZWdhbGVjdXM8LWdnZHJhdyhkdW5rbGVvc3RldXNfcGVyY2VudF9oZWFkKSArDQogIGRyYXdfaW1hZ2UoIkFydGhyb2RpcmUgTW91dGhzIFN1cHBsZW1lbnRhcnkgRmlndXJlIDQgKEVwaW5lcGhlbHVzKS5wbmciLA0KICAgICAgICAgICAgIHg9MC40MTUseT0wLjMzNSxzY2FsZT0wLjE1KSsNCiAgZHJhd19pbWFnZSgiQXJ0aHJvZGlyZSBNb3V0aHMgU3VwcGxlbWVudGFyeSBGaWd1cmUgMiAoQ29yeXBoYWVuYSkucG5nIiwNCiAgICAgICAgICAgICB4PTAuNDE1LHk9LS4wMSxzY2FsZT0wLjE1KSsNCiAgZHJhd19pbWFnZSgiQXJ0aHJvZGlyZSBNb3V0aHMgU3VwcGxlbWVudGFyeSBGaWd1cmUgMyAoUnV2ZXR0dXMpLnBuZyIsDQogICAgICAgICAgICAgeD0wLjQxNSx5PS4xNTUsc2NhbGU9MC4xNSkrDQogIGRyYXdfaW1hZ2UoIkFydGhyb2RpcmUgTW91dGhzIFN1cHBsZW1lbnRhcnkgRmlndXJlIDEgKFJlZ2FsZWN1cykucG5nIiwNCiAgICAgICAgICAgICB4PTAuNDE1LHk9LS4xOTUsc2NhbGU9MC4xNSkpDQoNCmdnc2F2ZSgiQXJ0aHJvZGlyZSBNb3V0aHMgRmlndXJlIDUgKFBlcmNlbnQgSGVhZCBEdW5rbGVvc3RldXMpLnBkZiIsDQogICAgICAgZHVua2xlb3N0ZXVzX3BlcmNlbnRfaGVhZF9yZWdhbGVjdXMsZGV2aWNlPSJwZGYiLA0KICAgICAgIHVuaXRzPSJtbSIsd2lkdGg9MTY1LGhlaWdodD0xMzApDQpgYGANCg0KSW4gZmFjdCwgdGhlIGVzdGltYXRlcyBmcm9tIEZlcnLDs24gZXQgYWwuICgyMDE3KSByZXN1bHQgaW4gKkR1bmtsZW9zdGV1cyogcGxvdHRpbmcgaW4gYSByZWdpb24gb2YgbW9ycGhvc3BhY2Ugb25seSBvY2N1cGllZCBieSBoaWdobHkgZWxvbmdhdGUgZmlzaC4gSW5kZWVkLCB0aGUgcmVzdWx0cyBvZiBGZXJyw7NuIGV0IGFsLiAoMjAxNykgc3VnZ2VzdCBoZWFkLWJvZHkgcHJvcG9ydGlvbnMgdGhhdCBhcmUgbW9yZSBleHRyZW1lIHRoYW4gdGhlIG1ham9yaXR5IG9mIG1vc3QgZWxvbmdhdGUtYm9kaWVkIGZpc2hlcywgZXZlbiBtb3Jlc28gdGhhbiBncm91cHMgc3VjaCBhcyBBbmd1aWxsaWZvcm1lcy4gVGhlIG9ubHkgZXh0YW50IGZpc2hlcyB3aGljaCBzaG93IHByb3BvcnRpb25zIGNvbXBhcmFibGUgdG8gdGhlc2UgaW5mZXJyZWQgcHJvcG9ydGlvbnMgYXJlICpFbGVjdHJvcGhvcnVzKiBhbmQgKlJlZ2FsZWN1cyosIG1ha2luZyBpdCBoaWdobHkgdW5saWtlbHkgdGhhdCAqRHVua2xlb3N0ZXVzKiB3YXMgYXMgbG9uZyBhcyBwcmVkaWN0ZWQgYnkgRmVycsOzbiBldCBhbC4gKDIwMTcpIG9yIG1hbnkgcHJldmlvdXMgc3R1ZGllcyBvbiAqRHVua2xlb3N0ZXVzKiAoYXMgZXZlbiBtb3N0IHNtYWxsZXIgZXN0aW1hdGVzIGluIHByZXZpb3VzIHN0dWRpZXMgZm9yICpELiB0ZXJyZWxsaSogd291bGQgc3RpbGwgcmVzdWx0IGluIHRoaXMgdGF4b24gcGxvdHRpbmcgYW1vbmcgQW5ndWlsbGlmb3JtZXMgYW5kIHNpbWlsYXIgdGF4YSkuDQoNCiMjIFVzaW5nIHByZWNhdWRhbCBsZW5ndGgNCg0KUHJlY2F1ZGFsIGxlbmd0aCBmb3IgKkR1bmtsZW9zdGV1cyB0ZXJyZWxsaSogd2FzIGNhbGN1bGF0ZWQgYnkgdGFraW5nIHRoZSBsZW5ndGggb2YgdGhlIHVwcGVyIGxvYmUgb2YgdGhlIGNhdWRhbCBmaW4gZnJvbSBGZXJyw7NuIGV0IGFsLiAoMjAxNyksIG11bHRpcGx5aW5nIGJ5IHRoZSBjb3NpbmUgdG8gY2FsY3VsYXRlIHRoZSBhbnRlcm9wb3N0ZXJpb3IgbGVuZ3RoIG9mIHRoZSBmaW4gaW4gbmF0dXJhbCBwb3NpdGlvbiwgYW5kIHRoZW4gc3VidHJhY3RpbmcgdGhhdCBmcm9tIHRoZSB0b3RhbCBsZW5ndGguDQoNCihyZWY6cHJlY2F1ZGFsbGVuZ3RoKSBHcmFwaCBvZiBwZXJjZW50IGhlYWQgbGVuZ3RoIGFnYWluc3QgcHJlY2F1ZGFsIGxlbmd0aCBpbiBmaXNoZXMuIFRoaXMgc2hvd3MgaGVhZC1ib2R5IHByb3BvcnRpb25zIGJldHRlciBpbiB0YXhhIHdpdGggZXh0cmVtZWx5IGVsb25nYXRlIGNhdWRhbCBmaW5zIGxpa2UgKkFsb3BpYXMqIGFuZCAqU3RlZ29zdG9tYSosIGJ1dCBkb2VzIG5vdCBhbGxvdyBmb3IgY29tcGFyaXNvbnMgd2l0aCBhbmd1aWxsaWZvcm0gdGF4YSB0aGF0IGxhY2sgYSBkaXN0aW5jdCBjYXVkYWwgcmVnaW9uLg0KDQpgYGB7cixmaWcuY2FwPSIocmVmOnByZWNhdWRhbGxlbmd0aCkiLGZpZy53aWR0aD05LGZpZy5hc3A9MC42NX0NCmdncGxvdChkYXRhX21vdXRocyAlPiUNCiAgICAgICAgIGRyb3BfbmEoc2hhcGUpJT4lDQogICAgICAgICBmaWx0ZXIodG90YWxfbGVuZ3RoIT1wcmVjYXVkYWxfbGVuZ3RofGdlbnVzPT0iRHVua2xlb3N0ZXVzIiklPiUNCiAgICAgICAgIG11dGF0ZShwZXJjZW50X2hlYWRfbGVuZ3RoPWhlYWRfbGVuZ3RoL3ByZWNhdWRhbF9sZW5ndGgpLA0KICAgICAgIGFlcyhwcmVjYXVkYWxfbGVuZ3RoLGhlYWRfbGVuZ3RoL3ByZWNhdWRhbF9sZW5ndGgpKSsNCiAgZ2VvbV9zdGFyKGFlcyhzdGFyc2hhcGU9Y2xhZGUsZmlsbD1zaGFwZSkpKw0KICBzY2FsZV9zdGFyc2hhcGVfbWFudWFsKHZhbHVlcz1jKDE1LDEzLDEsMjgpKSsNCiAgZ2VvbV9zdGFyKGRhdGE9LiU+JWZpbHRlcihjbGFkZT09IlBsYWNvZGVybWkiLGdlbnVzIT0iRHVua2xlb3N0ZXVzIiksc3RhcnNoYXBlPTEsc2l6ZT01LjUsY29sb3I9IndoaXRlIixmaWxsPSJ3aGl0ZSIpKw0KICBnZW9tX3N0YXIoZGF0YT0uJT4lZmlsdGVyKGNsYWRlPT0iUGxhY29kZXJtaSIsZ2VudXMhPSJEdW5rbGVvc3RldXMiKSxzdGFyc2hhcGU9MSxzaXplPTMuNSxmaWxsPSJibGFjayIpKw0KICBnZW9tX2hsaW5lKHlpbnRlcmNlcHQ9MC4yMSxsaW5ldHlwZT0iZGFzaGVkIikrDQogIGdlb21fc3RhcihkYXRhPS4lPiUgZmlsdGVyKGdlbnVzPT0iRHVua2xlb3N0ZXVzIiklPiUNCiAgICAgICAgICAgICAgbXV0YXRlKHByZWNhdWRhbF9sZW5ndGg9aWZlbHNlKHNwZWNpbWVuPT0iQ01OSCA1NzY4IiwNCiAgICAgICAgICAgICAgICAgICAgICAgICAgICAgICAgICAgICAgICAgICAgIHRvdGFsX2xlbmd0aC1jb3MoMjgqKHBpLzE4MCkpKjE3MCwNCiAgICAgICAgICAgICAgICAgICAgICAgICAgICAgICAgICAgICAgICAgICAgIHByZWNhdWRhbF9sZW5ndGgpLA0KICAgICAgICAgICAgICAgICAgICAgcHJlY2F1ZGFsX2xlbmd0aD1pZmVsc2Uoc3BlY2ltZW49PSJDTU5IIDc0MjQiLA0KICAgICAgICAgICAgICAgICAgICAgICAgICAgICAgICAgICAgICAgICAgICAgdG90YWxfbGVuZ3RoLWNvcygyMioocGkvMTgwKSkqODAsDQogICAgICAgICAgICAgICAgICAgICAgICAgICAgICAgICAgICAgICAgICAgICBwcmVjYXVkYWxfbGVuZ3RoKSwNCiAgICAgICAgICAgICAgICAgICAgIHByZWNhdWRhbF9sZW5ndGg9aWZlbHNlKHNwZWNpbWVuPT0iQ01OSCA3MDU0IiwNCiAgICAgICAgICAgICAgICAgICAgICAgICAgICAgICAgICAgICAgICAgICAgIHRvdGFsX2xlbmd0aC1jb3MoMjcqKHBpLzE4MCkpKjE0MiwNCiAgICAgICAgICAgICAgICAgICAgICAgICAgICAgICAgICAgICAgICAgICAgIHByZWNhdWRhbF9sZW5ndGgpLA0KICAgICAgICAgICAgICAgICAgICAgcHJlY2F1ZGFsX2xlbmd0aD1pZmVsc2Uoc3BlY2ltZW49PSJDTU5IIDYwOTAiLA0KICAgICAgICAgICAgICAgICAgICAgICAgICAgICAgICAgICAgICAgICAgICAgdG90YWxfbGVuZ3RoLWNvcygyNioocGkvMTgwKSkqMTMyLA0KICAgICAgICAgICAgICAgICAgICAgICAgICAgICAgICAgICAgICAgICAgICAgcHJlY2F1ZGFsX2xlbmd0aCkpLA0KICAgICAgICAgICAgc3RhcnNoYXBlPTEsc2l6ZT0zLjUsZmlsbD0ieWVsbG93Iixjb2xvcj0iYmxhY2siKSsNCiAgYW5ub3RhdGUoZ2VvbT0idGV4dCIseD02ODAseT0wLjE1NSxoanVzdD0xLGxhYmVsPSJEdW5rbGVvc3RldXMiLGZvbnRmYWNlPTMpKw0KICBzY2FsZV9maWxsX21hbnVhbCh2YWx1ZXM9YyhodWVfcGFsKCkoNSksImdyYXkiKSkrDQogIHNjYWxlX3hfY29udGludW91cyh0cmFucz0ibG9nMTAiKSsNCiAgc2NhbGVfeV9jb250aW51b3VzKGxhYmVscz1zY2FsZXM6OnBlcmNlbnRfZm9ybWF0KGFjY3VyYWN5PTEpKSsNCiAgbGFicyh4PSJQcmVjYXVkYWwgTGVuZ3RoIChjbSkiLHk9IlBlcmNlbnQgSGVhZCBMZW5ndGgiLGZpbGw9IkJvZHkgU2hhcGUiLHN0YXJzaGFwZT0iQ2xhZGUiKSsNCiAgdGhlbWVfY2xhc3NpYygpKw0KICBndWlkZXMoZmlsbD1ndWlkZV9sZWdlbmQobnJvdz0yLG92ZXJyaWRlLmFlcyA9IGxpc3Qoc3RhcnNoYXBlPTE1KSksDQogICAgICAgICBzdGFyc2hhcGU9Z3VpZGVfbGVnZW5kKG5yb3c9MikpKw0KICB0aGVtZShsZWdlbmQucG9zaXRpb249ImJvdHRvbSIsbGVnZW5kLmJveCA9ICJob3Jpem9udGFsIikNCmBgYA0KDQpBcyBjYW4gYmUgc2VlbiBoZXJlLCB0aGlzIHN0aWxsIHJlc3VsdHMgaW4gKkR1bmtsZW9zdGV1cyB0ZXJyZWxsaSogcGxvdHRpbmcgYXMgYSBsYXJnZSBvdXRsaWVyIHRvIGFsbW9zdCBhbGwgb3RoZXIgZmlzaGVzLCBwYXJ0aWN1bGFybHkgZnVzaWZvcm0gc3BlY2llcy4NCg0KIyMgVHJlYXRpbmcgaGVhZCBsZW5ndGggaW4gYXJ0aHJvZGlyZXMgYXMgZXF1aXZhbGVudCB0byBwcmVicmFuY2hpYWwgbGVuZ3RoDQoNCmBgYHtyLGZpZy53aWR0aD05LGZpZy5hc3A9MC42NSxmaWcuY2FwPSJQbG90IG9mIHBlcmNlbnQgcHJlYnJhbmNoaWFsIGxlbmd0aCBhZ2FpbnN0IHRvdGFsIGxlbmd0aCBpbiBmaXNoZXMsIHRyZWF0aW5nIGhlYWQgbGVuZ3RoIGluIGFydGhyb2RpcmVzIGFzIGVxdWl2YWxlbnQgdG8gcHJlYnJhbmNoaWFsIGxlbmd0aCJ9DQpnZ3Bsb3QoZGF0YV9tb3V0aHMlPiUNCiAgICAgICAgIGRyb3BfbmEoc2hhcGUpJT4lDQogICAgICAgICBtdXRhdGUocHJlYnJhbmNoaWFsX2xlbmd0aD1pZmVsc2UoY2xhZGU9PSJQbGFjb2Rlcm1pIiwNCiAgICAgICAgICAgICAgICAgICAgICAgICAgICAgICAgICAgICAgICAgICBoZWFkX2xlbmd0aCxwcmVicmFuY2hpYWxfbGVuZ3RoKSklPiUNCiAgICAgICAgIGZpbHRlcighaXMubmEocHJlYnJhbmNoaWFsX2xlbmd0aCkpJT4lDQogICAgICAgICBmaWx0ZXIoY2xhZGUhPSJQbGFjb2Rlcm1pInwhaXMubmEodG90YWxfbGVuZ3RoKSklPiUNCiAgICAgICAgIG11dGF0ZShwZXJjZW50X3ByZWJyYW5jaGlhbF9sZW5ndGg9cHJlYnJhbmNoaWFsX2xlbmd0aC90b3RhbF9sZW5ndGgpLA0KICAgICAgIGFlcyh5PXBlcmNlbnRfcHJlYnJhbmNoaWFsX2xlbmd0aCx4PXRvdGFsX2xlbmd0aCkpKw0KICBnZW9tX3N0YXIoYWVzKGZpbGw9c2hhcGUsc3RhcnNoYXBlPWNsYWRlKSkrDQogIHNjYWxlX3N0YXJzaGFwZV9tYW51YWwodmFsdWVzPWMoMTUsMTMsMSwyOCkpKw0KICBzY2FsZV9maWxsX21hbnVhbCh2YWx1ZXM9YyhodWVfcGFsKCkoNSksImdyYXkiKSkrDQogIGdlb21fc3RhcihkYXRhPS4lPiVmaWx0ZXIoY2xhZGU9PSJQbGFjb2Rlcm1pIixnZW51cyE9IkR1bmtsZW9zdGV1cyIpJT4lDQogICAgICAgICAgICAgIG11dGF0ZShwZXJjZW50X3ByZWJyYW5jaGlhbF9sZW5ndGg9aGVhZF9sZW5ndGgvdG90YWxfbGVuZ3RoKSwNCiAgICAgICAgICAgIHN0YXJzaGFwZT0xLHNpemU9NS41LGNvbG9yPSJ3aGl0ZSIsZmlsbD0id2hpdGUiKSsNCiAgZ2VvbV9zdGFyKGRhdGE9LiU+JWZpbHRlcihjbGFkZT09IlBsYWNvZGVybWkiLGdlbnVzIT0iRHVua2xlb3N0ZXVzIiklPiUNCiAgICAgICAgICAgICAgbXV0YXRlKHBlcmNlbnRfcHJlYnJhbmNoaWFsX2xlbmd0aD1oZWFkX2xlbmd0aC90b3RhbF9sZW5ndGgpLA0KICAgICAgICAgICAgc3RhcnNoYXBlPTEsc2l6ZT0zLjUsZmlsbD0iYmxhY2siKSsNCiAgZ2VvbV9zdGFyKGRhdGE9bW91dGhfcHJlZGljdGlvbnMlPiUNCiAgICAgICAgICAgICAgZmlsdGVyKGdlbnVzPT0iRHVua2xlb3N0ZXVzIiklPiUNCiAgICAgICAgICAgICAgbXV0YXRlKHRvdGFsX2xlbmd0aD1maXQuVUpQLmZpdHRlZCwNCiAgICAgICAgICAgICAgICAgICAgIHBlcmNlbnRfcHJlYnJhbmNoaWFsX2xlbmd0aD1oZWFkX2xlbmd0aC90b3RhbF9sZW5ndGgpLA0KICAgICAgICAgICAgc3RhcnNoYXBlPTEsDQogICAgICAgICAgICBzaXplPTMsZmlsbD0ieWVsbG93IikrDQogIHNjYWxlX3hfY29udGludW91cyh0cmFucz0nbG9nMTAnKSsNCiAgc2NhbGVfeV9jb250aW51b3VzKGxhYmVscz1zY2FsZXM6OnBlcmNlbnRfZm9ybWF0KGFjY3VyYWN5PTEpKSsNCiAgYW5ub3RhdGUoZ2VvbT0idGV4dCIseD02ODAseT0wLjA4NSxoanVzdD0xLGxhYmVsPSJEdW5rbGVvc3RldXMiLGZvbnRmYWNlPTMpKw0KICBsYWJzKHg9IlRvdGFsIExlbmd0aCAoY20pIix5PSJQZXJjZW50IFByZWJyYW5jaGlhbCBMZW5ndGgiLGZpbGw9IkJvZHkgU2hhcGUiLA0KICAgICAgIHN0YXJzaGFwZT0iQ2xhZGUiKSsNCiAgdGhlbWVfY2xhc3NpYygpKw0KICBndWlkZXMoZmlsbD1ndWlkZV9sZWdlbmQobnJvdz0yLG92ZXJyaWRlLmFlcyA9IGxpc3Qoc3RhcnNoYXBlPTE1KSksDQogICAgICAgICBzdGFyc2hhcGU9Z3VpZGVfbGVnZW5kKG5yb3c9MikpKw0KICB0aGVtZShsZWdlbmQucG9zaXRpb249ImJvdHRvbSIsbGVnZW5kLmJveCA9ICJob3Jpem9udGFsIikNCmBgYA0KDQpFdmVuIGlmIHRyZWF0aW5nIGhlYWQgbGVuZ3RoIGluIGFydGhyb2RpcmVzIGFzIGVxdWl2YWxlbnQgdG8gcHJlLWJyYW5jaGlhbCBsZW5ndGggaW4gb3RoZXIgZmlzaGVzLCB0aGlzIHN0aWxsIHJlc3VsdHMgaW4gKkR1bmtsZW9zdGV1cyogaGF2aW5nIGEgYmVsb3ctYXZlcmFnZSBwcmVicmFuY2hpYWwgbGVuZ3RoIGZvciBpdHMgc2l6ZSAocGxvdHRpbmcgYW1vbmcgZWxvbmdhdGUsIGFuZ3VpbGxpZm9ybSwgYW5kIG1hY3J1cmlmb3JtIHRheGEpLCBhbmQgZG9lcyBub3QgcmVzZW1ibGUgdGhlIHBhdHRlcm5zIHNlZW4gaW4gb3RoZXIgYXJ0aHJvZGlyZXMsIHdoaWNoIGhhdmUgInByZWJyYW5jaGlhbCBsZW5ndGhzIiB0aGF0IHBsb3QgbXVjaCBjbG9zZXIgdG8gZnVzaWZvcm0gZmlzaGVzLg0KDQojIyBIaWdobGlnaHRpbmcgZ3JvdXBlcnMNCg0KKHJlZjpTZXJyYW5pZGFlKSBQbG90IG9mIHBlcmNlbnQgaGVhZCBsZW5ndGggYWdhaW5zdCBsb2ctdHJhbnNmb3JtZWQgdG90YWwgbGVuZ3RoLCBzaG93aW5nIGhvdyBncm91cGVycyAoU2VycmFuaWRhZSkgaGF2ZSBtdWNoIGxhcmdlciBoZWFkcyB0aGFuIG90aGVyIGZpc2hlcy4gVGhlIG9uZSBwb2ludCBiZWxvdyB0aGUgdGhyZXNoaG9sZCBmb3IgZWxvbmdhdGUgYm9kaWVzIGlzICpQc2V1ZGFudGhpYXMgZmFzY2lhdHVzKiwgd2hpY2ggaGFzIGEgdmVyeSBsb25nIGNhdWRhbCBmaW4uDQoNCmBgYHtyLGZpZy5jYXA9IihyZWY6U2VycmFuaWRhZSkifQ0KZ2dwbG90KA0KICBkYXRhX21vdXRocyAlPiUNCiAgICBkcm9wX25hKGhlYWRfbGVuZ3RoLHRvdGFsX2xlbmd0aCklPiUNCiAgICBtdXRhdGUocGVyY2VudF9oZWFkX2xlbmd0aD1oZWFkX2xlbmd0aC90b3RhbF9sZW5ndGgpLA0KICBhZXModG90YWxfbGVuZ3RoLGhlYWRfbGVuZ3RoL3RvdGFsX2xlbmd0aCkpKw0KICBnZW9tX3N0YXIoYWVzKHN0YXJzaGFwZT1jbGFkZSksZmlsbD0ibGlnaHQgZ3JheSIsY29sb3I9ImRhcmsgZ3JleSIpKw0KICBnZW9tX3N0YXIoZGF0YT0uJT4lZmlsdGVyKGZhbWlseSAlaW4lIGMoIlNlcnJhbmlkYWUiKSksDQogICAgICAgICAgICBhZXMoc3RhcnNoYXBlPWNsYWRlLGZpbGw9ZmFtaWx5KSkrDQogIHNjYWxlX3N0YXJzaGFwZV9tYW51YWwodmFsdWVzPWMoMTUsMTMsMSwyOCwxMSksZ3VpZGU9Im5vbmUiKSsNCiAgZ2VvbV9obGluZSh5aW50ZXJjZXB0PTAuMTcsbGluZXR5cGU9ImRhc2hlZCIpKw0KICBhbm5vdGF0ZShnZW9tPSJ0ZXh0Iix4PTIseT0wLjE2LGxhYmVsPSJFZWwtTGlrZSBUYXhhIikrDQogIHNjYWxlX3hfY29udGludW91cyh0cmFucz0ibG9nMTAiKSsNCiAgc2NhbGVfeV9jb250aW51b3VzKGxhYmVscz1zY2FsZXM6OnBlcmNlbnRfZm9ybWF0KGFjY3VyYWN5PTEpKSsNCiAgbGFicyh4PSJUb3RhbCBMZW5ndGggKGNtKSIseT0iUGVyY2VudCBIZWFkIExlbmd0aCIsZmlsbD0iQ2xhZGUiKSsNCiAgZ3VpZGVzKGZpbGw9Z3VpZGVfbGVnZW5kKG5yb3c9MixvdmVycmlkZS5hZXMgPSBsaXN0KHN0YXJzaGFwZT0xNSkpKSsNCiAgdGhlbWVfY2xhc3NpYygpKw0KICB0aGVtZShsZWdlbmQucG9zaXRpb249YygwLjE1LDAuMSkpDQpgYGANCg0KIyMgVXNpbmcgbGVuZ3RocyBvZiBFbmdlbG1hbiAoMjAyMykNCg0KVGhpcyBwYXBlciB3YXMgb3JpZ2luYWxseSBpbnRlbmRlZCB0byBjb21lIG91dCBiZWZvcmUgRW5nZWxtYW4gKDIwMjMpLCBpbiBvcmRlciB0byBsYXkgdGhlIGdyb3VuZHdvcmsgZm9yIHRoYXQgc3R1ZHkuIEhvd2V2ZXIsIGR1ZSB0byB0aGUgdmFnYXJpZXMgb2YgcHVibGljYXRpb24sIEVuZ2VsbWFuICgyMDIzKSBhY3R1YWxseSBlbmRlZCB1cCBjb21pbmcgb3V0IGluIHByaW50IGZpcnN0LiBUaGlzIHN0dWR5IGlzIHN0aWxsIHdyaXR0ZW4gdHJlYXRpbmcgdGhlIHdvcmsgb2YgRW5nZWxtYW4gKDIwMjMpIGFzIGlmIGl0IG5ldmVyIGV4aXN0ZWQsIGJ1dCBiZWNhdXNlIHRoYXQgc3R1ZHkgaXMgYWxyZWFkeSBvdXQgaXQgaXMgcG9zc2libGUgdG8gbWFrZSBhIHVzZWZ1bCBjcm9zcy1zdHVkeSBjb21wYXJpc29uIHNob3dpbmcgaG93IHRoZSBsZW5ndGggZXN0aW1hdGVzIGluIEVuZ2VsbWFuICgyMDIzKSByZXN1bHQgaW4gKkR1bmtsZW9zdGV1cyogcG9zaXRpb24gd2l0aGluIHRoZSBoZWFkLXRydW5rIHByb3BvcnRpb25zIG9mIGZpc2hlcyBhcyBhIHBvc3RzY3JpcHQuDQoNCihyZWY6ZW5nZWxtYW5oZWFkKSBIZWFkLXRydW5rIHByb3BvcnRpb25zIG9mICpEdW5rbGVvc3RldXMqIGluIEVuZ2VsbWFuICgyMDIzKSBjb21wYXJlZCB0byB0aG9zZSB1c2luZyBlc3RpbWF0ZXMgZnJvbSBGZXJyw7NuIGV0IGFsLiAoMjAxNykNCg0KYGBge3IsZmlnLmNhcD0iKHJlZjplbmdlbG1hbmhlYWQpIn0NCndyYXBwZXIgPC0gZnVuY3Rpb24oeCwgLi4uKSBwYXN0ZShzdHJ3cmFwKHgsIC4uLiksIGNvbGxhcHNlID0gIlxuIikNCihlbmdlbG1hbl9oZWFkX2xlbmd0aDwtZ2dwbG90KA0KICAgIGRhdGFfbW91dGhzICU+JQ0KICAgICAgZHJvcF9uYShoZWFkX2xlbmd0aCx0b3RhbF9sZW5ndGgpJT4lDQogICAgICBtdXRhdGUocGVyY2VudF9oZWFkX2xlbmd0aD1oZWFkX2xlbmd0aC90b3RhbF9sZW5ndGgsDQogICAgICAgICAgICAgZWxvbmdhdGU9aWZlbHNlKHNoYXBlICVpbiUgYygiYW5ndWlsbGlmb3JtIiwibWFjcnVyaWZvcm0iKXwNCiAgICAgICAgICAgICAgICAgICAgICAgICAgICAgICBnZW51cyAlaW4lIGMoIkNoZWlyb2NlbnRydXMiLCJDb3J5cGhhZW5hIiwiVGV0cmFwdHVydXMiLA0KICAgICAgICAgICAgICAgICAgICAgICAgICAgICAgICAgICAgICAgICAgICAiUG9seXB0ZXJ1cyIsIkFsZXBpc2F1cnVzIiwiR29ub3J5bmNodXMiLA0KICAgICAgICAgICAgICAgICAgICAgICAgICAgICAgICAgICAgICAgICAgICAiUmhhcGhpb2RvbiIsDQogICAgICAgICAgICAgICAgICAgICAgICAgICAgICAgICAgICAgICAgICAgICJUaHlyc2l0b2lkZXMiLCJHZW1weWx1cyIsIlRoeXJzaXRlcyIpfA0KICAgICAgICAgICAgICAgICAgICAgICAgICAgICAgIGZhbWlseSAlaW4lICJCZWxvbmlkYWUiLA0KICAgICAgICAgICAgICAgICAgICAgICAgICAgICAiRWxvbmdhdGUgUG9zdGNyYW5pdW0iLCJOb3QgRWxvbmdhdGUiKSklPiUNCiAgICAgIGFycmFuZ2UoZWxvbmdhdGUpLA0KICAgIGFlcyh0b3RhbF9sZW5ndGgsaGVhZF9sZW5ndGgvdG90YWxfbGVuZ3RoKSkrDQogICAgZ2VvbV9zdGFyKGFlcyhzdGFyc2hhcGU9Y2xhZGUsZmlsbD1lbG9uZ2F0ZSksZGF0YT0uJT4lZmlsdGVyKGdlbnVzIT0iRHVua2xlb3N0ZXVzIikpKw0KICAgIGdlb21fc3RhcihkYXRhPS4lPiVmaWx0ZXIoZWxvbmdhdGU9PSJFbG9uZ2F0ZSBQb3N0Y3Jhbml1bSIsZ2VudXMhPSJEdW5rbGVvc3RldXMiKSwNCiAgICAgICAgICAgICAgYWVzKHN0YXJzaGFwZT1jbGFkZSxmaWxsPWVsb25nYXRlKSkrDQogICAgc2NhbGVfc3RhcnNoYXBlX21hbnVhbCh2YWx1ZXM9YygxNSwxMywxLDI4LDExKSkrDQogICAgZ2VvbV9zdGFyKGRhdGE9LiU+JWZpbHRlcihjbGFkZT09IlBsYWNvZGVybWkiLGdlbnVzIT0iRHVua2xlb3N0ZXVzIiksDQogICAgICAgICAgICAgIHN0YXJzaGFwZT0xLHNpemU9NCwNCiAgICAgICAgICAgICAgY29sb3I9IndoaXRlIixmaWxsPSJibGFjayIpKw0KICAgIGdlb21faGxpbmUoeWludGVyY2VwdD0wLjE3LGxpbmV0eXBlPSJsb25nZGFzaCIpKw0KICAgICAgZ2VvbV9zZWdtZW50KGRhdGE9LiU+JWZpbHRlcihzcGVjaW1lbj09IkNNTkggNTc2OCIpICU+JSBtdXRhdGUodG90YWxfbGVuZ3RoPTM0MC43KSwNCiAgICAgICAgICAgICAgIGFlcyh4ZW5kPTExMDAseWVuZD1oZWFkX2xlbmd0aC90b3RhbF9sZW5ndGgpLGNvbG9yPSJibGFjayIpKw0KICAgIGdlb21fc2VnbWVudChkYXRhPS4lPiVmaWx0ZXIoc3BlY2ltZW49PSJDTU5IIDU3NjgiKSwNCiAgICAgICAgICAgICAgICAgYWVzKHhlbmQ9MTEwMCx5ZW5kPWhlYWRfbGVuZ3RoL3RvdGFsX2xlbmd0aCksY29sb3I9ImJsYWNrIikrDQogIGdlb21fc3RhcihkYXRhPS4gJT4lIGZpbHRlcihnZW51cz09IkR1bmtsZW9zdGV1cyIpICU+JQ0KICAgICAgICAgICAgICBtdXRhdGUodG90YWxfbGVuZ3RoPWNhc2Vfd2hlbihzcGVjaW1lbj09IkNNTkggNTc2OCJ+MzQwLjcsDQogICAgICAgICAgICAgICAgICAgICAgICAgICAgICAgICAgICAgICAgICAgIHNwZWNpbWVuPT0iQ01OSCA3NDI0In4xODguOSwNCiAgICAgICAgICAgICAgICAgICAgICAgICAgICAgICAgICAgICAgICAgICAgc3BlY2ltZW49PSJDTU5IIDYwOTAifjI4My4zLA0KICAgICAgICAgICAgICAgICAgICAgICAgICAgICAgICAgICAgICAgICAgICBzcGVjaW1lbj09IkNNTkggNzA1NCJ+Mjk1LjUpKSAlPiUNCiAgICAgICAgICAgICAgbXV0YXRlKHBlcmNlbnRfaGVhZF9sZW5ndGg9aGVhZF9sZW5ndGgvdG90YWxfbGVuZ3RoKSwNCiAgICAgICAgICAgIHN0YXJzaGFwZT0xLHNpemU9NSxmaWxsPSJ3aGl0ZSIsY29sb3I9IndoaXRlIikrDQogIGdlb21fc2VnbWVudCgNCiAgICBkYXRhPWRhdGFfbW91dGhzICU+JSBmaWx0ZXIoZ2VudXM9PSJEdW5rbGVvc3RldXMiKSAlPiUgZHJvcF9uYShVSlApICU+JQ0KICAgICAgbXV0YXRlKG5ld19sZW5ndGg9Y2FzZV93aGVuKHNwZWNpbWVuPT0iQ01OSCA1NzY4In4zNDAuNywNCiAgICAgICAgICAgICAgICAgICAgICAgICAgICAgICAgICBzcGVjaW1lbj09IkNNTkggNzQyNCJ+MTg4LjksDQogICAgICAgICAgICAgICAgICAgICAgICAgICAgICAgICAgc3BlY2ltZW49PSJDTU5IIDYwOTAifjI4My4zLA0KICAgICAgICAgICAgICAgICAgICAgICAgICAgICAgICAgIHNwZWNpbWVuPT0iQ01OSCA3MDU0In4yOTUuNSkpICU+JQ0KICAgICAgbXV0YXRlKHBlcmNlbnRfaGVhZF9sZW5ndGgxPWhlYWRfbGVuZ3RoL3RvdGFsX2xlbmd0aCwNCiAgICAgICAgICAgICBwZXJjZW50X2hlYWRfbGVuZ3RoMj1oZWFkX2xlbmd0aC9uZXdfbGVuZ3RoKSwNCiAgICBhZXMoeT1wZXJjZW50X2hlYWRfbGVuZ3RoMSx4PXRvdGFsX2xlbmd0aCwNCiAgICAgICAgeWVuZD1wZXJjZW50X2hlYWRfbGVuZ3RoMix4ZW5kPW5ld19sZW5ndGgpLA0KICAgIGxpbmV0eXBlPSJkYXNoZWQiKSsNCiAgICBnZW9tX3N0YXIoZGF0YT0uICU+JSBmaWx0ZXIoZ2VudXM9PSJEdW5rbGVvc3RldXMiKSwNCiAgICAgICAgICAgIHN0YXJzaGFwZT0xLHNpemU9NCxmaWxsPSJ3aGl0ZSIsY29sb3I9ImJsYWNrIikrDQogICAgZ2VvbV9zdGFyKGRhdGE9LiAlPiUgZmlsdGVyKGdlbnVzPT0iRHVua2xlb3N0ZXVzIiksDQogICAgICAgICAgICAgIHN0YXJzaGFwZT0xLHNpemU9NCxhbHBoYT0wLjUsZmlsbD0ieWVsbG93Iixjb2xvcj0iZ3JleSIpKw0KICAgIGdlb21fc3RhcihkYXRhPS4gJT4lIGZpbHRlcihnZW51cz09IkR1bmtsZW9zdGV1cyIpICU+JQ0KICAgICAgICAgICAgICAgIG11dGF0ZSh0b3RhbF9sZW5ndGg9Y2FzZV93aGVuKHNwZWNpbWVuPT0iQ01OSCA1NzY4In4zNDAuNywNCiAgICAgICAgICAgICAgICAgICAgICAgICAgICAgICAgICAgICAgICAgICAgICBzcGVjaW1lbj09IkNNTkggNzQyNCJ+MTg4LjksDQogICAgICAgICAgICAgICAgICAgICAgICAgICAgICAgICAgICAgICAgICAgICAgc3BlY2ltZW49PSJDTU5IIDYwOTAifjI4My4zLA0KICAgICAgICAgICAgICAgICAgICAgICAgICAgICAgICAgICAgICAgICAgICAgIHNwZWNpbWVuPT0iQ01OSCA3MDU0In4yOTUuNSkpICU+JQ0KICAgICAgICAgICAgICAgIG11dGF0ZShwZXJjZW50X2hlYWRfbGVuZ3RoPWhlYWRfbGVuZ3RoL3RvdGFsX2xlbmd0aCksDQogICAgICAgICAgICBzdGFyc2hhcGU9MSxzaXplPTQsZmlsbD0ieWVsbG93Iixjb2xvcj0iYmxhY2siKSsNCiAgICBhbm5vdGF0ZShnZW9tPSJ0ZXh0Iix4PTIseT0wLjE2LHNpemU9MyxsYWJlbD0iRWVsLUxpa2UgVGF4YSIpKw0KICAgIGFubm90YXRlKGdlb209InRleHQiLHg9MTE1MCx5PTAuMTgxLGhqdXN0PTAsbGFiZWw9IkVuZ2VsbWFuICgyMDIzKSIsDQogICAgICAgICAgIHNpemU9MykrDQogICAgYW5ub3RhdGUoZ2VvbT0idGV4dCIseD0xMTUwLHk9MC4wOSxoanVzdD0wLA0KICAgICAgICAgICAgIGxhYmVsPXdyYXBwZXIoIkZlcnLDs24gZXQgYWwuICgyMDE3KSIsd2lkdGggPSAxNCksc2l6ZT0zKSsNCiAgICBjb29yZF9jYXJ0ZXNpYW4oeGxpbT1jKDEsMjkwMCkseWxpbT1jKDAuMDQsMC4zOSkpKw0KICAgIHRoZW1lX2NsYXNzaWMoKSsNCiAgICBzY2FsZV94X2NvbnRpbnVvdXModHJhbnM9ImxvZzEwIikrDQogICAgc2NhbGVfeV9jb250aW51b3VzKGxhYmVscz1zY2FsZXM6OnBlcmNlbnRfZm9ybWF0KGFjY3VyYWN5PTEpKSsNCiAgICBsYWJzKHg9IlRvdGFsIExlbmd0aCAoY20pIix5PSJQZXJjZW50IEhlYWQgTGVuZ3RoIixmaWxsPSJCb2R5IFNoYXBlIixzdGFyc2hhcGU9IkNsYWRlIikrDQogICAgZ3VpZGVzKGZpbGw9Z3VpZGVfbGVnZW5kKG5yb3c9MixvdmVycmlkZS5hZXMgPSBsaXN0KHN0YXJzaGFwZT0xNSkpLA0KICAgICAgICAgICBzdGFyc2hhcGU9Z3VpZGVfbGVnZW5kKG5jb2w9MixvdmVycmlkZS5hZXMgPSBsaXN0KGZpbGw9ImxpZ2h0IGdyYXkiKSkpKw0KICAgIHRoZW1lKGxlZ2VuZC5wb3NpdGlvbj0iYm90dG9tIiwNCiAgICAgICAgICBsZWdlbmQudGV4dD1lbGVtZW50X3RleHQoc2l6ZT05KSwNCiAgICAgICAgICBsZWdlbmQudGl0bGU9ZWxlbWVudF90ZXh0KHNpemU9MTApKSkNCg0KZ2dzYXZlKCJBcnRocm9kaXJlIE1vdXRocyBFbmdlbG1hbiBIZWFkIExlbmd0aHMucG5nIixlbmdlbG1hbl9oZWFkX2xlbmd0aCwNCiAgICAgICB3aWR0aD0xNzgsaGVpZ2h0PTEzNCx1bml0cz0ibW0iLGRldmljZT0icG5nIikNCmBgYA0KDQpBcyBjYW4gYmUgc2VlbiwgdGhlIG5ldyBsZW5ndGggZXN0aW1hdGVzIHJlc3VsdCBpbiBoZWFkLXRydW5rIHByb3BvcnRpb25zIGZvciAqRHVua2xlb3N0ZXVzKiB3aXRoaW4gdGhlIHJhbmdlIG9mIHR5cGljYWwgZnVzaWZvcm0gb3N0ZWljaHRoeWFucyBhbmQgY2hvbmRyaWNodGh5YW5zIGFzIHdlbGwgYXMgYWdyZWVpbmcgbW9yZSBjbG9zZWx5IHdpdGggdGhvc2Ugb2YgY29tcGxldGUgYXJ0aHJvZGlyZXMsIGFsYmVpdCBsb3dlciB0aGFuIGV4cGVjdGVkIGJhc2VkIG9uIGNvY2Nvc3Rlb21vcnBocy4gKkFtYXppY2h0aHlzKiBzdGlsbCBwbG90cyBiZWxvdyB0aGUgdGhyZXNoaG9sZCBmb3IgdGF4YSB3aXRoIGVsb25nYXRlIHRydW5rcywgYnV0IHRoZW4gYWdhaW4gc3BlY2ltZW5zIG9mICpBbWF6aWNodGh5cyogc2hvdyBhIGJvZHkgcGxhbiB3aXRoIGFuIGVsb25nYXRlIHRydW5rLiBUaGUgZmFjdCB0aGF0ICpEdW5rbGVvc3RldXMqIGlzIGNsb3NlIHRvIGJ1dCBwZXJmZWN0bHkgYWxpZ25zIHdpdGggbmVpdGhlciBjb2Njb3N0ZW9tb3JwaHMgbm9yICpBbWF6aWNodGh5cyogaXMgdG8gYmUgZXhwZWN0ZWQgZ2l2ZW4gaXQgaXMgaW4gYSBzZXBhcmF0ZSBjbGFkZSBmcm9tIHRoZXNlIGdyb3VwcyAoRHVua2xlb3N0ZW9pZGVhKSBhbmQgaW4gc2V2ZXJhbCByZXNwZWN0cyBpdHMgY3JhbmlhbCBtb3JwaG9sb2d5IGlzIGRlY2lkZWRseSBnZW5lcmFsaXplZCAoZS5nLiwgaXQgbGFja3MgdGhlIG92ZXJoYW5naW5nIG5ldXJvY3Jhbml1bSBvZiBjb2Njb3N0ZW9tb3JwaHMpLg0KDQpIb3dldmVyLCB0aGUgbmV3IGxlbmd0aCBlc3RpbWF0ZXMganVzdCAqYmFyZWx5KiBwdXQgRHVua2xlb3N0ZXVzIHRlcnJlbGxpIHdpdGhpbiB0aGUgcmFuZ2Ugb2YgaGVhZC1ib2R5IHByb3BvcnRpb25zIGV4cGVjdGVkIGZvciBmaXNoZXMgd2l0aG91dCBncmVhdGx5IGVsb25nYXRlZCB0cnVua3MuIEluIG90aGVyIHdvcmRzLCBkZXNwaXRlIHRoZSBleHRyZW1lbHkgc2hvcnQsIGNodW5reSBib2R5IGRlcGljdGVkIGluIEVuZ2VsbWFuICgyMDIzKSwgdGhhdCBib2R5IHNoYXBlIGlzIGFsbW9zdCB0aGUgKmxvbmdlc3QqICpEdW5rbGVvc3RldXMqIGNhbiBiZSAgd2l0aG91dCB2aW9sYXRpbmcgYSB0eXBpY2FsIGZpc2ggYm9keSBwbGFuLg0KDQpBZ2Fpbiwgc29tZSBmaXNoZXMgbGlrZSAqQ29yeXBoYWVuYSogaGF2ZSB2ZXJ5IGVsb25nYXRlIHRydW5rcyBhbmQgYSBzb21ld2hhdCBmdXNpZm9ybSBzaGFwZSwgYnV0ICpEdW5rbGVvc3RldXMq4oCZIHByZXNlcnZlZCBhbmF0b215IGRvZXMgbm90IHNob3cgYW55IGZlYXR1cmVzIGluZGljYXRpdmUgb2YgYSAqQ29yeXBoYWVuYSotbGlrZSBib2R5IHBsYW4uICpEdW5rbGVvc3RldXMqIGxhY2tzIHRoZSBlbG9uZ2F0ZSB2ZW50cmFsIGFybW9yIG9mICpBbWF6aWNodGh5cyosIGFuZCBhbG1vc3QgZXZlcnkgYm9keSBtZWFzdXJlbWVudCBhc3NvY2lhdGVkIHdpdGggdGhpcyBzcGVjaWVzIHByb2R1Y2VzIGxlbmd0aHMgdGhhdCBhZ3JlZSB3aXRoIHRoZSBzaG9ydGVyIGJvZHkgcGxhbiAoRW5nZWxtYW4sIDIwMjMpLiBUaHVzLCBhIG11Y2ggbG9uZ2VyIGJvZHkgc2hhcGUgd291bGQgYmUgY29tcGxldGVseSBzcGVjdWxhdGl2ZSwgYW5kIGFjdHVhbGx5IGhhdmUgdG8gZGlzYWdyZWUgd2l0aCBtYW55IHByb3BvcnRpb25zIHN0cm9uZ2x5IGNvbnNlcnZlZCBhY3Jvc3MgZmlzaGVzLg0KDQojIE1vdXRoIGxlbmd0aA0KDQoocmVmOm1vdXRobGVuZ3RoKSBQbG90IG9mIHBlcmNlbnQgbW91dGggbGVuZ3RoIGFnYWluc3QgaGVhZCBsZW5ndGggaW4gZmlzaGVzLCBzaG93aW5nIGhvdyAqRHVua2xlb3N0ZXVzIHRlcnJlbGxpKiAoeWVsbG93IHN0YXJzKSwgZXNwZWNpYWxseSBhZHVsdCBpbmRpdmlkdWFscyBvZiAqRC4gdGVycmVsbGkqLCBoYXZlIG11Y2ggbGFyZ2VyIG1vdXRocyB0aGFuIG90aGVyIGFydGhyb2RpcmVzDQoNCmBgYHtyLGZpZy5jYXA9IihyZWY6bW91dGhsZW5ndGgpIn0NCihtb3V0aF9sZW5ndGhfcGxvdDwtZGF0YV9tb3V0aHMlPiUNCiAgZHJvcF9uYShjbGFkZSklPiUNCiAgbXV0YXRlKHBlcmNlbnRfbW91dGhfbGVuZ3RoPW1vdXRoX2xlbmd0aC9oZWFkX2xlbmd0aCklPiUNCiAgbXV0YXRlKGNsYWRlPWlmZWxzZShnZW51cz09IkR1bmtsZW9zdGV1cyIsIkR1bmtsZW9zdGV1cyIsY2xhZGUpLA0KICAgICAgICAgY2xhZGU9aWZlbHNlKGNsYWRlPT0iUGxhY29kZXJtaSImZ2VudXMhPSJEdW5rbGVvc3RldXMiLCJPdGhlciBQbGFjb2Rlcm1pIixjbGFkZSkpJT4lDQogIGFycmFuZ2UocmV2KGNsYWRlKSklPiUNCiAgZ2dwbG90KGFlcyhoZWFkX2xlbmd0aCxwZXJjZW50X21vdXRoX2xlbmd0aCkpKw0KICBnZW9tX3N0YXIoYWVzKHN0YXJzaGFwZT1jbGFkZSxmaWxsPWNsYWRlKSkrDQogIHNjYWxlX3N0YXJzaGFwZV9tYW51YWwodmFsdWVzPWMoMTUsMTMsMSwxLDI4KSwNCiAgICAgICAgICAgICAgICAgICAgICAgICBsYWJlbHM9YygiQWN0aW5vcHRlcnlnaWkiLCJDaG9uZHJpY2h0aHllcyIsIipEdW5rbGVvc3RldXMqIiwNCiAgICAgICAgICAgICAgICAgICAgIk90aGVyIFBsYWNvZGVybWkiLCJTYXJjb3B0ZXJ5Z2lpIiksDQogICAgICAgICAgICAgICAgICAgIGJyZWFrcz1jKCJBY3Rpbm9wdGVyeWdpaSIsIkNob25kcmljaHRoeWVzIiwiRHVua2xlb3N0ZXVzIiwNCiAgICAgICAgICAgICAgICAgICAgICAgICAgICAgIk90aGVyIFBsYWNvZGVybWkiLCJTYXJjb3B0ZXJ5Z2lpIikpKw0KICBzY2FsZV9maWxsX21hbnVhbCh2YWx1ZXM9YyhodWVfcGFsKCkoMylbMV0saHVlX3BhbCgpKDMpWzNdLA0KICAgICAgICAgICAgICAgICAgICAgICAgICAgICAieWVsbG93IiwiYmxhY2siLGh1ZV9wYWwoKSgzKVsyXSksDQogICAgICAgICAgICAgICAgICAgIGxhYmVscz1jKCJBY3Rpbm9wdGVyeWdpaSIsIkNob25kcmljaHRoeWVzIiwiKkR1bmtsZW9zdGV1cyoiLA0KICAgICAgICAgICAgICAgICAgICAiT3RoZXIgUGxhY29kZXJtaSIsIlNhcmNvcHRlcnlnaWkiKSwNCiAgICAgICAgICAgICAgICAgICAgYnJlYWtzPWMoIkFjdGlub3B0ZXJ5Z2lpIiwiQ2hvbmRyaWNodGh5ZXMiLCJEdW5rbGVvc3RldXMiLA0KICAgICAgICAgICAgICAgICAgICAgICAgICAgICAiT3RoZXIgUGxhY29kZXJtaSIsIlNhcmNvcHRlcnlnaWkiKSkrDQogIGdlb21fc3RhcihkYXRhPS4lPiVmaWx0ZXIoY2xhZGU9PSJPdGhlciBQbGFjb2Rlcm1pIixnZW51cyE9IkR1bmtsZW9zdGV1cyIpLHNpemU9NCwNCiAgICAgICAgICAgIHN0YXJzaGFwZT0xLGNvbG9yPSJ3aGl0ZSIsZmlsbD0iYmxhY2siLHN0YXJzdHJva2U9MC41KSsNCiAgZ2VvbV9zdGFyKGRhdGE9LiU+JWZpbHRlcihnZW51cz09IkR1bmtsZW9zdGV1cyIpLHN0YXJzaGFwZT0xLHNpemU9My41LA0KICAgICAgICAgICAgY29sb3I9ImJsYWNrIixmaWxsPSJ5ZWxsb3ciKSsNCiAgc2NhbGVfeF9jb250aW51b3VzKHRyYW5zPSJsb2cxMCIpKw0KICBzY2FsZV95X2NvbnRpbnVvdXMobGFiZWxzPXNjYWxlczo6cGVyY2VudF9mb3JtYXQoYWNjdXJhY3k9MSkpKw0KICBsYWJzKHN0YXJzaGFwZT0iQ2xhZGUiLGZpbGw9IkNsYWRlIix4PSJIZWFkIExlbmd0aCAoY20pIiwNCiAgICAgICB5PSJNb3V0aCBMZW5ndGggKGFzIHBlcmNlbnQgb2YgSGVhZCBMZW5ndGgpIikrDQogIHRoZW1lX2NsYXNzaWMoKSsNCiAgdGhlbWUobGVnZW5kLnBvc2l0aW9uPWMoMC4xNSwwLjg1KSwNCiAgICAgICAgbGVnZW5kLnRleHQ9ZWxlbWVudF9tYXJrZG93bigpKSkNCmdnc2F2ZSgiQXJ0aHJvZGlyZSBNb3V0aHMgRmlndXJlIDkgKE1vdXRoIExlbmd0aCkucGRmIixtb3V0aF9sZW5ndGhfcGxvdCwNCiAgICAgICB3aWR0aD0xNjUsaGVpZ2h0PTEyNSx1bml0cz0ibW0iLGRldmljZT0icGRmIikNCmBgYA0KDQpCYXNlZCBvbiB0aGlzLCBpdCBpcyBhcHBhcmVudCB0aGF0ICpEdW5rbGVvc3RldXMqIGhhcyBhIG11Y2ggbGFyZ2VyIG1vdXRoIChyZWxhdGl2ZSB0byBoZWFkIHNpemUpIHRoYW4gbW9zdCBvdGhlciBhcnRocm9kaXJlcywgZXZlbiBjb21wYXJlZCB0byB0aGUgY2xvc2VseSByZWxhdGVkIGR1bmtsZW9zdGVvaWQgKkVhc3RtYW5vc3RldXMgY2FsbGlhc3BpcyouIFRoZSBvbmx5IGFydGhyb2RpcmVzIHdoaWNoIGhhcyBtb3V0aCBwcm9wb3J0aW9ucyBzaW1pbGFyIHRvICpEdW5rbGVvc3RldXMqIGFyZSB0aGUgRnJhc25pYW4gKkhhZHJvc3RldXMgcmFwYXgqIGFuZCB0aGUgY29ldmFsIENsZXZlbGFuZCBTaGFsZSBhcnRocm9kaXJlcyAqSGVpbnR6aWNodGh5cyBnb3VsZGlpKiBhbmQgKkJ1bmdhcnRpdXMgcGVyaXNzdXMqLiBFdmVuIHRoZXNlIHRheGEgb25seSBoYXZlIG1vdXRocyBzaW1pbGFyIGluIHNpemUgdG8ganV2ZW5pbGUgKkQuIHRlcnJlbGxpKiwgdGhlIGFkdWx0cyAod2hpY2ggaGF2ZSBwcm9wb3J0aW9uYWxseSBsYXJnZXIgbW91dGhzKSBoYXZlIG1vdXRocyBtdWNoIGxhcmdlciB0aGFuIGFueSBvdGhlciBhcnRocm9kaXJlLg0KDQpUaGlzIHJlc3VsdCBoaWdobGlnaHRzIHR3byBpbXBvcnRhbnQgcG9pbnRzOg0KMS4gKkR1bmtsZW9zdGV1cyogaGFzIGEgcHJvcG9ydGlvbmFsbHkgbGFyZ2UgbW91dGggZXZlbiBhbW9uZyBhcnRocm9kaXJlcyAod2hpY2ggc2hvdyBtdWNoIGxhcmdlciBtb3V0aHMgcmVsYXRpdmUgdG8gbGVuZ3RoIHRoYW4gc2hhcmtzKSwgd2hpY2ggbWVhbnMgdGhhdCBlc3RpbWF0ZXMgYmFzZWQgb24gbW91dGggZGltZW5zaW9ucyBhcmUgZXZlbiBtb3JlIGxpa2VseSB0byBiZSBvdmVyZXN0aW1hdGVzLiANCjIuICpEdW5rbGVvc3RldXMqIGhhcyBhbiBleHRyZW1lbHkgbGFyZ2UgbW91dGggcmVsYXRpdmUgdG8gb3RoZXIgYXJ0aHJvZGlyZXMgYW5kIHRoaXMgaXMgaGlnaGx5IGxpa2VseSB0byBoYXZlIGFmZmVjdGVkIGl0cyBwYWxlb2Jpb2xvZ3kgYW5kIHBhbGVvZWNvbG9neSAoaS5lLiwgdGFraW5nIGxhcmdlciBwcmV5IHRoYW4gb3RoZXIgYXJ0aHJvZGlyZXMpLg0KDQojIFJlZmVyZW5jZXMNCg0KPGRpdiBzdHlsZT0idGV4dC1pbmRlbnQ6IC00MHB4OyBwYWRkaW5nLWxlZnQ6IDQwcHg7IG1hcmdpbi1ib3R0b209MDsgbWFyZ2luLXRvcD0wIj4NCkJyYW5zdGV0dGVyIFMsIGFuZCBTdGlsZXMgUi4gMTk4Ny4gQWdlIGFuZCBncm93dGggZXN0aW1hdGVzIG9mIHRoZSBidWxsIHNoYXJrLCAqQ2FyY2hhcmhpbnVzIGxldWNhcyosIGZyb20gdGhlIG5vcnRoZXJuIEd1bGYgb2YgTWV4aWNvLiAqRW52aXJvbm1lbnRhbCBCaW9sb2d5IG9mIEZpc2hlcyogMjA6MTY54oCTMTgxLg0KDQpDbGlmZiBHLiAxOTk1LiBTaGFya3MgY2F1Z2h0IGluIHRoZSBwcm90ZWN0aXZlIGdpbGwgbmV0cyBvZmYgS3dhWnVsdS1OYXRhbCwgU291dGggQWZyaWNhLiA4LiBUaGUgR3JlYXQgaGFtbWVyaGVhZCBzaGFyayAqU3BoeXJuYSBtb2thcnJhbiogKFLDvHBwZWxsKS4gKlNvdXRoIEFmcmljYW4gSm91cm5hbCBvZiBNYXJpbmUgU2NpZW5jZSogMTU6MTA1LTExNC4gRE9JIDEwLjI5ODkvMDI1Nzc2MTk1Nzg0MTU2MzMxLg0KDQpEZW5uaXMgSywgYW5kIE1pbGVzIFJTLiAxOTgxLiBBIHBhY2h5b3N0ZW9tb3JwaCBhcnRocm9kaXJlIGZyb20gR29nbywgV2VzdGVybiBBdXN0cmFsaWEuICpab29sb2dpY2FsIEpvdXJuYWwgb2YgdGhlIExpbm5lYW4gU29jaWV0eSogNzM6MjEzLTI1OC4gRE9JIDEwLjExMTEvai4xMDk2LTM2NDIuMTk4MS50YjAxNTk0LnguDQoNCkRlc21vbmQgQUouIDE5NzQuIE9uIHRoZSBjb2Njb3N0ZWlkIGFydGhyb2RpcmUgKk1pbGxlcm9zdGV1cyBtaW5vciouICpab29sb2dpY2FsIEpvdXJuYWwgb2YgdGhlIExpbm5lYW4gU29jaWV0eSogNTQ6Mjc3LTI5OC4gRE9JIDEwLjExMTEvai4xMDk2LTM2NDIuMTk3NC50YjAwODA0LnguDQoNCkVuZ2VsbWFuIFJLLiAyMDIzLiBBIERldm9uaWFuIEZpc2ggVGFsZTogQSBOZXcgTWV0aG9kIG9mIEJvZHkgTGVuZ3RoIEVzdGltYXRpb24gU3VnZ2VzdHMgTXVjaCBTbWFsbGVyIFNpemVzIGZvciAqRHVua2xlb3N0ZXVzIHRlcnJlbGxpKiAoUGxhY29kZXJtaTogQXJ0aHJvZGlyYSkuICpEaXZlcnNpdHkqIDE1OjMxOC4gRE9JIDEwLjMzOTAvZDE1MDMwMzE4Lg0KDQpGZXJyw7NuIEhHLCBNYXJ0aW5lei1QZXJleiBDLCBhbmQgQm90ZWxsYSBILiAyMDE3LiBFY29tb3JwaG9sb2dpY2FsIGluZmVyZW5jZXMgaW4gZWFybHkgdmVydGVicmF0ZXM6IHJlY29uc3RydWN0aW5nICpEdW5rbGVvc3RldXMgdGVycmVsbGkqIChBcnRocm9kaXJhLCBQbGFjb2Rlcm1pKSBjYXVkYWwgZmluIGZyb20gcGFsYWVvZWNvbG9naWNhbCBkYXRhLiAqUGVlckoqIDU6ZTQwODEuIERPSSAxMC43NzE3L3BlZXJqLjQwODEuDQoNCkZyYW5jaXMgTVAuIDIwMDYuIE1vcnBob21ldHJpYyBtaW5lZmllbGRz4oCUdG93YXJkcyBhIG1lYXN1cmVtZW50IHN0YW5kYXJkIGZvciBjaG9uZHJpY2h0aHlhbiBmaXNoZXMuICpFbnZpcm9ubWVudGFsIEJpb2xvZ3kgb2YgRmlzaGVzKiA3Nzo0MDctNDIxLiBET0kgMTAuMTAwNy9zMTA2NDEtMDA2LTkxMDktMS4NCg0KR2FyZGluZXIgQkcsIGFuZCBNaWxlcyBSUy4gMTk5NC4gRXVicmFjaHl0aG9yYWNpZCBhcnRocm9kaXJlcyBmcm9tIEdvZ28sIFdlc3Rlcm4gQXVzdHJhbGlhLiAqWm9vbG9naWNhbCBKb3VybmFsIG9mIHRoZSBMaW5uZWFuIFNvY2lldHkqIDExMjo0NDMtNDc3LiBET0kgMTAuMTExMS9qLjEwOTYtMzY0Mi4xOTk0LnRiMDAzMzEueC4NCg0KR2FycmljayBKQUYuIDE5NjcuIFJldmlzaW9uIG9mIHNoYXJrcyBvZiBnZW51cyAqSXN1cnVzKiB3aXRoIGRlc2NyaXB0aW9uIG9mIGEgbmV3IHNwZWNlcyAoR2FsZW9pZGVhLCBMYW1uaWRhZSkuICpQcm9jZWVkaW5ncyBvZiB0aGUgVW5pdGVkIFN0YXRlZCBOYXRpb25hbCBNdXNldW0qIDExODo2NjPigJM2OTAuDQoNCkdpbG1vcmUgUkcuIDE5ODMuIE9ic2VydmF0aW9ucyBvbiB0aGUgRW1icnlvcyBvZiB0aGUgTG9uZ2ZpbiBNYWtvLCAqSXN1cnVzIHBhdWN1cyosIGFuZCB0aGUgQmlnZXllIFRocmVzaGVyLCAqQWxvcGlhcyBzdXBlcmNpbGlvc3VzKi4gKkNvcGVpYSogMTk4MzozNzUtMzgyLiBET0kgMTAuMjMwNy8xNDQ0MzgwLg0KDQpIeWRlIEpFLCBhbmQgSHV3aWcgSy4gMjAwNC4gSW1hZ2VzIGZyb20gdGhlIEplc3NlIEVhcmwgSHlkZSBDb2xsZWN0aW9uLCBDYXNlIFdlc3Rlcm4gUmVzZXJ2ZSBVbml2ZXJzaXR5IChDV1JVKSBEZXBhcnRtZW50IG9mIEdlb2xvZ2ljYWwgU2NpZW5jZXMuICpBdmFpbGFibGUgYXQgaHR0cHM6Ly9jYXNsYWJzLmNhc2UuZWR1L2h5ZGUtY29sbGVjdGlvbi8qIChhY2Nlc3NlZCBTZXB0ZW1iZXIgMSAyMDIyKS4NCg0KTG93cnkgRCwgZGUgQ2FzdHJvIEFMRiwgTWFyYSBLLCBXaGl0ZW5hY2sgTEIsIERlbGl1cyBCLCBCdXJnZXNzIEdILCBhbmQgTW90dGEgUC4gMjAwOS4gRGV0ZXJtaW5pbmcgc2hhcmsgc2l6ZSBmcm9tIGZvcmVuc2ljIGFuYWx5c2lzIG9mIGJpdGUgZGFtYWdlLiAqTWFyaW5lIEJpb2xvZ3kqIDE1NjoyNDgzLTI0OTIuIERPSSAxMC4xMDA3L3MwMDIyNy0wMDktMTI3My0zLg0KDQpNaWxlcyBSUywgYW5kIERlbm5pcyBLLiAxOTc5LiBBIHByaW1pdGl2ZSBldWJyYWNoeXRob3JhY2lkIGFydGhyb2RpcmUgZnJvbSBHb2dvLCBXZXN0ZXJuIEF1c3RyYWxpYS4gKlpvb2xvZ2ljYWwgSm91cm5hbCBvZiB0aGUgTGlubmVhbiBTb2NpZXR5KiA2NjozMS02Mi4gRE9JIDEwLjExMTEvai4xMDk2LTM2NDIuMTk3OS50YjAxOTAwLnguDQoNCk1pbGVzIFJTLCBhbmQgV2VzdG9sbCBUUy4gMTk2OC4gVGhlIFBsYWNvZGVybSBGaXNoICpDb2Njb3N0ZXVzIGN1c3BpZGF0dXMqIE1pbGxlciBleCBBZ2Fzc2l6IGZyb20gdGhlIE1pZGRsZSBPbGQgUmVkIFNhbmRzdG9uZSBvZiBTY290bGFuZC4gUGFydCBJLiBEZXNjcmlwdGl2ZSBNb3JwaG9sb2d5LiAqVHJhbnNhY3Rpb25zIG9mIHRoZSBSb3lhbCBTb2NpZXR5IG9mIEVkaW5idXJnaCogNjc6MzczLTQ3Ni4gRE9JIDEwLjEwMTcvUzAwODA0NTY4MDAwMjQwNzguDQoNCk1pbGVzIFJTLCBhbmQgV2hpdGUgRUkuIDE5NzEuIFRoZSBIb2xvbmVtYXRpZGFlIChwbGFjb2Rlcm0gZmlzaGVzKSwgYSByZXZpZXcgYmFzZWQgb24gbmV3IHNwZWNpbWVucyBvZiAqSG9sb25lbWEqIGZyb20gdGhlIFVwcGVyIERldm9uaWFuIG9mIFdlc3Rlcm4gQXVzdHJhbGlhLiAqUGhpbG9zb3BoaWNhbCBUcmFuc2FjdGlvbnMgb2YgdGhlIFJveWFsIFNvY2lldHkgb2YgTG9uZG9uIEIsIEJpb2xvZ2ljYWwgU2NpZW5jZXMqIDI2MzoxMDEtMjM0LiBET0kgMTAuMTA5OC9yc3RiLjE5NzEuMDExMS4NCg0KUGllcmN5IEFOLCBDYXJsc29uIEpLLCBTdWxpa293c2tpIEpBLCBhbmQgQnVyZ2VzcyBHSC4gMjAwNy4gQWdlIGFuZCBncm93dGggb2YgdGhlIHNjYWxsb3BlZCBoYW1tZXJoZWFkIHNoYXJrLCAqU3BoeXJuYSBsZXdpbmkqLCBpbiB0aGUgbm9ydGgtd2VzdCBBdGxhbnRpYyBPY2VhbiBhbmQgR3VsZiBvZiBNZXhpY28uICpNYXJpbmUgYW5kIEZyZXNod2F0ZXIgUmVzZWFyY2gqIDU4OjM0LTQwLg0KDQpQb2xsYWNrIEFHLCBEcmlnZ2VycyBXQiwgSUlJLCBIYW5pc2tvIERTLCBhbmQgSW5ncmFtIEdXLCBKci4gMjAxOS4gRGlzdHJpYnV0aW9uIGFuZCBMZW5ndGggRGF0YSBmb3IgQmxhY2t0aXAgU2hhcmtzIENhcHR1cmVkIG9uIHRoZSBOT0FBL05NRlMvU0VGU0MvTVNMQUJTIEJvdHRvbSBMb25nbGluZSBTdXJ2ZXkgaW4gdGhlIFdlc3Rlcm4gTm9ydGggQXRsYW50aWMgT2NlYW4uIE5vcnRoIENoYXJsZXN0b246IFNFREFSLiBwIDE2Lg0KDQpSYW5kYWxsIEpFLiAxOTk3LiBSYW5kYWxsJ3MgdGFuayBwaG90b3MuIENvbGxlY3Rpb24gb2YgMTAsMDAwIGxhcmdlLWZvcm1hdCBwaG90b3MgKHNsaWRlcykgb2YgZGVhZCBmaXNoZXMuICpBdmFpbGFibGUgYXQgd3d3LmZpc2hiYXNlLm9yZyAoc2VlIHNwZWNpZXMgcGFnZXMgZm9yIG1vcmUgZGV0YWlscykqIChhY2Nlc3NlZCBKdW5lIDI5IDIwMjIpLg0KDQpUcmluYWpzdGljIEsuIDE5OTkuIE5ldyBhbmF0b21pY2FsIGluZm9ybWF0aW9uIG9uICpIb2xvbmVtYSogKFBsYWNvZGVybWkpIGJhc2VkIG9uIG1hdGVyaWFsIGZyb20gdGhlIEZyYXNuaWFuIEdvZ28gRm9ybWF0aW9uIGFuZCB0aGUgR2l2ZXRpYW4tRnJhc25pYW4gR25ldWRuYSBGb3JtYXRpb24sIFdlc3Rlcm4gQXVzdHJhbGlhLiAqR2VvZGl2ZXJzaXRhcyogMjE6NjktODQuDQoNClRyaW5hanN0aWMgSywgQnJpZ2dzIERFRywgYW5kIExvbmcgSkEuIDIwMjIuIFRoZSBHb2dvIEZvcm1hdGlvbiBMYWdlcnN0w6R0dGU6IGEgdmlldyBvZiBBdXN0cmFsaWEncyBmaXJzdCBncmVhdCBiYXJyaWVyIHJlZWYuICpKb3VybmFsIG9mIHRoZSBHZW9sb2dpY2FsIFNvY2lldHkqIDE3OTpqZ3MyMDIxLTIxMDUuIERPSSAxMC4xMTQ0L2pnczIwMjEtMTA1Lg0KDQpUcmluYWpzdGljIEssIFNhbmNoZXogUywgRHVwcmV0IFYsIFRhZmZvcmVhdSBQLCBMb25nIEosIFlvdW5nIEcsIFNlbmRlbiBULCBCb2lzdmVydCBDLCBQb3dlciBOLCBhbmQgQWhsYmVyZyBQRS4gMjAxMy4gRm9zc2lsIG11c2N1bGF0dXJlIG9mIHRoZSBtb3N0IHByaW1pdGl2ZSBqYXdlZCB2ZXJ0ZWJyYXRlcy4gKlNjaWVuY2UqIDM0MToxNjAtMTY0LiBET0kgMTAuMTEyNi9zY2llbmNlLjEyMzcyNzUuDQoNClbDqXppbmEgRC4gMTk4OC4gKlBsb3VyZG9zdGV1cyBjYW5hZGVuc2lzKiAoV29vZHdhcmQgMTg5MiksIHVuIEFydGhyb2RpcmUgZHUgRnJhc25pZW4gaW5mw6lyaWV1ciBkdSBDYW5hZGE6IGNvbnRyaWJ1dGlvbiDDoCBsJ8OpdHVkZSBtb3JwaG9sb2dpcXVlIGV0IHBoeWxvZ8OpbsOpdGlxdWUgZGVzIFBsb3VyZG9zdGVpZGFlIChWZXJ0ZWJyYXRhLCBQbGFjb2Rlcm1pKSBkdSBEw6l2b25pZW4gbW95ZW4gZXQgc3Vww6lyaWV1ci4gUGhELiBsJ1VuaXZlcnNpdMOpIGRlIFBhcmlzIFZJSS4NCg0KVsOpemluYSBELiAxOTkwLiBMZXMgUGxvdXJkb3N0ZWlkYWUgZmFtLiBub3YuIChQbGFjb2Rlcm1pLCBBcnRocm9kaXJhKSBldCBsZXVycyByZWxhdGlvbnMgcGh5bMOpdGlxdWVzIGF1IHNlaW4gZGVzIEJyYWNoeXRob3JhY2kuICpDYW5hZGlhbiBKb3VybmFsIG9mIEVhcnRoIFNjaWVuY2VzKiAyNzo2NzctNjgzLiBET0kgMTAuMTEzOS9lOTAtMDY1Lg0KDQpXaGl0bmV5IE5NLCBhbmQgQ3JvdyBHTC4gMjAwNy4gUmVwcm9kdWN0aXZlIGJpb2xvZ3kgb2YgdGhlIHRpZ2VyIHNoYXJrICgqR2FsZW9jZXJkbyBjdXZpZXIqKSBpbiBIYXdhaWkuIE1hcmluZSBCaW9sb2d5IDE1MTo2My03MC4gRE9JIDEwLjEwMDcvczAwMjI3LTAwNi0wNDc2LTAuDQo8L2Rpdj4NCg0KIyBTZXNzaW9uIEluZm9ybWF0aW9uDQoNCmBgYHtyfQ0KeGZ1bjo6c2Vzc2lvbl9pbmZvKCkNCmBgYA==

×

- 1 Loading in data
  - 1.1 Separating out fossil taxa
  - 1.2 Loading shark data from Lowry et al. (2009) used by Ferron et al. 2017
  - 1.3 Loading in jaw data for FSBC sharks
- 2 Data
  - 2.1 Measurements
  - 2.2 Taxonomic distribution of observations
- 3 Additional notes on data collection
  - 3.1 Method of collecting measurements in Arthrodira
  - 3.2 Length-length equations for sharks
  - 3.3 Note on the ventral armor of *Dunkleosteus*
- 4 Shark UJP plot
  - 4.1 Using Precaudal Length
- 5 Comparison of estimates using upper jaw perimeter and mouth width
  - 5.1 Specimen-level analysis
    - 5.1.1 Diagnostic plots
    - 5.1.2 Allometric relationship
  - 5.2 Examining the allometric relationship of mouth width using species averages
    - 5.2.1 Regression equation
    - 5.2.2 Allometric relationship
  - 5.3 Comparison of estimates using UJP and mouth width in arthrodires
  - 5.4 Testing accuracy of UJP and mouth width in estimating total length in sharks
  - 5.5 Using *Brachyplatystoma* as a Case Study
- 6 Using mouth width to estimate total length in arthrodires
  - 6.1 Mouth width versus total length in chondrichthyans (and arthrodires)
  - 6.2 Testing using inner mouth width in arthrodires
  - 6.3 Mouth width versus total length in osteichthyans (and arthrodires)
  - 6.4 Error in using mouth proportions in sharks to estimate length in complete arthrodires
  - 6.5 Estimating mouth size in arthrodires like *Coccosteus* assuming similar proportions to sharks
- 7 Estimating the length of arthrodires using approximated UJP
  - 7.1 Testing how closely estimated UJP matches actual UJP in FSBC Sharks
  - 7.2 Comparing estimated UJP to actual UJP in arthrodires
  - 7.3 Length estimates in arthrodires using estimated UJP
  - 7.4 Scatterplot of UJP versus total length
  - 7.5 Relative size of UJP in *Coccosteus*
- 8 Diagnostic plots for UJP and mouth width
  - 8.1 UJP
  - 8.2 Mouth Width
- 9 Plotting against head length
  - 9.1 Highlighting taxa with elongate trunks
  - 9.2 Using precaudal length
  - 9.3 Treating head length in arthrodires as equivalent to prebranchial length
  - 9.4 Highlighting groupers
  - 9.5 Using lengths of Engelman (2023)
- 10 Mouth length
- 11 References
- 12 Session Information
