# Supplementary figures and images for: Giant, swimming mouths: oral dimensions of extant sharks do not accurately predict body size in Dunkleosteus terrelli (Placodermi: Arthrodira)
Source: PeerJ. 2023 Apr 10;11:e15131. doi: 10.7717/peerj.15131 (PMC10100833; doi:10.7717/peerj.15131)

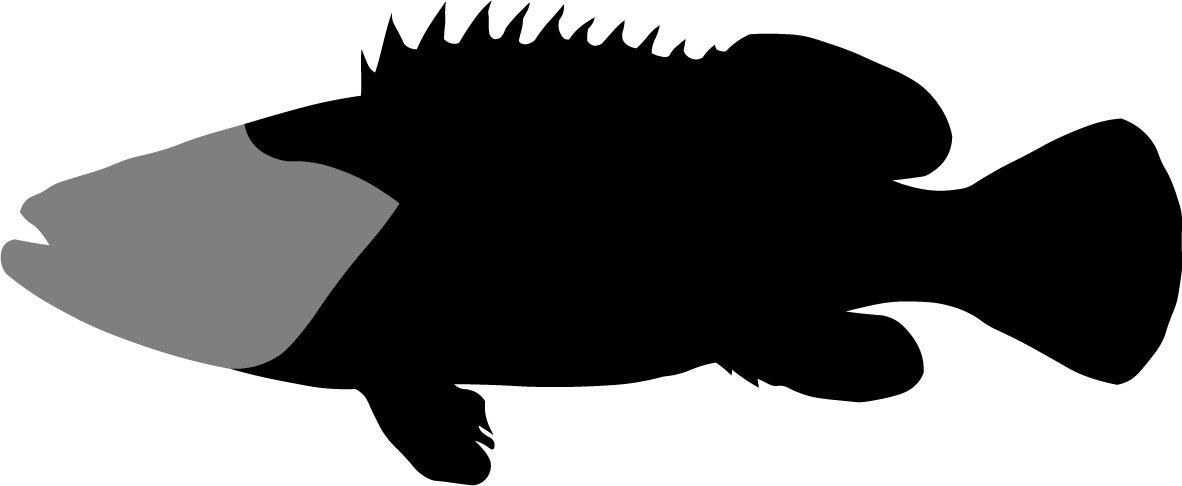

Supplement: Supplemental Information 5 — This image is used to produce Fig. 5, and necessary to rerun the code. Silhouette modified from image in Randall (1997). [file peerj-11-15131-s005.png]

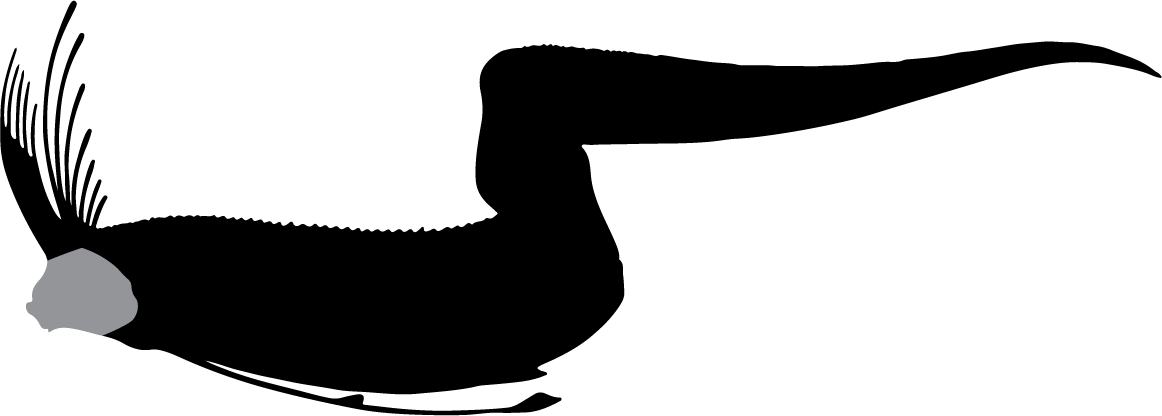

Supplement: Supplemental Information 7 — This image is used to produce Fig. 5, and necessary to rerun the code. Regalecus silhouette modified from image by John Norris Wood. [file peerj-11-15131-s007.png]

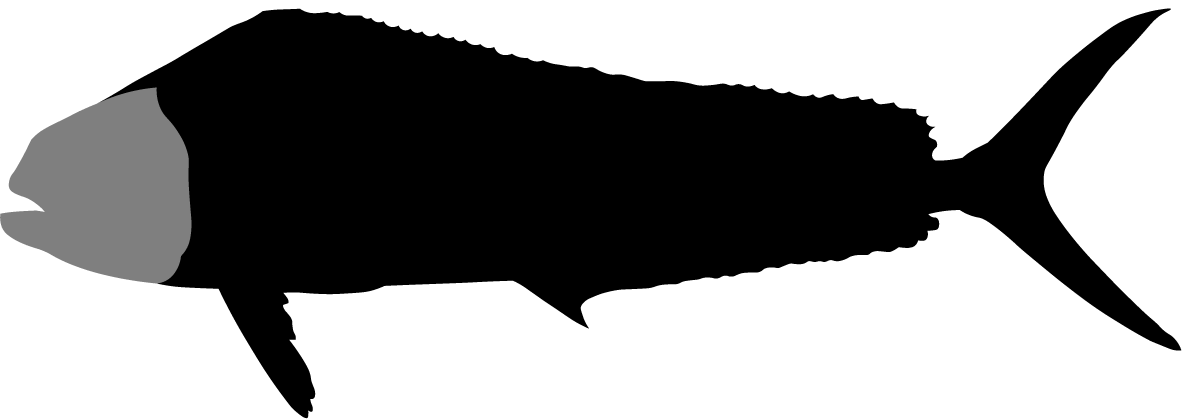

Supplement: Supplemental Information 8 — This image is used to produce Fig. 5, and necessary to rerun the code. Corphaena silhouette modified from image by Richard Winterbottom (from https://www.fishbase.se/photos/PicturesSummary.php?StartRow=10&ID=6&what=species&TotRec=21, used with permission). [file peerj-11-15131-s008.png]

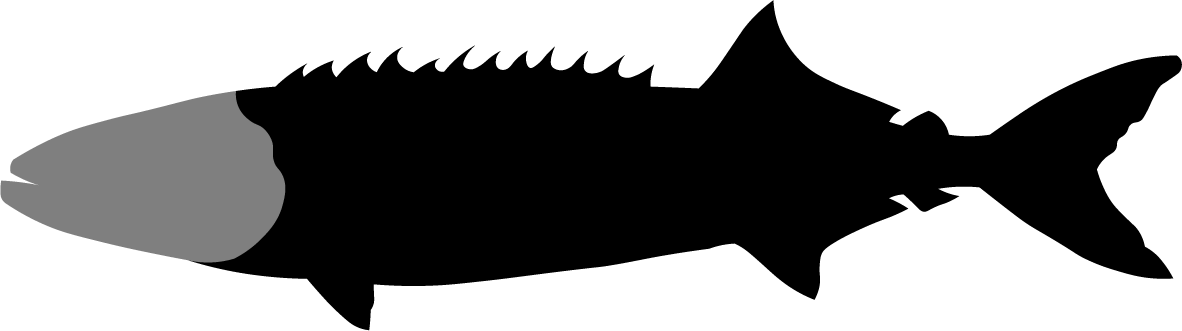

Supplement: Supplemental Information 9 — This image is used to produce Fig. 5, and necessary to rerun the code. Silhouette modified from image in Randall (1997). [file peerj-11-15131-s009.png]
