# Supplementary material for: Giant, swimming mouths: oral dimensions of extant sharks do not accurately predict body size in Dunkleosteus terrelli (Placodermi: Arthrodira)
Source: PeerJ. 2023 Apr 10;11:e15131. doi: 10.7717/peerj.15131 (PMC10100833; doi:10.7717/peerj.15131)
Supplement: Supplemental Information 6 [file peerj-11-15131-s006.docx]

# Institutional Abbreviations

**AA.MEM.DS.8** – Université Cadi Ayyad, Marrakech, Morocco

**AMNH** – American Museum of Natural History, New York, New York, USA

**AMS** – Australian Museum, Sydney, Australia

**ANSP** – Academy of Natural Sciences, Philadelphia, Pennsylvania, USA

**ASIZP** – Biodiversity Research Museum, Chinese Academy of Sciences, Nankang, Taipei City, Taiwan

**AtlantNIRO** – Atlantic Scientific Research Institute of Marine Fisheries and Oceanography, Kaliningrad, Russia

**AUBM** – American University of Beirut, Beirut, Lebanon

**AZUSC** – Acervo Zoológico da Universidade Santa Cecília, Santos, Brazil

**BMNH** – The Natural History Museum (London), London, U.K.

Note: these are specimens referred to in older studies before the institutional abbreviation change to NHMUK, abbreviations are kept the same here for easier reference to cited study.

**BPBM** - Bernice Pauahi Bishop Museum, Department of Zoology, Honolulu, Hawaii, USA

**BRT-I** – Blue Resources Trust Ichthyology Collection, Sri Lanka

**CAS (incl. CAS SU)** – California Academy of Sciences, San Francisco, California, USA

**CBM-ZF** – Natural History Museum & Institute, Chiba, Japan

**CCC** – Coelacanth Conservation Council (no address). Numbers are not specimen numbers but internal numbering system within cited references.

**CICIMAR-CI** – Ichthyological Collection, Centro Interdisciplinario de Ciencias Marinas, La Paz, Baja California Sur, Mexico

**CI-UABC** – Ichthyology Collection, Universidad Autónoma de Baja California, Ensenada, Baja California, Mexico

**CMC** – Canterbury Museum, Christchurch, New Zealand

**CMFRI** – Marine Biodiversity Museum, Central Marine Fisheries Research Institute, Kochi, India

**CMK** – Maurice Kottelat, personal collection

**CMNH** – Cleveland Museum of Natural History, Cleveland, Ohio, USA

**CNPE-IBUNAM** – Colección Nacional de Peces of the Instituto de Biología, Universidad Nacional Autónoma de México, Mexico City, Mexico

**CNPICT** – Centro Nacional Patagónico, Puerto Madryn, Argentina

**COLETA** – Institute of Marine Research (IMAR - Azores), Portugal

**CSIRO** – Australian National Fish Collection, Hobart, Tasmania

**DSZ** – Department of Animal Science, University of Sassari, Sassari, Italy

**DU-FS** - KaziZaker Husain Museum of the Department of Zoology, University of Dhaka, Dhaka, Bangladesh

**EBFS** – Department of Environmental Biology and Fisheries Science, National Taiwan Ocean University, Keelung, Taiwan.

**ECOSC** – El Colegio de la Frontera Sur, San Cristóbal de Las Casas, Mexico

**ERB** – Elasmobranch Research Belgium, Berlaar, Belgium

**ESFM-PIS** – Fisheries Faculty, Ege University, Bornova/İzmir, Turkey

**FAKU** – Kyoto University Museum, Kyoto, Japan

**FMNH** – The Field Museum, Chicago, USA

**FMRI (MMMC)** – Marine Biodiversity Museum of Mandapam Regional Centre, Central Marine Fisheries Research Institute (CMFRI), Mandapam, Ramanathapuram District, India

**FSB** – Faculté des Sciences Biologiques (FSB), Université des sciences et de la technologie Houari-Boumediene (USTHB), Bab Ezzouar, Algeria

**FSBC** – Florida State Biodiversity Collection, St. Petersburg, Florida, USA

**FST** – Faculté des Sciences de Tunis, University of Tunis El Manar, Tunis, Tunisia

**FUMT** – Department of Fisheries, University Museum, University of Tokyo (FUMT) [specimens now catalogued in National Science Museum, Department of Zoology, Tokyo, Japan]

**GAFR** – Ichthyological Collection of the General Commission for Fisheries Resources Lattakia, Syria

**GCRL** – Gulf Coast Research Laboratory Museum, Ocean Springs, USA

**GEA** – Laboratório de Ictiologia do Grupo de Ecologia Aquática, Universidade Federal do Pará, Belém, Brazil

**GVF** – George Vanderbilt Foundation, Stanford, USA [Specimens now at California Academy of Science]

**HUJ** – The Hebrew University of Jerusalem, Jerusalem, Israel

**HUMZ** – Hokkaido University Museum, Sapporo, Japan

**HVM** – Heda Village Museum, Heda, Shizuoka, Japan

**IC-MBP** – Marine Biology Station, National Institute of Biology, Piran, Slovenia

**IFAN** - Institut Fondamental d’Afrique noire Cheikh Anta Diop de Dakar, Senegal

**IFREDI-PO** – Inland Fisheries Research and Development Institute, Phnom Penh, Cambodia

**INHS** – Illinois Natural History Survey, University of Illinois at Urbana-Champaign, Champaign

**INPA** – Instituto Nacional de Pesquisas da Amazônia, Manaus, Brazil

**INVEMAR** – Instituto de Investigaciones Marinas y Costeras "José Benito Vives de Andréis", Museo de Historia Natural Marina de Colombia (MHNMC), Rodadero Sur, Santa Marta, Colombia

**IPPS** – Institut Penyelidikan Perikanan Sarawak [Fisheries Research Institute Sarawak (FRIS)], Kuching, Malaysia

**IRDN** – Institut de recherche pour le développement (IRD), Nouméa, New Caledonia

**IVPP** – Institute of Vertebrate Paleontology and Paleoanthropology, Chinese Academy of Sciences [Academia Sinica], Beijing, China

**JNC** – Unclear, may pertain to specimens in MNHN Paris

**JNU** – Fish Genetics and Breeding Laboratory, Jeju National University, Jeju City, Korea

**KA** – University of Canterbury, Edward Percival Field Centre, Kaikoura, New Zealand

**KAU** - King Abdulaziz University Marine Museum, Jeddah, Saudi Arabia

**KAUM** – Kagoshima University Museum, Kagoshima, Japan

**KBF-I** – Kuroshio Biological Research Foundation, Kochi, Japan

**KPM-NI** – Kanagawa Prefectural Museum of Natural History, Odawara. Includes: fishes fromt Izu Oceanic Park (IOP, IOPM), Ito, Shizuoka, Japan

**LACM** – Los Angeles County Museum of Natural History, Los Angeles, California, USA

**LBP** – Laboratório de Biologia e Genética de Peixes (LBGP), Departamento de Morfologia, Universidade Estadual Paulista "Júlio de Mesquita Filho" (UNESP), Campus de Botucatu

**LGE-P** – Laboratório de Genética de Peixes, Departamento de Biologia, Instituto de Biociências, Universidade Estadual Paulista "Júlio de Mesquita Filho" UNESP), Campus de Rio Claro, Brazil

**LJVC** – L. J. V. Compagno personal collection

**MBUCV** - Museo de Biología de la Universidad Central de Venezuela, Caracas, Venezula

**MCP** - Museu de Ciências e Tecnologia (MCT), Pontifícia Universidade Católica do Rio Grande do Sul (PUCRS), Porto Alegre, Rio Grande do Sul, Brazil

**MCZ** – Museum of Comparative Zoology, Harvard University, Cambridge, USA

**MEPN** – Museo de Historia Natural "Gustavo Orcés V.", Instituto de Ciencias Biológicas, Escuela Politécnica Nacional, Quito

**MEUFC** – Museum of the Systematics, Faculty of Fisheries, Mersin University, Mersin, Turkey

**MHNUSC** – Museo de Historia Natural da Universidade de Santiago de Compostela, Santiago de Compostela

**MNHM** – Musée d'Histoire Naturelle de Miguasha, Quebec, Canada

**MNHN** – Muséum national d'Histoire naturelle, Paris, France

**MNHNC** – Museo Nacional de Historia Natural, Santiago. Includes: fish collections from the Universidad de Chile, Santiago, Chile

**MNRJ** – Museu Nacional, Universidade Federal do Rio de Janeiro, Rio de Janeiro, Brazil

**MPEG** – Museu Paraense "Emilio Goeldi", Belém, Brazil

**RCMA (MRAC)** – Royal Museum for Central Africa [Musée royal de l'Afrique centrale (MRAC), Koninklijk Museum voor Midden-Afrika (KMMA)], Tervuren, Belgium

**ROM** – Royal Ontario Museum, Toronto, Canada

**MSCB** – Marine Science Center, University of Basra, Basra, Iraq

**MSL** – Marine Sciences Laboratory, Agriculture Faculty at Tishreen University, Latakia, Syria

**MSNC**– Museo di Storia Naturale di Comiso, Comiso, Italy

**MUFS** – Division of Fisheries Science, University of Miyazaki, Miyazaki, Japan

**MUSM** – Museo de Historia Natural, Universidad Nacional Mayor de San Marcos, Lima, Peru

**MZB** – Bidang Zoologi, Pusat Penelitian Biologi, Lembaga Ilmu Pengetahuan Indonesia (LIPI), Cibinong, Bogor Regency, Indonesia

**MZL** - Musée Cantonal de Zoologie, Lausanne, Switzerland

**MZUSP** - Museu de Zoologia da Universidade de São Paulo, São Paulo, Brazil

**NBFGR/PP**

**NHMUK** – Natural History Museum (London), London, United Kingrom

**NIBR** – National Institute of Biological Resources, Korean Ministry of the Environment, Incheon, South Korea

**NMMB-P** - National Museum of Marine Biology, Pingtung, Taiwan

**NMNHS** – National Museum of Natural History, Bulgarian Academy of Sciences, Sofia, Bulgaria

**NMNZ** - Museum of New Zealand Te Papa Tongarewa, Wellington, New Zealand

**NMS** – National Museum of Scotland, Edinburgh, Scotland, United Kingdom

**NMST-P** - National Science Museum, Department of Zoology, Tokyo, Japan

**NMV** – Museums Victoria, Melbourne, Australia

**NMW** – Naturhistorisches Museum, Wien [Vienna], Austria

**NRM** – Naturhistoriska riksmuseet [Swedish Museum of Natural History], Departments of Zoology and Paleontology, Stockholm, Sweden

**NSMT** – National Museum of Nature and Science, Zoology Department, Division of Fishes, Tsukuba, Ibaraki, Japan

**NTM S** – Museum and Art Gallery of the Northern Territory, Darwin, Australia

**NMW** – Naturhistorisches Museum, Vienna, Austria

**OMNH-P** – Osaka Museum of Natural History, Osaka, Japan

**ORID** – Oceanographic Research Institute, South African Association for Marine Biological Research (SAAMBR), Durban, South Africa

**OSUM** – Ohio State University Museum of Biological Diversity, Columbus, Ohio, USA

**PKU** – Pukyong National University, Department of Marine Biology, Busan, South Kora

**PMBC** – Phuket Marine Biological Centre, Phuket, Thailand

**PSUZC** – Princess Maha Chakri Sirindhorn Natural History Museum, Prince of Songkla University in Hat Yai, Songkhla, Thailand

**PU/B92N** – Museum of Department of Ocean Studies and Marine Biology, Pondicherry University, Port Blair Campus, Andaman Islands

**RMNH** – Rijksmuseum van Natuurlijke Historie, Leiden, Netherland

**ROM** – Royal Ontario Museum, Toronto, Ontario

**RUSI/SAIAB –** South African Institute for Aquatic Biodiversity, Makhanda [Grahamstown], South Africa

**SAM** – South African Museum, Cape Town, South Africa

**SCUM** – Sichuan University Museum, Chengdu, China

**SFRI** – Institut Penyelidikan Perikanan Sarawak/Sarawak Fisheries Research Institute, Kuching, Malaysia

**SG** – S. H. Gruber personal collection

**SIO** – Scripps Institution of Oceanography, La Jolla, USA

**SM** – *Shoyo Maryu* field number

**SMBL-F** – Seto Marine Biological Laboratory of Kyoto University, Shirahama, Japan

**SMF** – Senckenberg Forschungsinstitut und Naturmuseum [Senckenberg Research Institute and Natural History Museum], Frankfurt am Main, Germany

**SMNK** – Staatliches Museum für Naturkunde Karlsruhe [Karlsruher Naturkundemuseum], Karlsruhe, Germany

**SNM** – Slovak National Museum [Slovenské národné múzeum], Natural Science Museum, Bratislava, Slovakia

**STRI** – Smithsonian Tropical Research Institute, Balboa, Panamá

**TAMAR** – Projeto Tamar, São João da Mata, Brazil

**TAMBL** – Toba Aquarium, Toba, Japan

**TAU** – Tel Aviv University, Tel Aviv, Israel

**TCWC** – Biodiversity Research and Teaching Collections (BRTC), Department of Wildlife and Fisheries Sciences, Texas A&M University, College Station, USA

**TMFE** – Elasmobranchii Collection, Department of Fisheries, Faculty of Marine Science and Technology, Tokai University, Shimizu, Shizuoka, Japan

**TU** – Tulane University, New Orleans, USA

**UCR** – Museo de Zoología, Escuela de Biología, Universidad de Costa Rica, Costa Rica

**UF** – Florida Museum of Natural History, Gainesville, Florida, USA

**UFBA** – Museu de Zoologia da Universidade Federal da Bahia, Salvador, Brazil

**UFRO** – Universidade Federal de Rondônia, Porto Velho, Brazil

**UMCZ** – University Museum of Zoology, University of Cambridge, Cambridge, United Kingdom

**UMML** – University of Miami Institute of Marine Science, Miami, USA

**UMMZ** – University of Michigan Museum of Zoology, Ann Arbor, USA

**UNC** – University of North Carolina at Chapel Hill, Institute of Marine Sciences, Morehead City

Note: these specimens now in the collections at the North Carolina Museum of Natural Sciences (NCSM), but the revised specimen numbers are unknown.

**UNPSJB** - Universidad Nacional de la Patagonia San Juan Bosco, Chubut Province, Argentina

**UNS** – University of Science, Hồ Chí Minh City, Vietnam

**URM-P** – Yoshino collection from the University of the Ryukyus, currently at Okinawa Churashima Foundation Research Center, Kunigami, Japan

**USNM** – Smithsonian National Museum of Natural History, Washington D.C., USA

**UWBM** – Burke Museum of Natural and Science, Seattle, USA

**WAM** ­– Western Australian Museum, Perth, Australia

**YCM** – Yokosuka City Museum, Yokosuka, Japan

**ZMB** - Museum für Naturkunde, Berlin, Germany

**ZMH** – Centrum für Naturkunde (CeNak), Zoologisches Museum, Universität Hamburg, Hamburg, Germany

**ZMUB** - Bergen Museum, University of Bergen, Bergen, Norway

**ZRC** – Zoological Reference Collection, Lee Kong Chian Natural History Museum, Singapore

**ZSI** – Zoological Survey of India, Indian Museum, Kolkata, India

**ZSI/ANRC** – Zoological Survey of India, Port Blair, India

**ZSI/CLT** – Zoological Survey of India, Kozhikode, India

**ZSM** – Bavarian State Collections of Zoology, Munich, Germany

**ZUMT** – The University Museum, University of Tokyo, Tokyo, Japan

**ZVC** – Zoological Vertebrate Collection-Peces, Museum of Natural History, Montevideo, Uruguay

Every effort has been made to list the abbreviations used here, however given the large size of the dataset and the fact that many of these data come from the previously published literature rather than measured in person means that some may have slipped through the cracks. All specimens for which data was measured directly by the author have their specimen numbers listed. In the event that a specimen number is accidentally not listed here, please consult Sabaj (2020) for a comprehensive list of institutional abbreviations in ichthyology.

Sabaj MH. 2020. Codes for Natural History Collections in Ichthyology and Herpetology. *Copeia* 108:593-669. DOI 10.1643/ASIHCODONS2020.

# Arthrodira

***Dunkleosteus***

Branson EB. 1908. Notes on *Dinichthys terrelli* Newberry, with a restoration. *The Ohio Naturalist* 8:363-369.

Branson EB. 1911. Notes on the Ohio Shales and their faunas. *The University of Missouri Bulletin Science Series* 2:23-32.

Johanson Z, Trinajstic K, Cumbaa S, and Ryan MJ. 2019. Fusion in the vertebral column of the pachyosteomorph arthrodire *Dunkleosteus terrelli* (‘Placodermi’). *Palaeontologia Electronica* 22.2.20A. DOI 10.26879/872.

***Heintzichthys***

Heintz A. 1931. A reconstruction of *Stenognathus gouldi*. *Annals and Magazine of Natural History* 8:242–249.

***Coccosteus***

Miles RS, and Westoll TS. 1968. The Placoderm Fish *Coccosteus cuspidatu*s Miller ex Agassiz from the Middle Old Red Sandstone of Scotland. Part I. Descriptive Morphology. *Transactions of the Royal Society of Edinburgh* 67:373-476. DOI 10.1017/S0080456800024078.

***Africanaspis***

Gess RW, and Trinajstic KM. 2017. New morphological information on, and species of placoderm fish *Africanaspis* (Arthrodira, Placodermi) from the Late Devonian of South Africa. *PLoS ONE* 12:e0173169. DOI 10.1371/journal.pone.0173169.

**Gogo Formation Arthrodires**

Dennis K, and Miles RS. 1979. Eubrachythoracid arthrodires with tubular rostral plates from Gogo, Western Australia. *Zoological Journal of the Linnean Society* 67:297-328. DOI 10.1111/j.1096-3642.1979.tb01118.x.

Dennis K, and Miles RS. 1981. A pachyosteomorph arthrodire from Gogo, Western Australia. *Zoological Journal of the Linnean Society* 73:213-258. DOI 10.1111/j.1096-3642.1981.tb01594.x.

Gardiner BG, and Miles RS. 1990. A new genus of eubrachythoracid arthrodire from Gogo, Western Australia. *Zoological Journal of the Linnean Society* 99:159-204. DOI 10.1111/j.1096-3642.1990.tb00566.x.

Gardiner BG, and Miles RS. 1994. Eubrachythoracid arthrodires from Gogo, Western Australia. *Zoological Journal of the Linnean Society* 112:443-477. DOI 10.1111/j.1096-3642.1994.tb00331.x.

Long J. 1988. A new camuropiscid arthrodire (Pisces: Placodermi) from Gogo, Western Australia. *Zoological Journal of the Linnean Society* 94:233-258. DOI 10.1111/j.1096-3642.1988.tb01194.x.

Long J. 1994. A second incisoscutid arthrodire (Pisces, Placodermi) from the Late Devonian Gogo Formation, Western Australia). *Alcheringa: An Australasian Journal of Palaeontology* 18:59–69.

Long JA. 1995. A new plourdosteid arthrodire from the Upper Devonian Gogo Formation of Western Australia. *Palaeontology* 38:39-62.

Miles RS, and Dennis K. 1979. A primitive eubrachythoracid arthrodire from Gogo, Western Australia. *Zoological Journal of the Linnean Society* 66:31-62. DOI 10.1111/j.1096-3642.1979.tb01900.x.

Miles RS, and White EI. 1971. The Holonematidae (placoderm fishes), a review based on new specimens of *Holonema* from the Upper Devonian of Western Australia. *Philosophical Transactions of the Royal Society of London B, Biological Sciences* 263:101-234. DOI 10.1098/rstb.1971.0111.

Trinajstic K. 1999. New anatomical information on *Holonema* (Placodermi) based on material from the Frasnian Gogo Formation and the Givetian-Frasnian Gneudna Formation, Western Australia. *Geodiversitas* 21:69-84.

Trinajstic K, and Hazelton M. 2007. Ontogeny, phenotypic variation and phylogenetic implications of arthrodires from the Gogo Formation, Western Australia. *Journal of Vertebrate Paleontology* 27:571-583. DOI 10.1671/0272-4634(2007)27[571:OPVAPI]2.0.CO;2.

Trinajstic K, Sanchez S, Dupret V, Tafforeau P, Long J, Young G, Senden T, Boisvert C, Power N, and Ahlberg PE. 2013. Fossil musculature of the most primitive jawed vertebrates. *Science* 341:160-164. DOI 10.1126/science.1237275.

**Other Taxa**

Boyle J, and Ryan MJ. 2017. New information on *Titanichthys* (Placodermi, Arthrodira) from the Cleveland Shale (Upper Devonian) of Ohio, USA. *Journal of Paleontology* 91:318–336. DOI 10.1017/jpa.2016.136.

Carr RK. 1994. A redescription of *Gymnotrachelus* (Placodermi: Arthrodira) from the Cleveland Shale (Famennian) of northern Ohio, U.S.A. *Kirtlandia* 48:3-21.

Dean B. 1909. Notes on a newly mounted *Titanichthys*. *Memoirs of the American Museum of Natural History* 9:270–271.

Dunkle DH, and Bungart PA. 1940. On one of the least known of the Cleveland Shale Arthrodira. *Kirtlandia* 8:29-47.

Gross W. 1932. Die Arthrodira Wildungens. *Geologische und Paläeontologische Abhandlungen* 19:1-61.

Hussakof L, and Bryant WL. 1918. Catalog of the fossil fishes in the museum of the Buffalo society of natural sciences. *Bulletin of the Buffalo Society of Natural Sciences* 12:1-198.

# Actinopterygii

## Basal Actinopterygii

### Acipenseriformes

Colway C, and Stevenson DE. 2007. Confirmed Records of Two Green Sturgeon from the Bering Sea and Gulf of Alaska. *Northwestern Naturalist* 88:188-192. DOI 10.1898/1051-1733(2007)88[188:Crotgs]2.0.Co;2.

Hata H, Yamada M, and Motomura H. 2018. Morphological and ecological notes on *Acipenser sinensis* (Chondrostei: Acipenseriformes) in Kagoshima Prefecture, Japan. *Nature of Kagoshima* 44:157-161.

Honma Y, and Itano H. 1994. A Record of a Great Siberian Sturgeon, *Huso dauricus*, off Niigata, Sea of Japan (Osteichthyes: Acipenseridae). *Japanese Journal of Ichthyology* 41:317–321.

Kalmykov VA, Ruban GI, and Pavlov DS. 2009. On the populational structure of sterlet *Acipenser ruthenus* (Acipenseridae) from the Volga Lower reaches. *Journal of Ichthyology* 49:339-347. DOI 10.1134/s0032945209040067.

Mikodina EV, Novosadov AG, and Koshelev VN. 2015. On biology of Kaluga sturgeon *Acipenser dauricus* (Acipenseridae) from the Viakhtu River (Northwestern Sakhalin). *Journal of Ichthyology* 55:567-575. DOI 10.1134/s0032945215040062.

Ting H-p. 1949. Notes on a Sturgeon from the Min River, China. *Copeia* 1949:65-68. DOI 10.2307/1437665.

Williams JD, and Clemmer GH. 1991. *Scaphirhynchus suttkusi*, a new sturgeon (Pisces: Acipenseridae) from the Mobile Basin of Alabama and Mississippi. *Bulletin of the Alabama Museum of Natural History*:17-31.

### Lepisosteiformes

McDonald DL, Anderson JD, Hurley C, and Bumguardner BW. 2013. Sexual Dimorphism in Alligator Gar. *North American Journal of Fisheries Management* 33:811–816. DOI 10.1080/02755947.2013.812586.

Snow RA, Porta MJ, and Sager CR. 2020. Length-Weight Relationships and Potential Biases for Alligator Gar (*Atractosteus spatula*) from Texoma Reservoir, Oklahoma. *Proceedings of the Oklahoma Academy of Science* 100:30-37.

### Polypteriformes

Hanssens MM, Teugels GG, and Dirk FETvDA. 1995. Subspecies in the *Polypterus palmas* Complex (Brachiopterygii; Polypteridae) from West and Central Africa. *Copeia* 1995:694–705. DOI 10.2307/1446767.

Schliewen UK, and Schäfer F. 2006. *Polypterus mokelembembe*, a new species of bichir from the central Congo River basin (Actinopterygii: Cladistia: Polypteridae). *Zootaxa* 1129. DOI 10.11646/zootaxa.1129.1.2.

## Basal Teleostei

### Albuliformes

**Albulidae**

Randall JE. 1997. Randall's tank photos. Collection of 10,000 large-format photos (slides) of dead fishes. *Available at www.fishbase.org (see species pages for more details)* (accessed June 29 2022).

### Anguilliformes

Hatooka K, and Randall JE. 1992. A new moray eel (*Gymnothorax*: Muraenidae) from Japan and Hawaii. *Japanese Journal of Ichthyology* 39:183-190.

Li Y, Zhang L, Zhao L, Feng J, Loh K, Zheng X, and Lin L. 2018. New identification of the moray eel *Gymnothorax minor* (Temminck & Schlegel, 1846) in China (Anguilliformes, Muraenidae). *ZooKeys*:149-161. DOI 10.3897/zookeys.752.24231.

Loh K-H, Hussein MAS, Chong V-C, and Sasekumar A. 2015. Notes on the moray eels (Anguiliiformes: Muraenidae) of Malaysia with two new records. *Sains Malaysiana* 44:41-47.

Rafrafi-Nouira S, Souissi JB, and Capapé C. 2018. About teleost species from deep marine Tunisian waters: with additional records of Sloane's viperfish *Chauliodus sloani* and confirmed occurence of blackfin sorcerer *Nettastoma melanurum*. *Annales, Series historia naturalis* 28:51–58. DOI 10.19233/ASHN.2018.08.

Yagi M, Shimoda M, Uchida J, Shimizu K, Aoshima T, and Kanehara H. 2013. Record body size of the beach conger *Conger japonicus* (Anguilliformes: Congridae) in the East China Sea. *Marine Biodiversity Records* 6:e110. DOI 10.1017/S1755267213000882.

### Elopiformes

**Elopidae**

Bovcon ND, Cochia PD, Trobbiani GA, Belleggia M, Jacobi KJ, De Wysiecki AM, Figueroa DE, and Irigoyen AJ. 2022. Southernmost record of *Elops smithi* in the Southwest Atlantic. A tropical species in waters of Patagonia, Argentina. *Journal of Applied Ichthyology* 38:320-324. DOI 10.1111/jai.14299.

McBride RS, Rocha CR, Ruiz-Carus R, and Bowen BW. 2010. A new species of ladyfish, of the genus *Elops* (Elopiformes: Elopidae), from the western Atlantic Ocean. *Zootaxa* 2346:29-41.

**Megalopidae**

Arronte JC, Pis-Millán JA, Fernández MP, and García L. 2004. First records of the subtropical fish *Megalops atlanticus* (Osteichthyes: Megalopidae) in the Cantabrian Sea, northern Spain. *Journal of the Marine Biological Association of the United Kingdom* 84:1091-1092. DOI 10.1017/S0025315404010501h.

Bañón R, Farias C, De Carlos A, Arronte JC, Varela JL, Arias A, Barros-Garcia D, and Gonzalez-Ortegon E. 2019. New record and revised list of *Megalops atlanticus* (Elopiformes: Megalopidae) from Atlantic European waters. *Cybium* 43:203–207.

Dolganov VN, Kharin VE, and Zemnukhov VV. 2008. The Megalopidae, a new family of fishes for fauna of Russia. *Journal of Ichthyology* 48:275. DOI 10.1134/S0032945208030107.

Roux C. 1960. Note sur le Tarpon [*Megalops atlanticus* C. et V.) des côtes de la République du Congo. *Bulletin du Muséum national d'histoire naturelle* 32:314-319.

Twomey E, and Byrne P. 1985. A new record for the tarpon, *Tarpon atlanticus* Valenciennes (Osteichthyes‐Elopiformes‐Elopidae), in the eastern North Atlantic. *Journal of Fish Biology* 26:359–362.

### Osteoglossiformes

**Mormyridae**

Monarch. 2022. Fish specimens housed at the California Academy of Sciences uploaded to Monarch. *Available at http//:monarch.calacademy.org/index.php* (accessed July 24 2022).

**Osteoglossidae**

Leal MEC, and De Sant-Anna VB. 2006. Quantitative analysis of interspecific and ontogenetic variation in *Osteoglossum* species (Teleostei: Osteoglossiformes: Osteoglossidae). *Zootaxa* 1239:49–68. DOI 10.11646/zootaxa.1239.1.4.

Pouyaud L, Sudarto TG, and Teugels G. 2003. The different colour varieties of the Asian arowana *Scleropages formosus* (Osteoglossidae) are distinct species: morphologic and genetic evidences. *Cybium* 27:287–305.

Scadeng M, McKenzie C, He W, Bartsch H, Dubowitz DJ, Stec D, and St. Leger J. 2020. Morphology of the Amazonian Teleost Genus Arapaima Using Advanced 3D Imaging. *Frontiers in Physiology* 11:260.

Stewart DJ. 2013. A New Species of *Arapaima* (Osteoglossomorpha: Osteoglossidae) from the Solimões River, Amazonas State, Brazil. *Copeia* 2013:470-476. DOI 10.1643/ci-12-017.

## Ostariophysi

### Alepocephaliformes

Fujiwara Y, Kawato M, Poulsen JY, Ida H, Chikaraishi Y, Ohkouchi N, Oguri K, Gotoh S, Ozawa G, Tanaka S, Miya M, Sado T, Kimoto K, Toyofuku T, and Tsuchida S. 2021. Discovery of a colossal slickhead (Alepocephaliformes: Alepocephalidae): an active-swimming top predator in the deep waters of Suruga Bay, Japan. *Scientific Reports* 11:2490. DOI 10.1038/s41598-020-80203-6.

Goode GB, and Bean TH. 1879. Description of *Alepocephalus bairdii*, a new species of fish from the deep sea fauna of the western Atlantic. *Proceedings of the United Stated National Museum* 2:55-57.

### Characiformes

**Acestrorhynchidae**

Einhardt MDS, Corrêa F, Cavalheiro ACM, Piedras S, and Pouey J. 2014. New area of occurence to *Acestrorhynchus pantaneiro* (Menezes, 1992) (Characiformes, Acestrorhynchidae) in the Chasqueiro Stream Basin, Patos-Mirim system, Rio Grande do Sul, Brazil. *Boletín de la Sociedad Zoológica del Uruguay* 23:36-42.

**Alestidae**

Ahmed EY, Hamid MM, Mahmoud ZN, Hagar ESA, and Masri MA. 2020. Meristic, morphological and genetic identification of the fish *Brycinus nurse* (Alestidae) from Sinnar, Blue Nile. *International Journal of Fisheries and Aquatic Studies* 8:373–376.

Brewster B. 1986. A review of the genus *Hydrocynus* Cuvier 1819 (Teleostei, Characiformes). *Bulletin of the British Museum (Natural History)* 50:163-206.

Monarch. 2022. Fish specimens housed at the California Academy of Sciences uploaded to Monarch. *Available at http//:monarch.calacademy.org/index.php* (accessed July 24 2022).

**Anostomidae**

Birindelli JLO, Peixoto LAW, Wosiacki WB, and Britski HA. 2013. New Species of *Hypomasticus* Borodin, 1929 (Characiformes: Anostomidae) from Tributaries of the Lower Rio Amazonas, Brazil. *Copeia* 2013:464-469. DOI 10.1643/ci-12-148.

**Characidae**

Benine RC, Melo BF, Castro RM, and Oliveira C. 2015. Taxonomic revision and molecular phylogeny of *Gymnocorymbus* Eigenmann, 1908 (Teleostei, Characiformes, Characidae). *Zootaxa* 3956:1-28. DOI 10.11646/zootaxa.3956.1.1.

Bertaco VA, and Malabarba LR. 2010. A review of the Cis-Andean species of *Hemibrycon* Günther (Teleostei: Characiformes: Characidae: Stevardiinae), with description of two new species. *Neotropical Ichthyology* 8:737–770. DOI 10.1590/s1679-62252010000400005.

Camelier P, Dagosta FCP, and Marinho MMF. 2018. New remarkable sexually dimorphic miniature species of *Hyphessobrycon* (Characiformes: Characidae) from the upper Rio Tapajos basin. *Journal of Fish Biology* 92:1149-1162. DOI 10.1111/jfb.13579.

dos Santos MR, and Renesto E. 2013. Genetic variability in *Oligosarcus paranensis* (Teleostei: Characiformes) from the São Francisco river, Ivaí river basin – Paraná State, Brazil. *Acta Scientiarum Biological Sciences* 35. DOI 10.4025/actascibiolsci.v35i3.14179.

Fuster de Plaza ML. 1950. Una contribución al conocimiento del dorado (*Salminus maxillosus* Cuv. y Val.). *Revista del Museo de La Plata* 6:172–214.

Lima FCT, and Britski HA. 2007. *Salminus franciscanus*, a new species from the rio São Francisco basin, Brazil (Ostariophysi: Characiformes: Characidae). *Neotropical Ichthyology* 5:237-244. DOI 10.1590/s1679-62252007000300001.

Mattox GMT, and Conway KW. 2021. Osteology of *Tucanoichthys tucano* Géry and Römer, an enigmatic miniature fish from the Amazon basin, Brazil (Teleostei: Characiformes: Characidae). *Vertebrate Zoology* 71:645-667. DOI 10.3897/vz.71.e71886.

Mendonça MB, Peixoto LAW, Dutra GM, and Netto-Ferreira AL. 2016. A new miniature of Xenurobryconini (Characiformes: Characidae) from the rio Tapajós basin, Brazil. *Neotropical Ichthyology* 14. DOI 10.1590/1982-0224-20150057.

Menezes NA. 2007. A new species of *Cynopotamus* Valenciennes, 1849 (Characiformes, Characidae) with a key to the species of the genus. *Zootaxa* 1635:55-61. DOI 10.11646/zootaxa.1635.1.4.

Menezes NA, and Ribeiro AC. 2010. *Oligosarcus jacuiensis* (Characiformes: Characidae), a new species from the Uruguay and Jacuí River basins, southern Brazil. *Neotropical Ichthyology* 8:649-653. DOI 10.1590/s1679-62252010000300010.

Pinho SM, David LH, Garcia F, Keesman KJ, Portella MC, and Goddek S. 2021. South American fish species suitable for aquaponics: a review. *Aquaculture International* 29:1427-1449. DOI 10.1007/s10499-021-00674-w.

**Cynodontidae**

Brewster B. 1986. A review of the genus *Hydrocynus* Cuvier 1819 (Teleostei, Characiformes). *Bulletin of the British Museum (Natural History)* 50:163-206.

Toledo-Piza M, Menezes NA, and Mendes dos Santos G. 1999. Revision of the neotropical fish genus *Hydrolycus* (Ostariophysi: Cynodontinae) with the description of two new species. *Ichthyological Exploration of Freshwaters* 10:255–280.

**Hepsetidae**

Decru E, Vreven E, and Snoeks J. 2012. A revision of the West African *Hepsetus* (Characiformes: Hepsetidae) with a description of *Hepsetus akawo* sp. nov. and a redescription of *Hepsetus odoe* (Bloch, 1794). *Journal of Natural History* 46:1-23. DOI 10.1080/00222933.2011.622055.

**Iguanodectidae**

Silva-Oliveira C, Moreira CR, Lima FCT, and Py-Daniel LR. 2020. The true identity of *Bryconops cyrtogaster* (Norman), and description of a new species of *Bryconops* Kner (Characiformes: Iguanodectidae) from the Rio Jari, lower Amazon basin. *Journal of Fish Biology* 97:860-868. DOI 10.1111/jfb.14445.

**Serrasalmidae**

Andrade MC, Sousa LM, Ota RP, Jégu M, and Giarrizzo T. 2016. Redescription and Geographical Distribution of the Endangered Fish *Ossubtus xinguense* Jégu 1992 (Characiformes, Serrasalmidae) with Comments on Conservation of the Rheophilic Fauna of the Xingu River. *PLoS ONE* 11:e0161398. DOI 10.1371/journal.pone.0161398.

Bonani Mateussi NT, Melo BF, and Oliveira C. 2020. Molecular delimitation and taxonomic revision of the wimple piranha *Catoprion* (Characiformes: Serrasalmidae) with the description of a new species. *Journal of Fish Biology* 97:668-685. DOI 10.1111/jfb.14417.

Deprá GC, Oliveira AG, Silva AB, Frota A, Proença HC, Message HJ, dos Reis RB, and Ota RR. 2021. A New Potential Invader: First Record of the Pirambeba *Serrasalmus geryi* (Characiformes: Serrasalmidae) in the upper Paraná River floodplain, Brazil. *Journal of Ichthyology* 61:190-195. DOI 10.1134/s0032945221020041.

Escobar LM, Ota RP, Machado-Allison A, Andrade-Lopez J, Farias IP, and Hrbek T. 2019. A new species of *Piaractus* (Characiformes: Serrasalmidae) from the Orinoco Basin with a redescription of *Piaractus brachypomus*. *Journal of Fish Biology* 95:411-427. DOI 10.1111/jfb.13990.

Fink WL. 1993. Revision of the Piranha Genus *Pygocentrus* (Teleostei, Characiformes). *Copeia* 1993:665–687. DOI 10.2307/1447228.

Gómez SE, Bentos CA, and Ramirez JL. 2004. Humans attacked by piranhas (Pisces: Serrasalmidae) in Buenos Aires Province, Argentina. *Aqua, Journal of Ichthyology and Aquatic Biology* 9:25–28.

Hilsdorf AWS, Uliano-Silva M, Coutinho LL, Montenegro H, Almeida-Val VMF, and Pinhal D. 2021. Genome assembly and annotation of the tambaqui (*Colossoma macropomum*): an emblematic fish of the Amazon River Basin. *Gigabyte* 2021:1-14. DOI 10.46471/gigabyte.29.

Nakayama CM, Feldberg E, and Bertollo LAC. 2012. Karyotype differentiation and cytotaxonomic considerations in species of Serrasalmidae (Characiformes) from the Amazon basin. *Neotropical Ichthyology* 10:53-58. DOI 10.1590/s1679-62252012000100005.

Ota RP, Machado VN, Andrade MC, Collins RA, Farias IP, and Hrbek T. 2020. Integrative taxonomy reveals a new species of pacu (Characiformes: Serrasalmidae: *Myloplus*) from the Brazilian Amazon. *Neotropical Ichthyology* 18. DOI 10.1590/1982-0224-20190112.

Ota RP, Py-Daniel LHR, and Jégu M. 2016. A new Silver Dollar species of *Metynnis* Cope, 1878 (Characiformes: Serrasalmidae) from Northwestern Brazil and Southern Venezuela. *Neotropical Ichthyology* 14. DOI 10.1590/1982-0224-20160023.

Ruiz-Carus R, and Davis SB. 2003. Register of an exceptionally large redbellied pacu, *Piaractus brachypomus* (Teleostei, Characidae) in east-central Florida, with gonad and diet analyses. *Florida Scientist* 66:184–188.

### Clupeiformes

**Cheirocentridae**

Monarch. 2022. Fish specimens housed at the California Academy of Sciences uploaded to Monarch. *Available at http//:monarch.calacademy.org/index.php* (accessed July 24 2022).

Randall JE. 1997. Randall's tank photos. Collection of 10,000 large-format photos (slides) of dead fishes. *Available at www.fishbase.org (see species pages for more details)* (accessed June 29 2022).

**Clupeidae**

Bañón R, Punzón A, Barros-García D, and De Carlos A. 2019. Range extension of the Atlantic herring *Clupea harengus* (Clupeiformes: Clupeidae) southern part of the Northeast Atlantic Ocean. *Cybium* 43:291–293. DOI 10.26028/cybium/2019-433-011.

Khamees N, Adday T, and Abed J. 2018. Occurrence and Redescription of *Thryssa setirostris* (Broussonet, 1782) (Clupiformes, Engraulidae) from Iraqi Marine Water. *Bulletin of the Iraq Natural History Museum* 15:123-130. DOI 10.26842/binhm.7.2018.15.2.0123.

Kohanestani ZM, Ghorbani R, Yelghi S, Fazel A, and Zoghi M. 2013. An investigation on morphology, age and growth of the Caspian Sea Kilka (*Clupeonella cultriventris*) in Babolsar, southern Caspian Sea *International Journal of Aquatic Biology* 1:143–149.

Randall JE. 1997. Randall's tank photos. Collection of 10,000 large-format photos (slides) of dead fishes. *Available at www.fishbase.org (see species pages for more details)* (accessed June 29 2022).

Reyes RB. 2017. Photos contributed by Rodolfo B. Reyes to Fishbase. *Available at www.fishbase.org (see species pages for more details)* (accessed July 31 2022).

**Dussumieriidae**

Mili S, Ennouri R, and Rafrafi-Nouira S. 2020. Additional record of Golani round herring, *Etrumeus golanii* (Osteichthyes: Dussumieriidae) from Tunisian waters with comments on its distribution in the Mediterranean Sea. *Annales, Series historia naturalis* 30:105-110.

Randall JE, and DiBattista JD. 2012. *Etrumeus makiawa*, a New Species of Round Herring (Clupeidae: Dussumierinae) from the Hawaiian Islands. *Pacific Science* 66:97-110. DOI 10.2984/66.1.6.

**Engraulidae**

Garcia Júnior J, Nóbrega MF, and Oliveira JEL. 2015. Coastal fishes of Rio Grande do Norte, northeastern Brazil, with new records. *Check List* 11. DOI 10.15560/11.3.1659.

Monarch. 2022. Fish specimens housed at the California Academy of Sciences uploaded to Monarch. *Available at http//:monarch.calacademy.org/index.php* (accessed July 24 2022).

Strasburg DW. 1960. A new Hawaiian engraulid fish. *Pacific Science* 14:3953–3999.

### Cypriniformes

**Cyprinidae**

Ali AM, and Tomas NK. 2009. Some ecological aspects of bizz *Barbus esocinus* Heckel, 1843 (Actinopterygii, Cyprinidae) from Tigris and Euphrates rivers - Iraq. *Tropical Freshwater Biology* 18:27–50.

Britz R, Conway KW, and Rüber L. 2014. Miniatures, morphology and molecules: *Paedocypris* and its phylogenetic position (Teleostei, Cypriniformes). *Zoological Journal of the Linnean Society* 172:556-615. DOI 10.1111/zoj.12184.

Brraich OS, and Akhter S. 2015a. Morphometric characters and meristic counts of a Fish, *Crossocheilus latius latius* (Hamilton-Buchanan) from Ranjit Sagar Wetland, India. *International Journal of Fisheries and Aquatic Studies* 2:260-265.

Brraich OS, and Akhter S. 2015b. Morphometric characters and meristic counts of a fish, *Garra gotyla gotyla* (Gray) from Ranjit Sagar Wetland, situated in the Himalayan foothills, India. *International Research Journal of Biological Sciences* 4:66-72.

Hoang HD, Pham HM, Durand JD, Tran NT, and Phan PD. 2015. Mahseers genera *Tor* and *Neolissochilus* (Teleostei: Cyprinidae) from southern Vietnam. *Zootaxa* 4006:551-568. DOI 10.11646/zootaxa.4006.3.8.

Hora SL. 1943. The Game Fishes of India. XVI.-The mahseers or the large-scaled barbels of India. 9. Further observations on mahseers from the Deccan. *Journal of the Bombay Natural History Society* 44:1-8.

Hossain MY, Ohtomi J, and Ahmed ZF. 2009. Morphometric, meristic characteristics and conservation of the threatened fish, *Puntius sarana* (Hamilton, 1822) (Cyprinidae) in the Ganges River, Northwestern Bangladesh. *Turkish Journal of Fisheries and Aquatic Sciences* 9:223-225. DOI 10.4194/trjfas.2009.0215.

Jurajda P, and Pavlov I. 2016. Rediscovery of *Rutilus virgo* in the River Dyje, Czech Republic. *Folia Zoologica* 65:98–100.

Kaeding LR, Burdick BD, Schrader PA, and Noonan WR. 1986. Recent Capture of a Bonytail (*Gila elegans*) and Observations on This Nearly Extinct Cyprinid from the Colorado River. *Copeia* 1986:1021-1023. DOI 10.2307/1445305.

Kottelat M, and Lim KKP. 2021. Two new species of *Barbodes* from the Malay Peninsula and comments on ‘cryptic species’ in the *B. binotatus* group (Teleostei: Cyprinidae). *Raffles Bulletin of Zoology* 69:522-540. DOI 10.26107/RBZ-2021-0069.

Naeem M, Abidi SZA, Khan M, Ishtiaq A, and Naeem Z. 2017. Length–weight, length-length and condition factor relationships of *Labeo gonius* (Hamilton) from Taunsa Barrage, River Indus, Pakistan *International Journal of Fisheries and Aquatic Studies* 5:15-19.

Rehman FU, Rehman HU, Aman S, Aziz S, Shabir H, Majid A, Ullah A, Rehman Safi AU, and Subhan F. 2015. Morphometric and meristic analysis of silver carp (*Hypophthalmichthys molitrix*). *Global Veterinaria* 15:82-92. DOI 10.5829/idosi.gv.2015.15.01.95217.

Shao KT, and Lim PL. 1991. *Fishes of freshwater and estuary. Encyclopedia of field guide in Taiwan*. Taipei: Recreation Press.

Talabishka E, Didenko A, and Velykopolskiy I. 2015. Some biological data on cactus roach, *Rutilus virgo* (Heckel), in rivers of the Transcarpathian region of Ukraine. *Archives of Polish Fisheries* 23:67–77. DOI 10.1515/aopf-2015-0008.

### Gonorynchiformes

**Chanidae**

Gandhi V, Mohanraj G, and Thiagarajan R. 1986. Biology and biometry of milkfish *Chanos chanos* (Forsskal). *Journal of the Marine Biological Association of India* 28:169–171.

**Gonorynchidae**

Roberts CD, and Grande TC. 1997. The sandfish, *Gonorynchus forsteri* (Gonorynchidae), from bathyal depths off New Caledonia, with notes on New Zealand specimens. *Proceedings of the 5th Indo-Pacific Conference, Nouméa*:195–205.

Te Papa Museum. 2022. sandfish, *Gonorynchus forsteri* Ogilby, 1911. *Available at https://collections.tepapa.govt.nz/object/207459* (accessed August 5 2022).

### Gymnotiformes

**Gymnotidae**

de Santana CD, Crampton WGR, Dillman CB, Frederico RG, Sabaj MH, Covain R, Ready J, Zuanon J, de Oliveira RR, Mendes-Júnior RN, Bastos DA, Teixeira TF, Mol J, Ohara W, Castro NCe, Peixoto LA, Nagamachi C, Sousa L, Montag LFA, Ribeiro F, Waddell JC, Piorsky NM, Vari RP, and Wosiacki WB. 2019. Unexpected species diversity in electric eels with a description of the strongest living bioelectricity generator. *Nature Communications* 10:4000. DOI 10.1038/s41467-019-11690-z.

Maxime EL, and Albert JS. 2009. A new species of *Gymnotus* (Gymnotiformes: Gymnotidae) from the Fitzcarrald Arch of southeastern Peru. *Neotropical Ichthyology* 7:579-585. DOI 10.1590/s1679-62252009000400004.

### Siluriformes

Note: for catfishes the data in Schultz (1944) was considered excluding Loricariidae, as measurements for these taxa were reported including the supraoccipital and it is not clear if this measurement corresponds to head length in the same way as it does for other bony fishes.

Schultz LP. 1944. The catfishes of Venezula, with descriptions of thirty-eight new forms. *Proceedings of the United Stated National Museum* 94:173-338.

**Ariidae**

Betancur-R R, and P AA. 2004. Description of *Notarius biffi* n. sp. and redescription of *N. insculptus* (Jordan and Gilbert) (Siluriformes: Ariidae) from the eastern Pacific, with evidence of monophyly and limits of *Notarius*. *Zootaxa* 703. DOI 10.11646/zootaxa.703.1.1.

Betancur-R R, and P AAA. 2005. Description of *Cathorops mapale*, a new species of sea catfish (Siluriformes: Ariidae) from the Colombian Caribbean, based on morphological and mitochondrial evidence. *Zootaxa* 1045. DOI 10.11646/zootaxa.1045.1.4.

De la Cruz-Agüero J, Cota-Gómez VM, and Nieto-Navarro JT. 2010. New maximum size record for the Chili sea catfish *Notarius troschelii* (Siluriformes: Ariidae) from the tropical eastern Pacific. *Marine Biodiversity Records* 3. DOI 10.1017/s1755267210000916.

Garcia Júnior J, Nóbrega MF, and Oliveira JEL. 2015. Coastal fishes of Rio Grande do Norte, northeastern Brazil, with new records. *Check List* 11. DOI 10.15560/11.3.1659.

**Auchenipteridae**

Schultz LP. 1944. The catfishes of Venezula, with descriptions of thirty-eight new forms. *Proceedings of the United Stated National Museum* 94:173-338.

**Bagridae**

Amin MR, Mollah MF, Taslima K, and Muhammadullah. 2014. Morphological observation and length-weight relationship of critically endangered riverine catfish *Rita rita* (Hamilton). *Pakistan Journal of Biological Sciences* 17:234-240. DOI 10.3923/pjbs.2014.234.240.

Nair SM, Kumari K, Kumar AP, Raghavan R, and Jaiswar AK. 2021. The identity and distribution of striped bagrid catfish, *Mystus tengara* (Hamilton 1822) revealed through integrative taxonomy. *Molecular Biology Reports*. DOI 10.1007/s11033-021-06880-2.

Priyanka C, Tewari G, Datta SN, and Kumar B. T. N. 2020. Morphometric and meristic variations in different fish species of family Bagridae from Harike wetland. *Journal of Entomology and Zoology Studies* 8:1788-1793.

Saini A, Dua A, and Mohindra V. 2008. Comparative morphometrics of two populations of giant river catfish (*Mystus seenghala*) from the Indus river system. *Integrative Zoology* 3:219–226. DOI 10.1111/j.1749-4877.2008.00099.x.

Vo LTT, Tran AN, Phan TQ, and Dinh QM. 2021. Morphometrics variations of *Mystus mysticetus* Roberts, 1992 in the Mekong Delta, Vietnam. *AACL Bioflux* 14:3423–3431.

**Heptapteridae**

Benitez MF, Terán GE, Alonso F, Aguilera G, and Mirande JM. 2017. *Cetopsorhamdia iheringi* (Siluriformes, Heptapteridae): a new record for the freshwater ichthyofauna of Argentina. *Revista del Museo Argentino de Ciencias Naturales* 19:113-119.

Kütter MT, Bemvenuti MDA, and Moresco A. 2009. Feeding strategy of the jundiá *Rhamdia quelen* (Siluriformes, Heptapteridae) in costal lagoons of southern Brazil. *Acta Scientiarum Biological Sciences* 31. DOI 10.4025/actascibiolsci.v31i1.335.

Pinho SM, David LH, Garcia F, Keesman KJ, Portella MC, and Goddek S. 2021. South American fish species suitable for aquaponics: a review. *Aquaculture International* 29:1427-1449. DOI 10.1007/s10499-021-00674-w.

**Ictaluridae**

Bean TH. 1880. Description of a new species of *Amiurus* (*A. ponderosus*) from the Mississippi River. *Proceedings of the United Stated National Museum* 2:286–290.

**Pangasiidae**

Ajith Kumar TT, Kumar S, and K. Lal K. 2020. Length-weight relationship of a newly described catfish *Pangasius silasi* Dwivedi et al., 2017 from Nagarjuna Sagar Dam, Telengana, India. *Indian Journal of Fisheries* 67. DOI 10.21077/ijf.2019.67.2.88546-19.

Ayyathurai KPV, Kodeeswaran P, Mohindra V, Singh RK, Ravi C, Kumar R, Valaparambil B, Thipramalai Thangappan AK, Jena J, and Lal KK. 2022. Description of a new *Pangasius* (Valenciennes, 1840) species, from the Cauvery River extends distribution range of the genus up to South Western Ghats in peninsular India. *PeerJ* 10. DOI 10.7717/peerj.14258.

Dwivedi AK, Gupta BK, Singh RK, Mohindra V, Chandra S, Easawarn S, Jena J, and Lal KK. 2017. Cryptic diversity in the Indian clade of the catfish family Pangasiidae resolved by the description of a new species. *Hydrobiologia* 797:351-370. DOI 10.1007/s10750-017-3198-z.

Gustiano R, Teugels GG, and Pouyaud L. 2003. Revision of the *Pangasius kunyit* catfish complex, with description of two new species from South-East Asia (Siluriformes; Pangasiidae). *Journal of Natural History* 37:357-376. DOI 10.1080/713834687.

Pouyaud L, and Teugels GG. 2000. Description of a new pangasiid catfish from East Kalimantan, Indonesia (Siluriformes, Pangasiidae). *Ichthyological Exploration of Freshwaters* 11:193-200.

**Pimelodidae**

Buitrago–Suárez UA, and Burr BM. 2007. Taxonomy of the catfish genus *Pseudoplatystoma* Bleeker (Siluriformes: Pimelodidae) with recognition of eight species. *Zootaxa* 1512:1-38. DOI 10.11646/zootaxa.1512.1.1.

Garavello JC. 2005. Revision of genus *Steindachneridion* (Siluriformes: Pimelodidae). *Neotropical Ichthyology* 3:607-623. DOI 10.1590/s1679-62252005000400018.

Lundberg JG. 2005. *Brachyplatystoma promagdalena*, new species, a fossil goliath catfish (Siluriformes: Pimelodidae) from the Miocene of Colombia, South America. *Neotropical Ichthyology* 3:597–605. DOI http://dx.doi.org/10.1590/S1679-62252005000400017.

Lundberg JG, Mago-Leccia F, and Nass P. 1991. *Exallodontus anguanai*, a new genus and species of Pimelodidae (Pisces: Siluriformes) from deep river channels of South America, and delimitation of the subfamily Pimelodinae. *Proceedings of the Biological Society of Washington* 104:840=869.

Masdeu M, Mello FT-d, Loureiro M, and Arim M. 2011. Feeding habits and morphometry of *Iheringichthys labrosus* (Lütken, 1874) in the Uruguay River (Uruguay). *Neotropical Ichthyology* 9:657-664. DOI 10.1590/s1679-62252011005000034.

Schultz LP. 1944. The catfishes of Venezula, with descriptions of thirty-eight new forms. *Proceedings of the United Stated National Museum* 94:173-338.

Stewart DJ. 1986. Revision of *Pimelodina* and Description of a New Genus and Species from the Peruvian Amazon (Pisces: Pimelodidae). *Copeia* 1986:653–672. DOI 10.2307/1444947.

**Plotosidae**

Ali M, Saad A, and Soliman A. 2015. Expansion Confirmation of the Indo-Pacific Catfish, *Plotosus lineatus* (Thunberg, 1787), (Siluriformes: Plotosidae) into Syrian Marine Waters. *American Journal of Biology and Life Sciences* 3:7-11.

**Sisoridae**

Dahanukar N, Diwekar M, and Paingankar M. 2011. Rediscovery of the threatened Western Ghats endemic sisorid catfish *Glyptothorax poonaensis* (Teleostei: Siluriformes: Sisoridae). *Journal of Threatened Taxa* 3:1885–1898.

Dey S, Manorama M, and Ramanujam SN. 2015. New records of three species of fish in the upper reaches of the Brahmaputra and Surma-Meghna river basins, Meghalaya, India. *Journal of Threatened Taxa* 7:7922–7926. DOI 10.11609/JoTT.o3297.7922-6.

## Stem Euteleostei

### Aulopiformes

**Alepisauridae**

Bean TH. 1883. Description of a new species of *Alepidosaurus* (*A. aesculapius*) from Alaska. *Proceedings of the United Stated National Museum* 5:661-663.

Gill T. 1862. Description of new species of Alepidosauroidae. *Proceedings of the Academy of Natural Sciences of Philadelphia* 14:127-132.

**Paralepididae**

Mytilineou C, Anastasopoulou A, Christides G, Bekas P, Smith CJ, Papadopoulou KN, Lefkaditou E, and Kavadas S. 2013. New records of rare deep-water fish species in the Eastern Ionian Sea (Mediterranean Sea). *Journal of Natural History* 47:1645-1662. DOI 10.1080/00222933.2013.775372.

**Synodontidae**

Bogorodsky SV, Alpermann TJ, Mal AO, and Gabr MH. 2014. Survey of demersal fishes from southern Saudi Arabia, with five new records for the Red Sea. *Zootaxa* 3852:401-437. DOI 10.11646/zootaxa.3852.4.1.

Chhandaprajnadarsini EM, Roul SK, Swain S, Jaiswar AK, Shenoy L, and Chakraborty SK. 2018. Biometric analysis of brushtooth lizard fish *Saurida undosquamis* (Richardson, 1848) from Mumbai waters. *Journal of Entomology and Zoology Studies* 6:1165-1171.

Russell BC, Golani D, and Tikochinski Y. 2015. *Saurida lessepsianus* a new species of lizardfish (Pisces: Synodontidae) from the Red Sea and Mediterranean Sea, with a key to Saurida species in the Red Sea. *Zootaxa* 3956:559-568. DOI 10.11646/zootaxa.3956.4.7.

### Esociformes

**Esocidae**

Casselman JM, Crossman EJ, Ihssen PE, Reist JD, and Booke HE. 1986. Identification of muskellunge, northern pike, and their hybrids. *American Fisheries Society Special Publication* 15:14–46.

Denys GP, Dettai A, Persat H, Hautecoeur M, and Keith P. 2014. Morphological and molecular evidence of three species of pikes *Esox* spp. (Actinopterygii, Esocidae) in France, including the description of a new species. *Comptes Rendus Biologies* 337:521-534. DOI 10.1016/j.crvi.2014.07.002.

### Gadiformes

**Bregmacerotidae**

Bogorodsky SV, Alpermann TJ, Mal AO, and Gabr MH. 2014. Survey of demersal fishes from southern Saudi Arabia, with five new records for the Red Sea. *Zootaxa* 3852:401-437. DOI 10.11646/zootaxa.3852.4.1.

**Gadidae**

Privalikhin AM, and Norvillo GV. 2010. On the Finding of a Rare Species—Norwegian Pollock *Theragra finnmarchica* Koefoed, 1956 (Gadidae)—in the Barents Sea. *Journal of Ichthyology* 50:143–147.

**Lotidae**

Pais A, Merella P, Follesa MC, Garippa G, and Golani D. 2008. New data on *Gaidropsarus granti* (Regan, 1903) (Gadiformes: Lotidae) from the Mediterranean Sea, with emphasis on its parasites. *Scientia Marina* 72:461–468.

**Moridae**

Bilecenoglu M, Bogaç Kunt K, and Taşkavak E. 2002. First record of *Mora moro* (Risso, 1810) (Pisces, Moridae) from the eastern Mediterranean Sea. *Israel Journal of Zoology* 48:243-257.

Diatta Y, Rafrafi-Nouira S, Diaby A, Reynaud C, and Capapé C. 2021. First substantial records of *Physiculus dalwigki* (Moridae) from the coast of Senegal (eastern tropical Atlantic). *Thalassia Salientia* 43:3-8. DOI 10.1285/i15910725v43p3.

### Galaxiiformes

**Galaxiidae**

Te Papa Museum. 2022. Eldon's galaxias, *Galaxias eldoni* McDowall, 1997. *Available at https://collections.tepapa.govt.nz/object/208192* (accessed August 5 2022).

### Lampriformes

Sulić Šprem J, Dobroslavić T, Kožul V, Prusina I, Onofri V, and Antolović N. 2014. New record of *Lophotus lacepede* Giorna, 1809 and *Lampris guttatus* (Brünnich, 1788) in the southeastern Adriatic Sea (Croatian coast). *Cahiers de Biologie Marine* 55:371–373.

**Lampridae**

Gudger EW. 1930. The Opah or Moonfish, *Lampris luna*, on the Eastern Coast of North America. *The American Naturalist* 64:168-178.

Gudger EW. 1931. The Opah or Moonfish, Lampris luna, on the Coasts of California and of Hawaii. *The American Naturalist* 65:531-540.

Sulić Šprem J, Dobroslavić T, Kožul V, Prusina I, Onofri V, and Antolović N. 2014. New record of *Lophotus lacepede* Giorna, 1809 and *Lampris guttatus* (Brünnich, 1788) in the southeastern Adriatic Sea (Croatian coast). *Cahiers de Biologie Marine* 55:371–373.

Underkoffler KE, Luers MA, Hyde JR, and Craig MT. 2018. A taxonomic review of *Lampris guttatus* (Brunnich 1788) Lampridiformes; Lampridae) with descriptions of three new species. *Zootaxa* 4413:551–565. DOI 10.11646/zootaxa.4413.3.9.

**Lophotidae**

Bachouche S, Etsouri M, and Rouidi S. 2016. The first record of Crested Oarfish, *Lophotus lacepede* (Actinopterygii: Lampriformes) from the marine waters of Algeria. *International Journal of Science and Knowledge* 5:19–22.

Dulčić J, and Soldo A. 2008. New finding of crested oarfish *Lophotus lacepede* (Lophotidae), in the Adriatic Sea. *Cybium* 32:93–94.

Falsone F, Geraci ML, Scannella D, Okpala COR, Giusto GB, Bosch-Belmar M, Gancitano S, and Bono G. 2017. Occurrence of two rare species from order Lampriformes: Crestfish *Lophotus lacepede* (Giorna, 1809) and scalloped ribbonfish *Zu cristatus* (Bonelli, 1819) in the northern coast of Sicily, Italy. *Acta Adriatica: International Journal of Marine Sciences* 58:137–146.

Sulić Šprem J, Dobroslavić T, Kožul V, Prusina I, Onofri V, and Antolović N. 2014. New record of *Lophotus lacepede* Giorna, 1809 and *Lampris guttatus* (Brünnich, 1788) in the southeastern Adriatic Sea (Croatian coast). *Cahiers de Biologie Marine* 55:371–373.

**Regalecidae**

Lee HW, Yang JH, Sohn MH, Lee JB, Chun YY, Hwang KS, and Lee DW. 2013. Occurrence of *Architeuthis* sp. and *Regalecus russellii* in the East Sea, Korea. *Korean Journal of Fisheries and Aquatic Sciences* 46:856–861.

Ruiz AE, and Gosztonyi AE. 2010. Records of regalecid fishes in Argentine waters. *Zootaxa* 2509. DOI 10.11646/zootaxa.2509.1.5.

**Trachipteridae**

Falsone F, Geraci ML, Scannella D, Okpala COR, Giusto GB, Bosch-Belmar M, Gancitano S, and Bono G. 2017. Occurrence of two rare species from order Lampriformes: Crestfish *Lophotus lacepede* (Giorna, 1809) and scalloped ribbonfish *Zu cristatus* (Bonelli, 1819) in the northern coast of Sicily, Italy. *Acta Adriatica: International Journal of Marine Sciences* 58:137–146.

### Salmoniformes

Holčík J, Hensel K, Nieslanik J, and Skácel L. 1988. *The Eurasian Huchen, Hucho hucho*.

Holčík J, and Stefanov T. 2008. Taxonomic status of salmonids in the Bulgarian stretch of the Danube River and their bionomic strategy. *Journal of Applied Ichthyology* 24:605-609. DOI 10.1111/j.1439-0426.2008.01082.x.

Pavlov SD, Pivovarov EA, and Ostberg CO. 2012. Dwarf char, a new form of chars (the genus *Salvelinus*) in Lake Kronotskoe. *Doklady Biological Science* 442:282-285. DOI 10.1134/S0012496612010048.

Rosenfield JA. 1998. Detection of Natural Hybridization between Pink Salmon (Oncorhynchus gorbuscha) and Chinook Salmon (Oncorhynchus tshawytscha) in the Laurentian Great Lakes Using Meristic, Morphological, and Color Evidence. *Copeia* 1998:706–714. DOI 10.2307/1447801.

Schwartz FJ. 2007. Morphological and meristic features of pure and hybrid North American trout (family Salmonidae). *Journal of the North Carolina Academy of Science* 123:242–251.

Te Papa Museum. 2022. Chinook salmon, Oncorhynchus tshawytscha (Walbaum, 1792). *Available at https://collections.tepapa.govt.nz/object/840404* (accessed August 5 2022).

### Stomiiformes

**Gonostomatidae**

Ayas D, Akbora HD, and Ergüden D. 2020. Maximum length report of *Gonostoma denudatum* Rafinesque, 1810 in the Eastern Mediterranean Sea. *Marine Science and Technology Bulletin* 9:83-86. DOI 10.33714/masteb.690826.

**Stomiidae**

Rafrafi-Nouira S, Souissi JB, and Capapé C. 2018. About teleost species from deep marine Tunisian waters: with additional records of Sloane's viperfish *Chauliodus sloani* and confirmed occurence of blackfin sorcerer *Nettastoma melanurum*. *Annales, Series historia naturalis* 28:51–58. DOI 10.19233/ASHN.2018.08.

### Zeiformes

Fernández AM, Lloris D, Pérez Gil JL, and Esteban A. 2012. On the occurrence of *Zenopsis conchifer* (Lowe, 1852) (Osteichthyes, Zeidae) in the Mediterranean Sea. *Arxius de Miscel·lània Zoològica* 10:50-54.

Ragonese S, and Giusto GB. 2007. *Zenopsis conchifera* (Lowe, 1852) (Pisces, Actinopterygii, Zeidae): a new alien fish in the Mediterranean Sea. *Journal of Fish Biology* 71:1853-1857. DOI 10.1111/j.1095-8649.2007.01636.x.

## Acanthopterygii

### Acanthuriformes

**Acanthuridae**

Randall JE. 1997. Randall's tank photos. Collection of 10,000 large-format photos (slides) of dead fishes. *Available at www.fishbase.org (see species pages for more details)* (accessed June 29 2022).

**Luvaridae**

Gregory WK, and Conrad GM. 1943. The osteology of *Luvarus imperialis*, a scombroid fish: a study in adaptive evolution. *Bulletin of the American Museum of Natural History* 81:225-283.

Gül B, and Ataç E. 2020. The rare and little-known luvar, *Luvarus imperialis* Rafinesque, 1810, from Edremit Bay (Aegean coast of Turkey). *Mediterranean Marine Science* 21:351-352.

Nichols JT, and Helmuth WT, III. 1940. A Long Island *Luvarus imperialis* Rafinesque. *American Museum Novitates* 1085:1-2.

### Acropomatiformes

Jawad LA, and Koeda K. 2013. Records of *Pempheris schwenkii* Bleeker (1855) and *Pempheris mangula* Cuvier (1829) from the Omani waters. *Journal of Applied Ichthyology* 29:1378–1379. DOI 10.1111/jai.12240.

Randall JE, and Bineesh KK. 2014. Review of the fishes of the genus *Pempheris* (Perciformes: Pempheridae) of India, with description of a new species and a neotype for *P. mangula* Cuvier. *Journal of the Ocean Science Foundation* 10:20-40.

**Epigoniidae**

Catarino D, Stefanni S, Porteiro FM, Rosa A, and Giacomello E. 2021. First record of the pencil cardinal *Epigonus denticulatus* (Perciformes: Epigonidae) in the Azores archipelago. *Journal of Fish Biology* 99:253-257. DOI 10.1111/jfb.14689.

Ergüden D, Bayhan YK, and Altun A. 2017. First record of *Epigonus denticulatus* Dieuzeide, 1950, (Osteichthyes: Epigonidae), from Mersin Bay, Turkey. *Cahiers de Biologie Marine* 58:359-362. DOI 10.21411/CBM.A.23961BB3.

Rafrafi-Nouira S, Golani D, and Capapé C. 2019. First substantiate record of *Epigonus telescopus* (Osteichthyes: Epigonidae) from the Tunisian coast (Central Mediterranean Sea). *Cahiers de Biologie Marine* 60:469-471. DOI 10.21411/CBM.A.BA22F39.

Ruiz-Pico S, Arronte JC, Punzón A, Serrano A, Velasco F, and Fernández-Zapico O. 2012. First records of *Epigonus denticulatus* (Perciformes: Epigonidae) in the northern waters of Spain: northernmost occurrences in the eastern North Atlantic. *Cybium* 36:578-580. DOI 10.26028/cybium/2012-364-011.

### Anabantiformes

**Channidae**

Kashyap A, Awasthi M, and Serajuddin M. 2014. Geographic Morphometric Variations of Freshwater Murrel, *Channa punctatus* from Northern and Eastern Parts of India. *Proceedings of the National Academy of Sciences, India Section B: Biological Sciences* 86:367–373. DOI 10.1007/s40011-014-0451-3.

Kumar R, Jaiswar AK, Sharma R, and Prasad L. 2020. Quantification of morphological variations among populations of *Channa gachua* (Hamilton, 1822) from different geographical locations in India. *Indian Journal of Fisheries* 67. DOI 10.21077/ijf.2019.67.2.93891-16.

Vodonnou DSJV, Kpogue DS, Akpo Y, Aknika M, and Amplifier ED. 2017. Determination of sexual dimorphism of snakehead (*Parachanna obscura*): morphometric and meristic parameters, weight-length relationship and condition factor. *International Journal of Biology, Chemistry, and Science* 11:1742-1752.

Yen DT, Duyen VN, Hien TTT, Pomeroy R, and Hillary E. 2019. Variation in morphometric characteristics between cultured and wild striped snakehead (*Channa striata*) populations in the Mekong Delta. *Can Tho University Journal of Science* 11:70–77. DOI 10.22144/ctu.jen.2019.010.

**Nandidae**

Chakrabarty P, Oldfield RG, and Ng HH. 2006. *Nandus prolixus*, a new species of leaf fish from northeastern Borneo (Teleostei: Perciformes: Nandidae). *Zootaxa* 1328. DOI 10.11646/zootaxa.1328.1.4.

Hasan Mredul MM, Alam MR, Akkas AB, Sharmin S, Pattadar SN, and Ali ML. 2021. Some reproductive and biometric features of the endangered Gangetic Leaf Fish, *Nandus nandus* (Hamilton, 1822): Implication to the baor fisheries management in Bangladesh. *Aquaculture and Fisheries* 6:634-641. DOI https://doi.org/10.1016/j.aaf.2020.10.007.

### Atheriniformes

Goode GB, and Bean TH. 1879. Catalogue of a collection of fishes sent from Pensacola, Florida, and vicinity, by Mr. Silas Stearns, with descriptions of six new species. *Proceedings of the United Stated National Museum* 2:121–156.

Kimura S, Iwatsuki Y, and Yoshino T. 2002. A new silverside, *Atherinomorus aetholepis* sp. nov., from the West Pacific (Atheriniformes: Atherinidae). *Ichthyological Research* 49:240-244.

Mazlan AG, Chung YS, Zaidi CC, Samat A, Arshad A, Seah YG, Alam GM, and Simon KD. 2012. Meristic, morphometrics and length-weight relationship of tropical silverside, *Atherinomorus duodecimalis* (Valenciennes in Cuvier and Valenciennes, 1835) in Seagrass and Mangrove Habitats of Tinggi Island, Johor, Malaysia. *Asian Journal of Animal and Veterinary Advances* 7:921-927. DOI 10.3923/ajava.2012.921927.

Motomura H, and Harazaki S. 2017. Annotated checklist of marine and freshwater fishes of Yaku-shima Island in the Osumi Island, Kagoshima, southern Japan, with 129 new records. *Bulletin of the Kagoshima University Museum* 9:1-183.

Randall JE. 1997. Randall's tank photos. Collection of 10,000 large-format photos (slides) of dead fishes. *Available at www.fishbase.org (see species pages for more details)* (accessed June 29 2022).

### Beloniformes

**Adrianichthyidae**

Mandagi IF, Mokodongan DF, Tanaka R, and Yamahira K. 2018. A New Riverine Ricefish of the Genus *Oryzias* (Beloniformes, Adrianichthyidae) from Malili, Central Sulawesi, Indonesia. *Copeia* 106:297-304. DOI 10.1643/ci-17-704.

**Belonidae**

Acarli D, Kale S, and Çakir K. 2018. A new maximum length for the garfish, *Belone belone* (Linnaeus 1761) in the coast of Gökçeada Island (Aegean Sea, Turkey). *Cahiers de Biologie Marine* 59:385–389. DOI 10.21411/cbm.a.55A28635.

Alshawy F, Ibrahim A, Hussein C, and Lahlah M. 2019. First record of the flat needlefish *Ablennes hians* (Valenciennes, 1846) from Syrian marine waters (eastern Mediterranean). *Marine Biodiversity Records* 12:15. DOI 10.1186/s41200-019-0174-5.

Deidun A, Zava B, Corsini-Foka M, Galdies J, Di Natale A, and Collete BB. 2021. First record of the flat needlefish, *Ablennes hians* (Belonidae) in central Mediterranean waters (Western Ionian Sea). *Annales, Series historia naturalis* 31:9-16. DOI 10.19233/ASHN.2021.02.

Dulçic J, and Soldo A. 2006. A new maximum length for the garpike *Belone belone* (Belonidae). *Cybium* 30:382.

Michailidis N. 2020. First confirmed record of the agujon needlefish *Tylosurus imperialis* (Rafinesque, 1810) from Cyprus. *Mediterranean Marine Science* 21:355-356.

Randall JE. 1997. Randall's tank photos. Collection of 10,000 large-format photos (slides) of dead fishes. *Available at www.fishbase.org (see species pages for more details)* (accessed June 29 2022).

Roul SK, Abdussamad EM, Rohit P, and Jaiswar AK. 2018a. New Distributional Record of Flat Needlefish *Ablennes hians* (Valenciennes, 1846) (Beloniformes: Belonidae) in the North-Eastern Indian Ocean with Taxonomic Details. *Thalassas: An International Journal of Marine Sciences* 35:43-47. DOI 10.1007/s41208-018-0082-1.

Roul SK, Retheesh TB, Ganga U, Abdussamad EM, Rohit P, and Jaiswar AK. 2018b. Length-weight relationships of five needlefish species from Kerala waters, south-west coast of India. *Journal of Applied Ichthyology* 34:190-192. DOI 10.1111/jai.13527.

**Exocoetidae**

Ben Souissi J, Golani D, Mejri H, and Capapé C. 2007. On the occurrence of *Cheilopogon furcatus* in the Mediterranean Sea. *Journal of Fish Biology* 67:1144-1149. DOI 10.1111/j.1095-8649.2005.00790.x.

Falautano M, Perzia P, and Castriota L. 2020. First record of the Lessepsian fish *Parexocoetus mento* in Italian waters and GIS-based spatial and temporal distribution in Mediterranean Sea. *Journal of the Marine Biological Association of the United Kingdom* 100:1163-1169. DOI 10.1017/s002531542000096x.

Randall JE. 1997. Randall's tank photos. Collection of 10,000 large-format photos (slides) of dead fishes. *Available at www.fishbase.org (see species pages for more details)* (accessed June 29 2022).

**Hemirhamphidae**

Rafrafi-Nouira S, Boumaïza M, Reynaud C, and Capape C. 2012. Additional records of Lessepian teleost species off the Tunisian coast (central Mediterranean). *Annales, Series historia naturalis* 22:55-62.

### Beryciformes

Hagiwara K, and Motomura H. 2019. First Japanese record of *Sargocentron iota* (Beryciformes: Holocentridae) from Kakeroma Island, Amami Islands, Japan. *Japanese Journal of Ichthyology* 66:1-5. DOI 10.11369/jji.18–035.

### Blenniifomes

**Bleniidae**

Randall JE. 1997. Randall's tank photos. Collection of 10,000 large-format photos (slides) of dead fishes. *Available at www.fishbase.org (see species pages for more details)* (accessed June 29 2022).

### Callionymiformes

Michailidis N, and Chartosia N. 2016. New record of the Seychelles dragonet *Synchiropus sechellensis* Regan, 1908 from the Mediterranean: accidental entrance or Lessepsian immigration? *BioInvasions Records* 5:291–294. DOI 10.3391/bir.2016.5.4.15.

### Carangiformes

**Carangidae**

Akyol O, and Çoker T. 2019. Maximum Size of Female Alexandria pompano *Alectis alexandrina* (Carangidae) in the Aegean Sea. *Comu Journal of Marine Science and Fisheries* 2:142–146.

Ali-Basha N, Saad A, Hamwi N, and Tufahha A. 2021. First record of pilotfish *Naucrates ductor* (Linnaeus 1758), Carangidae, in the syrian marine waters (Levantine Basin). *Marine Biodiversity Records* 14:7. DOI 10.1186/s41200-021-00202-y.

Andaloro F, Falautano M, Sinopoli M, Passarelli FM, Pipitone C, Addis P, Cau A, and Castriota L. 2005. The lesser amberjack *Seriola fasciata* (Perciformes: Carangidae) in the Mediterranean: A recent colonist? *Cybium* 29:141-145.

Bogorodsky SV, Alpermann TJ, Mal AO, and Gabr MH. 2014. Survey of demersal fishes from southern Saudi Arabia, with five new records for the Red Sea. *Zootaxa* 3852:401-437. DOI 10.11646/zootaxa.3852.4.1.

Capapé C, Rafrafi-Nouira S, Diatta Y, and Golani D. 2018. On the Mediterranean occurrence of Guinean amberjack *Seriola carpenteri* (Osteichthyes: Carangidae), with first confirmed record from the Tunisian coast. *Cahiers de Biologie Marine* 59:399–402. DOI 10.21411/CBM.A.FAC33F0D.

Ergüden D, and Ayas D. 2021. The confirmed occurrence of two specimens of *Remora remora* (Linnaeus, 1758) from Mersin Bay (NE Mediterranean, Turkey). *Aquatic Research* 4:293–298. DOI 10.3153/ar21023.

Hata H, Haraguchi Y, and Motomura H. 2015. First record of *Caranx lugubris* (Perciformes: Carangidae) from the Tokara Islands, Kagoshima Prefecture, southern Japan. *Nature of Kagoshima* 41:69-72.

Kim MJ, Kim JS, and Song CB. 2018. First Record of the Doublespotted Queenfish, *Scomberoides lysan* (Perciformes: Carangidae) from Korea. *Korean Journal of Ichthyology* 30:242-246.

Lin P-L, and Shao K-T. 1999. A review of the carangid fishes (family Carangidae) from Taiwan with description of four new records. *Zoological Studies* 38:33-68.

Motomura H, Iwatsuki Y, Yoshino T, Kimura S, and Osamu I. 1998. A record of a carangid fish, *Scomberoides commersonianus*, from Japan (Perciformes, Carangidae). *Japanese Journal of Ichthyology* 45:101-105.

Myoung SH, Myoung J-G, and Kim J-K. 2015. New Records of *Remora brachyptera* and *R. osteochir* (Perciformes: Echeneidae) from Korea. *Animal Systematics, Evolution and Diversity* 31:101-106. DOI 10.5635/ased.2015.31.2.101.

Nichols J. 1920. *Hynnis* and *Alectis* in the American Museum of Natural History. *Bulletin of the American Museum of Natural History* 42:285-292.

Osmany HB. pers. comm. Photos uploaded to Fishbase.org by H. B. Osmany. *Available at www.fishbase.org (see species pages for more details)* (accessed July 27 2022).

Randall JE. 1997. Randall's tank photos. Collection of 10,000 large-format photos (slides) of dead fishes. *Available at www.fishbase.org (see species pages for more details)* (accessed June 29 2022).

Ray D, Mishra SS, Mohapatra A, and Ghorai N. 2021. Notes on Carangids (Carangiformes: Carangidae) from West Bengal Coast with new records. *Records of the Zoological Survey of India* 121:511-520. DOI 10.26515/rzsi/v121/i4/2021/154542.

Saha T, Datta SK, Zhilik AA, Chowdhury NZ, Baki MA, and Ahmed MS. 2020. New Geographical Record of the Rainbow Runner, *Elagatis bipinnulata* (Quoy & Gaimard, 1825) (Perciformes: Carangidae) from the Bay of Bengal, Bangladesh. *Thalassas: An International Journal of Marine Sciences* 37:23–26. DOI 10.1007/s41208-020-00264-2.

Sajana N, and Bijoy Nandan S. 2017. Morphometry, length-weight relationship and relative condition factor of shrimp scad *Alepes djedaba* (Forsskal, 1775) off Cochin coast, Kerala. *Journal of Aquatic Biology and Fisheries* 5:160-167.

Sajina M, Chakraborty SK, Jaiswar AK, and Sudheesan D. 2013. Morphometric and meristic analysis of horse mackerel, *Megalaspis cordyla* (Linnaeus, 1758) populations along the Indian coast. *Indian Journal of Fisheries* 60:27–34.

Saker Y, Jaiswar AK, Chakraborty SK, and Swamy RP. 2004. Morphometry and length-weight relationship of *Megalaspis cordyla* (Linnaeus, 1758) from Mumbai coast. *Indian Journal of Fisheries* 51:481–486.

Whitley GP. 1947. New sharks and fishes from Western Australia. *The Australian Zoologist* 11:129-150.

**Coryphaenidae**

Akash M, Rahman R, Jahan R, and Naser MN. 2020. On the rare occurrence of the pompano Dolphinfish *Coryphaena equiselis* Linnaeus, 1758 (carangiformes: coryphaenidae) in maritime Bangladesh waters. *Species* 21:227-231.

Artüz ML, and Kubanç N. 2015. First record of *Coryphaena hippurus* (Linnaeus, 1758) from the Sea of Marmara. *Thalassas* 31:9–13.

Jawad LA, Mutlak FM, Al-Faisal AJ, and Waryani B. 2021. The common dolphinfish *Coryphaena hippurus* Linnaeus, 1758 (Perciformes, Coryphaenidae) in the marine waters of Iraq. *Thalassia Salientia* 43:23-28. DOI 10.1285/i15910725v43p23.

Nerlović V, Mravinac B, and Devescovi M. 2015. Additional information on the blue runner, *Caranx crysos* (Mitchill, 1815), from the northern Adriatic Sea:meristic and molecular characterizations. *Acta Adriatica: International Journal of Marine Sciences* 56:309–318.

**Echeneidae**

Tuncer S, Orlov AM, and Ozen O. 2012. First record of marlin sucker, *Remora osteochir* (Cuvier, 1829), from the northeastern Aegean Sea, Turkey. *Journal of Ichthyology* 52:400–408. DOI 10.1134/s0032945212040145.

### Carangaria Incertae Sedis

**Latidae**

Kimura Y, Yamanaka T, and Matsui S. 2019. Second record of Japanese snook *Lates japonicus* from Osaka Bay, Japan. *Japanese Journal of Ichthyology* 66:109-111. DOI 10.11369/jji.18-045.

Randall JE. 1997. Randall's tank photos. Collection of 10,000 large-format photos (slides) of dead fishes. *Available at www.fishbase.org (see species pages for more details)* (accessed June 29 2022).

**Sphyraenidae**

Akyol O. 2015. Additional record of the yellowmouth barracuda, *Sphyraena viridensis* Cuvier, 1829 (Perciformes: Sphyraenidae) from the NE Aegean Sea (Izmir Bay, Turkey). *Journal of Applied Ichthyology* 31:919-921. DOI 10.1111/jai.12832.

Ayo-Olalusi CI, and Ayoade AA. 2017. Biometric Characteristics of *Sphyraena afra* from Coastal Waters of Lagos State, Nigeria. *Journal of Biology, Agriculture, and Healthcare* 7.

Béarez P. 2008. Occurrence of *Sphyraena qenie* (Sphyraenidae) in the tropical eastern Pacific, with a key to the species of barracudas occurring in the area. *Cybium* 32:95–96.

Dulčić J, Kovačić M, and Dragičević B. 2009. Range extension and additional records of the yellowmouth barracuda, *Sphyraena viridensis* (Actinopterygii: Perciformes: Sphyraenidae) in the eastern Adriatic Sea. *Acta Ichthyologica et Piscatoria* 39:59–61. DOI 10.3750/aip2009.39.1.12.

Dulčić J, and Soldo A. 2004. On the occurence of the yellowmouth barracuda, *Sphyraena viridensis* Cuvier 1829 (Pisces: Sphyraenidae), in the Adriatic sea. *Annales, Series historia naturalis* 14:225–228.

González-Acosta AF, Findley LT, Ruiz-Campos G, Burnes-Romo LA, and Espinosa Pérez H. 2013. Extreme northern range extension of the Pelican barracuda *Sphyraena idiastes* (Perciformes: Sphyraenidae) in the eastern Pacific. *Journal of Applied Ichthyology* 29:655-657. DOI 10.1111/jai.12102.

Goode GB, and Bean TH. 1879. Catalogue of a collection of fishes sent from Pensacola, Florida, and vicinity, by Mr. Silas Stearns, with descriptions of six new species. *Proceedings of the United Stated National Museum* 2:121–156.

Kara H, and Bourehail N. 2020. First record of the Indo-Pacific yellowtail barracuda, *Sphyraena flavicauda* (Actinopterygii: Perciformes: Sphyraenidae), in the Western Mediterranean. *Acta Ichthyologica et Piscatoria* 50:363-366. DOI 10.3750/aiep/02987.

Manzoor H, Osmany HB, and Zohra K. 2020. First record of the Arabian barracuda *Sphyraena arabiansis* Abdussamad & Retheesh, 2015 (Family: Sphyraenidae) from Pakistan. *International Journal of Biology and Biotechnology* 17:463-468.

Morishita S, Miki R, Wada H, Itou M, and Motomura H. 2020. Morphological comparisons of *Sphyraena qenie* with *S. putnamae*, with a revised key to Indo-Pacific species of *Sphyraena* lacking gill rakers (Sphyraenidae). *Ichthyological Research* 67:456–463. DOI 10.1007/s10228-020-00738-6.

Whitley GP. 1947. New sharks and fishes from Western Australia. *The Australian Zoologist* 11:129-150.

Williams F. 1959. The barracudas (Genus *Sphyraena*) in British East African waters. *Annals and Magazine of Natural History* 2:92-128. DOI 10.1080/00222935908651031.

**Polynemidae**

Motomura H. 2001. A record of a threadfin, *Eleutheronema tetradactylum*, from Aomori Prefecture, northern Japan, and description of a newly-recognized diagnostic character for the species (Perciformes: Polynemidae). *Japanese Journal of Ichthyology* 48:41-47.

Motomura H, Ito M, Takayama M, Haraguchi Y, and Matsunuma M. 2007. Second Japanese Record of a Threadfin, *Eleutheronema rhadinum* (Perciformes, Polynemidae), with Distributional Implications. *Biogeography* 9:7-11.

### Centrarchiformes

**Kyphosidae**

Azzurro E, Peña-Rivas L, Lloris D, and Bariche M. 2013. First documented occurrence of *Kyphosus incisor* in the Mediterranean Sea. *Marine Biodiversity Records* 6. DOI 10.1017/s1755267213000717.

Canas A, Vasconcelos P, Lino PG, and Santos MN. 2005. Northernmost record of *Kyphosus sectator* (Osteichthyes: Perciformes: Kyphosidae) in the north-eastern Atlantic. *Journal of the Marine Biological Association of the United Kingdom* 85:1535-1537. DOI 10.1017/S0025315405012750.

Elbaraasi H, Bograra O, Elsilini O, and Bojwari J. 2013. First record of the Bermuda sea chub, *Kyphosus saltatrix* (Actinopterygii: Perciformes: Kyphosidae) in the coastal waters of Libya. *Acta Ichthyologica et Piscatoria* 43:251-253. DOI 10.3750/AIP2013.43.3.09.

Francour P, and Mouine N. 2006. First record of *Kyphosus sectator* (Kyphosidae) along the French Mediterranean coast. *Cybium* 32:275-276.

Groud LL, Chaoui L, and Kara MH. 2021. A new record of the brassy chub, Kyphosus vaigiensis (Actinopterygii: Perciformes: Kyphosidae), from the Mediterranean Sea. *Acta Ichthyologica et Piscatoria* 51:219-223.

Kiparissis S, Loukovitis D, and Batargias C. 2012. First record of the Bermuda sea chub *Kyphosus saltatrix* (Pisces: Kyphosidae) in Greek waters. *Marine Biodiversity Records* 5. DOI 10.1017/s1755267211001199.

Kiyağa VB, Mavruk S, Özyurt CE, Akamca E, and Coşkun Ç. 2019. Range extension of *Kyphosus vaigiensis* (Quoy & Gaimard, 1825) in the northeastern Mediterranean, İskenderun Bay, Turkey. *Turkish Journal of Zoology* 43:644-649. DOI 10.3906/zoo-1901-1.

**Latridae**

Roberts CD. 2003. A new species of trumpeter (Teleostei; Percomorpha; Latridae) from the central South Pacific Ocean, with a taxonomic review of the striped trumpeter *Latris lineata*. *Journal of the Royal Society of New Zealand* 33:731-754. DOI 10.1080/03014223.2003.9517756.

**Terapontidae**

Al Mabruk S, Zava B, Nour OM, Corsini-Foka M, and Deidun A. 2021. Record of *Terapon jarbua* (Forsskål, 1775) (Terapontidae) and *Acanthopagrus bifasciatus* (Forsskål, 1775) (Sparidae) in the Egyptian Mediterranean waters. *BioInvasions Records* 10:710-720. DOI 10.3391/bir.2021.10.3.21.

Bogorodsky SV, Alpermann TJ, Mal AO, and Gabr MH. 2014. Survey of demersal fishes from southern Saudi Arabia, with five new records for the Red Sea. *Zootaxa* 3852:401-437. DOI 10.11646/zootaxa.3852.4.1.

Golani D, and Appelbaum-Golani B. 2010. First record of the Indo-Pacific fish the Jarbua terapon (*Terapon jarbua*) (Osteichthyes: Terapontidae) in the Mediterranean with remarks on the wide geographical distribution of this species. *Scientia Marina* 74:717-720. DOI 10.3989/scimar.2010.74n4717.

### Cichliformes

Daneshvar E, Keivany Y, and Paknehad E. 2013. Comparative Biometry of the Iranian Cichlid, *Iranocichla hormuzensis*, in Different Seasons and Sexes. *Research in Zoology* 3:56–61. DOI 10.5923/j.zoology.20130302.02.

Nyom ARB, Kitio HI, Njom SD, Pariselle A, Snoeks J, and Bilong CFB. 2021. Morphological variations, length-weight relationships and condition factors of *Hemichromis camerounensis* (Cichliformes, Cichlidae) in three lakes in northern Cameroon. *International Journal of Fisheries and Aquaculture* 9:139-145. DOI 10.22271/fish.2021.v9.i3b2500.

Turner GF, and Stauffer JR. 1998. Three new deep water cichlid fishes of the genus *Diplotaxodon* from Lake Malawi, with a redescription of *Diplotaxodon ecclesi*. *Ichthyological Exploration of Freshwaters* 8:239-252.

### Eupercaria incertae sedis

**Nemipteridae**

Randall JE. 1997. Randall's tank photos. Collection of 10,000 large-format photos (slides) of dead fishes. *Available at www.fishbase.org (see species pages for more details)* (accessed June 29 2022).

**Sciaenidae**

Aydin M, and Bodur B. 2021. Morphologic Characteristics and Length-Weight Relationships of *Sciaena umbra* (Linnaeus, 1758) in the Black Sea Coast. *Marine Science and Technology Bulletin* In Press:8-15. DOI 10.33714/masteb.738661.

Cengiz Ö, Kızılkaya B, and Paruğ ŞŞ. 2019. Ege Denizi İçin Eşkina Balığı’nın (*Sciaena umbra* Linnaeus, 1758) Maksimum Boy Kaydı. *Kahramanmaraş Sütçü İmam Üniversitesi Tarım ve Doğa Dergisi* 22:659-663. DOI 10.18016/ksutarimdoga.vi.515704.

Cengiz O, and Parug SS. 2021. A New Maximum Size Record of the Shi Drum (*Umbrina cirrosa* Linnaeus, 1758) for Aegean Sea. *Brazilian Journal of Biology* 81:461-463. DOI 10.1590/1519-6984.231643.

Harayashiki C, Junior ASV, Burns M, and Vieira JP. 2014. Establishing evidence of a non-native species *Pachyurus bonariensis* Steindachner, 1879 (Perciformes, Sciaenidae) in Mirim Lagoon, Rio Grande do Sul (Brazil). *BioInvasions Records* 3:103-110. DOI 10.3391/bir.2014.3.2.08.

Kumar T, Chakraborty S, Jaiswar A, Sandhya K, and Panda D. 2012. Biometric studies on *Johnieops sina* (Cuvier, 1830) along Ratnagiri coast of Maharashtra. *Indian Journal of Fisheries* 559:7–13.

Tokaç A, Akyol O, Tosunoğlu Z, Aydin C, and Kaykaç H. 2017. Occurrence of a Huge Meagre, *Argyrosomus regius* in İzmir Bay (Aegean Sea, Turkey). *Turkish Journal of Maritime and Marine Sciences* 3:63-66.

Whitley GP. 1947. New sharks and fishes from Western Australia. *The Australian Zoologist* 11:129-150.

### Gobiiformes

Randall JE. 1997. Randall's tank photos. Collection of 10,000 large-format photos (slides) of dead fishes. *Available at www.fishbase.org (see species pages for more details)* (accessed June 29 2022).

Trkov D, and Lipej L. 2020. First record of the cryptobenthic goby *Odondebuenia balearica* (Pellegrin & Fage, 1907) in Slovenia. *Mediterranean Marine Science* 21:350.

### Istiophoriformes

**Istiophoridae**

Beerkircher LR, Lee D, and Hinteregger GF. 2008a. Roundscale spearfish *Tetrapturus georgii* (Lowe, 1840); updated morphology, distribution, and relative abundance in the western North Atlantic. *NOAA Technical Memorandum* NFMS-SEFSC-571:1-23.

Beerkircher LR, Lee DW, and Hinteregger GF. 2008b. Roundscale spearfish *Tetrapturus georgii*: morphology, distribution, and relative abundance in the western North Atlantic. *Bulletin of Marine Science* 82:155–170.

Conrad GM, and LaMonte F. 1937. Observations on the body form of the blue marlin (*Makaira nigricans ampla* Poey). *Bulletin of the American Museum of Natural History* 74:207–220.

De La Cruz-Agüero J, González-Armas R, García-Rodríguez FJ, and Cota-Gómez VM. 2019. First record of a specimen of the shortbill spearfish *Tetrapturus angustirostris* Tanaka, 1915 in the Pacific coast of Mexico. *Latin American Journal of Aquatic Research* 47:677–683. DOI doi:10.3856/vol47-issue4-fulltext-9.

Gregory WK, and Conrad GM. 1939. Body-forms of the black marlin (*Makaira nigricans marlina*) and striped marlin (*Makaira mitsukurii*) of New Zealand and Australia. *Bulletin of the American Museum of Natural History* 76:443–456.

Morrow JE, Jr. 1958. Names of the blue marlin and black marlin. *Bulletin of Marine Science* 8:356-359.

Nichols JT, and LaMonte F. 1935. The Tahitian black swordfish, or silver marlin swordfish. *American Museum Novitates* 807:1-2.

Prager MH, Prince ED, and Lee DW. 1995. Empirical length and weight conversions for blue marlin, white marlin, and sailfish from the North Atlantic Ocean. *Bulletin of Marine Science* 56:201-210.

Randall JE. 1997. Randall's tank photos. Collection of 10,000 large-format photos (slides) of dead fishes. *Available at www.fishbase.org (see species pages for more details)* (accessed June 29 2022).

Robins CR. 1974. Summer concentration of white marlin, *Tetrapturus albidus*, west of the straits of Gibraltar. In: Shomura S, and Williams F, eds. *Proceedings of the international billfish symposium Kailua-Kona, Hawaii, 9-12 August 1972 Part 2 Review and Contributed Papers*: National Oceanic and Atmospheric Administration, NOAA Technical Report NMFS SSRF-675, 164-174.

Robins CR, and De Sylva DP. 1963. A new western Atlantic spearfish, *Tetrapturus pfluegeri*, with a redescription of the Mediterranean spearfish *Tetrapturus belone*. *Bulletin of Marine Science of the Gulf and Caribbean* 13:85–122.

Velayudham R, Veeramuthu S, and Kesavan K. 2012. Length-weight relationship and morphometrics of the sailfish, Istiophorus platypterus (Shaw & Nodder) from Parangipettai, Southeast coast of India. *Asian Pacific Journal of Tropical Biomedicine* 2:S373–S376.

**Xiphiidae**

Gregory, WK, and Conrad GM. 1937. The comparative osteology of the swordfish (*Xiphias*) and the sailfish (*Istiophorus*). *American Museum Novitates* 952:1-25.

Quigley D, and Flannery K. 1989. Swordfish *Xiphias gladius* (L.). *The Irish Naturalists' Journal* 23:116–116.

Raven, HC, and F. LaMonte, F. 1937. Notes on the alimentary tract of the swordfish (*Xiphias gladius*). *American Museum Novitates* 902:1–13.

Taylor RG, and Murphy MD. 1992. Reproductive biology of the swordfish *Xiphias gladius* in the Straits of Florida and adjacent waters. *Fishery Bulletin* 90:809-816.

### Labriformes

Rafrafi-Nouira S, Boumaïza M, Reynaud C, and Capapé C. 2012. Confirmed occurence of cuckoo wrasse *Labrus mixtus* (Osteichthyes: Labridae) in Tunisian waters (central Mediterranean. *Annales: Series Historia Naturalis* 22:115–120.

**Labridae**

Randall JE. 1997. Randall's tank photos. Collection of 10,000 large-format photos (slides) of dead fishes. *Available at www.fishbase.org (see species pages for more details)* (accessed June 29 2022).

**Scaridae**

Kampouris TE, and Batjakas IE. 2018. The northernmost record of the thermophilic Mediterranean parrotfish *Sparisoma cretense* (Linnaeus, 1758) (Perciformes, Scaridae) in the eastern Mediterranean Sea (northwestern Aegean Sea). *Annales, Series historia naturalis* 28:111-116. DOI 10.19233/ASHN.2018.13.

Ogihara G, Yoshida T, Ito M, Yamashita M, Sakurai Y, and Motomura H. 2010. Record of *Bolbometopon muricatum* (Labroidei: Scaridae) from Kasasa, Kagoshima, southern Kyushu, Japan. *Nature of Kagoshima* 36:43-47.

Randall JE, and Earle JL. 1993. *Scarus obishime*, a new parrotfish (Perciformes: Scaridae) from the Ogasawara Islands. *Japanese Journal of Ichthyology* 39:287-293.

Westneat MW, Satapoomin U, and Randall JE. 2007. *Scarus maculipinna*, a new species of parrotfish (Perciformes, Scaridae) from the eastern Indian Ocean. *Zootaxa* 1628:59-68. DOI 10.11646/zootaxa.1628.1.4.

### Lutjaniformes

**Lutjanidae**

Bos AR, and Gumanao GS. 2013. Seven new records of fish (Teleostei: Perciformes) from coral reefs and pelagic habitats in southern Mindanao, the Philippines. *Marine Biodiversity Records* 6:e95. DOI 10.1017/S1755267213000614.

García-Mederos AM, and Tuset VM. 2014. First record of African brown snapper *Lutjanus dentatus* in the Canary Islands (north-eastern Atlantic Ocean). *Marine Biodiversity Records* 7:e65. DOI 10.1017/S1755267214000682.

Goode GB, and Bean TH. 1878. Description of two new species of fishes, *Lutjanus blackfordii* and *Lutjanus stearnsii*, from the coast of Florida. *Proceedings of the United Stated National Museum* 1:176–181. DOI https://doi.org/10.5479/si.00963801.1-24.176.

Goode GB, and Bean TH. 1879. Catalogue of a collection of fishes sent from Pensacola, Florida, and vicinity, by Mr. Silas Stearns, with descriptions of six new species. *Proceedings of the United Stated National Museum* 2:121–156.

Hata H, Haraguchi Y, and Motomura H. 2015. First record of *Etelis radiosus* (Perciformes: Lutjanidae) from the Tokara Islands in the Ryukyu Islands, southern Japan. *Nature of Kagoshima* 41:95-99.

Kim MJ, Mun TS, Lee J-H, Choi YM, and Song CB. 2015. New Record of the Chinamanfish, *Symphorus nematophorus* (Perciformes: Lutjanidae) from Korea. *Korean Journal of Ichthyology* 27:149-152.

Schwartz FJ. 1972. Recent Occurrences of Cubera Snappers (Pisces, Lutjanidae) in North Carolina Atlantic Ocean Waters. *Journal of the Elisha Mitchell Scientific Society* 88:252–254.

### Mugiliformes

**Mugilidae**

Randall JE. 1997. Randall's tank photos. Collection of 10,000 large-format photos (slides) of dead fishes. *Available at www.fishbase.org (see species pages for more details)* (accessed June 29 2022).

### Ophidiiformes

**Ophidiidae**

Pan M, Ouréns R, Dione L, Samba I, Sánchez-Carnero N, and Freire J. 2015. Length-weight relationships for 13 fish species from a coastal artisanal fishery at Cape Verde peninsula (Senegal). *Journal of Applied Ichthyology* 31:1177-1179. DOI 10.1111/jai.12890.

### Ovalentaria incertae sedis

**Ambassidae**

Randall JE. 1997. Randall's tank photos. Collection of 10,000 large-format photos (slides) of dead fishes. *Available at www.fishbase.org (see species pages for more details)* (accessed June 29 2022).

**Pseudochromidae**

Randall JE. 1997. Randall's tank photos. Collection of 10,000 large-format photos (slides) of dead fishes. *Available at www.fishbase.org (see species pages for more details)* (accessed June 29 2022).

### Pempheriformes

**Champsodontidae**

Bogorodsky SV, Alpermann TJ, Mal AO, and Gabr MH. 2014. Survey of demersal fishes from southern Saudi Arabia, with five new records for the Red Sea. *Zootaxa* 3852:401-437. DOI 10.11646/zootaxa.3852.4.1.

Yapici S, Karachle PK, and Filiz H. 2015. First length-weight relationships of 11 fish species in the Aegean Sea. *Journal of Applied Ichthyology* 31:398-402. DOI 10.1111/jai.12459.

### Perciformes

**Acropomatidae**

Carvalho-Filho A, Marcovaldi G, Sampaio CA, Paiva MIG, and Duarte I. 2010. Two new records of uncommon deep-sea perciform fishes from the Southwestern Atlantic. *Zootaxa* 2694:59-68.

**Centropomidae**

Pinho SM, David LH, Garcia F, Keesman KJ, Portella MC, and Goddek S. 2021. South American fish species suitable for aquaponics: a review. *Aquaculture International* 29:1427-1449. DOI 10.1007/s10499-021-00674-w.

**Channichthyidae**

La Mesa M, Vacchi M, Iwami T, and Eastman J. 2002. Taxonomic studies of the Antarctic icefish genus *Cryodraco* Dollo, 1900 (Notothenioidei: Channichthyidae). *Polar Biology* 25:384-390. DOI 10.1007/s00300-002-0358-y.

**Emmelichthyidae**

Yoshino T, and Kon T. 2000. First record of an emmelichthyid fish, *Emmelichthys karnellai*, from the Western North Pacific (Pisces: Perciformes: Emmelichthyidae). *Biogeography* 2:63-65.

**Lobotidae**

Daban İB, and Cabbar K. 2021. New Occurrence of Atlantic Tripletail, *Lobotes surinamensis* (Bloch, 1790) from the Turkish Part of the Aegean Sea, with Biological Notes. *Acta Aquatica Turcica* 17:327-333. DOI 10.22392/actaquatr.814774.

Soufi-Kechaou E, Ounifi-Ben Amour K, Ben Souissi J, and Rafrafi-Nouira S. 2018. Additional record of tripletail *Lobotes surinamensis* (Osteichthyes: Lobotidae) in Tunisian waters (central Mediterranean Sea). *Annales, Series historia naturalis* 28:37-42. DOI 10.19233/ASHN.2018.06.

**Haemulidae**

Al-Busaidi HK, Govender A, Al-Jufaily SM, and Al-Marzouqi A. 2021. Population Biology Including Population Structure, Spawning Cycle, and Maturity of *Plectorhinchus schotaf* (Forsskål, 1775) (Family: Haemulidae) Collected from the Arabian Sea Coast of Oman. In: Jawad LA, ed. *The Arabian Seas: Biodiversity, Environmental Challenges and Conservation Measures*. Cham: Springer International Publishing, 595-624.

Baset A, Liu Q, Liao B, Waris A, Ahmad I, Yanan H, and Qingqing Z. 2020. Population Dynamics of Saddle Grunt Fish, *Pomadasys maculatus* (Bloch, 1793) from Pakistani Waters. *Bioprocess Engineering* 4. DOI 10.11648/j.be.20200401.11.

Bogorodsky SV, Alpermann TJ, Mal AO, and Gabr MH. 2014. Survey of demersal fishes from southern Saudi Arabia, with five new records for the Red Sea. *Zootaxa* 3852:401-437. DOI 10.11646/zootaxa.3852.4.1.

Hata H, Fujiwara K, Takayama M, and Motomura H. 2014. First records of *Plectorhinchus schotaf* (Perciformes: Haemulidae) from Kagoshima Prefecture, southern Japan. *Nature of Kagoshima* 40:53-57.

Randall JE. 1997. Randall's tank photos. Collection of 10,000 large-format photos (slides) of dead fishes. *Available at www.fishbase.org (see species pages for more details)* (accessed June 29 2022).

Safi A, Khan MA, and Khan MZ. 2014a. Study of some morphometric and meristic characters of saddle grunt fish, *Pomadasys maculatus* (Bloch, 1793), off Karachi Coast, Pakistan. *American Journal of Marine Science* 2:38-42. DOI 10.12691/marine-2-2-2.

Safi A, Khan MA, and Khan MZ. 2014b. Study of some morphometric and meristic characters of striped piggy fish, *Pomadasys stridens* (Forsskal, 1775) from Karachi Coast, Pakistan. *The Journal of Zoology Studies* 1:1–6.

**Malacanthidae**

Goode GB, and Bean TH. 1878. Description of *Caulolatilus microps*, a new species of fish from the Gulf coast of Florida. *Proceedings of the United Stated National Museum* 1:42–45. DOI https://doi.org/10.5479/si.00963801.1-16.42.

Goode GB, and Bean TH. 1879. Description of a new genus and species of fish, *Lopholatilus chamaeleonticeps*, from the south coast of New England. *Proceedings of the United Stated National Museum* 2:205–209.

Pulver JR, and Whatley A. 2016. Length-weight relationships, location, and depth distributions for select Gulf of Mexico reef fish species. *NOAA Technical Memorandum* NMFS-SEFSC-693:1-100. DOI 10.7289/V58G8HQ2.

**Moronidae**

Meads DR, Crawford MK, and Dockendorf KJ. 2011. Predicting Weights of Striped Bass *Morone saxatilis* in the Roanoke River, North Carolina. *Journal of North Carolina Academy of Science* 127:216-218. DOI 10.7572/2167-5880-127.3.216.

**Mullidae**

Azzouz K, Diatta Y, Mansour S, Boumaïza M, Ben Amor MM, and Capapé C. 2011. First record of the west African goatfish, *Pseudupeneus prayensis* (Actinopterygii: Perciformes: Mullidae), off the Tunisian coast (central Mediterranean). *Acta Ichthyologica et Piscatoria* 41:133-136. DOI 10.3750/aip2011.41.2.10.

Bariche M, Bilecenoglu M, and Azzurro E. 2013. Confirmed presence of the Red Sea goatfish *Parupeneus forsskali* (Fourmanoir & Guézé, 1976) in the Mediterranean Sea. *BioInvasions Records* 2:173-175. DOI 10.3391/bir.2013.2.2.15.

Mehanna SF, Osman AGM, Farrag MMS, and Osman YAA. 2018. Age and growth of three common species of goatfish exploited by artisanal fishery in Hurghada fishing area, Egypt. *Journal of Applied Ichthyology* 34:917-921. DOI 10.1111/jai.13590.

**Pinguipedidae**

Bogorodsky SV, Alpermann TJ, Mal AO, and Gabr MH. 2014. Survey of demersal fishes from southern Saudi Arabia, with five new records for the Red Sea. *Zootaxa* 3852:401-437. DOI 10.11646/zootaxa.3852.4.1.

**Pomacanthidae**

Capapé C, Ali M, Ali A, and Esmail A. 2018. Second Mediterranean record of Emperor angelfish, *Pomacanthus imperator* (Osteichthyes: Pomacanthidae), and first record from the Syrian coast. *Cahiers de Biologie Marine* 59:396-397. DOI 10.21411/CBM.A.8C69C609.

Golani D, Salameh P, and Sonin O. 2010. First record of the Emperor angelfish, *Pomacanthus imperator* (Teleostei: Pomacanthidae) and the second record of the spotbase burrfish *Cyclichthys spilostylus* (Teleostei: Diodontidae) in the Mediterranean. *Aquatic Invasions* 5:S41-S43. DOI 10.3391/ai.2010.5.S1.010.

Randall JE. 1997. Randall's tank photos. Collection of 10,000 large-format photos (slides) of dead fishes. *Available at www.fishbase.org (see species pages for more details)* (accessed June 29 2022).

**Serranidae**

Bos AR, and Gumanao GS. 2013. Seven new records of fish (Teleostei: Perciformes) from coral reefs and pelagic habitats in southern Mindanao, the Philippines. *Marine Biodiversity Records* 6:e95. DOI 10.1017/S1755267213000614.

Bullock LH, Murphy MD, Godcharles MF, and Mitchell ME. 1992. Age, growth, and reproduction of jewfish *Epinephelus itajara* in the eastern Gulf of Mexico. *Fishery Bulletin* 90:243-249.

Francesconi JJ, and Schwartz FJ. 2000. Jewfish, *Epinephelus itajara*, from North Carolina, with range correction and body comparisons. *Journal of the Elisha Mitchell Scientific Society* 116:167–170.

Gökoğlu M, and Özvarol Y. 2015. *Epinephelus coioides* (Actinopterygii: Perciformes: Serranidae)—a new Lessepsian migrant in the Mediterranean coast of Turkey. *Acta Ichthyologica et Piscatoria* 45:307–309. DOI 10.3750/aip2015.45.3.09.

Goode GB, and Bean TH. 1878. A note upon the black grouper (*Epinephelus nigritus* (Holbrook) Gill) of the southern coast. *Proceedings of the United Stated National Museum* 1:182–184. DOI https://doi.org/10.5479/si.00963801.1-26.182.

Goode GB, and Bean TH. 1879. Catalogue of a collection of fishes sent from Pensacola, Florida, and vicinity, by Mr. Silas Stearns, with descriptions of six new species. *Proceedings of the United Stated National Museum* 2:121–156.

Kulbicki M, Guillemot N, and Amand M. 2005. A general approach to length-weight relationships for New Caledonian lagoon fishes. *Cybium* 29:232-252.

Nakamura J, and Motomura H. 2022. Review of all Japanese species and standard Japanese names of families, subfamilies, and genera, previously included in the family Serranidae sensu Johnson (1983). *Ichthy, Natural History of Fishes of Japan* 19:26-43.

Randall JE. 1997. Randall's tank photos. Collection of 10,000 large-format photos (slides) of dead fishes. *Available at www.fishbase.org (see species pages for more details)* (accessed June 29 2022).

Smith CL. 1971. A revision of the American groupers : *Epinephelus* and allied genera. *Bulletin of the American Museum of Natural History* 146.

Sureandiran B, Haq MB, Meetei KB, and Karuppasamy K. 2020. Report on Rare Occurrence of Queensland Grouper, *Ephinephelus Lanceolatus*, (Bloch, 1790) From Cuddalore Waters, South East Coast Of India. *International Journal of Future Generation Communication Networking* 13:3983–3990.

Thomas S, Sreeram MP, George RM, Naomi TS, and Sanil NK. 2008. First record of occurrence of Boulenger’s anthias *Sacura boulengeri* (Heemstra, 1973), Family: Serranidae, in Indian waters. *Journal of the Marine Biological Association of India* 50:69-73.

**Siganidae**

Dulčić J, Dragičević B, Grgičević R, and Lipej L. 2011. First substantiated record of a Lessepsian migrant—the dusky spinefoot, *Siganus luridus* (Actinopterygii: Perciformes: Siganidae), in the Adriatic Sea. *Acta Ichthyologica et Piscatoria* 41:141-143. DOI 10.3750/aip2011.41.2.12.

Rafrafi-Nouira S, Boumaïza M, Reynaud C, and Capape C. 2012. Additional records of Lessepian teleost species off the Tunisian coast (central Mediterranean). *Annales, Series historia naturalis* 22:55-62.

Soykan O, Gülşahin A, and Cerim H. 2020. Maximum size of Marbled spinefoot (*Siganus rivulatus* Forsskal & Niebuhr, 1775) for Aegean Sea. *Aquatic Sciences and Engineering* 36:42-45. DOI 10.26650/ase2020727909.

**Sillagidae**

Innal D, Kisin B, and Akdoganbulut D. 2015. Length-weight Relationships and Morphometry of *Sillago suezensis* from Antalya Gulf-Turkey *International Journal of Fisheries and Aquatic Studies* 2:107-112.

Kulbicki M, Guillemot N, and Amand M. 2005. A general approach to length-weight relationships for New Caledonian lagoon fishes. *Cybium* 29:232-252.

Pradhan SK, Roul SK, Ghosh S, Tripathy P, Jaiswar AK, Bhusan S, and Nayak BB. 2020. Biometry, length-weight and length-length relationships of flathead sillago *Sillaginopsis panijus* (Hamilton, 1822) (Perciformes: Sillaginidae) from the north-western Bay of Bengal. *Indian Journal of Fisheries* 67:144-151. DOI 10.21077/ijf.2020.67.3.95369-16.

Randall JE. 1997. Randall's tank photos. Collection of 10,000 large-format photos (slides) of dead fishes. *Available at www.fishbase.org (see species pages for more details)* (accessed June 29 2022).

### Pleuronectiformes

Goode GB, and Bean TH. 1879. Catalogue of a collection of fishes sent from Pensacola, Florida, and vicinity, by Mr. Silas Stearns, with descriptions of six new species. *Proceedings of the United Stated National Museum* 2:121–156.

Randall JE. 1997. Randall's tank photos. Collection of 10,000 large-format photos (slides) of dead fishes. *Available at www.fishbase.org (see species pages for more details)* (accessed June 29 2022).

### Priacanthiformes

Alshawy FA, Ibrahim A, Hussein C, and Lahlah M. 2019. First record of arrow bulleye, *Priacanthus sagittarius* Starnes, 1988 from the Syrian marine waters (Eastern Mediterranean). *FishTaxa* 4:21–24.

Gökoğlu M, and Teker S. 2018. Spread of the arrow bulleye *Priacanthus sagittarius* Starnes, 1988 in the Mediterranean Sea. *Acta Aquatica: Aquatic Sciences Journal* 5:1–3.

Jeong B, and Motomura H. 2014. First records of *Priacanthus zaiserae* (Perciformes: Priacanthidae) from Amami-oshima island, Kagoshima Prefecture, Japan. *Nature of Kagoshima* 40:43-46.

### Kurtiformes

Said Koya KP, Akhilesh KV, and Bineesh KK. 2011. A new record of Titan cardinalfish, *Holapogon maximus* (Apogonidae) along the south-west coast of India. *Marine Biodiversity Records* 4:e36. DOI 10.1017/s1755267211000340.

**Apogonidae**

Bogorodsky SV, Alpermann TJ, Mal AO, and Gabr MH. 2014. Survey of demersal fishes from southern Saudi Arabia, with five new records for the Red Sea. *Zootaxa* 3852:401-437. DOI 10.11646/zootaxa.3852.4.1.

KebapçIoğlu T, and Beğburs CR. 2020. Distributions and Length-Weight Relationships of Some Lessepsian Cardinalfishes (Apogonid species) in the Northeastern Mediterranean (Antalya, Turkey). *Acta Aquatica Turcica* 17:72-77. DOI 10.22392/actaquatr.755915.

### Scombriformes

**Bramidae**

Ali AM, and McNoon AH. 2010. Additions to Benthopelagic Fish Fauna of the Aden Gulf-Arabian Sea (Actinopterygii: Bramidae and Sternoptychidae). *Journal of Fisheries and Aquatic Science* 5:23-32. DOI 10.3923/jfas.2010.23.32.

Bos AR, and Gumanao GS. 2013. Seven new records of fish (Teleostei: Perciformes) from coral reefs and pelagic habitats in southern Mindanao, the Philippines. *Marine Biodiversity Records* 6:e95. DOI 10.1017/S1755267213000614.

Carvalho-Filho A, Marcovaldi G, Sampaio CA, Paiva MIG, and Duarte LAG. 2009. First report of rare pomfrets (Teleostei: Bramidae) from Brazilian waters, with a key to Western Atlantic species. *Zootaxa* 2290:1–26.

Jawad LA, Al-Mamry J, and Al-Busaidi HK. 2014. New Record of the Keeltail Pomfret, *Taractes rubescens* (Jordan & Evermann, 1887) (Perciformes: Bramidae) from the Sea of Oman. *International Journal of Marine Science* 4:227-230. DOI 10.5376/ijms.2014.04.0025.

Lee J-H, Lee W-J, and Kim J-K. 2019. First Reliable Record of the Keeltail Pomfret *Taractes rubescens* (Bramidae: Perciformes) from Korea. *Korean Journal of Fisheries and Aquatic Sciences* 52:283-287. DOI 10.5657/KFAS.2019.0283.

Mytilineou C, Anastasopoulou A, Christides G, Bekas P, Smith CJ, Papadopoulou KN, Lefkaditou E, and Kavadas S. 2013. New records of rare deep-water fish species in the Eastern Ionian Sea (Mediterranean Sea). *Journal of Natural History* 47:1645-1662. DOI 10.1080/00222933.2013.775372.

Puentes V, Rubio EA, and Zapata LA. 2001. Primer registro del género *Taractes* (Pisces: Bramidae) en el océano Pacífico colombiano. *Boletín de Investigaciones Marinas y Costeras-INVEMAR* 30:207-212.

**Centrolophidae**

Akyol O, and Özgül A. 2018. A new record of imperial blackfish, *Schedophilus ovalis* (Cuvier, 1833) from the Bay of Izmir (Aegean Sea, Turkey). *Journal of the B* 24:277-280.

Ayas D, Erguden D, Çiftçi N, and Bakan M. 2018. The New Locality Record of *Centrolophus niger* (Gmelin, 1789), from Büyükeceli Coast (North-Eastern Mediterranean Sea). *Comu Journal of Marine Science and Fisheries* 1:48-52.

Mackay KT. 1972. Further Records of the Stromateoid Fish *Centrolophus niger* from the Northwestern Atlantic, with Comments on Body Proportions and Behavior. *Copeia* 1972:185-187. DOI 10.2307/1442800.

Merriner JV, Foster WA, and Schwartz FJ. 1970. The Barrelfish, *Hyperoglyphe perciformis* (Pisces: Stromateidae), in Pamlico Sound, N. C., and Adjacent Atlantic Ocean. *Journal of the Elisha Mitchell Scientific Society* 86:28–30.

Mytilineou C, Anastasopoulou A, Christides G, Bekas P, Smith CJ, Papadopoulou KN, Lefkaditou E, and Kavadas S. 2013. New records of rare deep-water fish species in the Eastern Ionian Sea (Mediterranean Sea). *Journal of Natural History* 47:1645-1662. DOI 10.1080/00222933.2013.775372.

Reyes P, Hüne M, and Rui VH. 2007. El pez mesopelagico *Centrolophus niger* (Gmelin, 1789) (Pisces: Perciformes) y su hallazgo en aguas del talud continental chileno situadas sobre la placa sudamericana. *Gayana* 71:96-101. DOI 10.4067/S0717-65382007000100010

**Icosteidae**

Allen GH. 2001. The Ragfish, *Icosteus aenigmaticus* Lockington, 1880: A Synthesis of Historical and Recent Records From the North Pacific Ocean and the Bering Sea. *Marine Fisheries Review* 63:1-31.

Kubota T, and Uyeno T. 1971. First record of an adult specimen of ragfish *Icosteus aenigmaticus* from Japan. *Japanese Journal of Ichthyology* 18:51–54.

**Thunnini**

Adams JL, and Kerstetter DW. 2014. Age and Growth of Three Coastal-Pelagic Tunas (Actinopterygii: Perciformes: Scombridae) in the Florida Straits, USA: Blackfin Tuna, *Thunnus atlanticus*, Little Tunny, *Euthynnus alletteratus*, and Skipjack Tuna, *Katsuwonus pelamis*. *Acta Ichthyologica et Piscatoria* 3.

Allaya H, Faleh AB, Rebaya M, Zrelli S, Hattour A, Quignard J-P, and Trabelsi M. 2017. Morphological differences Between two populations of the Little Tunny, *Euthynnus alletteratus* (Rafinesque, 1810) in Tunisian Waters (Central Mediterranean Sea). *Pakistan Journal of Zoology* 49:2027–2035. DOI 10.17582/journal.pjz/2017.49.6.2027.2035.

Allaya H, Faleh AB, Zrelli S, Hajjej G, Hattour A, Quignard J-P, and Trabelsi M. 2016. Morphological variation of bullet tuna *Auxis rochei* (Risso, 1810) from Tunisian Waters. *Acta Zoologica Bulgarica* 68:433–442.

Bullis HR, Jr., and Mather FJ, III. 1956. Tunas of the genus *Thunnus* of the northern Caribbean. *American Museum Novitates* 1765:1–12.

Collete BB, and Chao LN. 1975. Systematics And Morphology Of Bonitos (*Sarda*) And Their Relatives (Scombridae, Sardini) *Fishery Bulletin* 73:516-625.

Insacco G, and Zava B. 2020. New record of *Orcynopsis unicolor* (Geoffroy Saint Hilaire, 1817) in Italian waters. *Mediterranean Marine Science* 21:344-345.

Olsen AM. 1962. *Allothunnus fallai* Serventy-A new record for Australian waters. *Papers and Proceedings of the Royal Society of Tasmania* 96:95–96.

Rivas LR. 1955. A comparison between giant bluefin tuna (*Thunnus thynnus*) from the Straits of Florida and the Gulf of Maine, with reference to migration and population identity. *Proceedings of the Gulf and Caribbean Fisheries Institute Seventh Annual Session*:133–149.

Roberts PE, Eggleston D, and James GD. 1977. Frigate tuna *Auxis thazard* in New Zealand waters. *New Zealand Journal of Marine and Freshwater Research* 11:163–167.

Romeo J, and Mansueti AJ. 1962. Little Tuna, *Euthynnus alletteratus*, in Northern Chesapeake Bay, Maryland, with an Illustration of Its Skeleton. *Chesapeake Science* 3:257-263. DOI 10.2307/1350633.

Russell FS. 1934. Tunny Investigations made in the North Sea on Col. E. T. Peel's Yacht, “St. George,” Summer, 1933. Part I. Biometric Data. *Journal of the Marine Biological Association of the United Kingdom* 19:503–522. DOI 10.1017/S0025315400046592.

Tičina V, Grubišić L, Šegvić Bubić T, and Katavić I. 2011. Biometric characteristics of small Atlantic bluefin tuna (Thunnus thynnus, Linnaeus, 1758) of Mediterranean Sea origin. *Journal of Applied Ichthyology* 27:971-976. DOI https://doi.org/10.1111/j.1439-0426.2011.01752.x.

Wolfe DC, and Webb BF. 1975. Slender tuna (*Allothunnus fallai* Serventy): First record of bulk catches, Tasmania, 1974. *Australian Journal of Marine and Freshwater Research* 26:213-221.

Zaki S, Al-Mamary J, Al-Marzouqi AA, and Al-Kharusi L. 2017. First record of the Pacific bluefin tuna *Thunnus orientalis* (Temminck & Schlegel, 1844) from the coast off Sur, Sultanate of Oman. *International Journal of Environment, Agriculture and Biotechnology* 2:1752–1757. DOI 10.22161/ijeab/2.4.36.

**Non-Thunnin Scombridae**

Beaumarriage DS. 1973. Age, growth, and reproduction of king mackerel, *Scomberomorus cavalla*, in Florida. *Florida Marine Research Publications* 1:1-45.

Butz G, and Mansueti RJ. 1962. First record of the King Mackerel, *Scomberomorus cavalla*, in Northern Chesapeake Bay, Maryland. *Chesapeake Science* 3:130-135. DOI 10.2307/1351227.

Cengiz Ö. 2020. The maximum length record of Atlantic Mackerel (*Scomber scombrus* Linnaeus, 1758) for Turkish Seas. *Marine and Life Sciences* 2:65-70.

Collette BB, and Russo JL. 1980. *Scomberomorus munroi*, a new species of Spanish mackerel from Australia and New Guinea. *Marine and Freshwater Research* 31:241-250.

Conrad GM, and LaMonte F. 1937. Observations on the body form of the blue marlin (*Makaira nigricans ampla* Poey). *Bulletin of the American Museum of Natural History* 74:207–220.

Gnanamuttu JC. 1971. On the occurence of *Rastrelliger faughni* Matsui in the Indian Waters. *Indian Journal of Fisheries* 1-2:170-173.

Goutham J, and Mohanraju R. 2015. Some aspects of mackerel diversity and morphometric studies of *Rastrelliger* genera from Port Blair Andaman waters. *International Journal of Fisheries and Aquatic Studies* 3:196–198.

Hogwarth WT. 1976. Life history aspects of the wahoo *Acanthocybium solandri* (Cuvier and Valenciennes) from the coast of North Carolina PhD. North Carolina State University.

Lheknim V. 2019. First record of double-lined mackerel *Grammatorcynus bilineatus* (Rüppell, 1836) from the Gulf of Thailand. *Phuket Marine Biological Center Research Bulletin* 76:19–23.

Randall JE. 1997. Randall's tank photos. Collection of 10,000 large-format photos (slides) of dead fishes. *Available at www.fishbase.org (see species pages for more details)* (accessed June 29 2022).

Rao KN. 1960. A short account of the wahoo, *Acanthocybium solandri* (Cuvier & Valenciennes). *Journal of the Marine Biological Association of India* 2:132–135.

Romeo T, Azzuro E, and Mostarda E. 2005. Record of *Acanthocybium solandri* in the central Mediterranean sea, with notes on parasites. *Journal of the Marine Biological Association of the United Kingdom* 85:1295-1296. DOI 10.1017/S0025315405012464.

Rotundo MM, Júnior TV, Albert DM, and Doria VS. 2015. Report of the butterfly kingfish, *Gasterochisma melampus* (Scombridae: Gasterochismatinae) in southeastern Brazil. *Pan-American Journal of Aquatic Sciences* 10:293–296.

Santos SR, and Nunan GW. 2015. A record of the Southern Ocean *Gasterochisma melampus* (Teleostei: Scombridae) in the tropical south-western Atlantic, with comments on previous records. *Marine Biodiversity Records* 8. DOI 10.1017/s1755267214001158.

Xu HL, Gu DX, Wang R, Sun JH, and Bai DQ. 2017. Length-weight and length-length relationships of eight fish species from Bohai Bay, China. *Journal of Applied Ichthyology* 33:642-644. DOI 10.1111/jai.13337.

Zorica B, and Sinovčić G. 2008. Biometry, length-length and length-weight relationships of juveniles and adults of Atlantic bonito, *Sarda sarda*, in the eastern Middle Adriatic Sea. *Acta Adriatica: International Journal of Marine Sciences* 49:65-72.

**Pomatomidae**

Virginia Institute of Science. 2017. Picture of adult bluefish on chesapeakebay.net. *Available at https://www.chesapeakebay.net/discover/field-guide/entry/bluefish* (accessed August 21 2022).

**Gempylidae**

Acarli D, Altinağaç U, Özekinci U, and Bayhan B. 2017. The first record of the oilfish Cocco, 1833 (Pisces: Gempylidae) from the Sea of Marmara, Turkey. *Oceanological and Hydrobiological Studies* 46:249–252. DOI doi:10.1515/ohs-2017-0026.

Bartlett MR, and Backus RH. 1962. A Catch of the Rare Gempylid *Lepidocybium flavo-brunneum* (Smith) in the Bahamas. *Copeia* 1962:845-847. DOI 10.2307/1440696.

Ben Amor MM, Ounifi-Ben Amour K, Bdioui M, and Capape C. 2021. The second record of oilfish, *Ruvettus pretiosus* (Gempylidae), in Tunisian waters (central Mediterranean sea). *Annales, Series historia naturalis* 31:211-216. DOI 0.19233/ASHN.2021.25.

Bettoso N, and Dulçic J. 1999. First record of the oilfish *Ruvettus pretiosus* (Pisces: Gempylidae) in the northern Adriatic sea. *Journal of the Marine Biological Association of the United Kingdom* 79:1145-1149.

Gómez-Cubillos MC, and Grijalba-Bendeck M. 2016. Presence of *Ruvettus pretiosus* (Gempylidae) in the Colombian continental Caribbean. *Universitas Scientiarum* 21. DOI 10.11144/Javeriana.SC21-1.porp.

Hata H, and Motomura H. 2017. First record of the snake mackerel *Epinnula magistralis* (Perciformes: Gempylidae) from the Tokara Islands, Japan. *Fauna Ryukyuana* 30:11-15.

Ho HC, Motomura H, Hata H, and Jiang WC. 2017. Review of the fish genus *Epinnula* Poey (Perciformes: Gempylidae), with description of a new species from the Pacific Ocean. *Zootaxa* 4363:393-408. DOI 10.11646/zootaxa.4363.3.5.

Kim MJ, Choi JH, Kim JN, Oh TY, and Lee DW. 2012. First Record of the Black Snoek *Thyrsitoides marleyi* (Pisces: Gempylidae) from Korea. *Fisheries and aquatic sciences* 15:251-253. DOI 10.5657/fas.2012.0251.

Nashad M, Swapnil SS, Pradeep HD, and Monalisa SD. 2018. First record of escolar, *Lepidocybium flavobrunneum* (Smith, 1843) from the Indian EEZ of Andaman Sea. *Indian Journal of Geo Marine Sciences* 47:1409-1412.

Osmany HB, Zohra K, and Manzoor H. 2019. First official record of the snake mackerel, *Gempylus serpens* Cuvier, 1829 (family Gempylidae) in Pakistan. *International Journal of Biology and Biotechnology* 16:979-981.

Randall JE. 1997. Randall's tank photos. Collection of 10,000 large-format photos (slides) of dead fishes. *Available at www.fishbase.org (see species pages for more details)* (accessed June 29 2022).

Song YS, Myoung SH, and Kim J-K. 2020. First Record of the Escolar *Lepidocybium flavobrunneum* (Perciformes: Gempylidae) from Jeju Island, Korea. *Korean Journal of Ichthyology* 32:26-31. DOI 10.35399/isk.32.1.5.

Zohra K, Osmany HB, and Manzoor H. 2020. First record of escolar, *Lepidocybium flavobrunneum* (Smith, 1843) from Pakistani off-shore water with comprehensive profile. *International Journal of Biology and Biotechnology* 17:383-389.

**Stromateoidei**

Ayas D, Erguden D, Çiftçi N, and Bakan M. 2018. The New Locality Record of *Centrolophus niger* (Gmelin, 1789), from Büyükeceli Coast (North-Eastern Mediterranean Sea). *Comu Journal of Marine Science and Fisheries* 1:48-52.

Horn MH, and Haedrich RL. 1973. Systematic and Distributional Status of Psenes sio and Psenes pellucidus (Pisces, Stromateoidei) in the Eastern Pacific. *Copeia* 1973:167–169. DOI 10.2307/1442384.

Mackay KT. 1972. Further Records of the Stromateoid Fish Centrolophus niger from the Northwestern Atlantic, with Comments on Body Proportions and Behavior. *Copeia* 1972:185-187. DOI 10.2307/1442800.

Reyes P, Hüne M, and Rui VH. 2007. El pez mesopelagico *Centrolophus niger* (Gmelin, 1789) (Pisces: Perciformes) y su hallazgo en aguas del talud continental chileno situadas sobre la placa sudamericana. *Gayana* 71:96-101. DOI 10.4067/S0717-65382007000100010

**Scombrolabracidae**

Bineesh KK, Akhilesh KV, Shanis CPR, Abdussamad EM, and Pillai NGK. 2012. First report of longfin escolar, *Scombrolabrax heterolepis* (Perciformes: Scombrolabracidae) from Indian waters. *Marine Biodiversity Records* 5. DOI 10.1017/s1755267212000656.

Carvalho-Filho A, Marcovaldi G, Sampaio CA, Paiva MIG, and Duarte I. 2010. Two new records of uncommon deep-sea perciform fishes from the Southwestern Atlantic. *Zootaxa* 2694:59-68.

Hata H, Koeda K, Ho H-C, and Motomura H. 2019. First record of the longfin escolar *Scombrolabrax heterolepis* Roule, 1921 (Perciformes, Scombrolabracoidei, Scombrolabracidae) from Taiwan. *Platax* 16:83-89. DOI 10.29926/PLATAX.201912_2019.0007.

**Trichiuridae**

Eduardo LN, Villarins BT, Lucena-Frédou F, Frédou T, Lira AS, Bertrand A, and Mincarone MM. 2018. First record of the intermediate scabbardfish *Aphanopus intermedius* (Scombriformes: Trichiuridae) in the western South Atlantic Ocean. *Journal of Fish Biology* 93:992-995. DOI https://doi.org/10.1111/jfb.13796.

Templeman W, and Squires HJ. 1963. Three records of the black scabbard fish, *Aphanopus carbo* Lowe, from the Canadian region of the Western Atlantic. *Journal Fisheries Research Board of Canada* 20:273-278.

### Scorpaeniformes

**Anarhinchadidae**

Liu Y, Li F, Zhang X, Bian L, Chen S, Qu J, Gao W, Liu C, and Ge J. 2021. Morphological Characteristics, Digestive System and Reproductive System of the Wolf-Eel (*Anarrhichthys ocellatus*). *Progress in Fishery Sciences* 42:79–86. DOI 10.19663/j.issn2095-9869.20200211003.

Rodríguez-Cabello C, Diez G, Perez M, and Banon R. 2015. Range extension: records of *Anarhichas denticulatus* (Perciformes: Anarhichadidae) caught in the southern Bay of Biscay (NE Atlantic). *Cybium* 39:309–312.

**Scorpaenidae**

Ali M, Alkusairy H, Saad A, Reynaud C, and Capape C. 2016. First record of *Pterois miles* (Osteichthyes:Scorpaenidae) in Syrian marine waters: Confirmation of its accordance in the eastern Mediterranean. *Tishreen University Journal for Research and Scientific Studies - Biological Series* 38:307-313.

Ali M, Reynaud C, and Capapé C. 2017. Has a viable population of common lionfish, *Pterois miles* (Scorpaenidae), established off the Syrian Coast (Eastern Mediterranean). *Annales, Series historia naturalis* 27:157-162. DOI 10.19233/ASHN.2017.19.

Ben Amor KO, Ben Souissi J, Capapé C, and Ben Amor MM. 2016. Unusual record of bluemouth rockfish *Helicolenus dactylopterus* (Osteichthyes: Scorpaenidae) in Tunis Southern Lagoon (Northern Tunisia, Central Mediterranean). *Journal of Ichthyology* 56:620–624. DOI 10.1134/S0032945216040081.

Guzmán-Méndez I, Rivera-Madrid R, Díaz-Jaimes P, García-Rivas M, Aguilar-Espinosa M, and Arias-González J. 2017. First genetically confirmed record of the invasive devil firefish *Pterois miles* (Bennett, 1828) in the Mexican Caribbean. *BioInvasions Records* 6:99–103. DOI 10.3391/bir.2017.6.2.02.

Oray IK, Sınay E, Saadet Karakulak F, and Yıldız T. 2015. An expected marine alien fish caught at the coast of Northern Cyprus: *Pterois miles* (Bennett, 1828). *Journal of Applied Ichthyology* 31:733-735. DOI 10.1111/jai.12857.

Taskavak E, Gurkan Ş, and Taylan B. 2021. Some Observations on the Depth Range and Size of Devil Firefish *Pterois miles* (Bennett, 1828) (Scorpaenidae) in Silifke Mersin (Turkey). *Acta Natura et Scientia* 2:53–57. DOI 10.29329/actanatsci.2021.314.9.

**Setarchidae**

Romanov EV, Cherel Y, and Marsac F. 2021. New record of *Ectreposebastes niger* (Fourmanoir, 1971) (Setarchidae, Scorpaeniformes): a rare bathypelagic fish from La Pérouse Seamount, Western Indian Ocean, and distribution of *Ectreposebastes* Garman, 1899 in the Indian Ocean. *Zoosystema* 43:283-296. DOI 10.5252/zoosystema2021v43a15.

**Synanceiidae**

Karna SK, Manna RK, Panda D, Mukherjee M, Suresh VR, Raut A, and Mukhopadhyay MK. 2019. First record of *Trachicephalus uranoscopus* (Bloch and Schneider, 1801) from Chilika lagoon, Odisha coast of India. *Indian Journal of Geo Marine Sciences* 48:1335–1337.

### Spariformes

**Lethridinidae**

Borsa P, Béarez P, and Chen W-J. 2010. Gymnocranius oblongus, a new large-eye bream species from New Caledonia (Teleostei: Lethrinidae). *Comptes Rendus Biologies* 333:241–247. DOI https://doi.org/10.1016/j.crvi.2009.12.015.

Randall JE. 1997. Randall's tank photos. Collection of 10,000 large-format photos (slides) of dead fishes. *Available at www.fishbase.org (see species pages for more details)* (accessed June 29 2022).

Reyes RB. 2017. Photos contributed by Rodolfo B. Reyes to Fishbase. *Available at www.fishbase.org (see species pages for more details)* (accessed July 31 2022).

**Sparidae**

Al Mabruk S, Zava B, Nour OM, Corsini-Foka M, and Deidun A. 2021. Record of *Terapon jarbua* (Forsskål, 1775) (Terapontidae) and *Acanthopagrus bifasciatus* (Forsskål, 1775) (Sparidae) in the Egyptian Mediterranean waters. *BioInvasions Records* 10:710-720. DOI 10.3391/bir.2021.10.3.21.

Cengiz Ö. 2019. Maximum Size Record of Sharpsnout Seabream (*Diplodus puntazzo* Walbaum, 1792) for Saros Bay, Northern Aegean Sea. *Marine Science and Technology Bulletin* 8:55-57. DOI 10.33714/masteb.596481.

Cengiz Ö, Kızılkaya B, and Paruğ ŞŞ. 2019a. Growth Characteristics of Annular Seabream (*Diplodus annularis* Linnaeus, 1758) for Turkish Waters. *Kahramanmaraş Sütçü İmam Üniversitesi Tarım ve Doğa Dergisi* 22:817-822. DOI 10.18016/ksutarimdoga.vi.525929.

Cengiz Ö, Paruğ ŞŞ, and Kızılkaya B. 2019b. Maximum Length Record of Common Two-banded Seabream (*Diplodus vulgaris* Geoffroy Saint-Hilaire, 1817) for Aegean Sea with Turkish Waters. *Alinteri Journal of Agriculture Sciences* 34:160–163. DOI 10.28955.alinterizbd.638974.

Goode GB, and Bean TH. 1879. Catalogue of a collection of fishes sent from Pensacola, Florida, and vicinity, by Mr. Silas Stearns, with descriptions of six new species. *Proceedings of the United Stated National Museum* 2:121–156.

Paruğ Ş, and Cengiz Ö. 2020a. The Maximum Length Record of the Blackspot Seabream (*Pagellus bogaraveo* Brünnich, 1768) for the Entire Aegean Sea and Turkish Territorial Waters. *Turkish Journal of Agriculture - Food Science and Technology* 8:2125-2130. DOI 10.24925/turjaf.v8i10.2125-2130.3597.

Paruğ Ş, and Cengiz Ö. 2020b. The Maximum Length Record of the White Seabream (*Diplodus sargus* Linnaeus, 1758) for the Aegean Sea. *Acta Natura et Scientia,* 1:96–108.

### Stephanoberyciformes

Schwartz FJ. 1990. *Acanthochaenus lütkenii* from off North Carolina with additional meristic and morphometric comparisons for Western Atlantic specimens. *Journal of the Elisha Mitchell Scientific Society* 106:51–55.

### Synbranchiformes

Duong T-Y, Tran LVD, Nguyen N-TT, Jamaluddin JAF, and Azizah MNS. 2020. Unravelling taxonomic ambiguity of the Mastacembelidae in the Mekong Delta (Vietnam) through DNA barcoding and morphological approaches. *Tropical Zoology* 33. DOI 10.4081/tz.2020.72.

### Tetraodontiformes

Afrisal M, Nurjirana, Irmawati, and Burhanuddin AI. 2019. Osteological study of Titan Trigger fish, *Balistoides viridescens* (Bloch and Schneider, 1801) (Balistidae: Tetraodontiformes) from the Spermonde Archipelago Waters. *IOP Conference Series: Earth and Environmental Science* 370. DOI 10.1088/1755-1315/370/1/012035.

Alshawy F, Ibrahim A, Hussein C, and Lahlah M. 2019. First record of the oceanic puffer *Lagocephalus lagocephalus* (Linnaeus, 1758) from the Syrian marine waters (eastern Mediterranean). *Marine Biodiversity Records* 12:11. DOI 10.1186/s41200-019-0170-9.

Erguden D, Gurlek M, and Turan C. 2017a. First occurrence of the oceanic puffer, *Lagocephalus lagocephalus* (Linnaeus, 1758) in Iskenderun Bay, north-eastern Mediterranean, Turkey. *Journal of Applied Ichthyology* 33:801–803. DOI https://doi.org/10.1111/jai.13363.

Erguden D, Kabaklı F, Uyan A, Doğdu SA, Karan S, Gurlek M, and Turan C. 2017b. New record of diamondback puffer *Lagocephalus guentheri* Miranda Ribeiro, 1915 from the North-Eastern Mediterranean, Turkey. *Natural and Engineering Sciences* 2:67-73. DOI 10.28978/nesciences.369554.

Hattour A, Nakamura I, and Nguira A. 2004. A large oceanic puffer fish rare in Tunisian waters. *Bulletin de l'Institut National des Sciences et Technologies de Mer de Salammbô* 31:123–125.

Kiparissis S, Peristeraki P, Tampakakis K, Kosoglou I, Doudoumis V, and Batargias C. 2018. Range expansion of a restricted lessepsian: westbound expansion breakthrough of *Lagocephalus spadiceus* (Richardson, 1844) (Actinopterygii: Tetraodontidae). *BioInvasions Records* 7:197–203. DOI 10.3391/bir.2018.7.2.13.

Lipej L, Mavrič B, and Paliska D. 2013. New northernmost record of the blunthead pufferfish, *Sphoeroides pachygaster* (Osteichthyes: Tetraodontidae) in the Mediterranean Sea. *Annales, Series historia naturalis* 23:103-114.

Metin G, and Akyol O. 2020. Maximum size of *Stephanolepis diaspros* (Tetraodontiformes: Monacanthidae). *Marine Science and Technology Bulletin* 10:23–27.

Teker S, Gökoğlu M, and Korun J. 2018. Antalya Körfezi’nde Nadir Bir Balon Balığı Türü; Mavi Balon Balığı *Lagocephalus lagocephalus* (Linnaeus, 1758). *Süleyman Demirel Üniversitesi Eğirdir Su Ürünleri Fakültesi Dergisi* 14:215–219. DOI 10.22392/egirdir.392146.

**Diodontidae**

Aditya Bandyopadhyay S. 2014. On the Record of a Spot-Fin Porcupine Fish, *Diodon hystrix* (Linnaeus, 1758) from Mandarmani, Bay of Bengal Coast of West Bengal, India. *Proceedings of the Zoological Society* 67:175–177. DOI 10.1007/s12595-013-0087-y.

**Molidae**

Babu C, Silambarasan K, Anrose A, and Ramalingam L. 2019. A new record of a rare species *Masturus lanceolatus* (Point-tail sunfish) from Chennai coastal waters, India. *Indian Journal of Geo Marine Sciences* 48:297–301.

Jawad L, Al-Mamry J, and Al-Kharusi L. 2012. First record of *Mola ramsayi* from the Sea of Oman, Sultanate of Oman. *Marine Biodiversity Records* 5:1–4. DOI 10.1017/S1755267212000462.

Yasemi M, and Nazari Bejgan AR. 2014. The first record of southern ocean sunfish, *Mola ramsayi* from Northern Oman Sea, Iran. *Iranian Journal of Fisheries Science* 13:242–246.

## Multiple Groups

Ali AM, Algurabi MA, Nasibulina BM, Kurochkina TF, and Bakhshalizadeh S. 2021. New Records of some fishes from Hadhramout coast, Gulf of Aden, Yemen. *Iranian Journal of Ichthyology* 8. DOI dx.doi.org/10.22034/iji.v8i3.564.

Dey S, Manorama M, and Ramanujam SN. 2015. New records of three species of fish in the upper reaches of the Brahmaputra and Surma-Meghna river basins, Meghalaya, India. *Journal of Threatened Taxa* 7:7922–7926. DOI 10.11609/JoTT.o3297.7922-6.

# Sarcopterygii

Bailes HJ, Trezise AEO, and Collin SP. 2007. The optics of the growing lungfish eye: Lens shape, focal ratio and pupillary movements in Neoceratodus forsteri (Krefft, 1870). *Visual Neuroscience* 24:377–387. DOI 10.1017/S0952523807070381.

Benno B, Verheij E, Stapley J, Rumisha C, Ngatunga B, Abdallah A, and Kalombo H. 2006. Coelacanth (*Latimeria chalumnae* Smith, 1939) discoveries and conservation in Tanzania. *South African Journal of Science* 102:486–490.

De Vos L, and Oyugi D. 2002. First capture of a coelacanth, Latimeria chalumnae Smith, 1939 (Pisces: Latimeriidae), off Kenya: news & views. *South African Journal of Science* 98:345–347.

Erdmann MV, Caldwell RL, Jewett SL, and Tjakrawidjaja A. 1999. The second recorded living coelacanth from north Sulawesi. *Environmental Biology of Fishes* 54:445–451.

Froese R, and Palomares MLD. 2000. Growth, Natural Mortality, Length–weight Relationship, Maximum Length and Length-at-first-maturity of the Coelacanth Latimeria chalumnae. *Environmental Biology of Fishes* 58:45–52. DOI 10.1023/A:1007602613607.

Gunther ACLG. 1871. Description of ceratodus, a genus of ganoid fishes, recently discovered in rivers of Queensland, Australia. *Philosophical Transactions of the Royal Society of London* 161:511-571. DOI 10.1098/rstl.1871.0020.

Saruwatari T, Iwata M, Yabumoto Y, Hukom FD, Peristiwady T, and Abe Y. 2019. A detailed morphological measurement of the seventh specimen of the Indonesian coelacanth, *Latimeria menadoensis*, with a compilation of current morphological data of the species. *Bulletin of the Kitakyushu Museum of Natural History and Human History, Series A (Natural History)* 17:67–80.

# Chondrichthyes

## Carcharhiniformes

**Carcharhinidae**

Baranes A, and Ben-Tuvia A. 1978. Occurence of the sandbar shark *Carcharhinus plumbeus* in the northern Red Sea. *Israel Journal of Zoology* 27:45-51. DOI 10.1080/00212210.1978.10688457.

Baranes A, and Ben-Tuvia A. 1979. Two rare carcharhinids, *Hemipristis elongatus* and *Iago omanensis*, from the northern Red Sea. *Israel Journal of Zoology* 28:39-50. DOI 10.1080/00212210.1979.10688469.

Ben Amor MM, Diatta Y, Diop M, Ben Salem M, and Capapé C. 2016. Confirmed occurrence in the Mediterranean Sea of milk shark *Rhizoprionodon acutus* (Chondrichthyes: Carcharhinidae) and first record off the Tunisian coast. *Cahiers de Biologie Marine* 57:145-149.

Bhagawati D, Nurani T, and Abulias MN. 2018. Jenis, performa, dan nisbah kelamin ikan hiu yang didaratkan di Pelabuhan Perikanan Samudra Cilacap. *Jurnal Iktiologi Indonesia* 17. DOI 10.32491/jii.v17i2.358.

Brum JMM, and Azevedo JMN. 1995. First record of the Galapagos shark *Carcharhinus galapagensis* (Snodgrass and Heller, 1905) (Carcharhinidae) on the Azores. *Boletim do Museu Municipal do Funchal* Supplement No. 4:139-143.

Cho H-G, Kweon S-M, and Kim B-J. 2014. New Record of the Spadenose Shark, *Scoliodon laticaudus* (Carcharhiniformes: Carcharhinidae) from South Sea, Korea. *Korean Journal of Ichthyology* 26:336-339.

Clark E, and Schmidt K, von. 1965. Sharks of the central Gulf Coast of Florida. *Bulletin of Marine Science* 15:13–83.

Compagno LJV, White WT, and Cavanagh RD. 2010. *Glyphis fowlerae* sp. nov., a new species of river shark (Carcharhiniformes; Carcharhinidae) from northeastern Borneo. *CSIRO Marine and Atmospheric Research Paper* 32:29-44.

Compagno LJV, White WT, and Last PR. 2008. *Glyphis garricki* sp. nov., a new species of river shark (Carcharhiniformes: Carcharhinidae) from northern Australia and Papua New Guinea, with a redescription of *Glyphis glyphis* (Müller & Henle, 1839). In: Last PR, White WT, and Pogonoski JJ, eds. *Descriptions of new Australian chondrichthyans*. Hobart: CSIRO Marine and Atmospheric Research, 203-225.

Dolganov VN. 2019. On the Capture of a Blue Shark, *Prionace glauca* (Carcharhinidae), in Peter the Great Bay, Sea of Japan. *Journal of Ichthyology* 59:430–431. DOI 10.1134/S0032945219030044.

Garrick JAF. 1982. Sharks of the genus *Carcharhinus*. *NOAA Technical Report NMFS Circular* 445:1–194.

Garrick JAF. 1985. Additions to a revision of the shark genus *Carcharhinus*: Synonymy of *Aprionodon* and *Hypoprion*, and description of a new species of *Carcharhinus* (Carcharhinidae). *NOAA Technical Report NMFS* 34:1–25.

Hoese HD. 1962. Sharks and rays of Virginia’s seaside bays. *Chesapeake Science* 3:166–172. DOI 10.2307/1350994.

Jensen C, Schwartz FJ, and Hopkins G. 1995. Occurrence of an adult male reef shark, *Carcharhinus perezi* (Carcharhinidae) off North Carolina. *Journal of the Elisha Mitchell Scientific Society* 111:121-125.

Lopes EQ, Paula dos Santos B, de, Chechim de Faria I, Gonçalves de Lima T, and Queiroz de Lucca DS. 2020. Records of occurrences of tiger sharks (*Galeocerdo cuvier*, Peron and Lesueur, 1822) off the coast of Peruíbe - São Paulo-Brazil, APA-CIP and Conservation Units of the Jureia Itatins-SP Mosaic. *Brazilian Journal of Animal and Environmental Research* 3:4270-4282. DOI 10.34188/bjaerv3n4-131.

McKenzie RA, and Tibbo SN. 1964. A Morphometric Description of Blue Shark (*Prionace glauca*) from Canadian Atlantic Waters. *Journal of the Fisheries Research Board of Canada* 21:865-866. DOI 10.1139/f64-081.

Moore ABM, White WT, Ward RD, Naylor GJP, and Peirce R. 2011. Rediscovery and redescription of the smoothtooth blacktip shark, *Carcharhinus leiodon* (Carcharhinidae), from Kuwait, with notes on its possible conservation status. *Marine and Freshwater Research* 62. DOI 10.1071/mf10159.

Nair RV, and Appukuttan KK. 1974. Observations on the developmental stages of the smooth dogfish, *Eridacnis radcliffei* Smith from Gulf of Mannar. *Indian Journal of Fisheries* 21:141-151.

Nakamura J, Yamada M, and Motomura H. 2021. Male and female specimens of the Sliteye Shark *Loxodon macrorhinus* (Carcharhiniformes: Carcharhinidae) from Amami-oshima island, Amami Islands, Satsunan Islands, Kagoshima Prefecture, Japan: the first records from the Satsunan Islands and the northernmost records for the species in the Pacific Ocean. *Ichthy, Natural History of Fishes of Japan* 11:17-20. DOI 10.34583/ichthy.11.0_17.

Peverell SC, McPherson GR, Garrett RN, and Gribble NA. 2006. New records of the River Shark *Glyphis* (Carcharhinidae) reported from Cape York Peninsula, northern Australia. *Zootaxa* 1233:53–68. DOI 10.11646/zootaxa.1233.1.2.

Rafrafi-Nouira S, El Kamel-Moutalibi O, Reynaud C, Boumaïza M, and Capapé C. 2015. Additional and unusual captures of elasmobranch species from the northern coast of Tunisia (central Mediterranean). *Journal of Ichthyology* 55:836–848. DOI 10.1134/S0032945215060181.

Radcliffe L. 1916. The sharks and rays of Beaufort, North Carolina. *Bulletin of the Bureau of Fisheries* 34:239-284.

Randall JE. 1977. Contribution to the biology of the whitetip reef shark (*Triaenodon obesus*). *Pacific Science* 31:143-164.

Sarangdhar. 1943. Tiger shark *Galeocerdo tigrinus* Muller & Henle. Feeding and breeding habits. *Journal of the Bombay Natural History Society* 46:102-110.

Schultz LP, Herald ES, Lachner EA, Welander AD, and Woods LP. 1953. Fishes of the Marshall and Marianas Islands. Volume I: Families from Asymmetronidae through Siganidae. *Bulletin of the United States National Museum* 202:1-685.

Schwartz FJ. 1959. Two Eight-Foot Cub Sharks, *Carcharhinus leucas* (Muller and Henle), Captured in Chesapeake Bay, Maryland. *Copeia* 1959:251–252. DOI 10.2307/1440400.

Schwartz FJ. 1984. Occurrence, Abundance, and Biology of the Blacknose Shark, *Carcharhinus acronotus*, in North Carolina. *Northeast Gulf Science* 7:1-19. DOI 10.18785/negs.0701.02.

Springer VG. 1964. A revision of the carcharhinid genera *Scoliodon*, *Loxodon*, and *Rhizoprionodon*. *Proceedings of the United Stated National Museum* 115:559–632.

Taniuchi T. 1975. Reef whitetip shark, *Triaenodon obesus*, from Japan. *Japanese Journal of Ichthyology* 22:167-170.

Thorburn DC, and Morgan DL. 2004. The northern river shark *Glyphis* sp. C (Carcharhinidae) discovered in Western Australia. *Zootaxa* 685. DOI 10.11646/zootaxa.685.1.1.

Veena S, Thomas S, Raje SG, and Durgekar R. 2011. Case of leucism in the spadenose shark, *Scoliodon laticaudus* (Müller and Henle, 1838) from Mangalore, Karnataka. *Indian Journal of Fisheries* 58:109-112.

Weigmann S. 2012. Contribution to the Taxonomy and Distribution of Six Shark Species (Chondrichthyes, Elasmobranchii) from the Gulf of Thailand. *ISRN Zoology* 2012:1–24. DOI 10.5402/2012/860768.

White WT, Last PR, and Lim APK. 2010a. Rediscovery of the rare and endangered Borneo Shark *Carcharhinus borneensis* (Bleeker, 1858) (Carcharhiniformes: Carcharhinidae). In: Last PR, White WT, and Pogonoski JJ, eds. *Descriptions of New Sharks and Rays from Borneo*. Canberra: CSIRO, 17-28.

White WT, Last PR, and Naylor GJP. 2010b. *Scoliodon macrorhynchos* (Bleeker, 1852), a second species of spadenose shark from the Western Pacific (Carcharhiniformes: Carcharhinidae). In: Last PR, White WT, and Pogonoski JJ, eds. *Descriptions of New Sharks and Rays from Borneo*. Canberra: CSIRO, 61-76.

White WT, Last PR, Naylor GJP, and Harris M. 2010c. Resurrection and redescription of the Borneo Broadfin Shark *Lamiopsis tephrodes* (Fowler, 1905) (Carcharhiniformes: Carcharhinidae). In: Last PR, White WT, and Pogonoski JJ, eds. *Descriptions of New Sharks and Rays from Borneo*. Canberra: CSIRO, 45-59.

Whitley GP. 1943. Ichthyological descriptions and notes. *Proceedings of the Linnean Society of New South Wales* 68:114-144.

Whitley GP. 1945. New sharks and fishes from western Australia. Part 2. *The Australian Zoologist* 11:1-45.

Whitley GP. 1950. A new shark from north-western Australia. *The Western Australian Naturalist* 1-2:100-105.

Yoshino T, Hiramatsu W, Toda M, and Uchida S. 1981. New records of two sharks, *Nebrius concolor* and *Negaprion acutidens*, from Japanese waters. *Bulletin of the College of Science, University of the Ryukyus* 32:37–46.

**Hemigaleidae**

Baranes A, and Ben-Tuvia A. 1979. Two rare carcharhinids, *Hemipristis elongatus* and *Iago omanensis*, from the northern Red Sea. *Israel Journal of Zoology* 28:39-50. DOI 10.1080/00212210.1979.10688469.

Bineesh KK, Sankar RK, Nashad M, Retheesh TB, Kumar RR, and Basheer VS. 2020. Report on Sicklefin weasel shark *Hemigaleus microstoma* (Bleeker, 1852) (Carcharhiniformes: Hemigaleidae) from the Andaman Islands, Indian EEZ with DNA barcodes. *Records of the Zoological Society of India* 120:153-159. DOI 10.26515/rzsi/v120/i2/2020/144516.

Compagno LJV, Krupp F, and Carpenter KE. 1996. A New Weasel Shark of the Genus *Paragaleus* from the Northwestern Indian Ocean and the Arabian Gulf (Carcharhiniformes: Hemigaleidae). *Fauna of Saudi Arabia* 15:391–401.

Chappell A, and Seret B. 2021. Functional Morphology of the Feeding Apparatus of the Snaggletooth Shark, *Hemipristis elongata* (Carcharhiniformes: Hemigaleidae). *Journal of Anatomy* 238:288-307. DOI 10.1111/joa.13313.

Smith JLB. 1957. The rare shark *Hemipristis elongatus* (Klunzinger), 1871, from Zanzibar and Mozambique. *Annals and Magazine of Natural History* 10:555-560. DOI 10.1080/00222935708655997.

Stevens JD, and Cuthbert GJ. 1983. Observations on the Identification and Biology of *Hemigaleus* (Selachii: Carcharhinidae) from Australian Waters. *Copeia* 1983:487–497. DOI 10.2307/1444394.

Tyabji Z, Jabado RW, and Sutaria D. 2018. New records of sharks (Elasmobranchii) from the Andaman and Nicobar Archipelago in India with notes on current checklists. *Biodivers Data J*:e28593. DOI 10.3897/BDJ.6.e28593.

Weigmann S. 2012. Contribution to the Taxonomy and Distribution of Six Shark Species (Chondrichthyes, Elasmobranchii) from the Gulf of Thailand. *ISRN Zoology* 2012:1–24. DOI 10.5402/2012/860768.

**Scyliorhinidae**

Akhilesh KV, Bineesh KK, Mishra SS, Ganga U, and Pillai NGK. 2014. Notes on the Indian swellshark, *Cephaloscyllium silasi* (Scyliorhinidae: Carcharhiniformes) from deep waters off the west coast of India. *Marine Biodiversity Records* 7:E25. DOI 10.1017/s1755267214000141.

Compagno LJV. 1988. *Scyliorhinus comoroensis* sp. n., a new catshark from the Comoro Islands, western Indian Ocean (Carcharhiniformes, Scyliorhinidae). *Bulletin du Muséum National d'Histoire Naturelle, (Série 4), Section A, Zoologie Biologie et Ecologie Animales* 10:603-625.

Compagno LJV, and Stevens JD. 1993a. *Galeus gracilis* n.sp., a New Sawtail Catshark from Australia, with Comments on the Systematics of the Genus *Galeus* Rafinesque, 1810 (Carcharhiniformes: Scyliorhinidae). *Records of the Australian Museum* 45:171–194.

Gomes UL, Peters GO, De Carvalho MR, and Gadig OBF. 2006. Anatomical investigation of the slender catshark *Schroederichthys tenuis* Springer, 1966, with notes on intrageneric relationships (Chondrichthyes: Carcharhiniformes: Scyliorhinidae). *Zootaxa* 1119. DOI 10.11646/zootaxa.1119.1.2.

Hsu HH, Joung SJ, Ebert DA, and Lin CY. 2013. Records of new and rare elasmobranchs from Taiwan. *Zootaxa* 3752:249-255. DOI 10.11646/zootaxa.3752.1.15.

Ito N, Fujii M, Nohara K, and Tanaka S. 2022. *Scyliorhinus hachijoensis*, a new species of catshark from the Izu Islands, Japan (Carcharhiniformes: Scyliorhinidae). *Zootaxa* 5092:331-349. DOI 10.11646/zootaxa.5092.3.5.

Konstantinou H, and Cozzi JR. 1998. *Galeus springeri*, a New Species of Sawtail Catshark from the Caribbean Sea (Chondrichthyes, Scyliorhinidae). *Copeia* 1998:151-158. DOI 10.2307/1447711.

McLaughlin DM, and Morrissey JF. 2004. New Records of Elasmobranchs from the Cayman Trench, Jamaica. *Bulletin of Marine Science* 75:481-485.

Randall JE. 1997. Randall's tank photos. Collection of 10,000 large-format photos (slides) of dead fishes. *Available at www.fishbase.org (see species pages for more details)* (accessed June 29 2022).

Rincon G, and Vooren CM. 2006. Taxonomic and biological records on the south Atlantic marbled catshark, *Galeus mincaronei* Soto, (Elasmobranchii: Scyliorhinidae) off the Southern Brazilian coast *Pan-American Journal of Aquatic Sciences* 1:1-7.

Soares KD, Gadig OF, and Gomes UL. 2015. *Scyliorhinus ugoi*, a new species of catshark from Brazil (Chondrichthyes: Carcharhiniformes: Scyliorhinidae). *Zootaxa* 3937:347-361. DOI 10.11646/zootaxa.3937.2.6.

Taylor LR. 1972. *Apristurus kampae*, a New Species of Scyliorhinid Shark from the Eastern Pacific Ocean. *Copeia* 1972:71-78. DOI 10.2307/1442783.

**Pentanchidae**

Iglésias SP. 2012. *Apristurus nakayai* sp. nov., a new species of deepwater catshark (Chondrichthyes: Pentanchidae) from New Caledonia. *Cybium* 36:511-519.

**Proscyllidae**

Springer S. 1968. *Triakis fehlmanni*, a new shark from the coast of Somalia. *Proceedings of the Biological Society of Washington* 81:613-624.

**Pseudotriakidae**

Allen GR, and Cowan MA. 1995. First record of the false catshark, *Pseudotriakis microdon*, from Australian seas. *Records of the Western Australian Museum* 17:235-236.

Baranes A. 2003. Sharks from the Amirantes Islands, Seychelles, with a description of two new species of squaloids from the deep sea. *Israel Journal of Zoology* 49:33-65.

Ebert DA, Akhilesh KV, and Weigmann S. 2018. *Planonasus indicus* sp. n., a new species of pygmy false catshark (Chondrichthyes: Carcharhiniformes: Pseudotriakidae), with a revised diagnosis of the genus and key to the family. *Marine Biodiversity* 49:1321-1341. DOI 10.1007/s12526-018-0915-4.

Kyne PM, Johnson JW, White WT, and Bennett MB. 2005. First records of the false catshark, *Pseudotriakis microdon* Capello, 1868, from the waters of eastern Australia and Indonesia. *Memoirs of the Queensland Museum* 51:525-530.

Taniuchi T, Kobayashi H, and Otake T. 1984. Occurence and reproductive mode of the false cat shark, *Pseudotriakis microdon*, in Japan. *Japanese Journal of Ichthyology* 31:88-92. DOI 10.11369/jji1950.31.88.

Yano K, and Musick JA. 1992. Comparison of Morphometrics of Atlantic and Pacific Specimens of the False Catshark, *Pseudotriakis microdon*, with Notes on Stomach Contents. *Copeia* 1992:877-886. DOI 10.2307/1446165.

**Sphyrnidae**

Clark E, and Schmidt K, von. 1965. Sharks of the central Gulf Coast of Florida. *Bulletin of Marine Science* 15:13–83.

Rajapackiam S, Balasubramanian KK, Ameer Hamsa KMS, and Kasim HM. 1994. On the landing of large sized hammer head shark *Sphyrna lewini* at Tuticorin. *Marine Fisheries Information Service, Technical and Extension Series* 127:14.

Weigmann S. 2012. Contribution to the Taxonomy and Distribution of Six Shark Species (Chondrichthyes, Elasmobranchii) from the Gulf of Thailand. *ISRN Zoology* 2012:1–24. DOI 10.5402/2012/860768.

**Triakidae**

Clark E, and Schmidt K, von. 1965. Sharks of the central Gulf Coast of Florida. *Bulletin of Marine Science* 15:13–83.

Compagno LJV, and Stevens JD. 1993b. *Hemitriakis falcata* n.sp. and *H. abdita* n.sp., Two New Houndsharks (Carcharhiniformes: Triakidae) from Australia. *Records of the Australian Museum* 45:195–220.

Garrick JAF. 1954. Studies on New Zealand Elasmobranchii. Part III. A New Species of *Triakis* (Selachii) from New Zealand. *Transactions and Proceedings of the Royal Society of New Zealand* 82:695–702.

Kato S. 1968. *Triakis acutipinna* (Galeoidea, Triakidae), a New Species of Shark from Ecuador. *Copeia* 1968:319-325. DOI 10.2307/1441759.

Lowry D, Motta PJ, and Hueter RE. 2007. The ontogeny of feeding behavior and cranial morphology in the leopard shark *Triakis semifasciata* (Girard 1854): A longitudinal perspective. *Journal of Experimental Marine Biology and Ecology* 341:153-167. DOI https://doi.org/10.1016/j.jembe.2006.07.018.

White WT, and Last PR. 2006. Description of two new species of smooth-hounds, *Mustelus widodoi* and *M. ravidus* (Carcharhiniformes: Triakidae) from the western central Pacific. *Cybium* 30:235-246.

Whitley GP. 1943. A new Australian shark. *Records of the South Australian Museum* 7:395-399.

## Chimaeriformes

Araya JF, Reyes P, and Hüne M. 2020. On the presence of the Eastern Pacific Black Ghostshark *Hydrolagus melanophasma* (Chondrichthyes: Chimaeridae) in northern Chile, with notes on its distribution in the Eastern Pacific. *Thalassas: An International Journal of Marine Sciences* 36:565–572. DOI 10.1007/s41208-020-00209-9.

Catarino D, Jakobsen K, Jakobsen J, Giacomello E, Menezes GM, Diogo H, Canha A, Porteiro FM, Melo O, and Stefanni S. 2020. First record of the opal chimaera, *Chimaera opalescens* (Holocephali: Chimaeridae) and revision of the occurrence of the rabbitfish *Chimaera monstrosa* in the Azores waters. *Journal of Fish Biology* 97:763-775. DOI 10.1111/jfb.14432.

Didier DA. 2002. Two new species of chimaeroid fishes from the southwestern Pacific Ocean (Holocephali, Chimaeridae). *Ichthyological Research* 49:299-306. DOI 10.1007/s102280200045.

Didier DA, and Nakaya K. 1999. Redescription of Rhinochimaera pacifica (Mitsukuri) and first record of R. africana Compagno, Stehmann & Ebert from Japan (Chimaeriformes: Rhinochimaeridae) *Ichthyological Research* 46:139–152.

Luchetti EA, Iglésias SP, and Sellos DY. 2011. Chimaera opalescens n. sp., a new chimaeroid (Chondrichthyes: Holocephali) from the north-eastern Atlantic Ocean. *Journal of Fish Biology* 79:399-417. DOI https://doi.org/10.1111/j.1095-8649.2011.03027.x.

Shao K-T, and Hwang D-F. 1997. *Rhinochimaera pacifica*, (Chimaeriformes, Rhinochimaeridae): the first rhinochimaerid recorded from Taiwan. *Acta Zoologica Taiwanica* 8:97–102.

Silas EG, Selvaraj D, and Regunathan A. 1969. Rare chimaeroid and elasmobranch fishes from the continental slope off the west coast of India. *Current Science* 38:105–106.

## Echinorhiniformes

Ángel F, Quinteiro J, and Rey-Méndez M. 2014. First record for the Caribbean Sea of the shark *Echinorhinus brucus* captured in Venezuelan waters. *Marine Biodiversity Records* 7:e91. DOI 10.1017/S1755267214000967.

Garrick JAF. 1960. Studies on New Zealand Elasmobranchii.-Part X. The genus *Echinorhinus*, with account of a second species, *E. cookei* Pietschmann, 1928, from New Zealand Waters. *Transactions and Proceedings of the Royal Society of New Zealand* 88:105-117.

Lee W-J, and Kim J-K. 2018. First Reliable Record of *Echinorhinus cookei* (Chondrichthyes: Elasmobranchii) Collected from Busan, Korea. *Korean Journal of Fisheries and Aquatic Sciences* 51:595-599. DOI 10.5657/KFAS.2018.0595.

Musick JA, and McEachran JD. 1969. The squaloid shark *Echinorhinus brucus* off Virginia. *Copeia* 1:205–206.

Nair RV, and Lal Mohan RS. 1971. On the occurence of the spiny shark *Echinorhinus brucus* (Bonnaterre) from the east coast of India with a note on its distribution. *Indian Journal of Animal Science* 41:1011-1014.

Schwartz FJ. 1993. A North Carolina capture of the bramble shark, *Echinorhinus brucus*, family Echinorhinidae, the fourth in the western Atlantic. *Journal of the Elisha Mitchell Scientific Society* 109:158–162.

Silas EG, Selvaraj D, and Regunathan A. 1969. Rare chimaeroid and elasmobranch fishes from the continental slope off the west coast of India. *Current Science* 38:105–106.

Varojean DH. 1972. Systematics of the genus *Echinorhinus* Blainville, based on a study of the prickly shark *Echinorhinus cookei* Pietschmann M. A. Fresno State College.

## Lamniformes

**Alopiidae**

Aneesh Kumar KV, Paresh Khanolkar S, Pravin P, Meenakumari B, and Radhakrishnan EV. 2012. First record of the pelagic thresher shark *Alopias pelagicus* (Pisces: Alopiiformes: Alopiidae) from the Lakshadweep Sea, India. *Marine Biodiversity Records* 5:e16. DOI 10.1017/S1755267211001114.

Bigelow HB, and Schroeder WC. 1948. Sharks. In: Tee-Van J, Breder CM, Hildebrand SF, Parr AE, and Schroeder WC, eds. *Fishes of the Western North Atlantic, Lancelets, Cyclostomes, and Sharks*. New Haven: Yale University Press, 59-546.

Choi Y, Kim I-S, and Nakaya K. 1997. New records of Sharks, *Sphyrna lewini* and *Alopias vulpinus* (Pisces: Elasmobranchii) in Korea. *Journal of Animal Systematics, Evolution, Diversity* 13:285–290.

Compagno LJV, and Smale MJ. 1986. Recent records of four warm-water elasmobranchs from the eastern Cape Province, South Africa. *South African Journal of Marine Science* 4:11–15. DOI 10.2989/025776186784461873.

Ergüden D, Gürlek M, and Turan C. 2015. Occurrence of the Thresher *Alopias vulpinus* (Bonnaterre, 1788) from the Northeastern Mediterranean coast of Turkey. *Biharean Biologist* 9:76-77.

Gruber SH, and Compagno LJV. 1981. Taxonomic status and biology of the bigeye thresher, *Alopias superciliosus*. *Fishery Bulletin* 79:617–640.

Nakamura H. 1935. On the two species of the thresher shark from Formosan waters. *Memoirs of the Faculty of Science and Agriculture, Taihoku Imperial University* 14:1-6.

**Cetorhinidae**

Ahnelt H, Sauberer M, Ramler D, Koch L, and Pogoreutz C. 2020. Negative allometric growth during ontogeny in the large pelagic filter-feeding basking shark. *Zoomorphology* 139:71–83. DOI 10.1007/s00435-019-00464-2.

Ali M, Saad A, Reynaud C, and Capapé C. 2012. Occurrence of basking shark, *Cetorhinus maximus* (Elasmobranchii: Lamniformes: Cetorhinidae), off the Syrian coast (eastern Mediterranean) with first description of egg case. *Acta Ichthyologica et Piscatoria* 42:335-339. DOI 10.3750/aip2012.42.4.07.

Gudger EW. 1948. The basking shark, *Cetorhinus maximus*, on the North Carolina coast. *Journal of the Elisha Mitchell Scientific Society* 64:41–44.

**Lamnidae**

Applegate SP. 1966. A possible record-size bonito shark, *Isurus oxyrinchus* Rafinesque, from southern California. *California Fish and Game* 52:204-207.

Applegate SP. 1977. A new record-size bonito shark, *Isurus oxyrinchus* Rafinesque, from southern California. *California Fish and Game* 63:126-129.

De Maddalena A, Glaizot O, and Olivier G. 2003. On the Great White Shark, *Carcharodon carcharias* (Linnaeus, 1758), preserved in the Museum of Zoology in Lausanne. *Marine Life* 13:53-59.

Dolganov VN. 2012. The capture of a great white shark *Carcharodon carcharias* Linnaeus, 1758 (Carcharodontidae) in peter the Great Bay (Sea of Japan). *Russian Journal of Marine Biology* 38:88–90. DOI 10.1134/S106307401201004X.

Garrick JAF. 1967. Revision of sharks of genus *Isurus* with description of a new speces (Galeoidea, Lamnidae). *Proceedings of the United Stated National Museum* 118:663–690.

Kabaskal H, and Kabaskal Ö. 2013. First record of a shortfin mako shark, *Isurus oxyrinchus* Rafinesque, 1810 (Chondrichthyes: Lamnidae) from the Bay of Saroz (NE Aegean Sea). *Annales, Series historia naturalis* 23:27–32.

Lipej L, Uhan J, Mavrič B, and Vujčić-Karlo S. 2016. A record of porbeagle, *Lamna nasus* (Bonnaterre, 1788), in the Gulf of Trieste with discussion on its occurrence in the Adriatic Sea. *Acta Adriatica: International Journal of Marine Sciences* 57:305–313.

McKenzie RA, and Tibbo SN. 1964. A Morphometric description of Porbeagle (*Lamna nasus*) from Canadian Atlantic waters. *Journal Fisheries Research Board of Canada* 21:863-864.

Mignucci-Giannoni AA, Cintrón-Nieves D, Franqui-Rivera G, Espinoza R, Orcera-Iglesias JM, Rivera-Ilarraza PJ, Rivera-Pérez CI, and Rodríguez-Ferrer G. 2020. First Confirmed Record of the Longfin Mako Shark (*Isurus paucus*) for Puerto Rico. *Caribbean Naturalist* 71:1-9.

Nakaya K. 1971. Descriptive notes on a porbeagle, *Lamna nasus*, from Argentine waters, compared with the North Pacific salmon shark, *Lamna ditropis*. *Bulletin of the Faculty of Fisheries, Hokkaido University* 21:269–279.

Pratt HL, Jr., Casey JG, and Conklin RB. 1982. Observations on large white sharks, *Carcharodon carcharias*, off Long Island, New York. *Fishery Bulletin* 80:153–156.

Springer S. 1939. The Great White Shark, *Carcharodon carcharias* (Linnaeus), in Florida Waters. *Copeia* 1939:114–115. DOI 10.2307/1435964.

Skud BE. 1962. Measurements of a White Shark, *Carcharodon carcharias*, Taken in Maine Waters. *Copeia* 1962:659–661. DOI 10.2307/1441208.

Stevens JD, Dunning MC, and Machida S. 1983. Occurence of the Porbeagle Shark, *Lamna nasus*, in the Tasman Sea. *Japanese Journal of Ichthyology* 30:301-307.

Tibbo SN, McKenzie RA, and Scott WB. 1963. An occurence of mako sharks, *Isurus oxyrinchus*, Rafinesque 1810, in the Canadian Arctic. *Journal Fisheries Research Board of Canada* 20:1353.

**Mitsukuriidae**

Duffy CAJ. 1997. Further records of the goblin shark, *Mitsukurina owstoni* (Lamniformes: Mitsukurinidae), from New Zealand. *New Zealand Journal of Zoology* 24:167-171. DOI 10.1080/03014223.1997.9518111.

Lee P-F, and Shao K-T. 2009. Two new records of lamniform shark from the waters adjacent to Taiwan. *Journal Of The Fisheries Society Of Taiwan* 36:303-311.

Rincon G, Vaske T, and Gadig OBF. 2012. Record of the goblin shark *Mitsukurina owstoni* (Chondrichthyes: Lamniformes: Mitsukurinidae) from the south-western Atlantic. *Marine Biodiversity Records* 5:E44. DOI 10.1017/s1755267211000923.

Yano K, Miya M, Aizawa M, and Noichi T. 2007. Some aspects of the biology of the goblin shark, *Mitsukurina owstoni*, collected from the Tokyo Submarine Canyon and adjacent waters, Japan. *Ichthyological Research* 54:388-398. DOI 10.1007/s10228-007-0414-2.

**Megachasmidae**

Berra TM, and Hutchins JB. 1990. A specimen of megamouth shark, *Megachasma pelagios* (Megachasmidae) from Western Australia. *Records of the Western Australian Museum* 14:651–656.

Lee P-F, and Shao K-T. 2009. Two new records of lamniform shark from the waters adjacent to Taiwan. *Journal Of The Fisheries Society Of Taiwan* 36:303-311.

Sawamoto S, and Matsumoto R. 2012. Stomach contents of a megamouth shark *Megachasma pelagios* from the Kuroshio Extension: evidence for feeding on a euphausiid swarm. *Plankton and Benthos Research* 7:203-206.

Taylor LR, Compagno LJV, and Struhsaker PJ. 1983. Megamouth - a new species, genus, and family of lamnoid shark (*Megachasma pelagios*, family Megachasmidae) from the Hawaiian Islands. *Proceedings of the California Academy of Science* 43:87-110.

Yano K, Yabumoto Y, Tanaka S, Tsukada O, and Furuta M. 1997. Capture of a mature female megamouth shark, *Megachasma pelagios*, from Mie, Japan. *Proceedings of the 5th Indo-Pacific Conference, Nouméa*:335–349.

**Odontaspidae**

Anguila R, Nieto Alvarado LE, and Hernández Beracasa L. 2016. Nuevos registros de peces de esqueleto cartilaginoso para el Caribe colombiano y uno como ampliación de su distribución geográfica en el Caribe colombiano para Bocas de Ceniza, Departamento de Atlántico, Colombia. *Bulletin of Marine and Coastal Research* 45:361–373. DOI 10.25268/bimc.invemar.2016.45.2.692.

Bonfil R. 1995. Is the ragged-tooth shark cosmopolitan? First record from the North Atlantic. *Journal of Fish Biology* 47:341-344.

Estupiñán-Montaño C, Galván-Magaña F, Hacohen-Domené A, and Estupiñán-Ortíz JF. 2016. First reports of smalltooth sand tiger sharks, *Odontaspis ferox* (Elasmobranchii: Lamniformes: Odontaspididae), off the continental Ecuador. *Acta Ichthyologica et Piscatoria* 46:251-253. DOI 10.3750/aip2016.46.3.09.

Radcliffe L. 1916. The sharks and rays of Beaufort, North Carolina. *Bulletin of the Bureau of Fisheries* 34:239-284.

Sadowsky V, Amorim AFd, and Arfelli CA. 1984. Second occurence of *Odontapsis noronhai* (Maul, 1955). *Boletim do Instituto de Pesca* 11:69–79.

Stone NR, and Shimada K. 2019. Skeletal Anatomy of the Bigeye Sand Tiger Shark, *Odontaspis noronhai* (Lamniformes: Odontaspididae), and Its Implications for Lamniform Phylogeny, Taxonomy, and Conservation Biology. *Copeia* 107:632-652. DOI 10.1643/CG-18-160.

**Pseudocarchariidae**

Kizhakudan SJ, and Rajapackiam S. 2013. First report of the crocodile shark *Pseudocarcharias kamoharai* (Matsubara,1936)from Chennai, southeast coast of India. *Journal of the Marine Biological Association of India* 55:86–88. DOI 10.6024/jmbai.2013.55.1.01734-14.

Pradeep HD, Swapnil SS, Ramachandran S, and Pattnayak SK. 2016. Report of the crocodile shark *Pseudocarcharias kamoharai* (Matsubara, 1936) from deep waters of the Andaman Sea. *Marine Biodiversity* 47:535–538. DOI 10.1007/s12526-016-0499-9.

Randall JE. 1997. Randall's tank photos. Collection of 10,000 large-format photos (slides) of dead fishes. *Available at www.fishbase.org (see species pages for more details)* (accessed June 29 2022).

Stewart AL. 2001. First record of the crocodile shark, *Pseudocarcharias kamoharai* (Chondrichthyes: Lamniformes), from New Zealand waters. *New Zealand Journal of Marine and Freshwater Research* 35:1001–1006. DOI 10.1080/00288330.2001.9517059.

## Hexanchiiformes

Anguila R, Alvarado LEN, Barandica JCN, Beracasa LH, and Acero P. A. 2016a. Ampliación geográfica del tiburón siete branquias o bocadulce *Heptranchias perlo* Bonnaterre (Hexanchiformes: Hexanchidae) para el Caribe continental Columbiano. *Boletin de Investigaciones Marinas y Costeras* 45:355-360.

Anguila R, Nieto Alvarado LE, and Hernández Beracasa L. 2016. Nuevos registros de peces de esqueleto cartilaginoso para el Caribe colombiano y uno como ampliación de su distribución geográfica en el Caribe colombiano para Bocas de Ceniza, Departamento de Atlántico, Colombia. *Bulletin of Marine and Coastal Research* 45:361–373. DOI 10.25268/bimc.invemar.2016.45.2.692.

Avalos-Castillo CG, Santana-Morales O, Becerril-García EE, and Areano E. 2020. New records and morphometry of the Atlantic sixgill shark *Hexanchus vitulus* in the Caribbean coast of Guatemala. *Latin American Journal of Aquatic Research* 48:488-491. DOI 10.3856/vol48-issue3-fulltext-2436.

Capapé C, Rafrafi-Nouira S, Ben Amor KO, and Ben Amor MM. 2018. Unusual and substantiated records of *Heptranchias perlo* (Chondrichthyes: Hexanchidae) in the N-E Tunisian waters (Central Mediterranean Sea). *Thalassia Salientia* 40:17-24. DOI 10.1285/i15910725v40p17.

Ebert DA, White WT, and Ho HC. 2013. Redescription of *Hexanchus nakamurai* Teng 1962, (Chondrichthyes: Hexanchiformes: Hexanchidae), with designation of a neotype. *Zootaxa* 3752:20-34. DOI 10.11646/zootaxa.3752.1.4.

Garrick JAF, and Paul LJ. 1971. *Heptranchias dakini* Whitley, 1931, a synonym of *H. perlo* (Bonnaterre, 1788), the sharpnose sevengill or Perlon shark, with notes on sexual dimorphism in this species. *Zoology Publications from Victoria University of Wellington* 55:1–14.

Imai C, Ikeda I, and Sakai H. 1995. A record of the rare broadnose sevengill shark *Notorynchus cepedianus* off Yamaguchi in the sea of Japan. *Journal of the National Fisheries University* 53:35-40.

Kumar R, S V, K. V A, K. K B, and P. T R. 2018. First report of four deep-sea chondrichthyans (Elasmobranchii and Holocephali) from Andaman waters, India with an updated checklist from the region. *Acta Ichthyologica et Piscatoria* 48:289-301. DOI 10.3750/aiep/02336.

Kukuev EI, and Pavlov VP. 2008. The first case of mass catch of a rare frill shark *Chlamydoselachus anguineus* over a seamount of the Mid-Atlantic Ridge. *Journal of Ichthyology* 48:676–678. DOI 10.1134/S0032945208080158.

McLaughlin DM, and Morrissey JF. 2004. New Records of Elasmobranchs from the Cayman Trench, Jamaica. *Bulletin of Marine Science* 75:481-485.

Mili S, Ghanem R, Ennouri R, Troudi D, Zarrouk H, and Jabbari S. 2021. Biological aspects of the Bluntnose Sixgill Shark, *Hexanchus* *griseus* (Bonnaterre, 1788) in Tunisian waters: implications for fishery management. *Journal of New Sciences* 15:333-345.

Nakaya K, and Bass AJ. 1978. The frill shark *Chlamydoselachus anguineus* in New Zealand seas. *New Zealand Journal of Marine and Freshwater Research* 12:397–398. DOI 10.1080/00288330.1978.9515767.

Rafrafi-Nouira S, El Kamel-Moutalibi O, Reynaud C, Boumaïza M, and Capapé C. 2015. Additional and unusual captures of elasmobranch species from the northern coast of Tunisia (central Mediterranean). *Journal of Ichthyology* 55:836–848. DOI 10.1134/S0032945215060181.

Sadowsky V. 1970. First record of broad-snouted seven-gilled shark from Cananéia, coast of Brazil. *Boletim da Inst Oceanogr Sao Paulo* 18:33–35.

Scott EOG. 1982. Dimensions and form of a seven-gilled shark, *Notorynchus cepedianus* (Péron) from the Tamar estuary, Tasmania. *Records of the Queen Victoria Museum Launceston* 76:1-14.

Springer S, and Waller RA. 1969. *Hexanchus vitulus*, a New Sixgill Shark from the Bahamas. *Bulletin of Marine Science* 19:159-174.

## Heterodontiformes

**Heterodontidae**

Baldwin ZH. 2005. A New Species of Bullhead Shark, Genus *Heterodontus* (Heterodontiformes: Heterodontidae), from Oman. *Copeia* 2005:262–264. DOI 10.1643/ci-04-168r1.

## Orectolobiformes

**Ginglymostomidae**

Fernando Del Moral-Flores L, Ramírez-Antonio E, Angulo A, and Pérez-Ponce de León G. 2015. Ginglymostoma unami sp. nov. (Chondrichthyes: Orectolobiformes: Ginglymostomatidae): una especie nueva de tiburón gata del Pacífico oriental tropical. *Revista Mexicana de Biodiversidad* 86:48-58. DOI 10.7550/rmb.46192.

Taniuchi T, and Yanagisawa F. 1987. Albinism and lack of second dorsal fin in an adult tawny nurse shark, *Nebrius concolor*, from Japan. *Japanese Journal of Ichthyology* 34:393–395.

Yoshino T, Hiramatsu W, Toda M, and Uchida S. 1981. New records of two sharks, *Nebrius concolor* and *Negaprion acutidens*, from Japanese waters. *Bulletin of the College of Science, University of the Ryukyus* 32:37–46.

**Hemiscyllidae**

Allen GR, Erdmann MV, and Dudgeon CL. 2013. *Hemiscyllium halmahera*, a new species of Bamboo Shark (Hemiscylliidae) from Indonesia. *Aqua, International Journal of Ichthyology* 19:123–136.

Devadoss P. 1986. Studies on the catshark *Chiloscyllium griseum* from Indian waters. *Journal of the Marine Biological Association of India* 28:192–198.

Weigmann S. 2012. Contribution to the Taxonomy and Distribution of Six Shark Species (Chondrichthyes, Elasmobranchii) from the Gulf of Thailand. *ISRN Zoology* 2012:1–24. DOI 10.5402/2012/860768.

**Orectolobidae**

Last PR, Chidlow JA, and Compagno LJV. 2006. A new wobbegong shark, *Orectolobus hutchinsi* n. sp. (Orectolobiformes: Orectolobidae) from southwestern Australia. *Zootaxa* 1239:35–48. DOI 10.11646/zootaxa.1239.1.3.

Last PR, Pogonoski JJ, and White WT. 2010. A new wobbegong shark, *Orectolobus leptolineatus* sp. nov. (Orectolobiformes: Orectolobidae), from the Western Central Pacific. In: Last PR, White WT, and Pogonoski JJ, eds. *Descriptions of New Sharks and Rays from Borneo*. Canberra: CSIRO, 1-16.

**Rhincodontidae**

Gudger EW. 1931. The fourth florida whale shark, *Rhineodon typus*, and the American Museum model based on it. *Bulletin of the American Museum of Natural History* 41:613–656.

Kasinathan C, Muniyandi K, Bose M, and Gandhi A. 2002. Observations on whale shark *Rhineodon typus* (Smith) caught at Pamban Palk Bay and Gulf of Mannar. *Marine Fisheries Information Service, Technical and Extension Series* 174:1015-1016.

Tomita T, Kawai T, Matsubara H, Kobayashi M, and Katakura S. 2014. Northernmost record of a whale shark *Rhincodon typus* from the Sea of Okhotsk. *Journal of Fish Biology* 84:243-246. DOI https://doi.org/10.1111/jfb.12273.

**Stegostomatidae**

Dahl RB, Sigsgaard EE, Mwangi G, Thomsen PF, Jørgensen RD, de Oliveira Torquato F, Olsen L, and Møller PR. 2019. The Sandy Zebra Shark: A New Color Morph of the Zebra Shark *Stegostoma tigrinum*, with a Redescription of the Species and a Revision of Its Nomenclature. *Copeia* 107:524–541. DOI 10.1643/CG-18-115.

## Rhinopristiformes

Compagno LJV, and Last PR. 2010. A new species of wedgefish, *Rhynchobatus springeri* (Rhynchobatoidei, Rhynchobatidae), from the Western Pacific. In: Last PR, White WT, and Pogonoski JJ, eds. *Descriptions of New Sharks and Rays from Borneo*: CSIRO, 77-88.

Koeda K, Itou M, Yamada M, and Motomura H. 2020. *Rhynchobatus mononoke*, a new species of wedgefish (Rhinopristiformes: Rhinidae) from Japan, with comments on *Rhynchobatus laevis* (Bloch and Schneider 1801). *Ichthyological Research* 68:223-238. DOI 10.1007/s10228-020-00777-z.

Rafrafi-Nouira S, El Kamel-Moutalibi O, Reynaud C, Boumaïza M, and Capapé C. 2015. Additional and unusual captures of elasmobranch species from the northern coast of Tunisia (central Mediterranean). *Journal of Ichthyology* 55:836–848. DOI 10.1134/S0032945215060181.

Wilson L, Zacharia PU, Kizhakudan SJ, Najmudeen TM, Santhosh B, Gop AP, Radhakrishnan M, Sunil KTS, and Pakkri Muthu S. 2020. Report on the landing of the critically endangered Rhinopterid *Acroteriobatus variegatus* (Nair & Lal Mohan, 1973) with some insights into its reproductive biology. *Journal of the Marine Biological Association of India* 62:60–64. DOI 10.6024/jmbai.2020.62.1.2145-07.

## Squaliformes

Baranes A. 2003. Sharks from the Amirantes Islands, Seychelles, with a description of two new species of squaloids from the deep sea. *Israel Journal of Zoology* 49:33-65.

Mas F, Forselledo R, Domingo A, Pin O, Troncoso P, Errico E, Marquez A, Tanaka S, and Weigmann S. 2020. New records and range extension of the Portuguese dogfish *Centroscymnus coelolepis* in the south-western Atlantic Ocean, with comments on its morphology. *Journal of Fish Biology* 96:601-616. DOI https://doi.org/10.1111/jfb.14246.

**Centrophoridae**

Diatta Y, Diaby A, Rafrafi-Nouira S, and Capapé C. 2019. Capture of a rare endangered species leafscale gulper shark *Centrophorus squamosus* (Chondrichthyes: Squalidae) from the coast of Senegal (eastern tropical Atlantic). *Annales, Series historia naturalis* 29:205-210. DOI 10.19233/ASHN.2019.20.

Duffy CAJ. 2007. First record of *Centrophorus harrissoni* from New Zealand, with observations on squamation in Centrophoridae (Squaliformes). *New Zealand Journal of Marine and Freshwater Research* 41:163-173. DOI 10.1080/00288330709509905.

McLaughlin DM, and Morrissey JF. 2004. New Records of Elasmobranchs from the Cayman Trench, Jamaica. *Bulletin of Marine Science* 75:481-485.

Pradeep HD, Shirke SS, Nashad M, and Sukham MD. 2017. A first record of the Smallfin Gulper Shark *Centrophorus moluccensis* Bleeker, 1860 (Chondrichthyes: Squaliformes: Centrophoridae) from the Andaman & Nicobar waters. *Journal of Threatened Taxa* 9:10899–10903. DOI 10.11609/jott.3315.9.11.10899-109030.

White WT, Ebert DA, Naylor GJ, Ho HC, Clerkin P, Verissimo A, and Cotton CF. 2013. Revision of the genus *Centrophorus* (Squaliformes: Centrophoridae): Part 1--Redescription of *Centrophorus granulosus* (Bloch & Schneider), a senior synonym of *C*. *acus* Garman and *C*. *niaukang* Teng. *Zootaxa* 3752:35-72. DOI 10.11646/zootaxa.3752.1.5.

Wienerroither RM, Bjelland O, Bachmann L, and Junge C. 2015. Northernmost record of the little gulper shark *Centrophorus uyato* in the north-eastern Atlantic Ocean, with taxonomical notes on Centrophorus zeehaani. *J Fish Biol* 86:834-844. DOI 10.1111/jfb.12602.

**Dalatiidae**

Da Silva AA, Duarte PC, Giga A, and Menezes G. 1998. First record of the spined pygmy shark, *Squaliolus laticaudus* (Smith & Radcliffe, 1912) in the Azores, extending its distribution in the north-eastern Atlantic. *Arquipélago* 16A:57-61.

Driggers WB, III, Burgess GH, Hamilton AN, Jr., Hopkins NM, and Schobernd CM. 2010. *Squaliolus laticaudus* in the Western North Atlantic Ocean: Distributional and Life History Observations. *Bulletin of Marine Science* 86:831-838. DOI 10.5343/bms.2010.1009.

Garrick JAF, and Springer S. 1964. *Isistius plutodus*, a New Squaloid Shark from the Gulf of Mexico. *Copeia* 1964:678-682. DOI 10.2307/1441443.

Hulley PA, and Penrith MJ. 1966. *Euprotomicroides zantedeschia*, a new genus and specise of pigmy dalatiid shark from South Africa. *Bulletin of Marine Science* 16:222–229.

Jahn AE, and Haedrich RL. 1988. Notes on the pelagic squaloid shark *Isistius brasiliensis*. *Biological Oceanography* 5:297–309. DOI 10.1080/01965581.1987.10749519.

Randall JE. 1997. Randall's tank photos. Collection of 10,000 large-format photos (slides) of dead fishes. *Available at www.fishbase.org (see species pages for more details)* (accessed June 29 2022).

Stehlmann MFW, Van Oijen M, and Kamminga P. 2016. Re-description of the rare taillight shark *Euprotomicroides zantedeschia* (Squaliformes, Dalatiidae), based on third and fourth record from off Chile *Cybium* 40:187–197.

Tagliafico A, Rago N, and Ron E. 2007. Primer reporte de tiburón *Dalatias licha* (Bonnaterre, 1788) (Elasmobranchii: Squaliformes: Dalatiidae) para Venezuela y el Mar Caribe. *Boletín del Instituto Oceanográfico de Venezuela* 46:113–117.

**Etmopteridae**

Shirai S, and Nakaya K. 1990. A New Squalid Species of the Genus *Centroscyllium* from the Emperor Seamount Chain. *Japanese Journal of Ichthyology* 36:391-398.

Wetherbee BM, and Kaijura SM. 2000. Occurrence of a rare squaloid shark, *Trigonognathus kabeyai*, from the Hawaiian islands. *Pacific Science* 54:389-394.

Więcaszek B, Sobecka E, Panicz R, Keszka S, Górecka K, and Linowska A. 2018. First record of the deep-water shark *Etmopterus spinax* (Chondrichthyes: Etmopteridae) from the southern Baltic Sea (Pomeranian Bay). *Oceanologia* 60:426–430. DOI <https://doi.org/10.1016/j.oceano.2018.02.001>.

Yano K, Mochizuki K, Tsukada O, and Suzuki K. 2003. Further description and notes of natural history of the viper dogfish, *Trigonognathus kabeyai* from the Kumano-nada Sea and the Ogasawara Islands, Japan (Chondrichthyes: Etmopteridae). *Ichthyological Research* 50:251-258. DOI 10.1007/s10228-003-0165-7.

**Oxynotidae**

Kousteni V, and Megalofonou P. 2016. Observations on the biological traits of the rare shark Oxynotus centrina (Chondrichthyes: Oxynotidae) in the Hellenic Seas. *Journal of Fish Biology* 89:1880–1888. DOI https://doi.org/10.1111/jfb.13077.

Viana S, and Lisher MW. 2018. On the taxonomy of the first record of rare deep-water rough shark species of Oxynotidae (Chondrichthyes: Squaliformes) in the western Indian Ocean. *Journal of Threatened Taxa* 10. DOI 10.11609/jott.3916.10.6.11732-11742.

Yano K, Matsuura K, and Tsukada O. 2002. Redescription of the Rare Squaloid Shark *Oxynotus japonicus* from Suruga Bay and the Enshu-nada Sea, Japan. *Species Diversity* 7:363-369.

**Somniosidae**

Benz GW, Hocking R, Kowunna A, Bullard SA, and George JC. 2004. A second species of Arctic shark: Pacific sleeper shark *Somniosus pacificus* from Point Hope, Alaska. *Polar Biology* 27:250-252. DOI 10.1007/s00300-003-0589-6.

Capapé C, Rafrafi-Nouira S, Diatta Y, and Christian R. 2020. First record of little sleeper shark, *Somniosus rostratus* (Elasmobranchii: Squaliformes: Somniosidae), from the Tunisian coast, central Mediterranean Sea. *Acta Ichthyologica et Piscatoria* 50:475-480. DOI 10.3750/aiep/02998.

Ebert DA, Compagno LJV, and Natanson LJ. 1987. Biological Notes on the Pacific Sleeper Shark, *Somniosus pacificus* (Chondrichthyes, Squalidae). *California Fish and Game* 73:117-123.

Ebert DA, Knuckey JDS, and Kamikawa DJ. 2014. First eastern North Pacific record of the velvet dogfish, *Zameus squamulosus* (Chondrichthyes: Squaliformes: Somniosidae). *Marine Biodiversity Records* 7. DOI 10.1017/s1755267214000499.

Francis MP, Stevens JD, and Last PR. 1988. New records of *Somniosus* (Elasmobranchii: Squalidae) from Australasia, with comments on the taxonomy of the genus. *New Zealand Journal of Marine and Freshwater Research* 22:401-409. DOI 10.1080/00288330.1988.9516311.

Hsu HH, Lin CY, and Joung SJ. 2020. *Somniosus* (*Rhinoscymnus*) *cheni* sp. nov., A New Species of Sleeper Shark (Squaliformes: Somniosidae) from Eastern Taiwan, with Aspects of Embryo Biology. *Zoological Studies* 59:e48. DOI 10.6620/ZS.2020.59-48.

Navarro SJ, Alayón JP, López Abellán LJ, Holtzhausen JA, and Jiménez JFG. 2011. Seamount associated species (fishes, crustaceans and cephalopods) Namibia-0802. *Preliminary report of the multidisciplinary research cruise on the Walvis Ridge seamounts (Atlantic Southeast-SEAFO)*. Madrid: Instituto Español de Oceanografía/National Marine Information and Research Centre, 98-191.

Wetherbee BM, and Crow GL. 1996. First record of the squaloid shark *Scymnodon squamulosus* from the Hawaiian Islands. *Ichthyological Research* 43:334–339.

White WT, Vaz DFB, Ho H-C, Ebert DA, de Carvalho MR, Corrigan S, Rochel E, de Carvalho M, Tanaka S, and Naylor GJP. 2015. Redescription of *Scymnodon ichiharai* Yano and Tanaka 1984 (Squaliformes: Somniosidae) from the western North Pacific, with comments on the definition of somniosid genera. *Ichthyological Research* 62:213-229. DOI 10.1007/s10228-014-0430-y.

**Squalidae**

Baranes A. 2003. Sharks from the Amirantes Islands, Seychelles, with a description of two new species of squaloids from the deep sea. *Israel Journal of Zoology* 49:33-65.

Daly-Engel TS, Koch A, Anderson JM, Cotton CF, and Rubbs RD. 2018. Description of a new deep-water dogfish shark from Hawaii, with comments on the Squalusmitsukurii species complex in the West Pacific. *ZooKeys*:135-157. DOI 10.3897/zookeys.798.28375.

Ebert DA, Cowley PD, and Compagno LJV. 2002. First records of the longnose spiny dogfish *Squalus blainvillei* (Squalidae) and the deep-water stingray *Plesiobatis daviesi* (Urolophidae) from South African waters. *African Journal of Marine Science* 24:355–357.

Ebert DA, White WT, Goldman KJ, Compagno LJV, Daly–Engel TS, and Ward RD. 2010. Resurrection and redescription of *Squalus suckleyi* (Girard, 1854) from the North Pacific, with comments on the *Squalus acanthias* subgroup (Squaliformes: Squalidae). *Zootaxa* 2612. DOI 10.11646/zootaxa.2612.1.2.

Garrick JAF, and Paul LJ. 1971a. *Cirrhigaleus barbifer* (Fam. Squalidae), a little known Japanese shark from New Zealand waters. *Zoology Publications from Victoria University of Wellington* 55:1–13.

Kempster RM, Hunt DM, Human BA, Egeberg CA, and Collin SP. 2013. First record of the mandarin dogfish *Cirrhigaleus barbifer* (Chondrichthyes: Squalidae) from Western Australia. *Marine Biodiversity Records* 6:e25. DOI 10.1017/S175526721300002X.

Last PR, Edmunds M, and Yearsley GK. 2007. Part 2 - *Squalus crassispinus* sp nov., a new spurdog of the *megalops*-*cubensis* group from the eastern Indian Ocean. *CSIRO Marine and Atmospheric Research Paper* 14:11–22.

Last PR, White WT, and Motomura H. 2007. Part 6 - Description of *Squalus chloroculus* sp. nov., a new spurdog from southern Australia, and the resurrection of *S. montalbani* Whitley. *CSIRO Marine and Atmospheric Research Paper* 14:55-69.

Orozco-Velasquez DM, Gomez-Delgado F, and Salcedo-Reyes JC. 2016. New record of *Squalus cubensis* Howell Rivero, 1936 (Chondrichthyes, Squalidae) in Colombia. *Universitas Scientarum* 21:159–166.

McGrouther M. 2019. Portuguese Dogfish, *Centroscymnus coelolepis* Bocage & Capello, 1864. *Available at https://australian.museum/learn/animals/fishes/portuguese-dogfish-centroscymnus-coelolepis-bocage-capello-1864/* (accessed July 20 2019).

McLaughlin DM, and Morrissey JF. 2004. New Records of Elasmobranchs from the Cayman Trench, Jamaica. *Bulletin of Marine Science* 75:481-485.

Silas EG, and Prasad NK. 1969. On the occurence of the deep-water squaloid shark *Squalus fernandinus* Molina from the Continental slope off the west coast of India. *Current Science* 38:1-3.

White WT, Last PR, and Stevens JD. 2007. *Cirrhigaleus australis* n. sp., a new Mandarin dogfish (Squaliformes: Squalidae) from the south-west Pacific. *Zootaxa* 1560:19-30. DOI 10.11646/zootaxa.1560.1.2.

## Squatiniformes

**Squatinidae**

Acero P A, Tavera JJ, Anguila R, and Hernández L. 2016. A New Southern Caribbean Species of Angel Shark (Chondrichthyes, Squaliformes, Squatinidae), Including Phylogeny and Tempo of Diversification of American Species. *Copeia* 104:577-585. DOI 10.1643/CI-15-292.

Akyol O, Unal V, and Capape C. 2015. Occurrence and Biological Observations on Angel shark *Squatina squatina* (Chondrichthyes: Squatinidae) from the Turkish Waters (Eastern Mediterranean). *Turkish Journal of Fisheries and Aquatic Sciences* 15:931–935. DOI 10.4194/1303-2712-v15_4_17.

Ergenler A, Turan F, and Turan C. 2020. Occurrence of A Sawback Angelshark (*Squatina aculeata* Cuvier, 1829) from the Iskenderun Bay, North-Eastern Mediterranean Coast of Turkey. *Biharean Biologist* 14:57-59.

Ergüden D, Ayas D, Gürlek M, Karan S, and Turan C. 2019. First documented smoothback angelshark *Squatina oculata* Bonaparte, 1840 from the North-Eastern Mediterranean Sea, Turkey. *Cahiers de Biologie Marine* 60:189-194.

Vaz DFB, and Carvalho M, R., de. 2018. New Species of *Squatina* (Squatiniformes: Squatinidae) from Brazil, with Comments on the Taxonomy of Angel Sharks from the Central and Northwestern Atlantic. *Copeia* 106:144-160. DOI 10.1643/CI-17-606.

Yığın CÇ, İşmen A, Daban B, Cabbar K, and Önal U. 2019. Recent findings of rare sharks, *Squatina oculata* Bonaparte, 1840 and *Squatina squatina* (Linnaeus, 1758) from Gökçeada Island, Northern Aegean Sea, Turkey. *Journal of the Black Sea* 25:305–314.

## Multiple Groups

Al-Shajibi SR, Chesalin MV, and Al-Shagaa GA. 2014. New Records of Sharks from Southern Coastal Waters of Oman in the Arabian Sea. *Pakistan Journal of Zoology* 46:281–284.

Branstetter S, and McEachran JD. 1986. A First Record of *Odontaspis noronhai* (Lamniformes: Odontaspididae) for the Western North Atlantic, with Notes on Two Uncommon Sharks from the Gulf of Mexico. *Northeast Gulf Science* 8:1–8. DOI 10.18785/negs.0802.08.

Compagno LJV, and Smale MJ. 1986. Recent records of four warm-water elasmobranchs from the eastern Cape Province, South Africa. *South African Journal of Marine Science* 4:11–15. DOI 10.2989/025776186784461873.

González-Acosta AF, Rodiles-Hernández R, González-Diaz AA, and Mendoza-Carranza M. 2017. Notes on the Presence of *Mustelus sinus mexicanus* and *Hexanchus nakamurai* (Chondrichthyes: Elasmobranchii) in Mexican waters. *Journal of Aquaculture & Marine Biology* 5:1–6. DOI 10.15406/jamb.2017.05.00133.
