# Supplementary material for: Giant, swimming mouths: oral dimensions of extant sharks do not accurately predict body size in Dunkleosteus terrelli (Placodermi: Arthrodira)
Source: PeerJ. 2023 Apr 10;11:e15131. doi: 10.7717/peerj.15131 (PMC10100833; doi:10.7717/peerj.15131)
Supplement: Supplemental Information 10 [file peerj-11-15131-s010.pdf]

Case Western Reserve University

Russell Engelman <rke3@case.edu>

Coryphaena picture

11 messages

Richard Winterbottom <priolepis@gmail.com> Sun, Jul 31, 2022 at 10:50 AM
To: rke3@case.edu

Hi Russell,

I've attached both images I have of coryphaenids (both from the Seychelles). They are slightly better (i.e. larger) than the one(s) of FishBase. Hopefully you can get enough detail for your measurements. Unfortunately, both specimens were discarded after photography - a bit too big to add to my luggage on the return flight! The number after the specific name is the standard length of the fish in mm.

Let me know if there is anything else you need.

Rick.

2 attachments

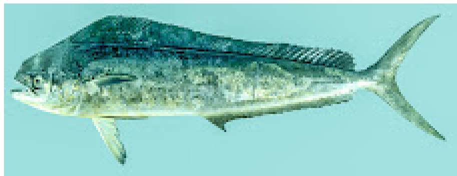

hippurus, 600, Seychelles, spec. discarded.jpg
311K

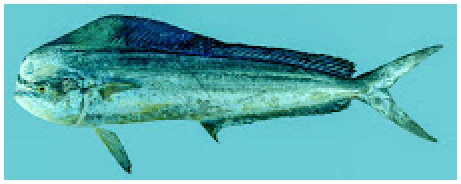

hippurus, 780 cms, Seychelles, LightRoom.jpg
303K

Russell Engelman <rke3@case.edu> Sun, Jul 31, 2022 at 12:01 PM
To: Richard Winterbottom <priolepis@gmail.com>

Dear Dr. Winterbottom,

Okay, thank you, this is a huge help.

Sincerely,
Russell
[Quoted text hidden]

Russell Engelman <rke3@case.edu> Sun, Jul 31, 2022 at 12:01 PM
To: Russell Engelman <neovenatoridae@gmail.com>

[Quoted text hidden]

---

2 attachments

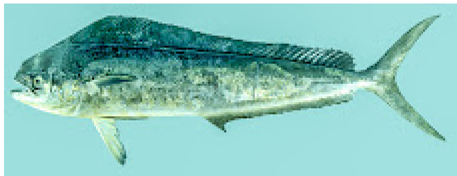

hippurus, 600, Seychelles, spec. discarded.jpg
311K

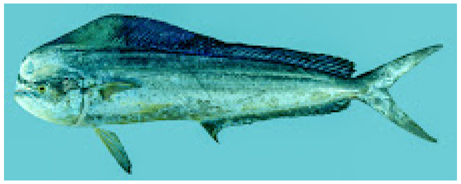

hippurus, 780 cms, Seychelles, LightRoom.jpg
303K

Russell Engelman <rke3@case.edu> Tue, Mar 7, 2023 at 8:02 PM
To: Richard Winterbottom <priolepis@gmail.com>

Dear Dr. Winterbottom,

Quick follow up question. Can I get your written permission to use a derivative of these images in a paper? I used a silhouette of them in a manuscript and the journal wants explicit permission from whoever took the original photos the silhouette was based on before going to proof.

Sincerely,
Russell
[Quoted text hidden]

Richard Winterbottom <priolepis@gmail.com> Tue, Mar 7, 2023 at 9:46 PM
To: Russell Engelman <rke3@case.edu>

Dear Russell,

Herewith my permission to use my image(s) in the manner in which you suggest. You can use a derivative image, a silhouette, or the images themselves in any paper you wish to. Either I, or the ROM, or both would appreciate an acknowledgement of the source, but it is not strictly necessary, and you can use any of the above without such acknowledgement.

Richard Winterbottom,
Curator Emeritus,
Royal Ontario Museum,

Toronto, Ontario
Canada.
[Quoted text hidden]

Russell Engelman <rke3@case.edu> Tue, Mar 7, 2023 at 10:53 PM
To: Richard Winterbottom <priolepis@gmail.com>

Since the specimen was disposed of, what is the best way to attribute it to the ROM? I say in the paper image provided by R. Winterbottom.

Russell
[Quoted text hidden]

Richard Winterbottom <priolepis@gmail.com> Wed, Mar 8, 2023 at 7:03 AM
To: Russell Engelman <rke3@case.edu>

That's good enough, Russell.
Rick.
[Quoted text hidden]

Russell Engelman <rke3@case.edu> Wed, Mar 8, 2023 at 10:50 PM
To: Richard Winterbottom <priolepis@gmail.com>

Dear Dr. Winterbottom,

PeerJ emailed me back, they said your permission via email is not enough and asked me to ask you to fill out this form for the image. I think it's a bit overkill given I just traced a silhouette off of it, but I think it's journal rules.

Sincerely,
Russell
[Quoted text hidden]

Photo-Video-Permission-Letter.docx
10K

Richard Winterbottom <priolepis@gmail.com> Thu, Mar 9, 2023 at 9:08 AM
To: Russell Engelman <rke3@case.edu>

Hi Russell,

I hate it when journals try to dictate to the people who provide their livelihood. And I am particularly suspicious of ones that try to get a copy of my signature - they could use that for other purposes. So I have a suggestion. Send me a copy of your tracing. I am sure I can find at least one difference between it and the photo. Then you can say your image is not identical to any photo you are aware of and therefore is an original work of art for which you personally own the copyright.

I'll be interested in the response!!!

Rick.
[Quoted text hidden]

Russell Engelman <rke3@case.edu> Thu, Mar 9, 2023 at 10:59 AM
To: Richard Winterbottom <priolepis@gmail.com>

Dear Dr. Winterbottom,

I agree. I even asked the journal if all this is even necessary given the image is clearly redrawn and not copied verbatim.

Here is the original image you gave me permission to use, and here is my modified silhouette. See if you can find any differences.

Sincerely,
Russell
[Quoted text hidden]

2 attachments

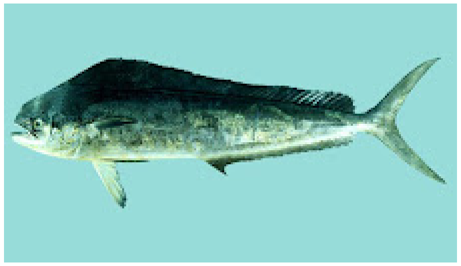

Coryphaena hippurus 60 SL Winterbottom.jpg
30K

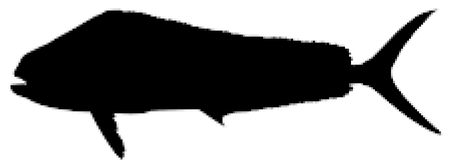

Coryphaena hippurus white background.png
15K

Richard Winterbottom <priolepis@gmail.com> Thu, Mar 9, 2023 at 2:31 PM
To: Russell Engelman <rke3@case.edu>

Hi Russell,

I think all that is needed is for you to smooth out the posterior margin of the pelvic fin (see below - oops, I simply copied your image to change it - do you think I have infringed on your copyright!!! 🙃 😛). Then you can claim that this is the image of a hypothetical fish. In my photo, the fin is slightly torn. You could also 'smooth away' the folded pelvic fin so that the abdominal outline is continuous rather than with a low bump just behind the extended pelvic fin. I would certainly back you up, although I cannot even begin to imagine anyone ever questioning it!!

Rick.

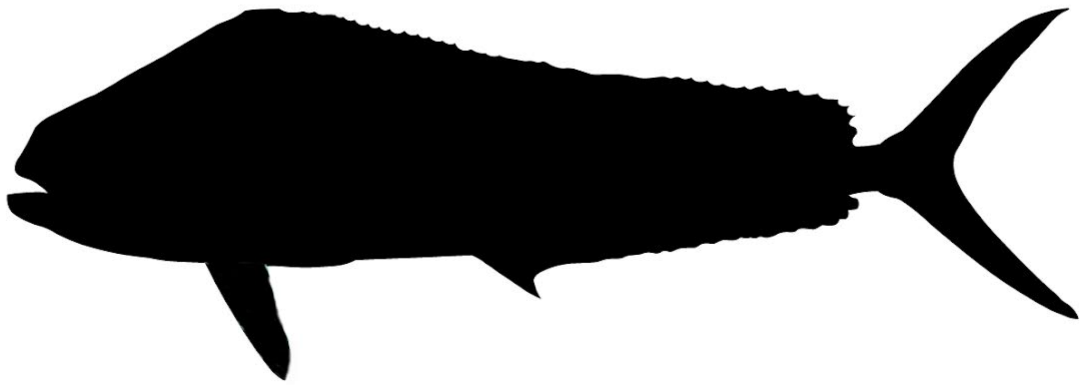

[Quoted text hidden]
